# Assessing and Responding to Palliative Care Needs in Rural Sub-Saharan Africa: Results from a Model Intervention and Situation Analysis in Malawi

**DOI:** 10.1371/journal.pone.0110457

**Published:** 2014-10-14

**Authors:** Michael E. Herce, Shekinah N. Elmore, Noel Kalanga, James W. Keck, Emily B. Wroe, Atupere Phiri, Alishya Mayfield, Felix Chingoli, Jason A. Beste, Listern Tengatenga, Junior Bazile, Eric L. Krakauer, Jonas Rigodon

**Affiliations:** 1 Department of Medicine, Division of Infectious Diseases, University of North Carolina School of Medicine, Chapel Hill, North Carolina, United States of America; 2 Abwenzi Pa Za Umoyo, Neno, Malawi; 3 Partners in Health, Boston, Massachusetts, United States of America; 4 Department of Global Health and Social Medicine, Harvard Medical School, Boston, Massachusetts, United States of America; 5 Department of Medicine, Brigham and Women’s Hospital, Boston, Massachusetts, United States of America; 6 Neno District Health Office, Neno District Hospital, Ministry of Health, Government of the Republic of Malawi, Neno, Malawi; 7 Department of Medicine, Division of Palliative Care, Massachusetts General Hospital, Boston, Massachusetts, United States of America; The James Cook University Hospital, United Kingdom

## Abstract

**Introduction:**

Palliative care is rarely accessible in rural sub-Saharan Africa. Partners In Health and the Malawi government established the Neno Palliative Care Program (NPCP) to provide palliative care in rural Neno district. We conducted a situation analysis to evaluate early NPCP outcomes and better understand palliative care needs, knowledge, and preferences.

**Methods:**

Employing rapid evaluation methodology, we collected data from 3 sources: 1) chart review of all adult patients from the NPCP’s first 9 months; 2) structured interviews with patients and caregivers; 3) semi-structured interviews with key stakeholders.

**Results:**

The NPCP enrolled 63 patients in its first 9 months. Frequent diagnoses were cancer (n = 50, 79%) and HIV/AIDS (n = 37 of 61, 61%). Nearly all (n = 31, 84%) patients with HIV/AIDS were on antiretroviral therapy. Providers registered 112 patient encounters, including 22 (20%) home visits. Most (n = 43, 68%) patients had documented pain at baseline, of whom 23 (53%) were treated with morphine. A majority (n = 35, 56%) had ≥1 follow-up encounter. Mean African Palliative Outcome Scale pain score decreased non-significantly between baseline and follow-up (3.0 vs. 2.7, p = 0.5) for patients with baseline pain and complete pain assessment documentation. Providers referred 48 (76%) patients for psychosocial services, including community health worker support, socioeconomic assistance, or both. We interviewed 36 patients referred to the NPCP after the chart review period. Most had cancer (n = 19, 53%) or HIV/AIDS (n = 10, 28%). Patients frequently reported needing income (n = 24, 67%) or food (n = 22, 61%). Stakeholders cited a need to make integrated palliative care widely available.

**Conclusions:**

We identified a high prevalence of pain and psychosocial needs among patients with serious chronic illnesses in rural Malawi. Early NPCP results suggest that comprehensive palliative care can be provided in rural Africa by integrating disease-modifying treatment and palliative care, linking hospital, clinic, and home-based services, and providing psychosocial support that includes socioeconomic assistance.

## Introduction

Palliative care encompasses assessment and relief of suffering of all kinds including pain, other physical and psychological symptoms, and social distress along the entire course of the patient’s disease process [1]. Palliative care has been shown to improve patient outcomes, including mortality, when integrated with disease-modifying therapy [2]. The integration of palliative care with routine treatment for HIV/AIDS, cancer and other serious chronic illnesses is an important strategy for increasing access to palliative care and improving the quality of health care [3]–[6]. Despite the documented benefits, however, access to comprehensive, integrated palliative care remains limited in most developing countries, where the global burden of chronic disease is greatest [7], [8].

Worldwide, 80% of patients lack access to adequate pain control–the cornerstone of palliative care [9]. In sub-Saharan Africa (SSA), only 5% of people in need of palliative care receive it despite the growing availability of palliative care programs [10]. The demand for palliative care in SSA is expected to increase over the next 20 years as more persons living with HIV (PLHIV) access life-prolonging antiretroviral therapy (ART) and develop long-term complications of HIV disease, and as non-communicable diseases (NCDs), including cancer, become more prevalent [10]–[12].

Malawi, a densely populated sub-Saharan African country with low per capita health expenditure, offers palliative care services at 21 sites–a fraction of the over 600 public and non-governmental health facilities located throughout the country [13], [14]. Despite recent increases in palliative care service availability, major challenges remain in making these services accessible to all Malawians in need, particularly in rural areas. Geographical coverage of palliative care remains concentrated in urban and peri-urban locales, often out of reach for the 80% of the population who reside in less densely populated areas [15]–[18]. In rural settings where health infrastructure and human resources are limited, HIV and NCDs strain the capacity of health systems to deliver palliative care services. Based on recent estimates, over 1 million Malawians are living with HIV/AIDS and more than 25,000 have cancer [13], [19], [20]. Many Malawian patients and their family caregivers endure these illnesses at home–where they may prefer to receive care. Yet their needs, knowledge, and preferences regarding palliative care are not well characterized.

Qualitative research provides insight into palliative care needs, knowledge, and preferences, identifies causes of suffering, and guides local responses to recognized needs [16], [21]–[25]. Available evidence from SSA indicates that the greatest needs of patients facing serious chronic illnesses and their caregivers may be psychosocial with basic socioeconomic necessities–including food, adequate shelter, and help with school fees–featuring prominently [1], [16]. However, few published accounts describe palliative care needs, knowledge, and preferences in rural areas [1], [26]–[27]. Fewer still detail efforts to translate identified needs into programmatic action and describe effective models of palliative care service delivery, particularly community- and home-based models that integrate palliative care with health services relevant to the SSA context, including treatment for HIV/AIDS [26], [28]–[29].

Since 2007, Partners In Health (PIH), a non-governmental organization based in Boston, USA, has partnered with its Malawian sister organization, Abwenzi Pa Za Umoyo (APZU), and the Malawi Ministry of Health (MoH) to provide comprehensive healthcare to the people of Neno, a rural and impoverished district in the country’s Southern region. In early 2012, APZU/PIH launched the Neno Palliative Care Program (NPCP) in order to integrate pain and symptom relief and psychosocial support with disease modifying treatment for patients with serious chronic illnesses and their families. As part of APZU/PIH’s ongoing palliative care work, we conducted a situation analysis of the palliative care needs, knowledge, preferences, and services available in Neno district, aiming to improve the NPCP in a manner responsive to identified needs. We report here the results of the situation analysis, including early outcomes from the NPCP.

## Methods

We performed a situation analysis employing rapid evaluation methodology (REM). First developed by the World Health Organization (WHO), REM involves collecting data through field-based interviews and observations to assist ministries of health and implementing partners in rapidly and efficiently taking programmatic action [30]. In SSA, REM has been used to evaluate health services, including palliative care services, and to guide quality improvement efforts [16], [30]–[32].

### Setting

**Figure 1:**
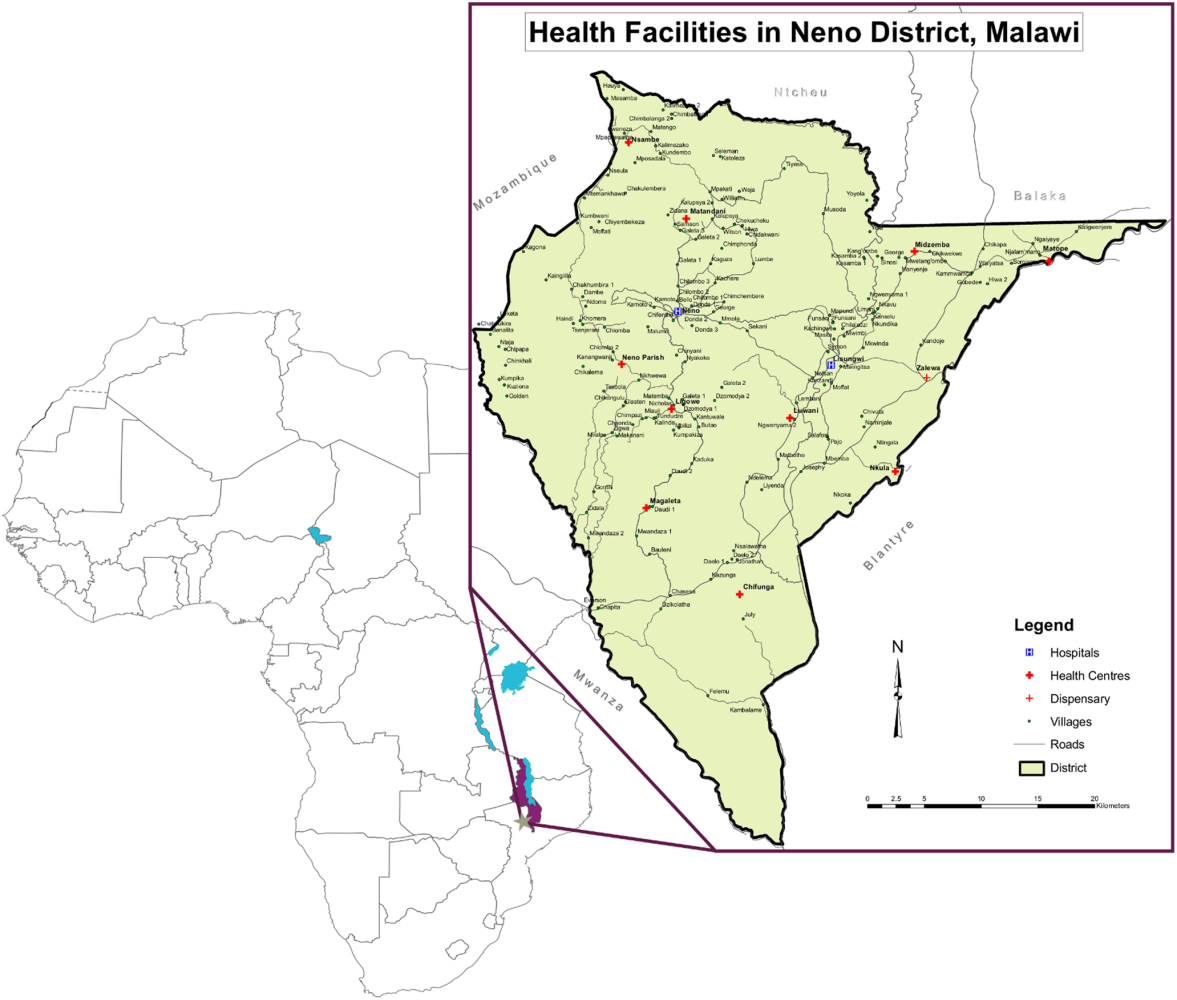
Map of Neno District, Republic of Malawi. The inset picture depicts Neno district, located in the southwestern portion of the country. The district shares an international border with Mozambique to the west and borders with Ntcheu district to the north; Balaka and Zomba districts to the northeast; Blantyre district to the east; Chikwawa to the south; and Mwanza district to the southwest. The inset legend illustrates the locations of major health facilities, including the district’s two hospitals–Neno District Hospital and Lisungwi Community Hospital–as well as the remaining 11 health centers.

We carried out the situation analysis in Neno district, Malawi. Neno district, with a population of 130,612, is a rural, hard-to-reach area bordered by Mozambique to the West and six Malawi districts along its remaining boundaries (Figure 1) [33]. The district’s main health and economic indicators are representative of national averages, with male and female life expectancy slightly higher in Neno at 48.6 and 53.1 years, respectively, than Malawi overall, and 56% of the population living below the World Bank poverty line of 116 USD of income per person per year [33], [34]. The Malawi Ministry of Health, through the Neno District Health Office (DHO), provides essential health services free of charge at 13 public health facilities, including Neno District Hospital situated in the administrative capital of the district–Neno boma [33].

### Data Collection Overview

Employing REM, we collected data from three principal sources: 1) chart review of paper-based and electronic medical records for adult palliative care patients enrolled in the NPCP during its first 9 months; 2) structured interviews with patients and their caregivers; and 3) semi-structured interviews with key stakeholders. Collecting data from three sources enabled verification of information through triangulation [25], [35]. We defined caregivers to be any accompanying family member, friend, or life partner identified by the patient as their primary custodian or “guardian” at the time of NPCP enrollment. We defined key stakeholders as frontline health workers and leaders of organizations involved in any aspect of palliative care delivery in Neno district or those involved in palliative care policy at the national level.

### Chart Review

We reviewed paper-based medical records and registers for all adult patients enrolled in the NPCP during its first 9 months of existence (mid-January through early October 2012). We assessed the first 9 months to better understand our patient population and referral practices and to evaluate the quality of service delivery at the start of the program. We used the APZU OpenMRS-based electronic medical record to augment data abstracted from paper-based records [36]. We collected de-identified data on the following patient dimensions: demographic information, primary diagnosis, HIV status, pain, other symptoms, psychosocial distress, encounter location (i.e. in the outpatient clinic, hospital ward, and/or home visit in the patient’s home), retention-in-care, and referral for psychosocial support services. Palliative care providers documented the severity and quality of patient pain, other symptoms, and psychosocial distress at the time of each clinical encounter using a visual hand-scale representation of the African Palliative Care Association (APCA) African Palliative Outcome Scale (African POS) [37]–[40]. The African POS is a multidimensional palliative care scale designed for and validated in African palliative care populations [38], [39]. Responses to African POS questions are scored using a 6-point Likert scale ranging from 0 to 5 [39]. When used to assess pain in adults, an African POS score of 3 or greater suggests moderate-to-severe pain, with a score of 5 representing the most severe or worst pain.

### Structured Interviews: Patients and Caregivers

We developed two interview instruments–one for patients and a second for caregivers–to qualitatively assess the following participant domains: 1) awareness of diagnosis and preferences for palliative care; 2) pain and other physical symptoms; and 3) psychological, social, and spiritual needs and sources of support. Both instruments incorporated structured items and open-ended questions with interviewee prompts. The patient and caregiver interview instruments included all items intended for patients and caregivers, respectively, of the African POS [37], [39]. The African POS has been used to measure patient outcomes on the three domains enumerated above; to evaluate caregiver knowledge, confidence, and anxiety; and to assess the quality of palliative care services [37]. The structured interview instrument was translated from English into the local language of patient and caregiver participants, Chichewa. The translation and preparation of the final Chichewa version of the instrument were performed at the APZU field office by trained local staff fluent in both English and Chichewa.

We planned to interview a sample of 80 individuals, comprising 5 patient participants at each of 4 antiretroviral therapy clinics (20 total), 10 hospitalized patients, and 10 patients receiving chemotherapy for Kaposi sarcoma, along with a matching number of accompanying caregivers (i.e. 40 caregiver participants total). We devised this sampling strategy in an attempt to capture a diverse range of patient and caregiver perspectives regarding palliative care services and experiences with serious chronic illness in Neno, Malawi. With this strategy in place, we recruited and interviewed a consecutive sample of patients and their caregivers 18 years of age or older who presented for routine HIV care, in-patient hospital care, or chemotherapy for Kaposi sarcoma, and who were newly referred for enrollment in the NPCP at 4 public health facilities in Neno District on selected days from January 14 to February 27, 2013. The facilities included one district hospital, one community hospital, and two health centers, chosen to represent the breadth of facility-based healthcare delivery in the district. We approached potential participants face-to-face at health facilities to maximize the efficiency of the recruitment process given the accessibility of the target population at health facilities and the cost and logistical limitations involved with alternative recruitment approaches (e.g. telephone, paper flier, etc.). While study investigators helped recruit interview participants, they had not met any participants prior to the time of the interview. Recruitment was stopped at 47 participants (36 patient and 11 caregiver participants) after an interim analysis revealed that saturation of major themes had been achieved. No patients or caregivers declined study participation.

Study investigators with formal medicine and public health training (S.N.E., E.B.W., and A.M.) conducted patient and caregiver interviews with the assistance of an independent, trained study interpreter fluent in both English and Chichewa. We obtained written informed consent from all participants prior to commencing interviews. As part of the informed consent process and prior to the structured interviews, study investigators (S.N.E., E.B.W., and A.M.) explained the rationale for the study but did not disclose any personal information regarding their interests in the research topic or their assumptions about possible study findings. Patient and caregivers were interviewed separately in the absence of the other person, whenever possible. However, at the discretion of patient participants, caregivers were allowed to be present during interview sessions and vice versa. All interviews were conducted in private areas at one of the four health facilities described above in a single session lasting approximately 30 to 45 minutes. An investigator led each interview session and read questions aloud sequentially, allowing enough time to fully capture participant responses and adhering to the format of the interview instrument as it progressed through the following sections: 1) Patient demographics, knowledge about their diagnosis and treatment, and medical care preferences; 2) African POS items; and 3) Psychosocial support and relevant information–needs, preferences, and ongoing sources of support (Appendix S1). For each participant, the study interpreter translated all questions aloud into the local language, Chichewa, and subsequently translated participant responses into English. The investigator then transcribed English-translated responses verbatim. No audiovisual recording equipment was used during interviews because of local restrictions on the use of such technology in patient care areas. No repeat interviews were carried out at any time during the study period.

### Semi-structured Interviews: Key Stakeholders

We interviewed a purposive sample of key local and national stakeholders, including at least one individual from all organizations in Neno district working in the palliative care arena. The specific sites were as follows: 1) an integrated cancer and palliative care clinic at Neno District Hospital; 2) a MoH ART clinic; 3) the Neno District Health Office home-based care program; 4) the APZU/PIH Program on Social and Economic Rights (POSER); 5) the APZU/PIH Village Health Worker Program; 6) Chiyembekezo Community-based Organization (CBO); and 7) the Palliative Care Association of Malawi (PACAM).

Stakeholders were interviewed at each site by one of the study investigators (S.N.E.) and asked to discuss the palliative care infrastructure and service delivery practices at their institution or organization. Transcripts were reviewed with key stakeholder participants for comment and correction. Palliative care policy documents, clinical encounter forms, data recording tools, and training materials were reviewed by the investigator prior to stakeholder interviews to provide context, among them the *NPCP Initial Assessment Form* (Appendix S2), *Malawi National Palliative Care Guidelines* (Appendix S3), the *Introduction to Palliative Care: Health Care Workers’ Service Providers Manual* (Appendix S4), and the *Malawi Guidelines for the Clinical Management of HIV in Children and Adults* (Appendix S5) [13], [41]–[43]. To minimize the influence of palliative care documents on stakeholder responses, these materials were not provided to stakeholders before or during study interviews. Preliminary study findings were shared at a dissemination meeting to refine our understanding and representation of perspectives expressed by key stakeholders during interviews.

### Data Analysis and Reporting

For qualitative data we employed content analysis methodology to develop thematic codes for patient, caregiver, and stakeholder interview responses using Dedoose software (SocioCultural Research Consultants, LLC, Manhattan Beach, CA, USA) [44], [45]. Two study investigators (S.N.E., E.B.W.) coded participant responses together (beginning with “participant 0” as designated automatically by Dedoose software) and reconciled differences by consensus. We calculated frequencies and simple proportions for all thematic codes generated, including root and child codes. We then used this information to develop a taxonomy of themes, providing a structure to interpret patient and caregivers’ experiences with illness and to describe palliative care knowledge and preferences among all interview participants. The results of structured interviews are reported in accordance with accepted criteria for qualitative research [46]. For quantitative survey and chart review data we calculated frequencies, proportions, means or medians, and measures of dispersion. For continuous variables, we compared means between two independent groups using the two-sample t-test and means of repeated measurements in the same group using the paired t-test. For variables with more than two categories, we compared means with the one-way analysis of variance. We considered a two-sided p-value<0.05 to be statistically significant. All quantitative analyses were performed in SPSS (Version 20, 2011, IBM, Armonk, NY, USA) or Stata (Version 12.1, 2011, StataCorp, College Station, TX, USA).

### Ethics Statement

All components of this study were conducted in accordance with the principles expressed in the Declaration of Helsinki. All patient and caregiver interview participants provided written informed consent prior to participation in the study. All key stakeholder interview participants, who were asked generic questions about palliative care programs and policies only and not about themselves or individual patients, provided verbal informed consent, which was documented on de-identified semi-structured interview instruments. All individuals depicted in this manuscript are NPCP service providers, not study participants, and have given written informed consent (as outlined in the PLOS consent form) to publish their likeness in photograph form. Human subjects research approval for all study procedures was granted by the National Health Sciences Research Committee of Malawi and by the Institutional Review Boards of the University of North Carolina, Chapel Hill, USA, Partners Healthcare System, Boston, USA and Harvard Medical School, Boston, USA.

## Results

### Overview

**Figure 2:**
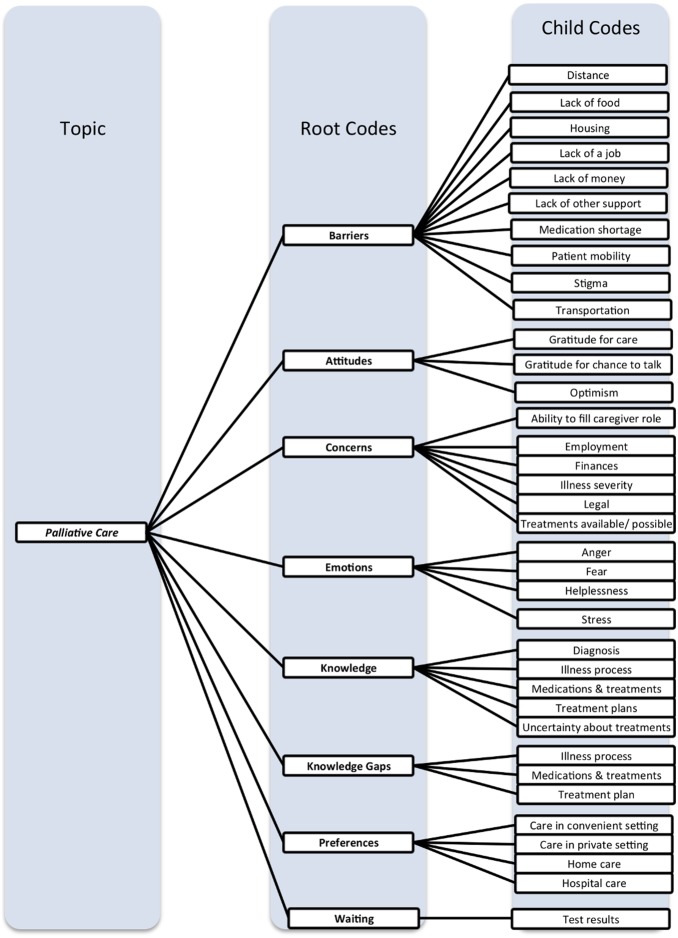
Major thematic codes generated from patient and caregiver interviews. The coding tree depicts major themes identified from structured patient and caregiver interviews. Codes relate to patient and family caregivers’ experiences with illness, palliative care services provided, and palliative care services desired. Subordinate child codes elaborate descriptions for each root code. All root and child codes were generated using content analysis methodology and Dedoose software (SocioCultural Research Consultants, LLC., Manhattan Beach, CA, USA).

We first describe the Neno Palliative Care Program (NPCP) model and early results from the NPCP. We then report major characteristics of patient and caregiver interview participants. Lastly, we present the results of patient, caregiver, and key stakeholder interviews, organized by our taxonomy of major and minor interview themes (Figure 2).

### The Neno Palliative Care Program Model

The joint MoH–APZU/PIH Neno palliative care team, first formed in late 2011 to implement the NPCP, consists of: 1) 1 physician; 2) 2 clinical officers (mid-level health workers common in Malawi); 3) 1 palliative care coordinator; 4) 1 nurse; 5) approximately 150 MoH home-based care volunteers; and 6) 691 community health workers, known locally as village health workers (VHWs), paid and trained by APZU/PIH to provide health-related services throughout the district. An experienced international palliative care physician-educator provides longitudinal training and clinical mentorship to NPCP clinical providers [47], [48]. All clinical providers received a week-long palliative care training in early January 2012 prior to NPCP implementation, followed by a week-long advanced training in March 2012.

**Figure 3:**
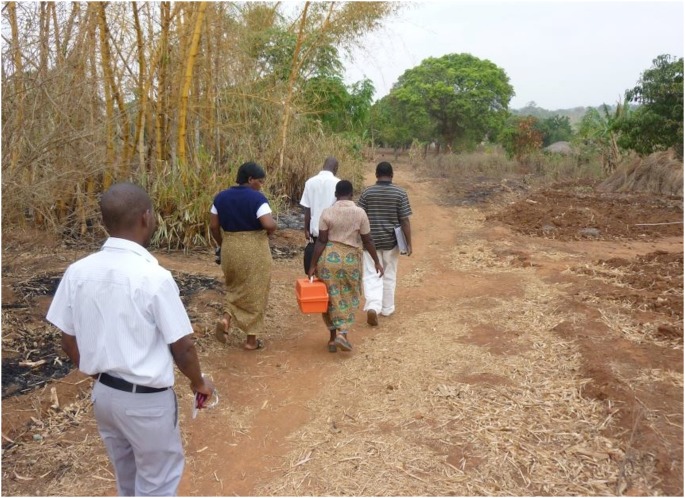
Neno Palliative Care Program team members conduct a home visit in a rural village. Five members of the Neno Palliative Care Program (NPCP) team travel by foot to reach the home of a palliative care patient living in rural Neno district, Malawi. An Abwenzi Pa Za Umoyo/Partners In Health Medical Officer with specialized training in palliative care (foreground) leads the home visit. He is accompanied by (from left to right) a palliative care nurse, a Program on Social and Economic Rights social worker, a Village Health Worker and a NPCP clerk. NPCP team members work together to assess and treat patient pain and other physical symptoms, provide disease-specific care for serious chronic illnesses, and offer psychosocial support to patients and their families.

NPCP clinical providers train VHWs to recognize pain, other symptoms, treatment side effects, major disease complications, and psychosocial distress, and to request an immediate home visit for patients with one of these issues (Figure 3). The palliative care clinical team manages a weekly outpatient clinic at Neno District Hospital, conducts daily inpatient hospital consultations, and oversees a weekly district-wide mobile outreach clinic. Clinical officers working closely with the supervising palliative care physician conduct clinical evaluations, administer disease-modifying chemotherapy for Kaposi sarcoma, and prescribe symptom-relieving medications (including morphine), following MoH guidelines and the three-step WHO pain relief ladder [13], [40], [41], [43], [49]. The Malawi MoH, with support from APZU/PIH, procures essential palliative care medicines and supplies for the NPCP and all district health facilities–including morphine liquid, slow-release morphine tablets, codeine phosphate tablets, ibuprofen, amitriptyline, diazepam, anti-emetics, antibiotics, incontinence pads, sterile gauze, and bandages, among other commodities–in accordance with local regulations, including the Malawi Controlled Drug Act [13]. MoH and APZU/PIH health workers and programmatic staff refer patients to the NPCP from patient support programs and health facilities located throughout Neno district, including the wards and clinical departments of the district’s two hospitals–Neno District Hospital and Lisungwi Community Hospital (Figure 1). At the discretion of the referring health worker, patients may start analgesia before or at the time of NPCP referral, or during the first NPCP encounter. For patients living with HIV, clinical officers provide integrated HIV treatment and primary care services, including assessment for ART initiation and evaluation for possible ART side effects in accordance with national guidelines [43].

The NPCP responds to patient and family psychosocial needs through the APZU/PIH Program on Social and Economic Rights (POSER). POSER provides counseling and emotional support, as well as socioeconomic assistance for selected patients, in partnership with local community-based and faith-based organizations and government Ministries, including the Ministry of Gender, Child Development and Community Development and the Neno Office of Social Welfare. POSER staff evaluate the vulnerability of referred patients using a standardized assessment tool, and provide qualifying patients with one or more of the following types of socioeconomic assistance: food packages, home repairs, transportation reimbursement, and school support for children.

### Early Results from the Neno Palliative Care Program

Over a 9-month period from mid-January through early October 2012, the NPCP enrolled 63 patients with serious chronic illnesses (Table 1). Most patients had cancer as a primary diagnosis (n = 50, 79%), and 32 of these (64%) had Kaposi sarcoma (Table 1). The majority of patients were HIV-infected (37 of 61, 61%). Of those with HIV infection, 31 (84%) lived in Neno district and received ART at 1 of the district’s 13 public clinics.

**Table 1:** Characteristics of patients at enrollment in the Neno Palliative Care Program (NPCP, January through October 2012).

Characteristic		n/N (%)
**Total cohort**		63/63 (100)
**Gender**		
	Male	37/63 (59)
**Age (years)**		
	Mean (standard error, SE)	48 (SE: 2.2)
**Primary diagnoses**		
Cancer		
	All cancers	50/63 (79)
	Kaposi sarcoma	32/50 (64)
	Cervical	7/50 (14)
	Other malignancy	11/50 (22)
Stroke		5/63 (8)
Cirrhosis		3/63 (5)
Peripheral Neuropathy		2/63 (3)
Other*		3/63 (5)
**HIV** § **status** ¶		
	Sero-positive	37/61 (61)
**HIV care**		
Enrollment status		
	Documented in HIV care and receiving ART• in Neno District	31/37 (84)
	No documentation of HIV care	6/37 (16)#

*Includes the following conditions: anal fissure, paraplegia, and tuberculosis.

§ HIV = Human immunodeficiency virus.

¶ Documented HIV sero-status is missing for 2 patients.

• ART = Antiretroviral therapy.

# For 3 of 6 patients documentation could not be verified because patients presented for palliative care from districts other than Neno district.

Neno palliative care providers conducted 112 patient encounters during the 9-month evaluation period. Most encounters occurred in the outpatient palliative care clinic based at Neno District Hospital (n = 57, 51%) (Table 2). NPCP providers conducted 22 home visits, comprising 20% of all patient encounters. Complementing clinical service delivery, APZU/PIH VHWs provided longitudinal psychosocial support to 35 (56%) patients in the form of counseling during regularly scheduled home visits. Similarly, 34 (54%) patients were referred to POSER for psychosocial support, which included emotional support and socioeconomic assistance (Table 2). In total, providers referred 48 (76%) patients for psychosocial services in the form of VHW home visits, POSER support, or both.

**Table 2:** Summary of palliative care services provided by the Neno Palliative Care Program (NPCP, January through October 2012).

NPCP Services		n/N (%)
**Clinical**		
Total encounters		112/112 (100)
Patient encounter setting*		
	Outpatient Clinic	57/112 (51)
	Hospital	32/112 (29)
	Patient Home Visit	22/112(20)
**Non-Clinical** §		
	APZU/PIH Village Health Worker assigned	35/63 (56)
	Referred for psychosocial services (includes socioeconomic assistance)¶	34/63 (54)

*Setting information is missing for 1 patient encounter.

§ Describes non-clinical services provided to the 63 patients enrolled in the NPCP from January through October 2012. Non-clinical services were provided by the Abwenzi Pa Za Umoyo (APZU)/Partners In Health (PIH) Village Health Worker program and Program on Social and Economic Rights in collaboration with the Ministry of Health, Ministry of Gender, Child Development and Community Development, and the Neno Office of Social Welfare.

¶ In addition to counseling and emotional support, psychosocial services include provision of in-kind support (soap, water collection receptacles, new shoes, etc.), food packages, housing repairs, and school tuition payments and school supplies for patients’ children.

Overall, 56 (89%) patients had a documented baseline pain assessment from their initial NPCP encounter, which included a record of the presence and severity of pain (Table 3). Among these 56 patients, 13 (23%) patients were found to be pain free (i.e. African POS score = 0) and 43 (77%) reported the presence of *any* pain (i.e. African POS score>0) (Table 3). Among the 43 patients reporting any pain at baseline, the mean African POS pain score was 3.2 (standard error, SE: 0.2), and 31 (72%) patients reported moderate-to-severe pain (i.e. African POS score≥3). Of the 43 patients with documented pain at baseline, 35 (81%) had a cancer diagnosis. Patients with documented baseline pain and cancer had a significantly higher mean African POS pain score than those without cancer (3.3 with cancer vs. 2.4 without cancer, p = 0.04) (Table 3). Among patients with baseline pain, pain severity did not differ significantly by age, gender, HIV status, or presence of Kaposi sarcoma (Table 3).

**Table 3:** Patient pain status, severity, and management at the initial Neno Palliative Care Program (NPCP) encounter (January through October 2012).

Variable		n/N (%)	Mean African POS*Pain Score§ (SE)¶	p-value
**Clinical status at initial encounter** •				
	Pain assessed & documented	56/63 (89)		
	Pain free#	13/56 (23)		
	Any pain**	43/56 (77)	3.2 (0.2)	
	Moderate-to-severe pain§§	31/43 (72)	3.8 (0.1)	
**Pain severity at initial encounter for patients with documented pain, by patient characteristic**				
Age				0.8
	<50	24/43 (56)	3.2 (0.3)	
	≥50	19/43 (44)	3.1 (0.2)	
Gender				0.6
	Male	25/43 (58)	3.2 (0.2)	
	Female	18/43 (42)	3.1 (0.3)	
HIV status¶¶				0.5
	Sero-positive	26/41 (63)	3.2 (0.3)	
	Sero-negative	15/41 (37)	2.9 (0.3)	
Cancer				0.04
	With Cancer Diagnosis	35/43 (81)	3.3 (0.2)	
	Without Cancer Diagnosis	8/43 (19)	2.4 (0.3)	
Kaposi sarcoma (KS)				0.3
	With KS Diagnosis	21/43 (49)	3.4 (0.3)	
	Without KS Diagnosis	22/43 (51)	3.0 (0.2)	
Analgesia at NPCP intake				0.4
	None	14/43 (33)	2.9 (0.3)	
	Any analgesia••	29/43 (67)	3.3 (0.2)	
	Non-opioids	13/43 (30)	3.0 (0.4)	
	Weak opioids (e.g. codeine)	2/43 (5)	3.5 (1.5)	
	Strong opioids (e.g. morphine)	14/43 (33)	3.5 (0.3)	
**Pain management during initial encounter for patients with documented pain**				
Provider prescribing actions				0.6
	Started analgesia	10/43 (23)	2.9 (0.4)	
	Adjusted analgesia	15/43 (35)	3.2 (0.4)	
	Continued previously prescribed analgesia	9/43 (21)	3.6 (0.4)	
	No documentation of analgesia prescription	9/43 (21)	3.0 (0.3)	
Morphine				<0.001
	Morphine prescribed	23/43 (53)	3.7 (0.2)	
	Other analgesia prescribed	11/43 (26)	2.1 (0.3)	

*African POS = African Palliative Care Association African Palliative Outcome Scale.

§ Pain measured using the African POS, which scores pain in 1-point increments from 0 to 5 on a 6-point Likert scale. Harding R, Selman L, Agupio G, Dinat N, Downing J, et al. (2010) Validation of a core outcome measure for palliative care in Africa: the APCA African Palliative Outcome Scale. Health and Quality of Life Outcomes 8∶10. doi:10.1186/1477-7525-8-10.

¶ SE = standard error.

• Documented baseline pain assessment at initial encounter is missing for 7 patients.

# Indicated by African POS = 0.

**Indicated by African POS>0.

§§ Indicated by African POS≥3.

¶¶ Documented HIV sero-status is missing for 2 patients.

•• Includes any non-opioid (e.g. ibuprofen), weak opioid (e.g. codeine), or strong opioid (e.g. morphine) prescribed by the referring health worker prior to NPCP enrollment.

Among the 43 patients with documented pain at NPCP intake, 29 (67%) reported ongoing use of pain medications initiated by a referring health worker (Table 3). Of these 29 patients: 13 (45%) described using non-opioid analgesics exclusively, such as ibuprofen, paracetamol, aspirin, amitriptyline or some combination thereof; 2 (7%) reported taking codeine–a weak opioid–with or without an accompanying non-opioid agent; and 14 (48%) reported using oral morphine–a strong opioid–with or without a co-administered non-opioid medication (Table 3).

For the 43 patients with documented pain at baseline, NPCP providers newly started 10 (23%) patients on analgesia, added a drug or changed the current analgesic regimen for 15 (35%) patients, and continued the previously prescribed pain medications in 9 (21%) patients; 9 (21%) patients had no documentation of receiving a prescription for an analgesic agent during their initial NPCP encounter (Table 3). Morphine–available free of charge from the Malawi MoH–was prescribed for 23 of 43 (53%) patients with documented baseline pain. Having documented moderate-to-severe baseline pain was significantly associated with receiving a prescription for morphine at the initial NPCP encounter (p = 0.001). The mean African POS pain score was significantly higher for patients prescribed morphine (3.7, SE: 0.2) than for patients prescribed codeine or a non-opioid analgesic (2.1, SE: 0.3) (p<0.001) (Table 3).

Most patients (n = 35, 56%) had at least one recorded follow-up encounter (Table 4). Of these, 26 (74%) patients had a documented pain assessment, with 5 (19%) noted to be pain free and 21 (81%) reporting *any* pain (Table 4). Among the 21 patients reporting any pain at follow-up, the mean African POS pain score was 2.9 (SE: 0.3) and 11 (52%) patients reported moderate-to-severe pain. Pain severity did not differ significantly by age, gender, HIV status, or the presence of cancer, including KS, for patients reporting pain at follow-up (Table 4). For patients with pain at baseline and documented pain assessment at enrollment and follow-up (n = 18), we observed a small, non-significant decrease in mean African POS pain score (3.0 vs. 2.7, p = 0.5) over the follow-up interval. We recorded 4 deaths (6%) that occurred before the patient’s first scheduled follow-up encounter, each transpiring within 1 month of program enrollment (Table 4).

**Table 4:** Patient pain status, severity, and management at the first follow-up Neno Palliative Care Program (NPCP) encounter (May through October 2012).

Variable	n/N (%)	Mean African POS*Pain Score§ (SE)¶	p-value
**Patient follow-up**			
≥1 Follow-up NPCP encounter	35/63 (56)		
Patient died before first follow-up (within 1 month of initial encounter)	4/63 (6)		
**Clinical status at first follow-up encounter**			
Pain assessed & documented•	26/35 (74)		
Pain free#	5/26 (19)		
Any pain**	21/26 (81)	2.9 (0.3)	
Moderate-to-severe pain§§	11/21 (52)	4.0 (0.2)	
**Pain severity at first follow-up encounter for patients with documented pain, by patient characteristic** ¶¶			
Age			0.1
<50	13/21 (62)	2.5 (0.3)	
≥50	8/21 (38)	3.5 (0.5)	
Gender			0.6
Male	15/21 (71)	2.8 (0.3)	
Female	6/21 (29)	3.2 (0.6)	
HIV status••			0.6
Sero-positive	13/20 (65)	3.0 (0.5)	
Sero-negative	7/20 (35)	2.7 (0.3)	
Cancer			0.5
With Cancer Diagnosis	17/21 (81)	3.0 (0.3)	
Without Cancer Diagnosis	4/21 (19)	2.5 (0.9)	
Kaposi sarcoma (KS)			0.5
With KS Diagnosis	11/21 (52)	2.7 (0.3)	
Without KS Diagnosis	10/21 (48)	3.1 (0.5)	
**Pain management during first follow-up encounter for patients with documented pain** ¶¶			
Morphine			0.4
Morphine prescribed	14/21 (67)	3.1 (0.3)	
Other analgesia prescribed##	7/21 (33)	2.6 (0.5)	

*African POS = African Palliative Care Association African Palliative Outcome Scale.

§ Pain measured using the African POS, which scores pain severity in 1-point increments from 0 to 5 on a 6-point Likert scale. Harding R, Selman L, Agupio G, Dinat N, Downing J, et al. (2010) Validation of a core outcome measure for palliative care in Africa: the APCA African Palliative Outcome Scale. Health and Quality of Life Outcomes 8∶10. doi:10.1186/1477-7525-8-10.

¶ SE = standard error.

• Documented follow-up pain assessment at first return encounter is missing for 9 patients.

# Indicated by African POS = 0.

**Indicated by African POS>0.

§§ Indicated by African POS≥3.

¶¶ For the 21 patients with documented pain at the first follow-up NPCP encounter.

•• Documented HIV sero-status is missing for 1 patient.

## Includes 2 patients without documented analgesia prescription with mean African POS pain score of 2.

### Characteristics of Patient and Caregiver Interview Participants

We interviewed 36 patients at the time of NPCP enrollment who were not in the early NPCP cohort, including 9 patients interviewed in the presence of their caregiver. Patients interviewed were, on average, 38 years old and 19 (53%) were female (Table 5). Most patients interviewed had cancer (n = 19, 53%), with Kaposi sarcoma being the most prevalent diagnosis in the sample (n = 13, 36%). HIV/AIDS was the primary diagnosis for 10 (28%) patients, with every known HIV-infected patient interviewed already enrolled in MoH HIV care. Those surveyed reported being ill for about 1 year, on average (median: 13 months; interquartile range, IQR: 0–156 months), at the time of the interview. Several patients (n = 7, 19%) were unable to work due to illness.

**Table 5:** Characteristics of patient and caregiver interview participants.

Characteristic		Patients (N = 36)	Caregivers (N = 11)
		n/N (%)	n/N (%)
**Gender**			
	Female	19/36 (53)	8/11 (73)
**Age (years)**			
	Mean (standard error, SE)	38 (SE: 1.8)	47 (SE: 5.1)
**Highest Education Level Completed**			
	None	8/36 (22)	3/11 (27)
	Primary	17/36 (47)	7/11 (64)
	Secondary	10/36 (28)	1/11 (9)
	University	1/36 (3)	0/11 (0)
**Marital Status**			
	Married	21/36 (58)	10/11 (91)
	Divorced	8/36 (22)	0/11 (0)
	Widowed	4/36 (11)	1/11 (9)
	Single	2/36 (6)	0/11 (0)
	Living as Married	1/36 (3)	0/11 (0)
**Household Size**	Median (IQR)*	6 (2–10)	6 (3–13)
**Living Children per Household**	Median (IQR)	3 (0–8)	4 (1–6)
**Employment Status**			
	Subsistence farmer	12/36 (33)	5/11 (45)
	Market vendor	7/36 (19)	4/11 (36)
	Unable to work due to illness	7/36 (19)	0/11 (0)
	Part-time unskilled labor	4/36 (11)	1/11 (9)
	Part-time skilled labor	4/36 (11)	0/11 (0)
	Unable to find work	2/36 (6)	1/11 (9)
**Patient Primary Diagnosis**			
HIV/AIDS§		10/36 (28)	
	In HIV care, on ART¶	9/10 (90)	
	In HIV care, HCC• Clinic	1/10 (10)	
Cancer		19/36 (53)	
	Kaposi sarcoma	13/36 (36)	
	Cervical cancer	4/36 (11)	
	Other malignancy	2/36 (6)	
Other serious chronic illness#		7/36 (19)	
**Illness Duration (months)**	Median (IQR)	13 (0–156)	
**Caregiver Present at Visit/Hospital** **		12/36 (33)	
**Caregiver Relationship to Patient**			
	Parent		3/11 (27)
	Spouse		3/11 (27)
	Sibling		3/11 (27)
	Other family		2/11 (18)
**Time as caregiver (months)**	Median (IQR)		13 (0–54)

*IQR = interquartile range.

§ Indicates a primary diagnosis of HIV/AIDS, in the absence of Kaposi sarcoma.

¶ ART = Antiretroviral Therapy.

• HCC = HIV Care Clinic.

# Other serious chronic illnesses include: multi-drug resistant tuberculosis (MDR-TB), end stage liver disease, and progressive neurological illness.

**One caregiver was ineligible for study inclusion because she was younger than 18.

We interviewed 11 of the 12 (92%) caregivers who had accompanied a patient interview participant and were available for study recruitment (Table 5). All caregivers surveyed were family relatives of a patient enrolled in the study and were predominantly female (n = 8, 73%) (Table 5). Most caregivers reported working, typically having jobs as subsistence farmers or market vendors. Caregivers reported having served in this role for a median of 13 months (IQR: 0–54 months).

### Prevalence of Pain and Other Symptoms among Patient Interview Participants

A majority of interviewed patients reported pain (n = 31, 86%), with 23 (64%) experiencing moderate-to-severe pain (African POS score≥3) (Table 6). Of those with moderate-to-severe pain, 13 (57%) had a cancer diagnosis. The mean African POS pain score of the 31 interview participants reporting pain was 3.3 (SE 0.2). Most interviewed patients reported additional physical symptoms, with numbness, cough, difficulty sleeping, and shortness of breath cited most frequently.

**Table 6:** Patient symptom presence and severity over the three days prior to Neno Palliative Care Program enrollment (N = 36).

Symptom	Present*		African POS¶
	n	%§	Mean Score (SE)•
Pain	31	86	3.3 (0.2)
Numbness	13	36	3.5 (0.4)
Cough	12	33	3.5 (0.4)
Difficulty sleeping	12	33	3.5 (0.3)
Shortness of breath	12	33	3.7 (0.4)
Nausea/vomiting	8	22	3.4 (0.5)
Constipation	8	22	2.8 (0.7)

*Present indicates score of >0 on the African Palliative Care Association African Palliative Outcome Scale.

¶ African POS = African Palliative Outcome Scale, which scores symptom severity in 1-point increments from 0 to 5 on a 6-point Likert scale. Harding R, Selman L, Agupio G, Dinat N, Downing J, et al. (2010) Validation of a core outcome measure for palliative care in Africa: the APCA African Palliative Outcome Scale. Health and Quality of Life Outcomes 8∶10. doi:10.1186/1477-7525-8-10.

§ Percentages do not sum to 100% as more than one symptom was reported per patient.

• SE = Standard error.

### Psychosocial Needs and Support Reported by Patient and Caregiver Interview Participants

In response to questions about desired psychosocial services, patients frequently mentioned socioeconomic needs (Table 7). Most patients cited a need for generating income (n = 24, 67%), receiving food (n = 22, 61%), or having their homes repaired (n = 22, 61%). Many patients mentioned poverty and lack of food as important barriers to their well-being:

**Table 7:** Patient-reported psychosocial needs and sources of psychosocial support (N = 36).

*Psychosocial needs*	n	%
Help getting a job/making money	24	67
Food support	22	61
Help with home repairs	22	61
Counseling/emotional support	21	58
Relief from other symptoms*	20	56
Help sending children to school	18	50
Doctor or nurse visit	12	33
Spiritual Advice	11	31
Care for children	9	25
Legal Advice	2	6
Other	7	19
***Sources of Psychosocial Support***		
Family	27	75
Church	18	50
Friends	14	39
APZU§/PIH¶ Program on Social and Economic Rights	12	33
APZU/PIH Village Health Worker Program	12	33
Neighbors	8	22
Community-Based Organization	1	3
Social Welfare Office/District Assembly	1	3
Other	4	11

*“Other symptoms” include any physical symptom other than pain.

§ APZU = Abwenzi Pa Za Umoyo.

¶ PIH = Partners In Health.

*‘I was told to eat a well-balanced diet, which I can't do because of financial constraints.*

*First I was given food rations, but I'm not getting them now and I'm losing weight.’ (Participant 20)*

*‘[My house is] very debilitated. It was built by the church. [I am] happy to be in the hospital with rain coming because I won’t get soaked. I sleep in mud at home.’ (Participant 26)*

*‘What I need most in my life is a job; I have always wanted to have a job. I would like help with that.’ (Participant 0)*

Most (n = 21, 58%) patients interviewed reported a desire for emotional support. Few patients (n = 2, 6%) mentioned HIV-related stigma when discussing their need for emotional support.

*‘There are people who still don’t understand. Neighbors. And I was told I would not be hired for a landscaping job because of my status.’ (Participant 19)*

Similar to patients, most caregivers (n = 7, 64%) stated that they needed emotional support. A high percentage of caregivers reported needs in helping meet the demands of caring for patients, including financial support (n = 10, 91%), food (n = 8, 73%), and physical assistance in providing care (n = 5 45%). A smaller number of caregivers reported the following additional needs: affordable medical care for the patient (n = 2, 18%) and help finding employment or help with transportation (n = 1, 9%).

All patients interviewed reported receiving some form of psychosocial support (Table 7). A majority of patients (n = 27, 75%) described receiving psychosocial support from family members. This most frequently consisted of emotional support, but often also included food, housing, caregiving, or financial support. Caregivers noted that close friends (n = 5, 45%), or a parent or neighbor (n = 2, 18%) were important sources of assistance. Several caregivers (n = 4, 36%) reported that they had received material support in the form of food, sharing of caregiving duties, and financial support. When provided, this support came from immediate or extended family members.

Community churches provided many patients (n = 18, 50%) and caregivers (n = 6, 55%) with psychosocial support consisting of home visits by clergy or congregation members or, occasionally, housekeeping and cleaning services. APZU/PIH was also a frequently cited source of psychosocial support, with 12 (33%) patients reporting longitudinal assistance from a VHW and an equal number (n = 12, 33%) mentioning that they had received POSER services. POSER provided patients with emotional support and socioeconomic assistance in the form of food packages, income generating activities, school fee funding, and housing renovations. Patients reported that village health workers in their communities provided them with frequent counseling during home visits.

### The Role of Caregivers

All caregivers reported that they took on the role because of familial obligation. However, a minority of caregivers interviewed also noted that volunteering, friendship, and being the only caregiver available (n = 1 for each response) were also valued reasons. All caregivers described a large time commitment involved in the caregiver role. Caregivers reported attending to patients from 6 to 24 hours per day (mean = 17 hours). Caregivers assisted with a variety of activities of daily living, including feeding, bathing and dressing. Caregivers also frequently gave medications (n = 7, 64%), offered emotional support (n = 7, 64%), and provided symptomatic care (n = 5, 45%). Transportation to the clinic and care for dependents were provided by most caregivers (n = 6, 55%), and several (n = 3, 27%) provided housing.

Caregivers reported considerable confidence in their abilities to provide care but also considerable worry (Table 8). Caregivers noted the following as their greatest fears: losing a loved one (n = 5, 45%); not having enough money or resources to care for the patient (n = 4, 36%); an undesirable future for the patient’s children (n = 3, 27%); an unwanted future for themselves (n = 2, 18%); and discrimination or not receiving any assistance in caring for the patient (n = 1, 9%).

**Table 8:** Caregiver-reported confidence, worry, and information over the past three days (N = 11).

Dimension	Mean Africa POS* Score (SE)§
Caregiving confidence level	3.9 (0.5)
Level of worry	3.6 (0.5)
Degree to which caregiver felt adequate information was provided by the palliative care provider(s)	3.4 (0.6)

*African POS = African Palliative Outcome Scale, which scores caregiver dimensions in 1-point increments from 0 to 5 on a 6-point Likert scale. Harding R, Selman L, Agupio G, Dinat N, Downing J, et al. (2010) Validation of a core outcome measure for palliative care in Africa: the APCA African Palliative Outcome Scale. Health and Quality of Life Outcomes 8∶10. doi:10.1186/1477-7525-8-10.

§ SE = Standard error.

*‘I [as a caregiver] worry about the illness because I don’t have enough money at home because I can’t work and take care of my brother, and I’m also taking care of two orphaned children.’ (Participant 7)*

*‘We cannot be open about this type of illness, so I am her only guardian…. Our neighbors think that they will get the disease if they come near, since they’re not sure what type of illness she has.’ (Participant 4)*

### Palliative Care Knowledge

With respect to knowledge about their illness, most patients interviewed were able to describe the nature of their illness and the names of, or indications for, any treatments that they were receiving. The most frequent gaps in knowledge existed for hospitalized patients. These patients often had a new or still unconfirmed diagnosis. One patient–who knew that he was HIV-positive but was not aware that he had just been diagnosed with Kaposi sarcoma–illustrated this point as follows:

*‘I have some sores on the chest, edema in the legs, and some shortness of breath,*

*and some bad pains. I haven't been told anything else.’ (Participant 33)*

Despite attending to the many physical and psychosocial needs of patients, few caregivers (n = 2, 18%) had received formal training or counseling in palliative care. Most caregivers (n = 7, 64%) identified training in patient care as a major need.

We interviewed 7 key stakeholders. All key stakeholders reported being familiar with the concept of palliative care, even those working for organizations providing psychosocial support without an explicit palliative care mission. All were aware that national palliative care guidelines existed and that they had been updated in 2011. However, most stakeholders not directly involved with the NPCP had not seen the updated guidelines, and noted that they had not yet been fully disseminated at the district level. Stakeholders noted that additional training was needed for all levels of palliative care providers.

### Preferences for Palliative Care Setting and Models of Service Delivery

Patient and caregiver interview participants were asked to select one or more settings where they would most like to receive palliative care from among five possibilities and to explain the rationale for their selections (Appendix S1). Patient responses most frequently included home-based care (46%), community-based care at a nearby health post or health center (30%), and hospital-based care (24%); only 1 response (2% of the 50 total responses) favored intensive palliative care provided in a designated hospital-based ward (a notion approximating the idea of a hospice). Caregivers reported split preferences for where they would most like patients to receive palliative care, with 46% of responses mentioning home-based care, 46% citing community-based care, and 36% referencing hospital-based care. Most caregivers (n = 9, 82%) reported wanting the support of a village health worker, home-based care volunteer, or other form of home-based care assistance.

*‘If I can get a doctor to visit at home that would be good because I came a very long way here and I have many other duties that I am missing at home.’ (Participant 13)*

*‘Assuming I will be discharged, will I be able to get care at home? I cannot walk, or use my arms and legs, so it was very difficult to get here, and I need care at home.’ (Participant 3)*

While most patients and caregivers expressed a preference for home-based care, hospital care was still important to many, particularly patients who felt that their illness was serious or required higher level care, and patients who expressed a distrust of the level of expertise that would be provided in a health center or in their home. For others, the convenience of receiving care in an easily accessible place was most important. Several patients noted that travel to the hospital could be difficult, time-consuming, and disruptive to work and home responsibilities. One caregiver summarized the conflicts between accessing healthcare and the other areas of his life this way:

*‘It would be good to have health care close to where we live, because then I would be able to take my brother for care and then go out and do my farming and work. Then I would be able to take care of our family.’ (Participant 6)*

All stakeholders who identified as health workers expressed that palliative care should be widely accessible and integrated with inpatient and outpatient clinical services.

*‘Palliative care should be part of the routine services, not a separate project. It is part of essential healthcare delivery.’ (Stakeholder 7)*

Health workers at public facilities noted that the integration of palliative care with other clinical services proved difficult because of limited health worker time and training. Most stakeholders favored a model in which a mobile palliative care team combined home-based care–where symptom assessment and psychosocial support would be provided by home-based care volunteers or village health workers in the community–with a dedicated hospital-based palliative care clinic or inpatient service for very sick or complex patients. They stated that a major success of palliative care in Neno District was the linking of palliative care in the home, the community, and health facilities. All stakeholders noted that trained village health workers and home-based care volunteers had been crucial in making palliative care, and healthcare in general, accessible to vulnerable patients in Neno district.

### Future Palliative Care Priorities

Opinions about future palliative care priorities varied widely among key stakeholders. Policy-makers felt that the integration of palliative care into national policy would assure its eventual implementation in all districts. Health workers prioritized development and scale up of new models of palliative care service delivery, particularly mobile teams. Psychosocial service providers from APZU/PIH and Chiyembekezo CBO cited the need to further integrate clinical and non-clinical palliative care services and reinforce existing referral systems between these services. Most stakeholders noted that improving coordination–both among palliative care-relevant governmental and non-governmental organizations and across tiers of the public health system–should be a priority for future palliative care activities in Neno district. All stakeholders cited a lack of consistent funding for palliative care activities, amidst competing health priorities, as a threat to the sustainability and scale-up of palliative care services.

*‘When donors provide money for certain activities, priorities shift and old plans can be forgotten. We need to have money [for palliative care] that is stable from year to year.’ (Stakeholder 5)*

## Discussion

We present the results of the first palliative care situation analysis, to our knowledge, from rural Malawi and one of the first from a rural setting in Sub-Saharan Africa. Using rapid evaluation methodology, we describe the scope of needs among patients, family caregivers, and key stakeholders, the availability of integrated palliative care services in Neno district, and a model for palliative care service delivery applicable to rural resource-poor settings. We report a high prevalence of pain, other physical symptoms, and psychosocial needs among patients with serious chronic illnesses, indicating a large need for palliative care in one rural district. Early results from the NPCP suggest that comprehensive palliative care can be provided and integrated with disease-modifying treatment even in the most challenging settings by creating a network of hospital, clinic, and home-based services that include psychosocial support incorporating socioeconomic assistance.

In sub-Saharan Africa, and Malawi in particular, there have been few published evaluations of palliative care programs and limited accounts of successful models of palliative care service delivery relevant to rural areas [27]–[29], [50]–[52]. Indeed, the African Palliative Care Research Network has identified describing and evaluating innovative models of palliative care service delivery as a priority area for palliative care research [29]. We describe an innovative district-wide palliative care program employing a tiered network of providers, integrated with HIV/AIDS and Kaposi sarcoma treatment, and providing novel linkages to psychosocial support services, which achieved several favorable outcomes despite the challenges posed by a rural, resource-limited setting. First, a majority of NPCP patients with pain at baseline successfully accessed oral morphine and NPCP’s trained palliative care providers generally prescribed morphine appropriately to those patients with the highest African POS pain scores. Previous accounts of palliative care services in SSA have highlighted the limited accessibility of strong opioids like morphine and the need for health worker training in pain management, particularly in rural areas [8], [25], [27]. Second, 56% of patients received at least one NPCP follow-up evaluation. This compares favorably to a hospital-based program in urban Malawi where only 40% of palliative care patients had a recorded follow-up visit [15]. We hypothesize that the higher retention we observed was due to patient home visits made by the Neno palliative care clinical team and the work of VHWs and home-based care volunteers who actively linked patients to palliative care services and provided psychosocial support. Moreover, an effective national ART program, supported in Neno by PIH community-based interventions, also may have contributed to favorable retention among HIV-infected NPCP patients. Third, we found that a high proportion of HIV-infected NPCP patients were enrolled in HIV care and on ART, suggesting integration of palliative care services with HIV/AIDS treatment in Neno district. Lastly, we observed a small, non-significant decrease in patient pain severity between the initial and follow-up NPCP encounters for patients with complete pain assessment documentation as well as a decrease in the proportion of patients experiencing moderate-to-severe pain at follow-up. However, inferences about the effectiveness of pain management in our program are limited by missing chart information and insufficiently granular prescribing data. We postulate that several patients experienced moderate-to-severe pain at follow-up either because providers were hesitant to start or escalate opioid therapy at the initial NPCP encounter or because of progression of their chronic disease.

Previous studies of palliative care programs in Malawi have reported the young age and high HIV prevalence of adult patients with serious chronic illnesses accessing palliative care services [15], [16]. We present similar findings for patients from rural Neno district, most of whom were young and HIV-infected. These data reflect the demographics of the generalized HIV epidemic in Malawi [19]. Unlike the national epidemic, however, in which women are affected disproportionately, most patients in the NPCP cohort were men–a gender composition similar to that of a hospital-based program in urban Malawi, but differing from female-predominant gender distributions reported by other palliative care centers in SSA [15], [53]. For patients with pain in our cohort, African POS pain scores did not differ significantly by gender at either the initial or fist follow-up encounter. Limited cross-sectional data suggest there may be an association between gender and pain, as well as between gender and overall symptom burden in the African context [27], [53], [54]. Additional observational studies from SSA are needed to better understand the effect of gender on symptom frequency, physical and psychological symptom burden, other palliative care outcomes, and care seeking behavior.

We identified Kaposi sarcoma (KS) as the most prevalent diagnosis among patients interviewed at the time of NPCP enrollment and the most common cancer among the first patients cared for in the NPCP. Kaposi sarcoma was also the most common diagnosis among palliative care patients at one center in Salima district, highlighting the burden that KS and other cancers place on patients and the health system in Malawi [17], [20]. We observed NPCP cancer patients to have a significantly higher baseline African POS pain score than patients without a cancer diagnosis. However, this difference was not observed when comparing patients with and without KS. While differences in needs and perspectives between cancer patients and PLHIV (without cancer) did not emerge as a theme in our qualitative analyses, ours is among the few studies from SSA to quantitatively evaluate adult cancer pain or compare pain by patient diagnosis [25], [27].

Our study also presents one of the largest samples of patients and caregivers from a single SSA country interviewed about palliative care needs, knowledge, and preferences. We report a high prevalence of patient pain, shortness of breath, and cough, reflecting a symptom profile similar to that of AIDS patients from rural Uganda and patients accessing palliative care services at a referral hospital in urban Malawi [15], [26]. Neno patients and caregivers expressed a strong desire for psychosocial support at the time of NPCP enrollment, with unmet socioeconomic needs cited most frequently. In an evaluation of an independent, home-based care program serving peri-urban areas outside Blantyre, Malawi, patients expressed a desire to have basic necessities met, including the need for employment, food, and shelter [16]. Patients in Neno reported similar needs at NPCP enrollment, requesting support for income generation, food, and home repairs.

Numerous Neno patients detailed how the effects of poverty exacerbated the physical and psychological symptoms of their chronic illness. Similar findings have been described elsewhere in SSA [25], [54]–[55]. In South Africa, for example, food insecurity was associated with greater HIV symptom severity, and in a survey of PLHIV from four Southern African countries, fewer disease symptoms were reported among study participants who had a modicum of socioeconomic security [54], [55]. Basic unmet socioeconomic needs also affected caregivers in Neno, contributing to caregiver worry and shaping perceptions about their ability to manage the caregiver role. Taken together, these findings underscore the importance of including socioeconomic interventions as a core component of palliative care.

Despite the well-documented socioeconomic needs reported by palliative care patients and caregivers, however, few programs have been described that provide integrated, psychosocial interventions to mitigate the adverse effects of poverty. One of the few programs to effectively incorporate such psychosocial support, Hospice Africa Uganda, uses a “comfort fund” to pay for patient transportation expenses and to cover the costs of basic patient necessities [56]. We report that 76% of patients in our NPCP cohort were referred for psychosocial services, including 54% linked directly to socioeconomic assistance, including food packages, transportation vouchers, and other forms of material support. Socioeconomic assistance, as well as counseling and emotional support, was provided by the APZU/PIH Program on Social and Economic Rights–a partnership with the Government of Malawi that enables integration of psychosocial and clinical palliative care services.

While the burden of caring for patients with serious, chronic illnesses in resource-limited settings often falls to caregivers [57], limited evidence describes their palliative care needs [58]. We report that caregivers are considerably worried about their loved ones and have fears about the future wellbeing of themselves, the patient, and the patient’s children. We found that caregivers were the primary providers of nursing care and emotional support, helping patients with activities of daily living 17 hours per day, on average. Caregivers expressed a desire for receiving assistance in the home from either a community health worker or home-based care volunteer to enable them to meet competing familial obligations while still providing direct care to the patient. Given the extent of their involvement in palliative care, we identified a need to improve caregiver training and psychosocial support to enable them to continue functioning in the caregiver role.

Due to funding and time constraints, our study was not designed to identify predictors of pain control, assess longitudinal patient outcomes, or explore all themes surrounding palliative care in the African context. Instead, structured interviews provided a comprehensive assessment of patient symptoms and support at the time of NPCP enrollment and the chart review described patient characteristics and changes in pain level over a short follow-up period. The qualitative components of our study may have been limited by information bias arising from the interview translation process or the presence of an accompanying counterpart during patient and caregiver interviews, recall bias as participants generated their responses, or selection bias due to the palliative care referral practices of health workers or the recruitment process for interview participants. In particular, selection bias may have been introduced by enrolling fewer than the targeted number of caregivers. While no caregivers refused study participation, only 25% of patient interview participants had an accompanying caregiver available for study recruitment, potentially limiting the breadth of caregiver perspectives represented in our study. We attempted to limit the systematic bias presented by these issues by employing a dedicated, trained study interpreter, incorporating the African POS into our structured interview instrument, sensitizing health workers throughout Neno district about appropriate indications for NPCP referral, and conducting an interim analysis to ensure that we had achieved saturation of major themes before concluding participant recruitment. Owing to the immediate de-identification of study data, it was not possible to obtain feedback on our findings from patient and caregiver interview participants, which may have limited full understanding of the meanings and perspectives derived from participant responses [46]. For the chart review, missing data due to patient loss to follow-up and incomplete documentation by palliative care providers limit the inferences that may be drawn regarding the effectiveness of pain management, clinical predictors of pain control, and provider analgesia-prescribing practices. While a prospective study design would have obviated many of these limitations, using routinely collected clinical data in our analyses provided a ‘real-world’ operational perspective on palliative care service delivery in rural Malawi.

Since completing data collection for the situation analysis, we have enrolled 105 additional patients in the NPCP (through December 31, 2013). We have applied our findings to recruit and train more village health workers, now numbering 820, to provide additional community-based psychosocial support to patients and their caregivers throughout Neno district, and we have further harmonized their services with those of home-based care volunteers. We have also worked to improve patient pain and symptom management by disseminating the most recent MoH palliative care guidelines, providing longitudinal mentorship to health workers on palliative care practice, including use of the WHO pain relief ladder, and strengthening analgesic supply chain management at all Neno health facilities. In the future, we plan to identify designated personnel to offer intensive caregiver counseling and to further integrate palliative care with non-communicable disease treatment provided at the district’s two hospitals. Notably, all components of the Neno Palliative Care Program continue to be jointly implemented by APZU/PIH in partnership with the government of Malawi, including the Ministry of Health and the Ministry of Local Government and Rural Development–distinguishing the Neno model from other palliative care programs and increasing the potential for replication in other Malawi districts.

In summary, pain, other physical symptoms and psychosocial distress are highly prevalent among patients with cancer, HIV/AIDS, and other serious chronic illnesses in rural Malawi. Patient suffering and caregiver anxiety are exacerbated by poverty and a lack of access to psychosocial support, particularly socioeconomic assistance. However, early results from the NPCP suggest it is possible to provide comprehensive palliative care integrated with disease-modifying treatment in a rural, resource-limited setting through public-private partnership and by establishing a network of community-based village health workers and home-based care volunteers that links households with health centers and hospitals. This network can provide care where most patients live and want services–in their homes–while providing a safety net of facility-based palliative care for patients with severe or refractory symptoms or who lack adequate family support. Further research is needed to assess the effect of comprehensive community-based palliative care on longitudinal patient clinical outcomes, including control of pain and other physical and psychological symptoms, and to assess the cost and scalability of our model. In Neno District, and likely other comparable rural settings in sub-Saharan Africa, palliative care can be improved by integrating palliative care with disease-specific therapies, aggressively treating pain and other symptoms, providing support for caregivers in the home, and pragmatically addressing the issues of poverty that exacerbate symptoms of chronic disease and patient and caregiver distress.

## Supporting Information

Appendix S1 **Structured patient and caregiver interview instrument.**
(PDF)

Appendix S2 **Neno Palliative Care Program initial assessment form.**
(PDF)

Appendix S3 **Malawi National Palliative Care Guidelines.**
(PDF)

Appendix S4 **Introduction to Palliative Care: Health Care Workers’ Service Providers Manual.**
(PDF)

Appendix S5 **Malawi Guidelines for the Clinical Management of HIV in Children and Adults.**
(PDF)
