# Supplementary material for: Assessing and Responding to Palliative Care Needs in Rural Sub-Saharan Africa: Results from a Model Intervention and Situation Analysis in Malawi
Source: PLoS One. 2014 Oct 14;9(10):e110457. doi: 10.1371/journal.pone.0110457 (PMC4197005; doi:10.1371/journal.pone.0110457)
Supplement: Appendix S1 — Structured patient and caregiver interview instrument. (PDF) [file pone.0110457.s001.pdf]

Initials/Interview #: ________

Date Entered: ___/___/___

# Palliative Care Needs Assessment - Patient Survey Form
English Version

Interview Date: __ /__/______ *(DD/MM/YYYY)* Patient Gender (circle): **M F**

Health Facility: ________________________________ Ward/Service: _____________________

Patient Village and District: ____________________________________________________________

Date of Admission or Most Recent Visit: __ /__/______ *(DD/MM/YYYY)*

Diagnosis/Differential: ______________________________ Diagnosis Date: __ /__/______ *(DD/MM/YYYY)*

## Section I: Demographics and Diagnosis

| # | Question | Response |
|---|---|---|
| 1 | What is your age?<br>*Check patient health passport or ask them to estimate if they are unsure.* | ______________ Age in Years<br>*DO NOT proceed if the patient is <15 years of age.* |
| 2 | What is the highest level of education you received?<br>*Clarify primary vs secondary if there is confusion over old vs new classification.* | (please circle one)<br>1) None at all<br>2) Primary<br>3) Secondary<br>4) University or higher |
| 3 | What is your marital status? | (please circle one)<br>1) Single<br>2) Married<br>3) Living as married<br>4) Separated<br>5) Divorced<br>6) Widowed |
| 4 | How many people live in your house including you?<br>*Include the patient in this count.* | __________ Number |
| 5 | How many of these are children? | __________ Number |
| 6 | What do you do currently for work?<br>*Ask patient what they did previously if they are out of work due to illness.* | |
| 7 | What is your religion?<br>*Record particular sect or church of Christianity, not simply 'Christian.'* | |
| 8 | When were you diagnosed with this illness?<br>*Ask the patient to approximate the month and year. Review the record *briefly* if available.* | __________ Month/Year |
| 9 | What have you been told about your illness?<br>*Record all of the patients thoughts.* | |
| 10 | What treatments are you receiving now?<br>*Ask the patient to describe as much as they know, asking if they know what the medicines/treatments are for.* | |

| # | Question | Response |
|---|---|---|
| 11 | What care or treatments did you receive before you came to this health facility?<br>*Ask for the general series of treatments and where they occurred.* | |
| 12 | Who is the person or people that help to take care of you?<br>*Record answers like 'mother' or 'brother.'* | |
| 13 | If you were able to choose, where would you most like to receive medical care?<br>*Carefully explain all options. Ask the patient what makes them choose one option vs the other.* | (Circle all that apply)<br>1) At Home<br>2) At the Hospital<br>3) At a health post or health center in the community<br>4) Hospice (i.e. A hospice is a hospital that provides special care for patients with life-threatening diseases, focusing on taking away pain and other debilitating symptoms).<br>5) Other |

**Section II: African Palliative Outcomes Scale**

*Use the visual symptom scale below to answer the questions in this section. Take time to explain the concept to patients. If it is not easy for them, ask as a yes/no question first, then ask them to assign a number.*

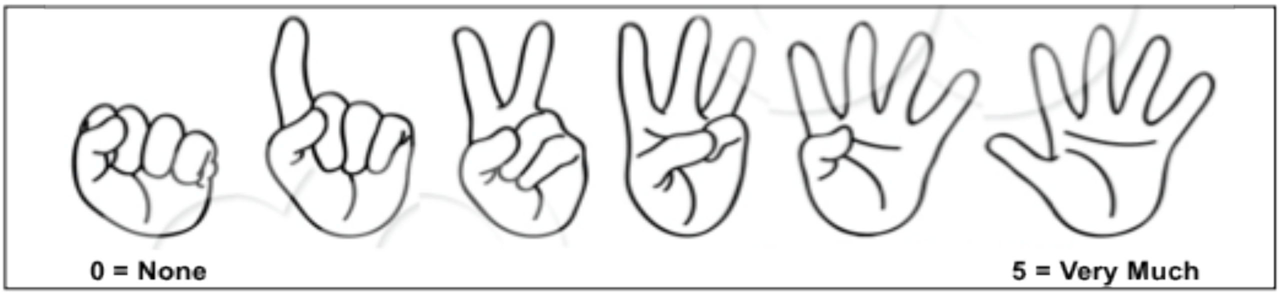


| # | Question | Response |
|---|---|---|
| 14 | Please rate your pain (from 0 = no pain to 5 = worst/ overwhelming pain) during the last 3 days | ________(0-5) |
| 15 | Have any other symptoms (e.g. nausea, coughing or constipation) been affecting how you feel in the last 3 days?<br>*Prompt with these common symptoms. Circle and record numbers:*<br>Nausea or Vomiting<br>Constipation<br>Numbness or tingling in your hands and feet<br>Trouble breathing<br>Coughing<br>Hiccups<br>Trouble sleeping | Specify Other Symptoms Here<br><br>_________________________(0-5)<br>_________________________(0-5)<br>_________________________(0-5)<br>_________________________(0-5)<br>_________________________(0-5)<br>_________________________(0-5)<br>_________________________(0-5)<br>_________________________(0-5) |
| 16 | Have you been feeling worried about your illness in the past 3 days? | ________(0-5) |
| 17 | Over the past 3 days, have you been able to share how you are feeling with your family or friends? | ________(0-5) |
| 18 | Over the past 3 days, have you felt that life was worthwhile? *Alternately, 'that life was good.'* | ________(0-5) |

| # | Question | Response |
|---|---|---|
| 19 | Over the past 3 days, have you felt at peace? | _______(0-5) |
| 20 | Over the past 3 days, have you had enough help and advice for your family to plan for the future? | _______(0-5) |

## Section III: Support and Other Services

| # | Question | Response |
|---|---|---|
| 21 | Do you receive any support (encouragement/emotional, spiritual, monetary, or physical) from any people or organizations? Can you describe what kind of support you get?<br><br>*Ask in detail about each agency and what kind of support is given. In particular, ask if family, friends, and neighbors give encouragement/ emotional support.* | (Circle all that apply)<br>1) Family<br>2) Neighbors<br>3) Friends<br>4) POSER/ Abwenzi Pa Za Umoyo<br>5) Village Health Worker<br>6) Church or Religious Group<br>7) Social Welfare Office/ District Assembly<br>8) Community-based organization in your area<br>9) Others:________________________________________<br>________________________________________ |
| 22 | What services do you need?<br><br>*Allow person to answer spontaneously; if they need prompting use prompts in next column and go through each in detail.* | (Circle all that apply)<br>1) Pain relief<br>2) Symptom relief<br>3) Having a doctor or nurse visit you in the home<br>4) Counseling/emotional support<br>5) Spiritual advice<br>6) Legal advice<br>7) Help getting a job/ making money<br>8) Care for my children<br>9) Help with sending children to school<br>10) Help making repairs to their house<br>11) Other:____________________ |
| 23 | What information do you need? | 1) Caring for yourself and your illness<br>2) Nutrition<br>3) How to make a living/get a job<br>4) Support services found in your community offered by a church, community-based organization or other group.<br>5) Other:________________________________________<br>________________________________________ |
| 24 | Do you have any other comments? | |
| 25 | Do you have any questions? | |

| | | |
|---|---|---|
| Clinical Pass-off to Palliative Care Team? (Circle One): | Yes | Patient Declines |
| Referal to POSER? (Circle One): | Yes | Not Indicated Patient Declines |

Initials/Interview #: ________

Date Entered: ___/___/___

# Rapid Needs Assessment - Guardian Survey Form
English Version

Interview Date: __ /__/______ Caregiver Gender (circle): **M F**

Health Facility: ________________________________ Ward/Service: _____________________

Guardian Village and District:_________________________________________________________

## Section I: Demographics and Diagnosis

| # | Question | Response |
|---|---|---|
| 1 | What is your age?<br>*Ask them to estimate if they are unsure.* | ______________ Age in Years<br>*DO NOT proceed if the guardian is <15 years of age.* |
| 2 | What is the highest level of education you received?<br>*Clarify primary vs secondary if there is confusion over old vs new classification.* | (please circle one)<br>1) None at all<br>2) Primary<br>3) Secondary<br>4) University or higher |
| 3 | What is your marital status? | (please circle one)<br>1) Single<br>2) Married<br>3) Living as married<br>4) Separated<br>5) Divorced<br>6) Widowed |
| 4 | How many people live in your house including you?<br>*Include the guardian in this count.* | __________ Number |
| 5 | How many of these are children? | __________ Number |
| 6 | What do you do currently for work?<br>*Ask guardian what they did previously if they are out of work due to illness.* | |
| 7 | What is your religion?<br>*Record particular sect or church of Christianity, not simply 'Christian.'* | |
| 8 | What is your relationship to the patient you care for? | |

## Section II: African Palliative Outcomes Scale

*Use the visual symptom scale below to answer the questions in this section. Take time to explain the concept to patients. If it is not easy for them, ask as a yes/no question first, then ask them to assign a number.*

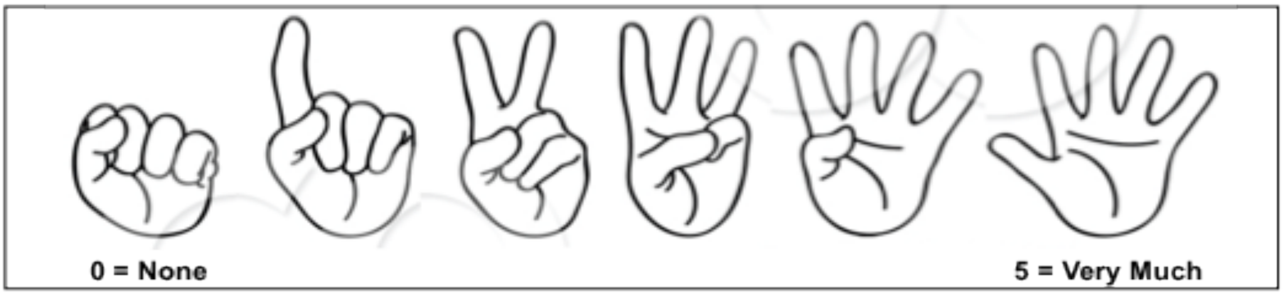

| # | Question | Response |
|---|---|---|
| 9 | Over the past 3 days, how much information have you been given about the patient's illness? | _______(0-5) |
| 10 | Over the past 3 days, how confident have you felt caring for the patient? | _______(0-5) |
| 11 | Have you been feeling worried about the patient over the last 3 days? | _______(0-5) |

**Section III: Caregiving and Support**

| # | Question | Response |
|---|---|---|
| 12 | How is your health? | (Please circle one)<br>1) Very poor<br>2) Poor<br>3) Neither poor nor good<br>4) Good<br>5) Very good |
| 13 | How long have you been providing care to the sick person? | ___ Days<br>___ Months<br>___Years |
| 14 | When did you first learn of their illness? | |
| 15 | How did you come to be providing care to this patient? | (Circle all that apply)<br>1) We are family members<br>2) I volunteered<br>3) S/he is my friend<br>4) I am the only one available<br>5) The patient was abandoned<br>6) Other:_______________________________ |
| 16 | How many hours a day do you provide care to the sick person?<br><br>*Try to quantify the number of waking hours that the person spending caring for the person. If a patient is completely unable to care for themselves, put 24 hours.* | ________Hours |
| 17 | Were you given any training to do what you are doing? | 1) Yes<br>2) No |
| 18 | If yes, by whom? | |
| 19 | Did this training provide you with the skills you need? | 1) Yes<br>2) Somewhat<br>3) No<br>4) Don't know |

| # | Question | Response |
|---|---|---|
| 20 | What major problems do you encounter when you look after this patient? | (Circle all that apply)<br>1) Not enough money<br>2) Discrimination<br>3) It is very hard work<br>4) I do not know how to provide care<br>5) I do not have any help<br>6) I worry about the future<br>7) I do not have enough time to do other important things<br>8) It makes me sad<br>9) I cannot do other work to make money or provide food for my family when I look after the patient.<br>10) Other:_______________________________ |
| 21 | What exactly do you do in caring for this patient? | (Circle all that apply)<br>1) Feed them<br>2) Bathe them<br>3) Give them medicines<br>4) Massage them<br>5) Dress them<br>6) House them<br>7) Transport them to the hospital or clinic<br>8) Provide care for their children and/or their dependents<br>9) Provide emotional support<br>10) Provide care for symptoms<br>11) Other:____________________________ |
| 22 | Do you receive any support from anyone for caring for this person? | 1) Yes<br>2) No |
| 23 | If yes, who has provided support? | 1) Hospital or Health Center Staff<br>2) POSER/ APZU (Abwenzi Pa Za Umoyo)<br>3) Village Health Workers<br>4) Church or Religious Group<br>5) Social Welfare Office/ District Assembly<br>6) Community-based organization in your area<br>7) Extended family<br>8) Friends<br>9) Neighbor<br>10) Other:_________________________ |
| 24 | What kind of support did you receive? | |
| 25 | Has/does anyone provide you with psychological or spiritual support if you need it? | 1)Yes<br>2) No<br>3) I have not needed this support |
| 26 | If yes, who? | (Circle all that apply)<br>1) Spouse<br>2) Mother/father<br>3) Sister/brother<br>4) Other family member(s)<br>5) Close friend<br>6) Health care worker(s)<br>7) Counsellor<br>8) A priest or clergy member<br>9) Member of community-based organization in your area<br>10) Other:<br>11) Nobody |

| # | Question | Response |
| --- | --- | --- |
| 27 | What is your greatest fear right now?<br>*Carefully read all options.* | (Circle all that apply)<br>1) The person I am caring for being in pain<br>2) The person I am caring for dying/ losing a loved one<br>3) Not having enough money to give them a proper funeral<br>4) Discrimination/ being judged, criticized by other<br>5) My future<br>6) The future of their children<br>7) Not ever receiving any help from anyone<br>8) Feeling hopeless<br>9) Not having enough energy to provide care<br>10) Not knowing how best to provide care<br>11) Not having enough money to provide care<br>12) Other:______________________________ |
| 28 | What is your greatest need right now?<br>*Carefully read all options.* | (Circle all that apply)<br>1) Food<br>2) Money<br>3) Affordable medical care<br>4) Knowledge/skills in how to provide care<br>5) Someone to help me with proving care<br>6) Someone to provide me with emotional support<br>7) A job/ way to make money<br>8) Love<br>9) Respect<br>10) Sympathy/understanding<br>11) Other:_______________________________ |
| 29 | What assistance, if given to you, would make it easier for you to look after this patient?<br>*Carefully read all options.* | (Circle all that apply)<br>1) Money<br>2) Food<br>3) Income generating activity<br>4) Caregiving supplies (eg bleach, gloves)<br>5) A caregiving assistant, like a village health worker<br>6) Home-based care services<br>7) Training in patient care<br>8) Other:__________________________ |
| 30 | Given a choice, where would you prefer this patient was cared for?<br><br>*Carefully explain all options. Ask the guardian what makes them choose one option vs the other.* | (Circle all that apply)<br>1) At home<br>2) At the hospital<br>3) At a health post or health center in the community<br>4) At a hospice (i.e. A hospice is a hospital that provides special care for patients with very serious diseases at the end of life, focusing on taking away pain and other debilitating symptoms).<br>5) I do not mind either way<br>6) Other:_________________________ |
| 31 | What information do you need?<br>*Carefully read all options.* | (Circle all that apply)<br>1) Caregiving skills<br>2) Nutrition<br>3) How to make a living/ get a job<br>4) Support services found in your community offered by a church, community-based organization or other group.<br>5) Other:_____________________________ |
| 32 | Do you have any other comments? | |
| 33 | Do you have any questions? | |

## Informed Consent for Patient Interviews - Rapid Situation Analysis of Palliative Care Needs and Services for Patients with Chronic, Serious Illnesses in Neno District, Malawi

Study Title: Rapid Situation Analysis of Palliative Care Needs and Services for Patients with Chronic, Serious Illnesses in Neno District, Malawi

Sponsors: Malawi Ministry of Health, Abwenzi Pa Za Umoyo (APZU)/Partners in Health (PIH), Dana Farber Cancer Institute, and Harvard Medical School

**Co-Principal Investigator:** Felix Chingoli, MBBS
**Institution:** MoH, Government of Malawi

**Contact:** Felix Chingoli, MBBS
**Daytime number:** + 265 (0) 881 765 805
**After hours:** + 265 (0) 881 765 805

**Co-Principal Investigator:** Michael Herce, MD, MPH
Institution: APZU/PIH, Brigham & Women's Hospital and Harvard Medical School

Contact: Michael Herce, MD, MPH
Daytime number: + 265 (0) 888 960 558
After hours: + 265 (0) 888 960 558

**To the Participant**: *This consent form may contain words that you do not understand. Please ask the study staff to explain any words or information that you do not clearly understand. You may take home* ***an unsigned copy of this consent*** *form to think about or discuss with family or friends* ***before making your decision.***

Dear Sir or Madam,

We are working to assess patients with serious illnesses in Neno need and how good our care is. We hope that this information will lead to improvements in the quality of care.

We are inviting you to consider helping us in this research. Your participation in this research is entirely voluntary.

If you agree to participate, our study interviewer will ask you what services you have received and what services that you need as a patient. There are no right or wrong answers; we only want to know what you have been feeling and what you think. The meeting with the study interviewer will take 30 minutes.

If at any time you would like to stop the interview or withdraw from the study, you are free to do so, and this will not affect in any way the care that you receive from your doctors, nurses, healthcare workers at the hospital or clinic or services you receive from Abwenzi Pa Za Umoyo/ Partners in Health in your community.

The information you provide for the research shall be kept confidential. We will do this by not writing your name on the answer sheet. Therefore, not even the researchers who review the answer sheets will know who said what. They will just know how many people gave each answer to each question. In addition, the study interviewer who speaks with you will not discuss your answers with your family, your doctor, neighbors in your community or anyone else outside the research study. However, if you specifically request that we provide all or part of your answers to your doctor, we are willing to do as you request.

There may be no benefit to you of participating in this research. If you request that we give your doctor some or all of the information you provide, it is possible that your doctor or healthcare team may be able to improve the care that you receive. Because your name will not be connected to the information you provide, the risk that someone outside of the study might learn confidential information about you is very small. Your name will NOT be connected with your information, UNLESS you request that we give your information to your doctor. It is possible that during the course of the interview, you may find it emotionally difficult to complete our survey. You should know that you may stop the interview at any time, and this will not affect the care or services you receive in any way.

Please feel free to ask any member of our research team for more information on this research. Should you wish to contact us at any stage regarding this consent form or for further information, you may contact **Dr. Michael Herce** at **0888960558** or **Dr. Felix Chingoli at 0881765805**.

**INFORMED CONSENT**

I hereby confirm that I have been informed by the study interviewer, ______________________, about the nature, conduct, benefits and risks of the study "Rapid Situation Analysis of Palliative Care Needs and Services for Patients with Chronic, Serious Illnesses in Neno District, Malawi"

- I have read or heard and understood the above information
- I am aware that the results of the study, including personal details such as my sex, age, and diagnosis will be made anonymous (separated from my name) and then processed into a study report.
- In view of the requirements of research, I agree that the data can be processed in a computerized system by the research team or on their behalf.
- I may, at any stage, without prejudice, withdraw my consent and participation in the study.
- I have had sufficient opportunity to ask questions and, of my own free will, declare myself prepared to participate in the study.

PARTICIPANT:

Printed Name Signature / Mark or Thumbprint Date and Time

-------------------------------------------------------------------------------------------------------------------

I, _________________________ (study interviewer), hereby confirm that the above participant has been fully informed about the nature, conduct and risks of the above study.

Study Staff:

Printed Name Signature Date and Time

## **Informed Consent Document for Caregiver Interviews - Rapid Situation Analysis of Palliative Care Needs and Services for Patients with Chronic, Serious Illnesses in Neno District, Malawi**

Study Title: Rapid Situation Analysis of Palliative Care Needs and Services for Patients with Chronic, Serious Illnesses in Neno District, Malawi

Sponsors: Malawi Ministry of Health, Abwenzi Pa Za Umoyo (APZU)/Partners in Health (PIH), Dana Farber Cancer Institute, and Harvard Medical School

**Co-Principal Investigator:** Felix Chingoli, MBBS
**Institution:** MoH, Government of Malawi

**Contact:** Felix Chingoli, MBBS
**Daytime number:** + 265 (0) 881 765 805
**After hours:** + 265 (0) 881 765 805

**Co-Principal Investigator:** Michael Herce, MD, MPH
Institution: APZU/PIH, Brigham & Women's Hospital and Harvard Medical School

Contact: Michael Herce, MD, MPH
Daytime number: + 265 (0) 888 960 558
After hours: + 265 (0) 888 960 558

**To the Participant**: *This consent form may contain words that you do not understand. Please ask the study staff to explain any words or information that you do not clearly understand. You may take home* ***an unsigned copy of this consent*** *form to think about or discuss with family or friends* ***before making your decision.***

Dear Sir or Madam,

We are working to assess what caregivers for patients with serious illnesses in Neno need and how good our care is. We hope that this information will lead to improvements in the quality of care.

We are inviting you to consider helping us in this research. Your participation in this research is entirely voluntary.

If you agree to participate, our study interviewer will ask you what services you have received and what services that you need to help you as a caregiver. There are no right or wrong answers; we only want to know what you have been feeling and what you think. The meeting with the study interviewer will take 30 minutes.

If at any time you would like to stop the interview or withdraw from the study, you are free to do so, and this will not affect in any way the care that the patient that you care for or the care that you yourself receive from your doctors, nurses, healthcare workers at the hospital or clinic or services you receive from Abwenzi Pa Za Umoyo/ Partners in Health in your community.

The information you provide for the research shall be kept confidential. We will do this by not writing your name on the answer sheet. Therefore, not even the researchers who review the answer sheets will know who said what. They will just know how many people gave each answer to each question. In addition, the study interviewer who speaks with you will not discuss your answers with your family, your doctor, neighbors in your community or anyone else outside the research study. However, if you specifically request that we provide all or part of your answers to the doctor of the patient that you care for, we are willing to do as you request.

There may be no benefit to you of participating in this research. If you request that we give to the doctor of the patient that you care for some or all of the information you provide, it is possible that

your doctor or healthcare team may be able to improve the care that the patient you care for receives. Because your name will not be connected to the information you provide, the risk that someone outside of the study might learn confidential information about you or the patient that you care for is very small. Your name and/or the name of the patient that you care for, will NOT be connected with your information, UNLESS you request that we give your information to the doctor. It is possible that during the course of the interview, you may find it emotionally difficult to complete our survey. You should know that you may stop the interview at any time, and this will not affect the care or services you or the patient that you care for receive in any way.

Please feel free to ask any member of our research team for more information on this research. Should you wish to contact us at any stage regarding this consent form or for further information, you may contact **Dr. Michael Herce** at **0888960558** or **Dr. Felix Chingoli at 0881765805**.

**INFORMED CONSENT**

I hereby confirm that I have been informed by the study interviewer, ______________________, about the nature, conduct, benefits and risks of the study “Rapid Situation Analysis of Palliative Care Needs and Services for Patients with Chronic, Serious Illnesses in Neno District, Malawi”

- I have read or heard and understood the above information
- I am aware that the results of the study, including personal details such as my sex, age, and diagnosis will be made anonymous (separated from my name) and then processed into a study report.
- In view of the requirements of research, I agree that the data collected during this study can be processed in a computerized system by the research team or on their behalf.
- I may, at any stage, without prejudice, withdraw my consent and participation in the study.
- I have had sufficient opportunity to ask questions and, of my own free will, declare myself prepared to participate in the study.

PARTICIPANT:

Printed Name　　Signature / Mark or Thumbprint　　Date and Time

-------------------------------------------------------------------------------------------------------------------

I, _________________________ (study interviewer), hereby confirm that the above participant has been fully informed about the nature, conduct and risks of the above study.

Study Interviewer:

Printed Name　　Signature　　Date and Time

**Kafukufuku wa zofuna – Fomu ya oyang’anira odwala (Rapid Needs Assessment - Caregiver Survey Form)**

Ya Chichewa (Chichewa Version)

Tsiku la kafukufuku: __ /__/______　　Oyang’anira odwala ndi (Zungulizani): Amuna / Akazi

Dzina la Chipatala: ________________________________　Ku Wodi ya: ____________________

Mudzi ndi Boma lochekera oyang'anira odwalayo:
______________________________________________________________

**Gawo loyamba: Mbiri**

| # | Funso | Yankho |
|---|---|---|
| 1 | Muli ndi zaka zingati? | _______________ (Zaka) |
| 2 | Maphunziro anu munapita nawo mpaka pati? | (Zungulizani pamodzi)<br>1) Sindinapiteko ku sukulu<br>2) Ndinalekeza ku Pulayimale<br>3) Ndinalekeza ku Secondale<br>4) Ndinapitako ku sukulu ya ukachenjede |
| 3 | Muli pa banja? | (Zungulizani pamodzi)<br>1) Osakwatira/ Osakwatiwa<br>2) Ndili pa banja<br>3) Banja linatha<br>4) Anamwalira |
| 4 | Kuphatikiza inuyo, pamodzi mumakhala anthu angati mmunyumba mwanu? | ___________ Nambala |
| 5 | Ana ndi angati pa nambala mwatchulayi? | ___________ Nambala |
| 6 | Mumagwira ntchito yanji kuti mupeze ndalama pamoyo wanu? | |
| 7 | Ndi inu a chipembedzo chanji? | |
| 8 | Chibale chanu ndi odwalayu ndi chotani? | |

**Gawo laChiwiri: Zotsatira za kuyang'anira odwala monga mwa ku Afrika**

*Gwiritsani ntchito zojamburazi pofuna kuyankha mafunso ali musiwa, agwiritse tchito zala pofuna kuyankha.*

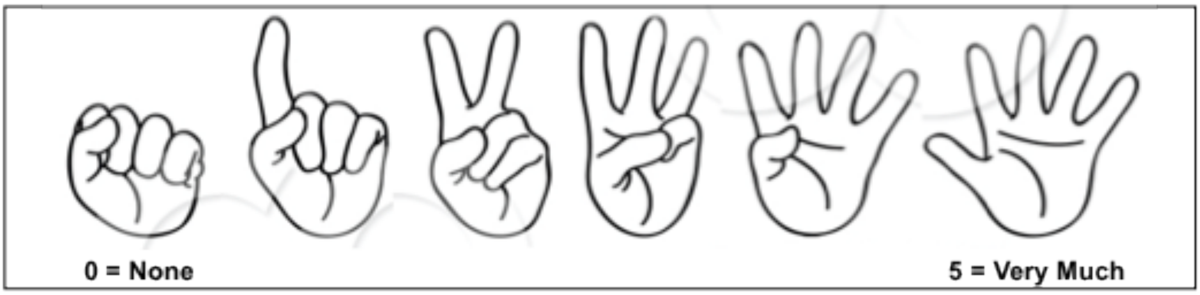


| # | Funso | Yankho |
|---|---|---|
| 9 | Pamasiku atatu adutsawa, mwapatsidwako maphunziro aliwonse? | ________(0-5) |
| 10 | Pamasiku atatu adutsawa, mwakhala muli omasuka kuyang'anira odwala wanu? | ________(0-5) |

| # | Funso | Yankho |
|---|---|---|
| 11 | Munakhalako odandaula ndi odwala wanuyu pa masiku atatu apitawa? | _______(0-5) |

**Gawo lachitatu: Zachisamaliro**

| # | Funso | Yankho |
|---|---|---|
| 12 | Thanzi lanu ndi lotani panopa?<br><br>*Awerengerni mayankhowa* | (Zungulizani pamodzi)<br>1) Thanzi silili bwino kwambiri<br>2) Thanzi silili bwino<br>3) Ndili pakati kati<br>4) Thanzi lili bwino<br>5) Thanzi lili bwino kwambiri |
| 13 | Mwakhala mukuyang'anira odwalayu kwanthawi yochuluka bwanji? | Masiku___<br>Miyezi___<br>Zaka___ |
| 14 | Munadziwa liti zamatenda awa? | |
| 15 | Chinakuoangitsani ndi chain kuti muyambe kumuthandiza odwalayu? | (Zungulizani zoyenera)<br><br>1) Ndi m'bale wanga<br>2) Ndinangozipereka<br>3) Ndi nzanga<br>4) Amene angamuyang;anire ndi ine ndekha<br>5) Odwala anatayidwa opanda omuyang'anira<br>6) Zina:_______________________________ |
| 16 | Ndi ma ola angati patsiku amene mumazamuthandiza odwalayu? | Maola________ |
| 17 | Munapatsidwako maphunziro apadela pa za momwe mungamusamalire odwalauy? | 1) Eya<br>2) Ayi |
| 18 | Ngati EYA, anakuphunzitsani ndi ndani? | |
| 19 | DMaphunziro amenewa anakupindulirani? | 1) Eya<br>2) Pang'ono<br>3) Ayi<br>4) Sindikudziwa |
| 20 | Mumakumana ndi mavuto otani poyang'anira odwala wanu? | Zungulizani zoyenera<br>1) Ndalama sizimakwana<br>2) Kusalidwa<br>3) Ndi ntchito yaikulu kwambiri<br>4) Sindimadziwa momwe ndingamusamalilire odwalayu<br>5) PAlibe amandithandiza<br>6) Ndimadandaula ndi za mtsogolo<br>7) Sindimakhala ndi thawi yopanga zinthu zina zanga<br>8) Ndimakhumudwa nazo<br>9) Ndimakanika mugwira ntchito zanga kuti ndipeze ndalama zothandizira banja langa.<br>10) Zina:_________________________________ |

| # | Funso | Yankho |
|---|---|---|
| 21 | Kwenikweni zimene mupachita pofuna kumuyang'anira patient wanuyu nda chani? | (Zungulizani zones zofunika)<br>1) Kumudyetsa<br>2) Kumusambitsa<br>3) Kuwamwetsa mankhwala<br>4) Kuwasisita pamene kakuwawa<br>5) Kuwaveka<br>6) Kuwapezera pokhala<br>7) Kuwaperekeza ku chipatala<br>8) Kuthandiza ana awo komanso onse amene amadalira odwalayu<br>9) Kuwathandiza pa maganizo<br>10) Kuwathandiza pa zizindikiro zina za matenda awo<br>11) Zina:_______________________________ |
| 22 | Mumalandira thandizi kuchokera kwa aliyenso lomuthandizira odwalayu? | 1) Eya<br>2) Ayi |
| 23 | Ngati EYA, amakuthandizani ndi ndani? | 1) Kuchipatala ndi ogwira tchito za umoyo<br>2) POSER/ APZU (Abwenzi Pa Za Umoyo)<br>3) Alangizi azaumoyo akumudzi<br>4) Amipingo<br>5) Social Welfare Office/ District Assembly<br>6) A CBO akudera kwanu<br>7) Achibale ena ndi ena<br>8) Anzanga<br>9) Oyandikana nawo nyumba<br>10) Ena:___________________________ |
| 24 | Ndithandizo lanji limene mumalandira? | |
| 25 | Mukafuna thandizo la moyo wa uzimu ndi la mmaganizo mumalipeza mosavuta? | 1)Eya<br>2) Ayi |
| 26 | Ngati EYA, ndi ndani amakuthandizani? | (Zungulizani zoyenera)<br>1) Mkazi/Mamuna wanga<br>2) Mayi/Bambo anga<br>3) Mchemwali/Mchimwene wanga<br>4) Achibale ena<br>5) Anzanga apamtima<br>6) Alangizi azaumoyo<br>7) Alangizi ena<br>8) Akulu ampingo ndi ena aku mpingo<br>9) Membala wa ku CBO<br>10) Ena:<br>11) Palibe |

| # | Funso | Yankho |
|---|---|---|
| 27 | Pakali pano mantha anu ndi otani? | (Zungulizani zoyenera)<br>1) Odwala amene ndikumuyang'anira akumva kuwawa kwambiri<br>2) Kuti amene odwalayu atha kumwalira kapena kutaya okondedwa<br>3) Ndilibe ndalama zozathandizira maliro<br>4) Kusalidwa kwambiri ndi anthu<br>5) Tsogolo langa<br>6) Tsogolo la ana a odwalayu<br>7) Sindikulandira thandizo kuchokera kwa aliyense<br>8) Ndilibe chikhulupiriro<br>9) Ndilibe mphamvu zokwanira kupereka thandizo<br>10) Sindimadziwa kuti thandizo labwino ndingapereke bwanji<br>11) Ndalama zothandizira odwalayu ndilibe<br>12) Zina:_______________________________ |
| 28 | Pakali pano chomwe mukufuna kwambiri ndi chani? | (Zungulizani zoyenera)<br>1) Chakudya<br>2) Ndalama<br>3) Thandizo la mankhwala ndi thanzi<br>4) Maphunziro oyenera pa kuyang'anira odwala<br>5) Munthu oti azindithandiza kuyang'anira odwayu<br>6) Munthu oti azindithandiza maganizo<br>7) Ntchito kapena njira yopezera ndalama<br>8) Chikondi<br>9) Kupatsidwa ulemu<br>10) Anthu azindimvetsa komanso azindimvera chisoni<br>11) Zina:________________________________ |
| 29 | Patakhala thandizo, ndi chain chimene munga konde mutapeza? | (Zungulizani zoyenera)<br>1) Ndalama<br>2) Chakudya<br>3) Ntchito yopezetsa ndalama<br>4) Katundu ofunika posamalira odwala<br>5) Munthu oti azindithandiza kusamala odwala, mlangizi wa za Umoyo waku mudzi<br>6) Thandizo la odwala<br>7) Maphunziro a kasamalidwe ka odwala<br>8) Zina:__________________________ |
| 30 | Mutapatsidwa mwayi, ndikuti kumene mungafune odwala wanu kuti akadwalire? | (Zinguzani pamodzi)<br>1) Kunyumba<br>2) Kuchipatala<br>3) Kuchipatala chaku mudzi<br>4) Kumalo amene amayang'anira odwala kwambiri pofuna kuwathandiza maganizidwe abwino pamene akudwala<br>5) Kulikonse<br>6) Zina:_________________________ |
| 31 | Mukufuna kudziwa mauthenga ati? | (Zungulizani zoyenera)<br>1) Ukadaulo wakuyang'anira odwala<br>2) Zakadyedwe<br>3) Zamomwe ndingamapezere ndalama<br>4) Zachithandizo chomwe amipingo amapereka komanso a CBO.<br>5) ZIna:_____________________________ |

| # | Funso | Yankho |
|---|---|---|
| 32 | Muli ndi zonena zili zonse? | |
| 33 | Muli ndi funso lili lonse? | |

**Kafukufuku wa Zofuna – fomu ya Odwala (Rapid Needs Assessment - Patient Survey Form)**
Ya Chichewa (Chichewa Version)

Tsiku la kafukufuku: __ /__/______ Odwala ndi : Wamamuna/ Wamkazi

Cipatala: ________________________________ Wodi uake: _____________________

Mudzi ndi Boma kochokera Odwala:
____________________________________________________________

Tsiku logonekedwa/Lomaliza kupita kuchipatala: __ /__/______

Anakupezani ndi Matenda anji: ______________________________ Munadziwa liti/kuyezedwa liti: __ /__/ ______

**Gawo loyamba: Mbiri**

| # | Funso | Yankho |
|---|---|---|
| 1 | Muli ndi zaka zingati? | Zaka______________ |
| 2 | Maphunziro anu munapita nawo mpaka pati? | (Zungulizani pamodzi)<br>1) Sindinapiteko ku sukulu<br>2) Ndinalekeza ku Pulayimale<br>3) Ndinalekeza ku Secondale<br>Ndinapitako ku sukulu ya ukachenjede |
| 3 | Muli pa banja? | (Zungulizani pamodzi)<br>1) Osakwatira/ Osakwatiwa<br>2) Ndili pa banja<br>3) Banja linatha<br>4) Anamwalira |
| 4 | Kuphatikiza inuyo, pamodzi mumakhala anthu angati mmunyumba mwanu? | __________ Nambala |
| 5 | How many of these are children? Panambala mwatchulayo ana alipo angati? | __________ Nambala |
| 6 | Mumagwira ntchito yanji kuti mupeze ndalama pamoyo wanu? | |
| 7 | Ndi inu a chipembedzo chanji? | |
| 8 | Chibale chanu ndi odwalayu ndi chotani? | |
| 9 | Munauzidwako chani za matenda anu? | |

| # | Funso | Yankho |
|---|---|---|
| 10 | Mukulandira mankhwala anji pakali pano? | |
| 11 | Musanabwere ku chipatala kuno munkalandira mankhwala otani? | |
| 12 | Ndi ndani amene amakuyang'anirani? | |
| 13 | Mungafune thandizo mutamalandirira kuti? | 1) Kunyumba<br>2) Kuchipatala<br>3) Kudera kwathu<br>4) Kumalo amene amayang'anira odwala kwambiri pofuna kuwathandiza ganizo lamaganizidwe<br>5) Zina |

**Gawo lachiwiri: Zotsatira za kuyang'anira odwala monga mwa ku Afrika**

*Gwiritsani ntchito zojamburazi pofuna kuyankha mafunso ali musiwa, agwiritse tchito zala pofuna kuyankha.*

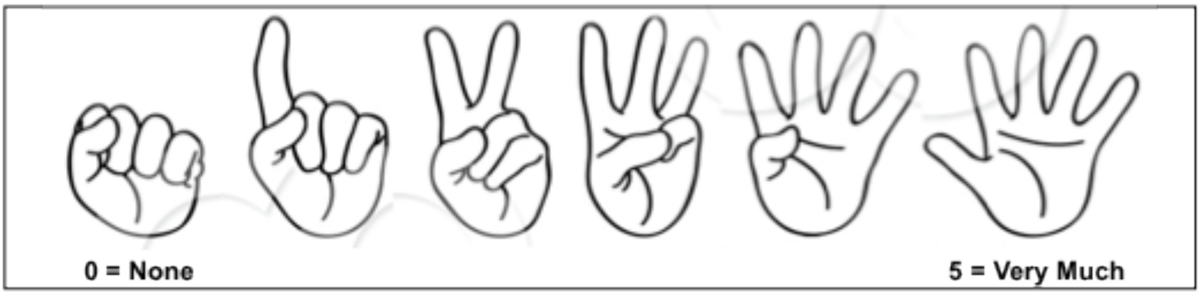


| # | Question | Response |
|---|---|---|
| 14 | Matenda anu mukumva kupweteka bwanji pa masiku atatu apitawa | ________(0-5) |
| 15 | Pa Masiku atatu apitawa mwaonako zizindikiro izi?(nselu, chifuwa ndi kutsegula mmimba)<br>Nseru kapena kusanza<br>Kutsegula mmimba<br>Dzanzi ndi kuyabwa mmanja<br>Kukanika kupuma<br>Kukhosomola<br>Ntchilikiro/Chidikhodikho<br>Kukanika kugona | ________________________(0-5)<br>________________________(0-5)<br>________________________(0-5)<br>________________________(0-5)<br>________________________(0-5)<br>________________________(0-5)<br>________________________(0-5)<br>________________________(0-5) |
| 16 | Pa masiku atatu apitawa mukhalako okhumudwa ndi matenda anu? | ________(0-5) |
| 17 | Munamufotokozerako aliyense za ululu omwe mukumva? | ________(0-5) |
| 18 | Pamasiku atatu apitawo mwaonako kuti moyo siwabwino kukhala? | ________(0-5) |

| # | Question | Response |
|---|---|---|
| 19 | Pamasiku atatu apitawa mwakhalako mumtendere? | _______(0-5) |
| 20 | Pamasiku atatu apitawa mwalandilako thandizo lamomwe munga konzekere za tsogolo lanu? | _______(0-5) |

**Gawo Lachitatu: Zachithandizo**

| # | Funso | Yankho |
|---|---|---|
| 21 | Kodi mumalandira thandizo lililonse kuchokera kulikonse, ndipo ngati zili choncho kodi ndi chani chimene mumalandira? | (Zunguluzani zimene)<br>1) Apabanja<br>2) Oyandikana nawo nyumba<br>3) Abwenzi<br>4) POSER/ Abwenzi Pa Za Umoyo<br>5) Mlangizi wa za Umoyo wa ku mudzi<br>6) Amipingo<br>7) Social Welfare Office/ District Assembly<br>8) a CBO akudela kuno<br>9) Zina:________________________________________ |
| 22 | Ndithandizo liti limene mumafuna mutalandira?<br><br>(Allow person to answer spontaneously;<br>if they need prompting use prompts in next column) | (Zungulizani zoyenera)<br>1) Kuti asiye kumva kuwawa<br>2) Kuchepetsa zovuta zina za matenda<br>3) Kuyenderedwa ndi Dokotala kunyumbako<br>4) Uphungu kapena thandizo la mumtima<br>5) Uphungu wa za umoyo<br>6) Uphungu wa za malanulo adziko<br>7) Kuthandizidwa kupeza njira zopezera ndalama<br>8) Chithandizo cha ana anga<br>9) Kuthandiza kuti ana azi[ita ku sukulu<br>10) Thandizo lito nyumba yanga ikonzedwe<br>11) ZIna:_____________________ |
| 23 | Kodi ndi mauthenga ati amene mumafuna mutamadziwa? | (Zunguzani zoyenera)<br>1) Momwe mungazisamalire komanso kusamala matenda anu<br>2) Zakadyedwe<br>3) Momwe munga pezere ntchito kapena kupeza ndalama<br>4) Thandizo lochokera kumpingo kapena ku CBO yaku dera kwanu.<br>5) Zina:________________________________________ |
| 24 | Muli ndi chonena chili chonse? | |
| 25 | Muli ndi mafunso ali wonse? | |

Initials/Interview #: ________

Date: ___/___/___

# Informed Consent Document for Caregiver Interviews - Chichewa Version

## NDONDOMEKO YA OYANG'ANIRA ODWALA KUVOMEREZA KUTENGA NAWO MBALI MU KAFUKUFUKU WA Zofuna zomwe anthu odwala matenda akuluakulu ndi owayang'anira amakumana nazo ku Neno, Malawi

Mutu wa Kafukufuku: Zofuna zomwe anthu odwala matenda akuluakulu ndi owayang'anira amakumana nazo ku Neno, Malawi

Othandiza Kafukufuku: Malawi Ministry of Health, Abwenzi Pa Za Umoyo (APZU)/Partners I Health (PIH), Dana Farber Cancer Institute ndi Harvard Medical School

Oyang'anira: Michael Herce, MD, MPH

Bungwe: APZU/PIH, Brigham & Women's Hospital and Harvard Medical School

Michael Herce, MD, MPH

Numbala ya foni: +265(0) 88 960 558

Oyang'anira: Felix Chingoli, MBBS

Bungwe: Malawi Ministry of Health

Felix Chingoli, MBBS

Numbala ya foni: + 265 (0) 881 765 805

**Kwa otenga nawo mbali:** Ngati mwapeza mawu amene mukuona kuti simukumvetsa mundondomekoyi chonde auzeni amene akukuyang'anirani kuti akuthandizeni.Mukhoza kutenga fomu iyi **musanailembe** kuti mukaganize bwino kapena kukambirana ndi apabanja panu **musanapange chiganizo.**

Okondedwa Bwana kapena a Dona,

Tili mkafukufuku ofuna kudziwa zimene oyang'anira odwala amafuna komanso kuti chithandizo chawo ndi chabwino bwanji. TIkukhuluoirira zimenezi zithandiza kwambiri pa za momwe timathandizira odwala athu.

Kukukupemphani kuti mutengepo mbali pa mchezo uyu umene uli wongothandiza ndipo ndi wa ulele palibe kulipidwa kwa mtundu uliwonse.

Ngati mwalora kutengapo mbali, mtolankhani wathu amene muzicheza naye akupemphani kuti mumufotokozere thandizo lomwe mwalandira komanso momwe mungafune kumathandizidwira. Palibe yankho lenileni ya kuchezaku tikungofuna kudziwa momwe mumaganizira pa nkhanizi, mchezowu utenga pafupifupi phindi 30.

Mutati mwafuna kudula mchezowu, mungathe kutero ndithu ndipo izi sizingapangitse kuti chithandizo chimene mumalandira ku chipatala chitsike.

Zomwe tikambirane pano zikhale ndi chinsinsikochuluka ndichifukwa dzina lanu sililembedwapo apa ndicifukwa chake ngakhale okaunika kafukufukuyu sadziwa kuti wayankha izi ndi ndani. Komanso mtolankhani wathu amene muzacheze naye sazafotokozera a dokotala anu, a kubanja anu ngakhale anzanu pa zimene tikambirane apa koma ngati mungafune nokha kuti tifotokozere aDokotala anu zimene tikambirane, ife tili okonzeka kutero.

Palibe chimene inu mulandire pamchezo umenewu koma ngati mungafune kuti tiwafotokozere aDokotala za odwala amene mumamuyang'anira izi ndi zotheka pofuna kukweza thandizo limene mumalandira kuchipatala. Mwina pakucheza kwathu mufuna patha kufika poti inu mufuna kusiya kuyankha mafunso, inu mukhoza kutero sipazakhala vuto lililonse kapena kuchepetsa thandizo lomwe limaperekedwa kwa odwala wanu.

Mukhale omasuka kufunsa mafunso aliwonse pa kafukufuku uyu kapena ngati muzafune kudziwa kalikonse ka kafukufuku uyu mukhoza kuiombira **Dr. Michael Herce pa 0 888 960 558.**

**KUTSIMIKIZA KUTI NDAMVETSETSE ZA KAFUKUFUKUYU**

Ndikuvomera kuti ndamvetsetsa zomwe ndauzidwa ndi____________________, za momwe kafukufukuyu alili, zofooka zake komanso zopindila zake.

- Ndalandira, kumva komanso kuwerenga zomwe zalembedwa pamwambazi komanso kulandira ndondomeko ya momwe akhalire afukufukuyu (Participation leaflet)
- Ndamvetsetsanso kuti zotsatira za kafukufukuyu sizisindikizidwa ndi dzina langa
- Ndikugwirizananso ndi zakuti zimene zitoleledwe mukafukufuku uyu zikhoza kugwiritsidwa ntchito ndi anthu ena amugulu mwathu
- Ndafunsa mafunso okwanira oa za kafukufuku ameneyu choncho ndili okonzeka kutenga nawo mbali mu kafukufuku ameneyu.

OTENGA MBALI

Dzina Posayina/Kudinda chala Tsiku ndi Nthawi

--------------------------------------------------------------------------------------------------------------------

Ine, _________________________ (Ntolankhani wa kafukufuku), ndikutsimikiza kuti munthu walembedwa muntundayu wavomereza zonse zoyenera asanayambe kutenga nawo mbali mu kafukufuku ameneyu.

Ntolankhani:

Dzina Posayina/Kudinda chala Tsiku ndi Nthawi

Initials/Interview #: ________

Date: ___/___/___

# Informed Consent Document for Patient Interviews - Chichewa Version

## NDONDOMEKO YA ODWALA KUVOMEREZA KUTENGA NAWO MBALI MU KAFUKUFUKU WA Zofuna zomwe anthu odwala matenda akuluakulu ndi owayang'anira amakumana nazo ku Neno, Malawi

Mutu wa Kafukufuku: Zofuna zomwe anthu odwala matenda akuluakulu ndi owayang'anira amakumana nazo ku Neno, Malawi

Othandiza Kafukufuku: Malawi Ministry of Health, Abwenzi Pa Za Umoyo (APZU)/Partners in Health (PIH), Dana Farber Cancer Institute ndi Harvard Medical School

Oyang'anira: Michael Herce, MD, MPH

Bungwe: APZU/PIH, Brigham & Women's Hospital and Harvard Medical School

Michael Herce, MD, MPH

Numbala ya foni: +265(0) 88 960 558

Oyang'anira: Felix Chingoli, MBBS

Bungwe: Malawi Ministry of Health

Felix Chingoli, MBBS

Numbala ya foni: + 265 (0) 881 765 805.

Okondedwa Bwana kapena a Dona,

**Kwa otenga nawo mbali:** Ngati mwapeza mawu amene mukuona kuti simukumvetsa mundondomekoyi chonde auzeni amene akukuyang'anirani kuti akuthandizeni.Mukhoza kutenga fomu iyi **musanailembe** kuti mukaganize bwino kapena kukambirana ndi apabanja panu **musanapange chiganizo.**

Tili mkafukufuku ofuna kudziwa zimene oyang'anira odwala amafuna komanso kuti chithandizo chawo ndi chabwino bwanji. TIkukhuluoirira zimenezi zithandiza kwambiri pa za momwe timathandizira odwala athu.

Kukukupemphani kuti mutengepo mbali pa mchezo uyu umene uli wongothandiza ndipo ndi wa ulele palibe kulipidwa kwa mtundu uliwonse.

Ngati mwalora kutengapo mbali pa kafukufuku uyu, dziwani kuti tikhala tikukambira za ululu omwe mukumva pa matenda anu ndi mavuto ena mbiri amene mukukumana nawo ngati kuvutika kupuma, kutsegula mmimba kapena chifuwa. Ngati izi zakhala zikukuchitikirani, mufunsidwa kuti kodi mavuto ake amakhala otani. Palibe mayankho eni eni a kafukufukuyu, koma tikungofuna kudziwa za momwe mumaganizira ndi kumvera pa nkhani ya matenda anu. Mchezowu okhoza kutenga mphindi pafupifupi 30.

Chonde ngati mukuona kuti muli ofooka kapena kudwalika kuti simungathe kuyankha mafunso mutiuze. Ngati muone kuti simungapitirize kucheza nafe mungathe kunena momwemo ndipo izi sikuti zisokoneza chibale chanu ndi madokotala kapena azaumoyo ena kapenanso thandizo limene mumalandira ku Abwenzi Pa Za Umoyo pa za thanzi lanu komanso kudera kwanu.

Zomwe tikambirane pano zikhale ndi chinsinsikochuluka ndichifukwa dzina lanu sililembedwapo apa ndicifukwa chake ngakhale okaunika kafukufukuyu sadziwa kuti wayankha izi ndi ndani. Komanso mtolankhani wathu amene muzacheze naye sazafotokozera a dokotala anu, a kubanja anu ngakhale anzanu pa zimene tikambirane apa koma ngati mungafune nokha kuti tifotokozere aDokotala anu zimene tikambirane, ife tili okonzeka kutero.

Palibe chimene inu mulandire pamchezo umenewu koma ngati mungafune kuti tiwafotokozere aDokotala za odwala amene mumamuyang'anira izi ndi zotheka pofuna kukweza thandizo limene mumalandira kuchipatala. Mwina pakucheza kwathu mufuna patha kufika poti inu mufuna kusiya kuyankha mafunso, inu mukhoza kutero sipazakhala vuto lililonse kapena kuchepetsa thandizo lomwe limaperekedwa kwa odwala wanu.

Mukhale omasuka kufunsa mafunso aliwonse pa kafukufuku uyu kapena ngati muzafune kudziwa kalikonse ka kafukufuku uyu mukhoza kuiombira **Dr. Michael Herce pa 0 888 960 558.**

**KUTSIMIKIZA KUTI NDAMVETSETSE ZA KAFUKUFUKUYU**

Ndikuvomera kuti ndamvetsetsa zomwe ndauzidwa ndi____________________, za momwe kafukufukuyu alili, zofooka zake komanso zopindila zake.

- Ndalandira, kumva komanso kuwerenga zomwe zalembedwa pamwambazi komanso kulandira ndondomeko ya momwe akhalire afukufukuyu (Participation leaflet)
- Ndamvetsetsanso kuti zotsatira za kafukufukuyu sizisindikizidwa ndi dzina langa
- Ndikugwirizananso ndi zakuti zimene zitoleledwe mukafukufuku uyu zikhoza kugwiritsidwa ntchito ndi anthu ena amugulu mwathu
- Ndafunsa mafunso okwanira oa za kafukufuku ameneyu choncho ndili okonzeka kutenga nawo mbali mu kafukufuku ameneyu.

OTENGA MBALI

Dzina Posayina/Kudinda chala Tsiku ndi Nthawi

---------------------------------------------------------------------------------------------------------------------

Ine, _________________________ (Ntolankhani wa kafukufuku), ndikutsimikiza kuti munthu walembedwa muntundayu wavomereza zonse zoyenera asanayambe kutenga nawo mbali mu kafukufuku ameneyu.

Ntolankhani:

Dzina Posayina/Kudinda chala Tsiku ndi Nthawi
