# Supplementary material for: Assessing and Responding to Palliative Care Needs in Rural Sub-Saharan Africa: Results from a Model Intervention and Situation Analysis in Malawi
Source: PLoS One. 2014 Oct 14;9(10):e110457. doi: 10.1371/journal.pone.0110457 (PMC4197005; doi:10.1371/journal.pone.0110457)
Supplement: Appendix S2 — Neno Palliative Care Program initial assessment form. (PDF) [file pone.0110457.s002.pdf]

PIH/MOH Malawi
Palliative Care Program

INITIAL ASSESSMENT FORM

A. Demographics

Last Name:______________________________

First Name:______________________________

Birthdate (Day/Month/Year): ___/___/_______

Age: ______ EMR #: ____________________

Sex: ☐ F ☐ M Marital Status:____________

Caregiver/Next of Kin:____________________

____

Village:________________________________

Referred From: ☐ CCC ☐ KS Clinic ☐ OPD

☐ Health Center ☐ Hospital Ward

☐ Other (Specify):______________________

Reason for Referral:_____________________

____

____

B. Medical History

Presenting Concerns:____

Diagnosis:____

Prognosis:____

HIV Status: ☐ Reactive ☐ Nonreactive ☐ Not Tested

Diagnosis Discussed with Patient: ☐ Yes ☐ No Diagnosis Discussed with Caregiver: ☐ Yes ☐ No

Past Medical History:____

____

History Given By: ☐ Patient ☐ Caregiver ☐ Other (Specify):______________

C. Physical Exam

General	Abdominal
Weight	Extremities
Pulmonary	Neurological
Cardiac	Musculoskeletal

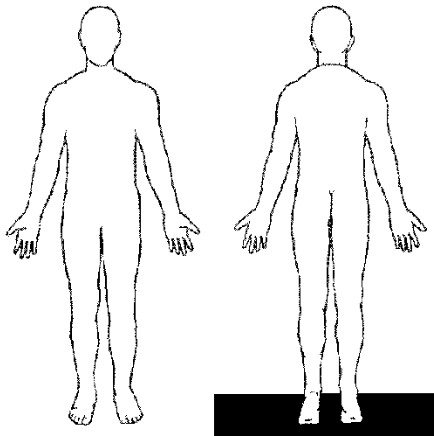

Front Back

D. Palliative Care Outcomes Scale *(Use Visual Symptom Scale Below)*	
Ask the patient to rate the following from 0 (not at all/nothing) to 5 (very much/everything):	
**1) Please rate your pain during the last 3 days: ______ (0-5)**	**3) Have you been feeling worried about your illness in the past 3 days? ______ (0-5)**
**2a) Have you had any other symptoms that have been affecting you for the last 3 days? ______ (0-5)**<br>**2b) If so, please rate each symptom during the last three days (Specify Symptoms Below):**<br>**____________________ ______ (0-5)**<br>**____________________ ______ (0-5)**<br>**____________________ ______ (0-5)**	**4) Over the past 3 days, have you been able to share how you are feeling with your family or friends? ______ (0-5)**<br>**5) Over the past 3 days have you felt that life was worthwhile? ______ (0-5)**<br>**6) Over the past 3 days, have you felt at peace? ______ (0-5)**<br>**7) Have you had enough help and advice for your family to plan for the future?______ (0-5)**
Ask the family/caregiver to rate the following from 0 (not at all/nothing) to 5 (very much/everything):	
**8) How much information have you and your family been given?______ (0-5)**<br>**9) How confident does the family feel caring for ____ (the patient)?______ (0-5)**<br>**10) Has the family been feeling worried about _______ (the patient) over the last 3 days?______ (0-5)**	

Visual Symptom Scale

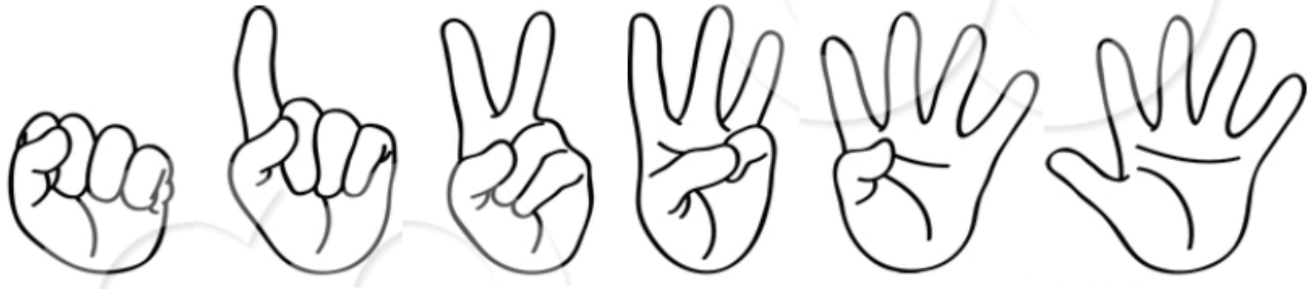

0 = None **5 = Very Much**

E. Pain Management *(Use Visual Symptom Scale Above)*	
**Pain Severity (From 0 to 5):______ (0-5)**<br>**Duration:____________________**<br>**Description of Pain:____________________**<br>____________________<br>____________________	**Pain Affects: Sleep?☐ Y☐ N Mobility?☐Y☐ N**<br>**Current Pain Medication(s):____________________**<br>____________________<br>**Effect of Current Medication(s):☐ None**<br>**☐ Partial Control ☐ Complete Control**

F. Management Plan	
**Patient Referred to POSER for evaluation? ☐ Y ☐ N**	
**Problem:**	**Plan:**
**Problem:**	**Plan:**

Completed By:____________________ **Date: ___/___/______**
