# Supplementary material for: Assessing and Responding to Palliative Care Needs in Rural Sub-Saharan Africa: Results from a Model Intervention and Situation Analysis in Malawi
Source: PLoS One. 2014 Oct 14;9(10):e110457. doi: 10.1371/journal.pone.0110457 (PMC4197005; doi:10.1371/journal.pone.0110457)
Supplement: Appendix S3 — Malawi National Palliative Care Guidelines. (PDF) [file pone.0110457.s003.pdf]

Republic of Malawi
Ministry of Health

NATIONAL PALLIATIVE CARE GUIDELINES

March 2011

FOREWORD IV
ACRONYMS V
ACKNOWLEDGMENTS 1
LIST OF CONTRIBUTORS: 1
CHAPTER 1: INTRODUCTION AND BACKGROUND 2
1.1 INTRODUCTION 2
1.2 BACKGROUND 2
1. 2.1 Situation analysis of Palliative Care services in Malawi 2
1.2.2 Rationale for the Palliative Care guidelines 3
1.2.3 Goal 3
CHAPTER 2: PALLIATIVE CARE GUIDING PRINCIPLES 4
2.1 GUIDING PRINCIPLES 4
2.1.1. Access to care 4
2.1.2 Interdisciplinary and Multisectoral approach 4
2.1.3 Service Delivery Model 4
2.1.4 Ethical and Legal Aspects of Care 5
2.2 PROVISION OF PALLIATIVE CARE SERVICES 5
2.2.1 Palliative care plan 5
2.2.2 Pain control 5
2.2.2.1 Pharmacological measures *5*
2.2.2.2 Non Pharmacological measures *6*
2.2.3 Symptom control 6
2.2.4 Medicines and supplies 6
2.2.5 Nutrition 6
2.2.6 Infection prevention and control 7
2.2.7 Care of Carers 7
2.2.8 Psychosocial care 7
2.2.9 End of life care 7
2.2.10 Grief and Bereavement 8
2.2.11 Paediatric Palliative Care 8
2.2.11.1 Paediatric Pain Control *8*
2.2.11 .2 Special needs for children *8*
2.3 Maintaining Best Practice 8
2.4 Education and Training 9
CHAPTER 3: RESPONSIBILITY AND AUTHORITY 9
3.1 Ministry of Health 9
3.2 Zonal offices 9
3.3 Central Hospitals 9
3.4 District Health Offices: 9
3.5 Health Centre 10
3.8 PACAM 10
3.9 Patients, families and communities 10
CHAPTER 4: MONITORING AND EVALUATION OF PALLIATIVE CARE PROGRAMMES .. 11
4.1 Palliative care Indicators 11
4.2 REPORTING SYSTEMS 12
ANNEXES: (1 - 8) 12

ANNEX: 1 ESSENTIAL PALLIATIVE CARE MEDICINES LIST 12
ANNEX 2: PAEDIATRIC DOSAGES 15
ANNEX: 3 16
SUPERVISORY CHECK LIST FOR PALLIATIVE CARE IMPLEMENTING FACILITIES 16
ANNEX: 4:REFERRAL FORM FOR HEALTH SERVICES IN PALLIATIVE CARE 18
ANNEX: 5 PALLIATIVE CARE PATIENT REGISTER 19
ANNEX: 6 20
PALLIATIVE CARE HOLISTIC ASSESSMENT FORM 20

PROBLEM LIST AND MANAGEMENT PLAN 24

ANNEX 7 THE WHO ANALGESIC LADDER 25
ANNEX 8 NUMERICAL PAIN INTENSITY SCALE 26

NUMERICAL PAIN RATING SCALE 26

DEFINITIONS OF TERMS: 27

REFERENCES 28

FOREWORD

FOREWORD

Malawi like other countries in Sub- Saharan Africa is still faced with the enormous burden of the HIV/AIDS pandemic as well as that of cancer. According to the 2008 Sentinel Surveillance Survey, HIV/AIDS prevalence rates were estimated at 12% among the 15 to 49 years age group. The London declaration on Cancer Control in Africa in 2007 states that 'African countries will account for over a million cases of cancer per year' with an estimated 88-95 percent of cancer patients presenting late or at end stage of disease. It is estimated that 30- 80 percent of patients will have pain in the terminal phase of their disease,. (Katabera 1998, Moss 2000). 25 percent of AIDS patients suffer severe pain during their terminal illness but pain in patients with HIV/AIDS is often under diagnosed and under treated. Effective pain relief has been identified by patients and their families as a main priority to improve their quality of life, and this priority is central to palliative care. In addition, there is also much suffering caused by other symptoms including psychological and spiritual distress. These patients urgently require Palliative Care services which have been highlighted as an urgent need for patients with both HIV/AIDS and cancer and other chronic diseases.

Implementation of palliative care services is regulated by international declarations like the Cape Town Declaration (2002) and the Korean Declaration of (2005). In Malawi Up to 2006, Palliative care services were being championed by CHAM and Non Governmental Organizations. To demonstrate Government commitment for development of palliative care services, from 2006 to date, Palliative care services has been coordinated by the Ministry of Health through the Directorate of Nursing. To promote and facilitate implementation of the service, there is a responsible designated desk officer based at the Ministry of Health Headquarters.

Development of these national palliative care guidelines will provide direction in the provision of palliative care services. Ministry of health is therefore appealing to all government facilities, CHAM institutions and Non Governmental organizations wishing to open palliative care sites and those currently providing palliative care services to operate in line with these guidelines

WILLIE SAMUTE
SECRETARY FOR HEALTH

ACRONYMS

APCA	:	African Palliative Care Association
CBO	:	Community Based Organization
CCW	:	Community Care Workers
CD	:	Controlled Drugs
CHAM	:	Christian Hospitals Association of Malawi.
CHBC	:	Community Home Based Care
CHN	:	Community Health Nursing
CO	:	Clinical Officer
CPD	:	Continued Professional Development
CS	:	Clinical Services
CWZ	:	Central West Zone
EHP	:	Essential Health Package
EN	:	Enrolled Nurse
EOL	:	End of Life
FBO	:	Faith Based Organization
GoM	:	Government of Malawi
HAU	:	Hospice Africa Uganda
HIV	:	Human Immunodeficiency Virus
HPCT	:	Hospital Palliative Care Team
HSA	:	Health Surveillance Assistant
MA	:	Medical Assistant
MO	:	Medical Officer
MOH	:	Ministry of Health
NGO	:	Non-governmental Organisation
NMT	:	Nurse Midwife Technician
ORS	:	Oral Rehydration Salts
PACAM	:	Palliative Care Association of Malawi
PC	:	Palliative Care
PLWHA	:	People Living with HIV and AIDS
PLWC	:	People Living with Cancer
PTB	:	Pulmonary Tuberculosis
QECH	:	Queen Elizabeth Central Hospital
SRN	:	State Registered Nurse
USAID	:	United States AID
SWAP	:	Sector Wide Approach
WHO	:	World Health Organisation

ACKNOWLEDGMENTS

The Ministry of Health in collaboration with Palliative Care Association of Malawi would like to thank all individuals and their organizations who contributed towards the development of this National Palliative Care Guidelines document.
Sincere appreciation goes to Pact Malawi supported by the United States Government and National AIDS Commission for funding the development of this palliative care guideline

List of contributors:
The following individuals dedicated their valuable time, effort and expertise in developing this document:

Sheila Bandazi	:	Director of Nursing services - MOH,
Immaculate Chamangwana	:	Deputy Director of Nursing Services - MoH
Rose Kolola – Nyirenda	:	Director of Mzuzu Central Hospital - MOH
Immaculate Kambiya	:	National CHBC Coordinator – MOH
Evelyn Chitsa-Banda	:	Assistant Zonal Supervisor – CWZ MOH
Lameck Thambo	:	National Coordinator - PACAM
Loveness Nyirenda	:	Principal PC Nursing Officer - QECH
Jane Bates	:	Palliative Care Physician- QECH
Thadeo Macosano	:	Palliative Clinical Officer St. Luke's Hospital
Mercy Pindani	:	Lecturer, Kamuzu College of Nursing
Catherine Chiwaula	:	Principal Nursing Officer - MOH
Francis Chafulumira	:	Director of Central Medical Stores -MOH
Enock Phale	:	Assistant Deputy Director - CS- MOH
Kondwani Banda	:	Palliative care clinical officer – QECH
Annie Chinguwo	:	Master Trainer - Palliative Care.
Angela Odiachi	:	Community, Advisor - USAID
Simon Makombe	:	Treatment, Care and Support Officer – MOH
Henry Ddungu	:	Advocacy Manager, APCA - Uganda
Fatia Kiyange	:	APCA -,Uganda
Kelvin Saukila	:	Chief Statistician, MoH
Grace Bamusi	:	Principal Quality Assurance Officer, MoH
Glenda Winga	:	Operations Manager, PACAM

CHAPTER 1: INTRODUCTION AND BACKGROUND

1.1 Introduction

Palliative care which has recently been highlighted as an urgent need for patients with both HIV/AIDS dates back to the second half of the fourth century when Fabiola opened a hospice for pilgrims and the sick in Italy. Hospice was used for the dying. In 1967, Dame Cicely Saunders established St Christopher's Hospice in London which led to the modern hospice movement which is currently being practiced worldwide. In Sub- Saharan Africa, the first Hospice was established in Harare Zimbabwe in 1979 which spread to South Africa in 1980, Nairobi in 1990 and Uganda in 1993 .In Malawi, the first Palliative care team was established in 2002 in the Pediatrics Department at Queen Elizabeth Central Hospital . Palliative care then spread to CHAM, NGOs, Central and District Hospitals.

Palliative care service delivery is guided by the following two declarations:

The Cape Town Declaration (2002)

- Palliative care is a right of every adult and child (accessibility, affordability)
- Control of pain and symptoms is a human right (drug availability)
- All members of health care teams and providers need training in palliative care
- Palliative care should be provided at primary, secondary and tertiary levels.

The Korea Declaration (2005)

Access to trained hospice and palliative care health care professionals, community volunteers and care workers (family caregivers &carers) via existing health care infrastructures is a worldwide problem.

Governments must:

- Integrate hospice and palliative care education and training into the undergraduate and post-graduate curricula of medicine, nursing, research, and other disciplines.
- Provide training, support and supervision of professional and non-professional care workers
- Strive to make hospice and palliative care available to all citizens in the setting of their choice.

1.2 Background

1. 2.1Situation analysis of Palliative Care services in Malawi

Malawi, like other countries in Sub-Saharan Africa, is tackling the enormous burden of HIV and AIDS pandemic and looming epidemic of cancer. There are more than a million people living with HIV and around sixty one thousand deaths per year are attributable to AIDS. It is estimated that about 25 thousand Malawians live with cancer and there are countless others with other diseases for which there are no curative treatments available at this time. The majority of patients who need palliative care live in rural areas, often far away from their nearest health facility. In the public sector, oncology services are based at Queen Elizabeth and Kamuzu Central Hospitals, focusing on the treatment of paediatric cancers.

There is no radiotherapy service available in the country and currently few patients are being managed through referral to neighboring countries such as Tanzania and South Africa.

Management of patients with cancer from the time of diagnosis requires a palliative care approach with optimal pain and symptom control. Morphine (sustained release tablets) and other essential drugs for palliative care are intermittently available, and research has shown that some health professionals have continued fears about prescribing opiates (Bates 2008).

By October 2009, there were 1585 trained service providers of all categories representing 7 percent of nurses and 6 percent of clinicians. Palliative care services are being delivered at 21 sites; which includes 10 Government Facilities, 7 CHAM Institutions and 4 Non Governmental Organizations.

1.2.2 Rationale for the Palliative Care guidelines

The rationale for setting palliative care guidelines is to enhance the provision of quality services as part of the national health sector response to the HIV and AIDS pandemic and life threatening conditions such as cancer. They will provide guidance and direction towards the implementation of a Palliative Care policy in Malawi. These guidelines are applicable to both the public and private health sector.

The guidelines for PC will be implemented in conjunction with other relevant policies and guidelines such as the HIV policy, CHBC guidelines, national infection prevention control policy and standard guidelines, Antiretroviral Therapy guidelines, nutrition and HIV guidelines, community integrated management of childhood illness guidelines etc

1.2.3 Goal

The goal of the guidelines is to streamline the provision of PC in Malawi, through the elaboration of key steps/processes required for quality service provision.

Specific Objectives for the Guidelines

- To provide direction for the establishment and implementation of quality palliative care services in institutions and communities.
- To promote access to quality palliative care services, including pain and symptom control.
- To provide a basis for lobbying availability, accessibility, safe handling and rational use of opioids for pain management, and other palliative care medications
- To provide basis for the development and implementation of palliative care standards in Malawi.

CHAPTER 2: PALLIATIVE CARE GUIDING PRINCIPLES

Palliative care is patient and family centered care. It optimizes quality of life by active anticipation, prevention and treatment of suffering. It emphasizes use of an interdisciplinary team[1] approach throughout the continuum of illness, placing critical importance on the building of respectful and trusting relationships. Palliative care addresses physical, intellectual, emotional, social and spiritual needs. It facilitates patient autonomy, access to information and choice. (HRSA care action, July 2000)

2.1 Guiding principles

Guiding principles of comprehensive palliative care service delivery shall include:

2.1.1 Access to care

- Palliative care is a right of every adult and child[i] therefore, it must be included in the Essential Health Care Package
- Patients and their families shall access holistic palliative care which aims to meet their physical, psychosocial and spiritual needs within their cultural context.
- Palliative care patients shall be referred to appropriate levels of palliative care service delivery

2.1.2 Interdisciplinary and Multisectoral approach

- Palliative care shall be provided by an interdisciplinary[1] team.
- Where an interdisciplinary team is not available, a core team shall be oriented on palliative care to ensure that all needs are met.
- Members of the team shall communicate and network the care of the patient and family through regular meetings to discuss case studies in order to share experiences, understand problems and identify appropriate solutions.

2.1.3 Service Delivery Model.

- Institutions, guided by the WHO Palliative Care Program Principles[2], shall choose a suitable model depending on their setting and resource availability without compromising quality of services.

The model shall be:

- Developed as a comprehensive and public health approach

[1] Team comprising of the patient, health care workers, allied health workers, spiritual leaders, social workers family and community members.

[2] Cecilia Sepulveda. From Concepts to Reality: Palliative Care in Resource-Constrained Settings for People Living with HIV and Other Life-Threatening Illnesses: The World Health Organization Approach

- Integrated within existing health care delivery systems in both public and private sector for scale up of the continuum of care for chronic , life-threatening illnesses
- Tailored to the specific cultural and social context.

2.1.4 Ethical and Legal Aspects of Care

- The intention of palliative care is to improve the quality of life of patients therefore care and support shall be provided for the benefit of the patient and family whilst causing them no harm.
- The patient's goals, preferences and choices shall be respected according to the laws of Malawi, and shall form the basis for the plan of care.
- Rights and ethical consideration for the patient shall be observed as outlined in Palliative Care Training Manual for Health Professionals
- When a child's wishes differ from those of the adult decision-maker, appropriate professional staff members shall be made available to assist the child.

2.2 Provision of Palliative Care Services

2.2.1 Palliative care plan

A patient requiring palliative care shall have a detailed holistic assessment and care plan developed by the palliative care provider in collaboration with the patient and family in order of priority.

2.2.2 Pain control

Effective pain control is central to palliative care using both pharmacological and non pharmacological measures. Providers shall be able to control pain according to WHO analgesic ladder.

2.2.2.1 Pharmacological measures

- The WHO analgesic ladder is the fundamental approach to all types of pain including somatic and neuropathic pain , and shall be used as the standard approach to the management of pain
- Pain control drugs shall be administered regularly – by the patient, by the clock, by the ladder, and by the mouth
- Opioids are indicated for the control of moderate-to-severe pain among patients with HIV and/or Cancer as well as other painful disease conditions.
- Prescription of opioids shall be carried out according to the laws of the government of Malawi – a registered doctor, clinical officers, and dental surgeon
- If there is no prescriber of opioids, patients shall be referred to the nearest health facility.

- Supply, storage, prescription, dispensing, receipts and consumption of Opioids shall follow the legal provisions and regulations as stipulated in the Controlled Drug Act[3].

2.2.2.2 Non Pharmacological measures

Non-pharmacological pain management is the management of pain without medications. This method utilizes ways to alter thoughts and focus concentration to better manage and reduce pain. Methods of non-pharmacological pain management shall include:

- Education of the patient and family on the condition to provide insight and support.
- psychosocial care - therapy/counseling ,individual counseling, family counseling, companionship, music, art, or drama therapy, and group counseling
- Physical care – which may involve the following: Exercises, heat/cold application, lotions/massage therapy, positioning, etc
- Spiritual care such as meditation and pastoral counseling

2.2.3 Symptom control

The general approach to symptom control in palliative care shall include:

- Assessment for the cause and severity of the symptom
- Treatment of reversible causes;
- Initiation of disease/symptom-specific medicines and non-drug measures
- Involvement of the patient and family on the management plan

2.2.4 Medicines and supplies

- Medications for symptom control including essential medications for opportunistic infections shall be made available for palliative care service provision in each District.
- Medicines, equipment and consumables required shall be made available as outlined in the essential palliative care drugs list (annex 1 and 2.)
- Palliative care medicines including antiretroviral drugs shall be dispensed free of charge at the service delivery point

2.2.5 Nutrition.

- Nutrition support has been shown to benefit palliative care patients by reducing physical deterioration, improving quality of life, and preventing the emotional effect of "starving the patient to death."
- Palliative care patients of all age groups shall be encouraged to eat the six food groups (vegetables, animal products, fruits, legumes, staples and fats and oils)
- The successful management of these drug food interactions requires understanding clients' individual food access as well as eating habits. Locally available foods are recommended.

- Management of patients shall include assessment and counseling on feeding with regard to the nutritional needs specific to the stage of the illness.

[3] Refer to CDA chapter/section

- Guardians shall be counseled on appropriate feeding according to the stage of the illness

2.2.6 Infection prevention and control

Palliative care services shall operate in accordance with National Infection Prevention and Control Policy and standard guidelines to minimize the risk of infections in patients, families and care providers in order to promote a safe caring environment.

Core infection prevention and control interventions shall include:

- Hand hygiene
- Use of personal protective equipment
- Isolation precautions
- Aseptic technique
- Cleaning and disinfection and
- Sterilization

2.2.7 Care of Carers

- The palliative care team shall be assisted to recognize the difficult situations they encounter, personal limitations and ways of utilizing effective coping strategies.
- Carers shall be provided with adequate resources for patient care.
- Regular team meetings and social gatherings shall be promoted to help reduce stress and burnout.
- Supervision, training and support shall be provided to health workers, family and community members.

2.2.8 Psychosocial care

- Psychological issues shall be assessed and managed based upon the best available evidence.
- Referrals to health care professionals with specialized skills shall be made available when appropriate.

2.2.9 End of life care

- Health care providers shall prepare both the patient and the family on the impending death
- Care provider shall be honest, attend to emotional responses and spiritual needs.
- Care providers shall maintain presence and talking to the patient even if the patient is unconscious. This practice shall be promoted.
- Comfort measures shall be provided depending on the presenting signs and symptoms of impending death.
- End-of-life concerns, hopes, fears, and expectations shall be openly and honestly addressed in the context of social and cultural customs in a developmentally appropriate manner.

2.2.10 Grief and Bereavement

- Grief and bereavement risk assessment shall be done routinely throughout the illness trajectory
- Care providers (family, friends, social and religious communities) shall provide a safe, comforting place to the bereaved family to enable them express their feelings, thoughts and needs as they are going through bereavement.

Customary and religious rituals shall be respected to help the family cope with death

2.2.11 Paediatric Palliative Care

Palliative care for children focuses on enhancement of quality of life for the child and support to the family. Emphasis shall be on pain assessment, psychological support and communication which shall be appropriate for the age and developmental stage of the child.

2.2.11.1 Paediatric Pain Control

- Pain assessment tools shall be age appropriate (annex 8).
- Aspirin is contraindicated in children under 12 years.
- Dosages shall be calculated in kilogram per body weight (annex 2)

2.2.11 .2 Special needs for children

- Special needs shall be identified through comprehensive assessment and addressed holistically.
- Children shall be involved in decisions about their own care.
- Recreation activities such as play activities, drawings, poems or songs shall be encouraged .
- Appropriate information according to age shall be communicated in clear and simple language at their pace
- Children shall be allowed to lead a normal life which includes access to education within the limitation of their illness. School teachers, community members including other children shall be encouraged to support and deal sensitively with the affected child
- Palliative care providers shall take into consideration the needs of orphans and vulnerable children and shall refer them to appropriate services for care and support

2.3 Maintaining Best Practice

- The palliative care team shall seek to maintain up to date skills in their area of work through Continuing Professional Development (CPD), refresher courses, regular clinical meetings – e.g. case conferences, refresher courses and journal clubs; personal reading, case study review and research.
- Palliative care providers shall always adhere to standard operating procedures as provided
- Treatment decisions shall be based on goals of care, assessment of risk and benefit, best evidence, and patient/family preferences.

- Treatment alternatives shall be documented and communicated clearly to permit patient and family make informed choices.
- Continuous monitoring and evaluation shall be provided

2.4 Education and Training

- Palliative care service providers shall be trained in palliative care. Training shall be appropriate for the cadre and their role in the interdisciplinary team.
- Palliative care concept shall be incorporated in the pre-service curricula for health training institutions
- Post-graduate training to specialization in palliative care medicine shall be encouraged
- The National Palliative Care training manual shall be used during the 5 day introductory course
- Trainings shall be coordinated and certified by MOH
- MOH in collaboration with relevant stakeholders shall establish resource centers and organize refresher courses to update service providers.

CHAPTER 3: RESPONSIBILITY AND AUTHORITY

There are various levels of responsibility regarding the implementation of Palliative Care: The roles and responsibilities shall be as outlined:

3.1 Ministry of Health

The ministry of Health through **the Directorate of Nursing:**

- Shall provide leadership and coordination of Palliative Care Services.
- Shall identify and prioritize specialized training needs in palliative care

3.2 Zonal offices

- Shall be responsible for monitoring adherence to the guidelines at district hospital level.
- Shall supervise, monitor and evaluate the implementation of palliative care services

3.3 Central Hospitals

- Shall offer tertiary palliative care services
- Shall network with home based care groups and other health facilities for referral
- Shall keep appropriate records and compile monthly reports

3.4 District Health Offices:

- Shall adhere to standards and guidelines in the management of palliative care patients
- Shall allocate financial resources in District implementation plan (DIP) for implementation of palliative care activities at the district and community levels
- Shall be responsible for implementing, coordinating, supervising and auditing palliative care services at all health facilities within the district.
- Shall be responsible for training and certification of service providers.

- Shall designate an officer to monitor implementation of palliative care services as provided by NGOs, FBOs and CBOs at district level.
- Shall keep appropriate records and compile monthly reports

3.5 Health Centre

- Shall develop a palliative care team with involvement of their local communities (including community volunteers) to provide services
- The team shall be responsible for identification, management, follow up and referral of patients
- Shall keep appropriate records and compile monthly reports which shall be submitted to the district coordinator

3.6 Pharmacy, Medicine and Poisons Board

- The board shall be responsible for regulating and reporting on the importation of morphine and other Opioids used for palliative care.
- The board shall review legislation on a regular basis to improve access to opiates

3.7 Central Medical Stores and facility pharmacy

- shall be responsible for availability and accessibility of all essential palliative care medicines including morphine
- shall be responsible for supervision of safe handling , storage and reporting of opiates at provider sites
- shall keep accurate records of all transactions on opiates

3.8 PACAM

In collaboration with MOH:

- Shall provide supervision to implementing sites
- Shall provide support for training of trainers
- Shall identify resources for Continued Professional Development.
- Shall advocate for palliative care services
- Shall conduct annual conferences on best practices and update members on emerging issues in Palliative care.
- Shall provide technical support for palliative care services
- Shall collaborate with national and international palliative care bodies
- Shall monitor adherence to palliative care guidelines and standards
- Shall mobilize resources for palliative care

3.9 Patients, families and communities

- Shall be actively involved and contribute towards self care
- Shall work in collaboration with health professionals and CBOs/FBOs/NGOs in their catchments area.
- Shall be involved in establishment and review of palliative care services.
- Shall advocate for better access to palliative care

CHAPTER 4: MONITORING AND EVALUATION OF PALLIATIVE CARE PROGRAMMES

Monitoring and evaluation shall be used as advocacy tool for use of evidence based decision making. Monitoring shall be conducted at all levels using appropriate indicators. Reviews shall be done annually to assess programme performance by comparing baselines against set target

4.1 Palliative care Indicators

Process Indicators	Definition of indicator/Measurement	Method of data collection	Frequency
Percentage of health professionals (nurses, doctors, clinical officers) trained and providing palliative care services	**Numerator:** N*umber of trained professional health workers providing palliative care services* **Denominator:** *Total number of professional health workers trained in palliative care*	Training records	Baseline/ Quarterly
Number and percentage of palliative care sites with minimum staff norms (1 trained nurse and 1 trained clinician)	***Denominator*** *– total number of sites currently providing palliative care services*	Supervision reports	Biannually
Number of drug day availability: level 1 aspirin, level 2 codein, level 3 morphine	***Denominator*** *365 days*	Supervision reports	Biannually
Total number of patients receiving palliative care services	***Denominator*** *– total number of patients registered for palliative care*	Supervision reports	Biannually
Percentage of palliative care sites supervised at	***Denominator*** *– Total number of sites currently providing palliative*	Quarterly reports	Quarterly

least twice a year	*care*		
Percentage of palliative care health facilities with resources (minimum of guidelines, pain medications, essential supplies)	**Numerator:** Number of PC accredited health care facilities with resources<br><br>**Denominator**: Total Number of PC accredited Institutions	Structured Audit tool with on-site inspections	Baseline/Quarterly
**Out puts indicators**			
Proportion of patients seen at home	Numerator: *Number of palliative care patients seen at home*<br>Denominator: *Total number of palliative patients on home care*	Health care records	Baseline/Monthly/Quarterly
Percentage of patients seen as inpatients in a health facility	Numerator: number of palliative care patients seen as inpatients<br>Denominator: *Total number of inpatients*	Health facility/Palliative care unit records	Baseline/Monthly/Quarterly
Outcome indicators			
PC coverage: Percentage of accredited palliative care health facilities providing minimum package of services	Numerator: *Number of accredited palliative care health facilities*<br><br>Denominator: *Total number of health facilities capable of providing minimum package of palliative care services*	Ministry of Health and Palliative care association records (Health service records)	Baseline/Quarterly
**Impact**			
Quality of palliative care services	Qualitatively	Client and family questionnaires	Every 2-3 years

4.2 Reporting Systems

Community services providers shall compile reports monthly to the nearest health facility, who will then submit to the district, then district and central hospital shall submit quarterly to the Ministry of Health (Nursing Directorate) using the Standardized forms (annex 4). PACAM shall obtain a copy from the MOH.

ANNEXES: (1 - 8)

ANNEX: 1 ESSENTIAL PALLIATIVE CARE MEDICINES LIST.

Drug Name	Properties	Clinical Uses	Alternative Drugs
Paracetamol	Non opioid<br>Analgesic<br>Antipyretic	Fever<br>Pain	
Aspirin	Non opioid<br>Analgesic<br>Antipyretic<br>Anti-inflammatory	Pain<br>Fever<br>Sore Mouth	

Ibuprofen	NSAID	Pain (esp. bone pain)<br>Fever<br>Anti inflammatory	Diclofenac<br>Indomethacin
Tramadol	Weak opioid<br>Analgesic	Pain	Codeine
Morphine liquid	Strong opioid<br>Analgesic	Pain<br>Introduction<br>Breakthrough pain<br>Difficulty swallowing<br>children<br>Breathlessness<br>Severe Diarrhoea	Morphine slow release tablets
Morphine (slow release tablets)	Strong opioid	Pain<br>Severe diarrhoea	Morphine liquid
Dexamethasone	Corticosteroid<br>Antinflamatory	Painful swelling and inflammation<br>Poor appetite	Prednisolone
Amitriptyline	Tricyclic<br>Antidepressant	Neuropathic pain (nerve pain)	Carbamazepine<br>Phenytoin
amitriptyline	Tricyclic antidepressant	depression	imipramine
Hyoscine Butyl bromide (Buscopan)	Antimuscarinic<br>Antispasmodic	Abdominal pain (Colic)	propantheline
Diazepam	Benzodiazepine<br>Anticonvulsant	Muscle spasm<br>Seizure<br>Anxiety, sedation	Lorazepam
Phenobarbitone	Anticonvulsant	Seizure	Diazepam
Metoclopramide	Antiemetic	Vomiting	Haloperidol<br>Domperidone<br>promethazine
metoclopramide	Pro-kinetic	Abdominal Fullness	
Chlorpromazine	Antipsychotic	Hiccups	Metoclopromide<br>Nifedipine
Magnesium Trislicate	Antacid	Indigestion<br>Gastro-oesophageal reflux<br>gastritis	Aluminium Hydroxide<br>Magnesium Hydroxide<br>Ranitidine<br>cimetidine
Loperamide	Antidiarrhoeal	Chronic diarrhoea	Codeine<br>Morphine
Bisacodyl	Stimulant laxative	Constipation	Sennakot
ORS	Rehydration Salt	Diarrhoea	

		Rehydration	
Chlorpheniramine	Antihistamine	Drug reactions	Promethazine
Flucloxacillin	Antibiotic	Chest infection<br>Skin infection	Erythromycin
Cotrimoxazole	Broad Spectrum Antibiotic	PCP treatment and prophylaxis<br>Infective diarrhoea in HIV/AIDS<br>Urinary Tract Infection	Ciprofloxacin<br><br>Amoxicillin, nitrofurantoin,
Metronidazole	Antibacterial for anaerobic infections	Foul smelling wounds<br>gingivitis<br>dysentery<br>Vaginal discharge	Nalidixic acid
Lumefantrine artemether(LA)	Anti- malarial	Malarial treatment	Quinine sulphate
Acyclovir	Antiviral	Herpes zoster	
Chloramphenicol eye ointment/drops	Antibacterial	Eye infections	Tetracycline, Gentamycin, ointment & drops
Fluconazole	Antifungal	Oral and Oesophageal candidiasis<br>Cryptococcal meningitis	Triconazole<br>Miconazole
Clotrimazole 1% Cream	Topical antifungal	Fungal Skin Infection	Whitfield ointment<br>Miconazole.<br>Griseofulvin
Nystatin Suspension and pessaries	Antifungal	Oral and vaginal candidiasis<br>Prophylaxis for patients on steroids	Clotrimazole pessaries Triconazole<br>Miconazole<br>GV paint
Petroleum jelly	Skin moisturizer and protection.	Dry skin<br>Pressure area care.	Emulsifying ointment
Potassium permanganate	Drying agent antiseptic	Oozing lesions<br>wet skin	
Gentian Violet Paint	Antimicrobial<br>Astrigent.	Bacterial & fungal skin infection	Clotrimazole pessaries<br>Nystatin Triconazole<br>Miconazole
Chlorinated Lime	disinfectant	Infection prevention	chlorine
Calamine Lotion	Itch	Rash	Aqueous Cream<br>10% salicyclic acid

Consumables

Gauze
Bandages
Cotton wool
Crepe bandage
Catheters
Gloves
Incontinence pads
colostomy bags
Plaster

ANNEX 2: Paediatric Dosages.

Drug	No. times/day	Single dose by weight	Approximate single dose by age*		
			< 1yr	1 – 5 yrs	6 – 12 yrs
Paracetamol for pain or fever	4	10 - 20mg/kg	62.5mg	125mg	250 - 500mg
Ibuprofen for pain or fever	3	5 - 10mg/kg	50mg	100mg	100 - 200mg
Codeine for moderate pain or diarrhea	4 - 6	0.5 - 1mg/kg	7.5mg	15mg	30mg
Oral morphine for severe pain	6	starting dose 0.1 - 0.3mg/kg	1 - 2mg	2.5mg	2.5 - 5mg
Bisacodyl for constipation	1	5mg total	5mg	5mg	5mg
Dexamethasone **	2, morning & lunchtime	0.1 - 0.5mg/kg	0.5 - 1mg	2mg	4mg
Prednisolone ** (if dexamethasone not available)	2, morning & lunchtime	1 - 2mg/kg	5mg	15mg	30mg
Amitriptyline for neuropathic pain	1 at night	0.2 - 0.5mg/kg max 2mg/kg	-	6.25mg	12.5mg
Metoclopramide for nausea/vomiting	3	0.1 - 0.5mg/kg	5mg	10mg	10mg

Loperamide for chronic diarrhoea (NB not for use in acute GE)	3	0.1 - 0.2 mg/kg	-	1mg	2mg
Diazepam for muscle spasm or agitation	2	0.25mg/kg	1.25mg	2.5mg	5mg
Chlorpheniramine for itching or night sedation	3	0.1mg/kg	0.5mg	1mg	2mg
Nystatin suspension for severe candida	3	1 drop	1 drop	1 drop	100mg

*These doses are given for guidance, taking into account the formulations most commonly available. Where liquid formulations are available, more accurate dosing using mg/kg is advised

** High doses are used for spinal cord compression and raised intracranial pressure. Lower doses (given above by weight) are used for reducing tumour mass causing obstruction, oedema or nerve compression. Short courses are advised, which can be repeated. If given for more than a week, steroids should be tailed off gradually. In some cases a maintenance dose may be necessary; this should be the lowest dose needed to control symptoms. Cover with antifungals in the immunosuppressed and those on long courses.

ANNEX: 3

SUPERVISORY CHECK LIST FOR PALLIATIVE CARE IMPLEMENTING FACILITIES

Name of Facility…………………………………………………………
Name of District……………………………………………..…………
Name of district palliative care coordinator: ………………….……………
Name of facility palliative care coordinator: …….…………..……………….
Contact address: …………………………………………………..………..…
TEL/CELL Number of facility coordinator..……………………………..…

1. **0 Capacity Building**

1.1 Do you have a palliative care Team? Y N

If no state reasons ---

If yes, give Composition of the Palliative care team by cadre and gender:

**Cadre**	**total**	**Number trained**	**trained and providing services**	TRAINED THIS QUARTER
Drs				
COs				
SRNs				
EN/NMTs				
Physiotherapist				
Pharmacy technicians				

MAs				
Volunteers				
Other (specify)				

1.2 Number of Palliative Care Trainings conducted in this quarter. Specify type of training and number trained by cadre and gender
(In-service, Initial, Refresher, orientation)

1.3 Number of palliative care team meetings conducted in this quarter? (Verify by checking minutes)

----

2. SERVICE PROVISION:

2.1 Indicate model of care by ticking in the box.
In patient care { } Outpatient { } Day care { } Home based care ({ }
Other s please specify

2.2 Indicate conditions and number of patients cared for during the quarter)? Report children separately

	M	F
Cancer		
AIDS		
Cancer+ AIDS		

Other conditions (please specify)

2.3 Number of patients referred to other services (specify type of service)

2.4 Mention type of support and organization and/or institutions networking with. .Please Specify names of institutions/ organizations and type of support :
Management support --
Transport for supervision---
Drugs and supplies,
Financial support---2.5Mention Challenges experienced in the implementation of palliative care services : ----------------------
----

Annex: 4:Referral form for health services in palliative care

Date:------------------------------Name of patient --------------------------------------<br>Sex --------------------- Age ------------------------------- Religion ---------------------<br>Occupation -------------------------- Marital status ---------------Tribe --------------<br>Physical address of patient---<br>--<br>--.<br>Name and address of next of Kin ---.<br>Relationship– of next of kin ---<br>Name of carer --<br>Relationship to patient--
Diagnosis(specify)---<br>Patient aware of diagnosis Y/ N<br>Carer aware of diagnosis Y /N
Main problems ---<br>--<br>---
Current treatment ---<br>--<br>Advice/counseling given on;<br>Symptom management;--<br>Care--<br>---<br>Other---
Referred from: (Full Address)--<br>--<br>Referred To: ---<br>Reason for referral--<br>--

Referred by---------------------------------Authorized Signature--------------------
Phone number--

(*Name and Designation*)

Annex: 5 Palliative care patient register

Patient Reg. no.	Date	Patient name	sex	age	Marital Status	Physical address	Referred from	diagnosis	Outcome Date (discharge/ referred/ died)

Annex: 6

Palliative care holistic assessment form

Patient Name<br><br>Contact Tel.	Date seen

Marital status<br><br>Tribe	physical address/location	Religion<br><br>Occupation	

Name of Next of kin /carer<br><br>Relationship with patient<br><br>Contact Tel.	**History from-**<br><br>Patient { } Carer { } other ( )	**Referred from**<br><br>CHBC ( )<br><br>Health Centre( )<br><br>OPD ( )<br><br>Hospital ward ( )

**Referred by :**<br><br>Dr /Clinician ( )<br><br>Nurse (<br><br>Com Vol ( )<br><br>Other ( )	**HIV status:**<br><br>Positive ( )<br>Disclosed ( )<br><br>Negative ( )<br><br>Unknown ( )<br><br>If unknown check clinical diagnosis / staging table	**Reason for referral :**<br>Pain Control ( )<br><br>Symptom Control ( )<br><br>Psychological support ( )<br><br>Other ( )
Diagnosis of patient(if	Diagnosis	**PAST MEDICAL AND SURGICAL** HISTOR

available)	discussed with carers no / yes	[illegible] about diabetes, hypertension, TB, past hospitalizations, [illegible]r [illegible] information)

Drug History

Previous medications(include steroids, opiods,chemo, ARVs, herbal medicine)

History of drug allergy/adverse drug reactions

Present Medications (All medications including ARVs Prophylaxis , Opiods)

If patient is receiving Opiods eg Morphine indicate dosage

History of present illness and treatment to date

(Include description of symptom noted and main concern)

Pain and symptom history Symptoms: 0 absent; + mild; ++ moderate +++ severe

Symptom	0	+	++	+++	Comments (incl onset)	Symptom	0	+	++	+++	Comment
Anorexia						Dry mouth					
Nausea						Skin Rash					
Vomiting						Skin Itch					
Dysphagia						Edema					
Painful Swallowing						Arthralgia (specify joints)					
Sore Mouth						Fatigue					
Dyspnoea						Confusion					
Cough						Drowsiness					
Headache						Diarrhea					
Paralysis						Other					

Pain chart

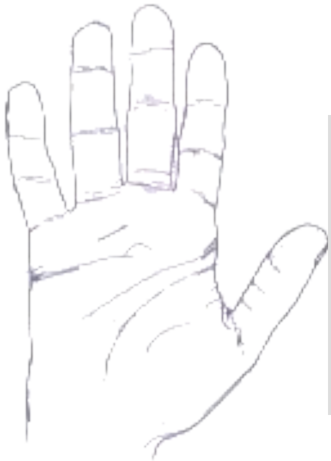

Plot each Pain Score into the graph below each time you see the patient please. Add NEW pains when they occur.

Visit		1st	2nd	3rd	4th	5th	6th	7th	8th	9th
**PAIN SCORE (Scale of 0-5)**	5									
	4									
	3									
	2									
	1									
	0									

Keys (symbols) for the different types of pain: If a new symbol is used, please indicate it below.

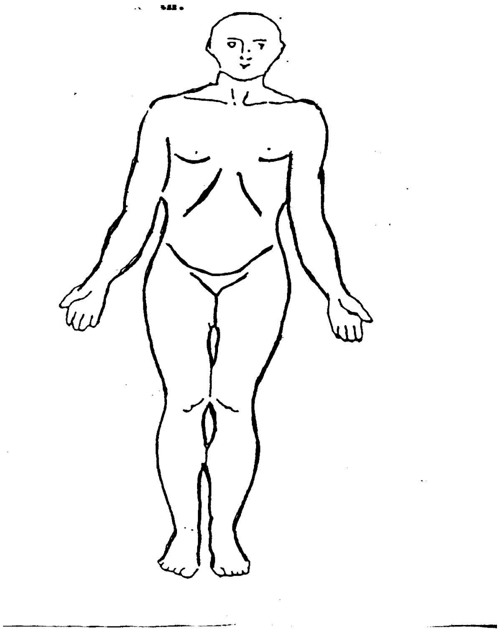

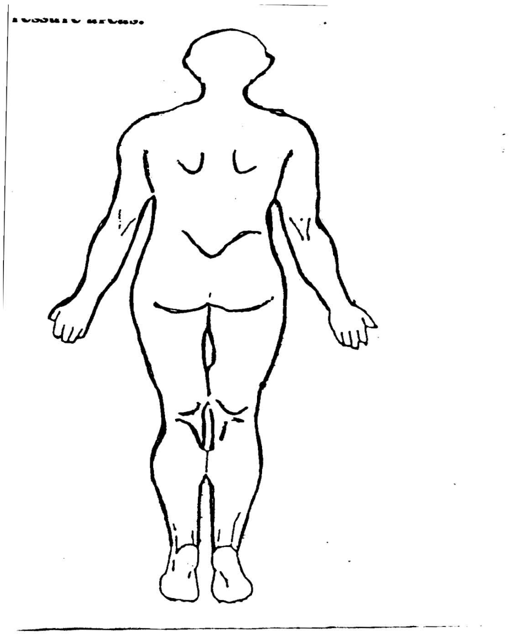

	Pain 1	Pain 2	Pain 3	Pain 4
Duration of pain				
Character/ description of pain				
Numerical Rating Scale (**0-5**)				
Periodicity (Constant /Intermittent)				
Precipitating Factors				
Relieving Factors				
Does pain affect sleep? Y/N				
Does pain affect mobility? Y/N				
Effect Of Current Medication – None, Partial, Complete Control				

SPIRITUAL ASSESSMENT-

What is your relationship with God?
What brings you hope?
Has your illness affected your Relationship with God?
Are your church member's visiting you?…………..
Do you have Fears/issues which are causing you distress?

PSYCHOSOCIAL HISTORY

What is source of income**?---**

What is the families main distress?, ……………………………………….

What are the ages of your biological children? , (oldest – youngest)…………………………………………… ………….………….

Number of children in school and

	class……………………… How is the family /community supporting you?......................... What cultural beliefs are associated with the illness, …………… Has the illness affected any close relationship………………………… Relationships?(Explore on sexuality as well),……………………

Physical Examination General condition:	Weight	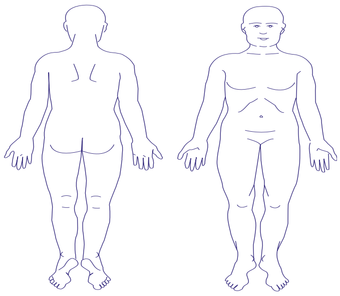
Chest	Neuro	
Abdominal	Other	

Diagnosis discussed with carers no / yes
Diagnosis discussed with patient no / yes

Problem list and Management Plan

Problem<br>Please list and number each problem (previous and new)<br><br>No. Problem		Management Plan<br>For each new and old problem note a brief management plan including non-pharmacological and pharmacological approaches. If problem no longer exists please explain why.

Annex 7 **The WHO analgesic ladder**

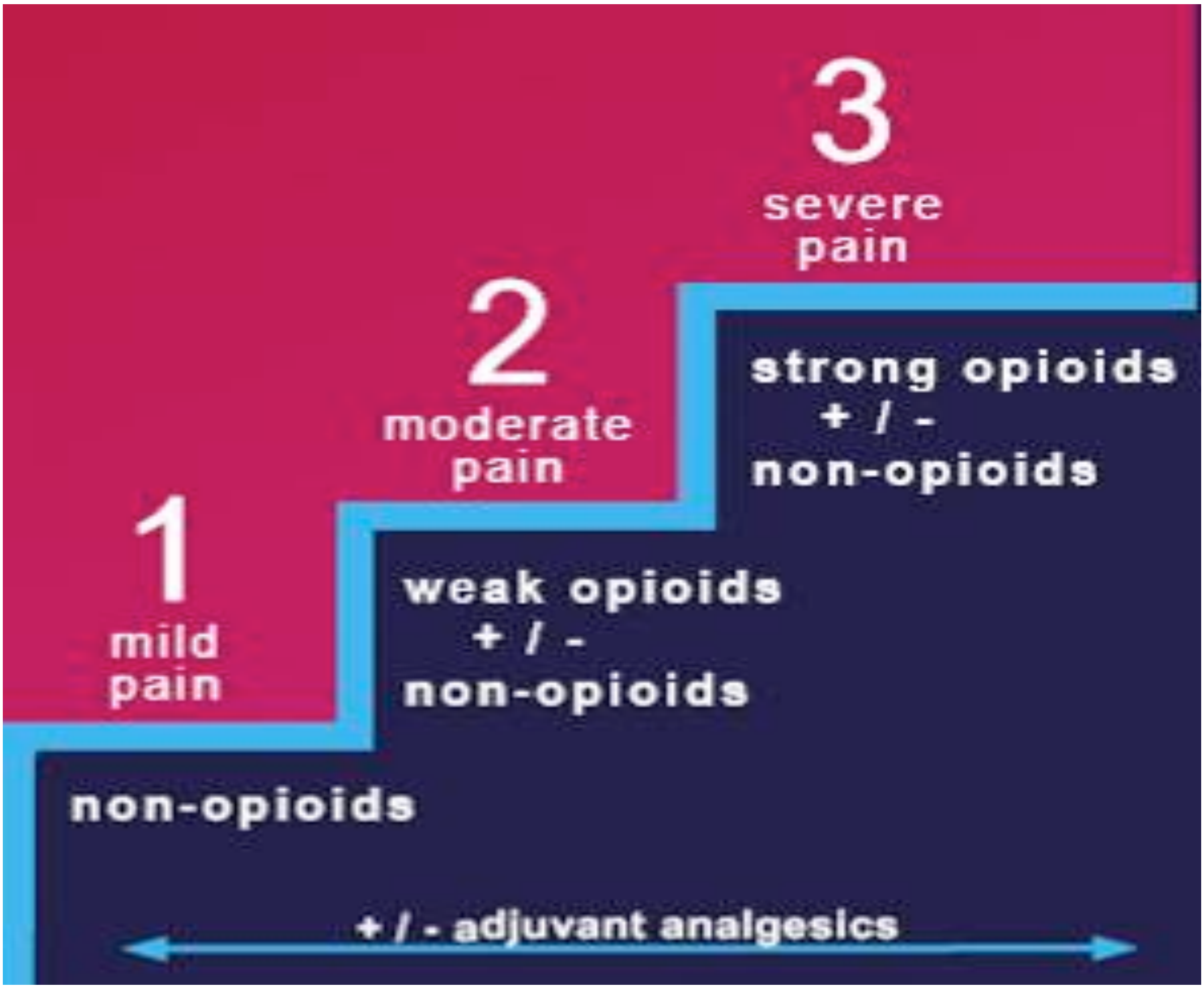

Annex 8   Numerical Pain Intensity Scale

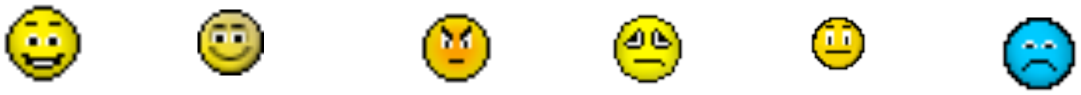

0 1 2 3 4 5 6 7 8 9 10

No pain   mild pain   moderate pain   severe pain   very severe pain

Numerical Pain Rating Scale

I Do not have any pain   0______1_____2______3______4______5   My pain could not be worse

Show on your fingers how severe is pain… 5 is most severe.

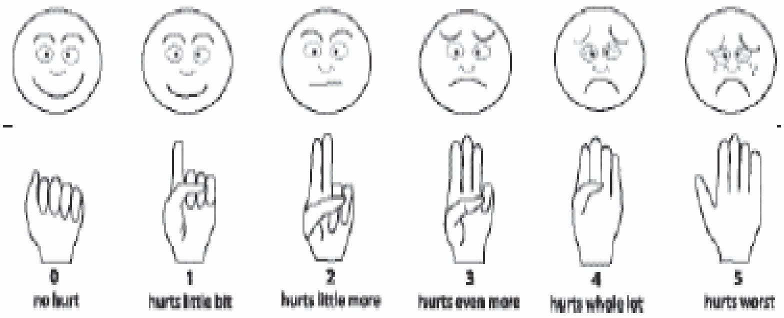

Definitions of terms:

Analgesic Ladder - *A three-step approach of administering the right drug in the right dose at the right time in the following order: non-opioids (aspirin and paracetamol); then, as necessary, mild opioids (codeine); then strong opioids such as morphine, until the patient is free of pain. .*

Bereavement– *the period of grief and mourning after a loss or death.*

Community Home Based Care : *Care given to an individual in his/her own natural environment not only provision of the physical and health needs , but also the spiritual , material and psycho-social needs Palliative Care : is an approach that improves the quality of life of patients and their families facing problems associated with life-threatening illness, through the prevention, and relief of suffering by means of early identification and impeccable assessment and treatment of pain and other problems, physical, psychosocial and spiritual. (WHO 2002)*

Day Care; *Caring for patients for the day away from their usual environment, where they can share with others, receive medical care and other therapies if available, a meal and entertainment*

End of life*, Special time before death when the patient and family require holistic support.*

Evaluation *Systematic process of attributing outcomes to their causes.*

Grief: Normal *process of reacting to a loss expressed through mental (anger, guilt, anxiety, sadness and despair), physical, social or emotional reaction.*

Health professionals *– All cadres of health care workers registered by the Medical Council of Malawi, Nurses and Midwives Council of Malawi and Pharmacy, Medicines and Poisons Board*

Home Based Palliative Care: *Provision of palliative care for the patient and family in the home.*

Indicator: *a unit of information, measured over time, that documents change*

Inpatient Care: Provision of palliative care in the hospital setting

Inpatient model shall use a dedicated unit in a general ward/specialist hospital, identified room within the hospital setting or a separate free standing unit within a hospital complex or a free –standing unit geographically separate from any other hospital and have appropriately qualified multidisciplinary staff trained in palliative care committed to offer 24 hours palliative care services

Monitoring *Systematic process of collecting, analyzing and using information to track performance of an organization in achievement of goals.*

Opiates: *Substances having "addiction-sustaining liability similar to morphine".*

Opioid *– all drugs either natural or synthetic with morphine-like actions – e.g. morphine, codeine etc.*

Reporting systematic and timely provision of useful information at specific periodic intervals.

.REFERENCES

1. Basemera B (2004) *'introduction to communication skills'* Hospice Africa Uganda.

2. Benson, T. F. and Merriman, A. (2006) 'Palliative medicine pain and symptom control in the cancer/and AIDS patients in Uganda and other African Countries' Fourth edition (the Blue Book)

3. Green K, Knh L and Khue L (2006) Findings from a Rapid Situation Analysis in Palliative care in Five Provinces, Viet Nam.

4. Grand E, Murray SA, Grant A, Brown J. '*A good death in rural Kenya? Listening to Meru patients and their families talk about care needs at end of life'*

5. *Katabera E.* (1998) in Moss V.(2000): Personal communication from Dr Elly Katabera, mulago Kampala and Dr Veronica Moss, Mildmay International, Kampala to Dr.Annie Merriman
6. Keiza K, (2002) '*importance of will in matters of inheritance'* Palliative Care Manual for health professional. (1st edn) Hospice Africa Uganda.

7. Kubler-Ross E (1969) '*on death and dying'* Tavistock, London

8. Merriman A (2002) '*Death and Dying'* (1st edn) Hospice Africa Uganda.

9. MOH (2005) Community Home based care policy and guidelines

10. MOH (2005) CHBC training manual

11. MOH (2008) Palliative care training manual for health workers

12. Mpanga Sebuyira, Lydia (2006) *'Hospice Africa Uganda; Present and Future'* Presentation at Advocacy Drug Workshop, Uganda

13. Doyle D, Hanks GWC, MacDonald N(eds): Oxford text book of palliative medicine, 2nd Edition, 1998.

14. Goldman A, Han R, Liben S, 2005, Pediatric Palliative care in Oxford Text book of Palliative Care for Children. 1st edition. London: Oxford Press.

15. Palliative Cancer Care Guidelines (2007) *scottish partnership agency with the clinical resource and audit group*

16. Palliative Care Standards for Uganda (2007)

17. Parry E.H. 'Principles of medicine in Africa' Oxford University press

18. Robbins RA (1995) 'Death anxiety, death competency and self – actualization in hospice volunteers' Hospice J. 7: 29-35.

19. Simon Makombe, Edwin Libamba, Eustice Mhango, Olga de ascura Teck, John Aberle, Mindy Hochgesang, Erik J. Schouten, Antony D harries, Harding R, Higgisson *'palliative care in Sub Sahara in Africa'* Lancet; 2005: 365:1971-1977

20. Singer PA, Bowman KW (2002) *'Quality care at the end of life'* BMJ 324: 1291-1292.

21. Shneidman. E (1973) *'The death of man'* New York Quadrangle/ The New York.Sliep Y (1997) *'Care Counseling for community Based Counselors'* Lilongwe: Action aid- Malawi.

22. Stedeford A (1994) *'facing death: patients, families and professionals'* (2nd edn) Sobell Publications, Oxford.

23. D. J. Weatherall, J. G. G. Ledingham And D. A. Warrell (1987) *Oxford Textbook Of Medicine,* Vol 1, 2nd Ed. Pg. 12.146

24. WHO (1996) cancer pain relief. Geneva

25. Management of HIV - Related Diseases Guidelines Ministry of Health, Second Edition 2008.

26. iii Ministry of Health ARV guidelines, Malawi. (Version 3) 2008

27. [iv]Cape Town Declaration 2002

28. .Pezzatini M, Marino G, Conte S, Catracchia V.Oncology: A forgotten territory in Africa. Annals of Oncology 2007 18(12): 2046-2047. Available from http://annonc.oxfordjournals.org/cgi/content/full/18/12/2046 (cited 2008, July 22)

29. http://cancer.iaea.org/documents/May2007_London_Declaration_on_Cancer_Control_in_Africa.pdf (cited 2008, August 8)

30. [1] Larue F, Fontaine A,Colleau S. Underestimation and undertreatment of pain in HIV disease: multicentre study. British Medical Journal 1997. 314:23. Available from : http://www.bmj.com/cgi/content/full/314/7073/23 (cited 2008, July 22)++++++
