# Supplementary material for: Assessing and Responding to Palliative Care Needs in Rural Sub-Saharan Africa: Results from a Model Intervention and Situation Analysis in Malawi
Source: PLoS One. 2014 Oct 14;9(10):e110457. doi: 10.1371/journal.pone.0110457 (PMC4197005; doi:10.1371/journal.pone.0110457)
Supplement: Appendix S4 — Introduction to Palliative Care: Health Care Workers’ Service Providers Manual. (PDF) [file pone.0110457.s004.pdf]

GOVERNMENT OF MALAWI

MINISTRY OF HEALTH

Introduction to Palliative Care

Health Care Workers' Service Providers Manual

March 2011

NATIONAL AIDS COMMISSION

PALLIATIVE CARE ASSOCIATION OF MALAWI

INTRODUCTION TO PALLIATIVE CARE

HEALTH CARE WORKERS' SERVIVE PROVIDERS MANUAL

MARCH 2011

Publication of the Ministry of Health enjoys copyright protection in accordance with the provisions of protocol 2 of the universal Copyright Convention. All rights reserved.

The Ministry of Health welcomes requests from persons or institutions wishing to reproduce or translate its publications, in part or in full. Applications and equires should be addressed to the Secretary for Heath, P.O. Box 30377, Lilongwe 3, who will provide the latest information on any changes made to the text, plans for new editions, and reprints and translation already available.

Ministry of Health 2011

The mention of certain manufacturers' products does not imply that they are endorsed or recommended by the Ministry of Health in preference to others of a similar nature that are not mentioned

Acknowledgement

Ministry of Health in collaboration with Palliative Care Association of Malawi would like to thank all individuals and their organizations who contributed towards the development of the Introduction to palliative care for health care workers training manual.

Sincere appreciation goes to Hospice Africa UK, Scottish Borders Africa AIDS group and National AIDS Commission for the financial assistance rendered for developing this manual.

List of contributors:

Sheila Bandazi: Director of Nursing Services, Ministry of Health
Evelyn Chitsa-Banda: Zone supervisor, Central West Zone
Lameck Thambo: National Coordinator, Palliative Care Association of Malawi
Annie Chinguwo: Former Home Based Care Coordinator, Save the Children, US
Immaculate Kambiya: Chief Community Nursing Officer, Ministry of Health
Mercy Pindani: Lecturer, Kamuzu College of Nursing, University of Malawi
Bernadette Mzunga: Tutor, St. Luke's College of Nursing, Malosa
Catherine Chiwaula: Principal Community Health Nursing Officer, Ministry of Health
Loveness Nyirenda: Principal Nursing Officer, Queen Elizabeth Central Hospital
Masika Kamphale: Former Palliative Care Coordinator, St. Josephs Hospital, Nguludi
Kondwani Banda: Palliative Care Coordinator, Queen Elizabeth Central Hospital
Dr Jane Bates: Palliative Care Physician, Queen Elizabeth Central Hospital
Thadeo Macosano: Former Palliative Care Coordinator, St Lukes Hospital
Suave Gombwa: Former Palliative Care Coordinator, Mulanje Mission Hospital
Emily Chiona: Palliative Care Provider, Ekwendeni Mission Hospital.
Anna Anderson: Palliative Care Specialist, Catholic Health Commission
Kondwani Mkandawire: Assistant Registrar, Medical Council of Malawi
Martha Mondiwa: Registrar, Nurses and Midwives Council of Malawi
Margaret Young: Palliative Care Nurse, St. Luke's Mission Hospital, Malosa
Bessie Phiri: Clinical Superintendent, Ministry of Health
Rose Kiwanuka: National Coordinator, Palliative Care Association of Uganda
Jessica Mackriell: Formal Program manager, Palliative Care Association of Malawi

Acronyms

AIDS	Acquired Immunodeficiency Syndrome
ARV	Anti-Retroviral
ART	Anti-Retroviral Therapy
APCA	African Palliative Care Association
BBP	Benzyl Benzoate Paint
CBO	Community-based organisation
CHBC	Community Home Based Care
CO	Clinical Officer
EN	Enrolled Nurse
GoM	Government of Malawi
HSA	Health Surveillance Assistant
HIV	Human Immunodeficiency Virus
MA	Medical Assistant
MOH	Ministry of Health
NGO	Non-governmental Organisation
NMT	Nurse Midwife Technician
ORS	Oral Rehydration Salts
PACAM	Palliative Care Association of Malawi
PTB	Pulmonary Tuberculosis
QECH	Queen Elizabeth Central Hospital
SRN	State Registered Nurse
TB	Tuberculosis
HTC	HIV Testing and Counselling
WHO	World Health Organisation

Table of Contents and Appendices

Page

Foreword ....
.............i
Acknowledgements..
............ii
Acronyms ....
.............iii
Table of
Contents..i
v
Introduction of the
course 1
Session 1. Introduction to Palliative
Care...2
Session 2. When to Start Palliative
Care...5
Session 3. The Holistic
Approach 10
Session 4. Models of
Care...13
Session 5. Introduction to
Communication 17
Session 6.
Teamwork..18

Session 7. Ethics in Palliative
Care...26
Session 8. Common Symptoms in Palliative
Care...32
Session 9. Principles of Pain Management in Palliative
Care...50
Session 10. Use of Morphine in Palliative
Care 59
Session 11. Management of Skin
problems 63
Session 12. Common diseases in Palliative
Care 69
Session 13. Anti-Retroviral Therapy in palliative
care 76
Session 14. Children's
Needs 79
Session 15. Spiritual
Support 83
Session 16. Breaking Bad
news 86
Session 17. Wills and
Inheritance 93
Session 18. Holistic Patient Assessments
(practical)...96
Session 19. Death and
Dying 97
Session 20. Grief and
Bereavement 101

Session 21. Care for
Carers 108
Session 22. Implementing Palliative Care in
Malawi 112
Annex 1. 5-day
timetable 116
Annex 2. Palliative Care Assessment
form 117
Annex 3. Palliative care service provider's supervisory
checklist..118
Annex 4. Palliative care institutional supervisory
checklist 120
Annex 5. Referral Form for palliative
care...122
Annex 6. Sample Will
Format 123
Annex 7. WHO clinical staging and HIV disease in adults and
children...............................129
Reference ....
............137

Introduction

The Manual

In order to ensure provision of quality palliative care services to the Malawi Population, Government, through the Ministry of Health in collaboration with Palliative Care Association of Malawi developed this Palliative Care Service Providers manual to provide guidance for palliative care service provision. Palliative care providers in the health sector are therefore encouraged to use this manual as a reference when providing palliative care services to improve quality of life of patients and their families. The health care workers shall be doctors, clinical officers, nurses and medical assistants.

Course aim

The five-day course is designed to equip health care workers with knowledge, skills and attitudes in the provision of palliative care services.

Course Objectives

By the end of the course, health workers should be able to:

- Demonstrate an understanding of the need to implement palliative care services

- Take holistic history from a palliative care patient
- Describe the management of common symptoms in palliative care patients
- Explain the management of pain using the who analgesic ladder
- State the role of ARVs in palliative care
- Provide end-of-life care
- Implement palliative care services in their own settings

SESSION 1: Introduction to Palliative Care

AIM: To enable health care workers to acquire knowledge of the concepts underlying palliative care

TIME ALLOCATION: 1 Hour

LEARNING OBJECTIVES

By the end of this session participants should be able to:

- Define palliative care according to WHO
- Explain the historical roots of palliative care at international, regional and national levels
- Describe principles of palliative care
- Explain the need for palliative care in Malawi

1. Definition of Palliative care

Palliative Care improves the quality of life of patients and their families facing the problems associated with life threatening illness, through the prevention and relief of pain and suffering by means of early identification and impeccable assessment and treatment of pain and other problems – physical, psychological and spiritual (The World Health Organization, 2002)

The word 'palliate' means: to care, to relieve, to comfort, and to ease without curing.

Palliative Care;

- Is holistic.
- Is a team effort
- Affirms life and regards death as a normal process
- Neither hastens nor postpones death
- Provides relief from pain and other distressing symptoms
- Integrates the psychological and spiritual aspects of care
- Offers a support system to help patients live as actively as possible until death
- Offers a support system to help the family during the patient's illness and in their own bereavement
- Uses a team approach to address the complex needs of patients and their families

- Works to enhance the quality of life
- May also positively influence the course of illness
- Works to treat and prevent pain and suffering.

2. The Historical Background of Palliative Care

International

- Palliative care dates back to the second half of the fourth century when Fabiola opened a hospice for pilgrims and the sick. Hospice was used for the dying. Then Mornarchs followed suit and then hospice became hospitals.
- In 1842 the first hospice was opened for the dying by Jean Garnier who lost two young children in France
- In 1897and 1905 the Irish sisters of charity started hospice for the dying at Dublin and st. Joseph hospice in London
- In 1957 Cicely Saunders established St. Christopher Hospice in London. Research and training in this hospice led to the development of hospices and home care teams throughout UK, Europe, US and Canada
- In 1967, Cicely Saunders established modern hospice movement currently practiced worldwide

Regional

- The first hospice in Sub Saharan Africa was commenced in Harare Zimbabwe in 1979, South Africa in 1980, Nairobi in 1990 and Uganda in 1993. as a role model hospice for African palliative care services

National

- The first Palliative care team was established in Malawi in 2002 in the Pediatrics Department at Queen Elizabeth Central Hospital. This was followed by establishment of palliative care sites at several institutions in CHAM and public hospitals.
- In 2005 Palliative care association of Malawi (PACAM) was established.

3. Principles of Palliative Care

The following principle should be observed for effective provision of palliative care services;

- Attention to detail: Time spent here can bring great relief to the patient

- Honesty and respect: This should be shown at all times and the truth given when asked for, in terms understandable by the patient. False reassurance helps no one.
- A holistic approach: Concern for the emotional, spiritual and social aspects of the patient's care as well as controlling their physical symptoms
- A patient centered approach: To deal with what the patient thinks is the most important in all aspects of his/her care
- A problem oriented approach: Taking time to deal with each problem in turn, no matter how small

4. The Need for Palliative Care in Malawi

Malawi, like other countries in Sub-Saharan Africa, is still faced with the enormous burden of the HIVAIDS pandemic as well as cancer. There are more than a million people living with AIDS, and about 25 thousand living with cancer and countless others living with other diseases for which there are no curative treatments available at this time. It is estimated that about 80% of cancer patients will have pain in the terminal phase of their disease, and that 25% of HIVAIDS patients suffer severe pain during their illness. These figures do not address the suffering caused by other symptoms and psychological and spiritual distress. How ever, these official statistics are probably an underestimate of the real problem.

Palliative care also addresses some of the following challenges:

- Unmet physical needs; including the need for relief of pain and other symptoms
- The need for food
- The high cost, or unavailability of, appropriate analgesic drugs, such as morphine
- The severe financial constraints on the family and caregivers
- The need to train family caregivers
- Lack of psychosocial support
- Social isolation due to stigma, age, blaming, or other factors
- Isolation of many villages, lack of transport and distance from health care facilities
- The care of orphans and vulnerable infants and children

The WHO states that, palliative care service in Sub-Saharan Africa should offer:

- Pain and symptom control measures
- Food support
- Family support
- Income generating activities
- Carer specific training

Session 2: When to Start Palliative Care

AIM: To enable health healthcare workers to know when to start providing palliative care

TIME ALLOCATION: 1 Hour

LEARNING OBJECTIVES

By the end of this session participants should be able to:

- Explain the current situation of cancer and HIV / AIDS in Malawi
- Describe the graphic presentation of Cancer and HIV/ADS journey
- Identify when to start providing palliative care

1. Scope of the Problem: Current Burden of HIV / AIDS and Cancer in Malawi

1a) Burden of HIV and AIDS

HIV/AIDS prevalence rates are increasing, According to Malawi Demographic and Health Survey of 2002, the HIV prevalence was 12% among persons aged 15 to 49 years and the Sentinel Surveillance Survey of 2005, estimated HIV prevalence at 14% among the 15 to 49 years age group. Life expectancy has fallen from 46 years in 1987 to 37 years in 2005, largely due to the HIV and AIDS epidemic

The Southern region of Malawi is the most densely populated and has the highest prevalence rates, followed by the Northern and Central regions. Prevalence is significantly higher in urban areas (17%) than in rural areas (11%) (Malawi Demographic and Health Survey, 2004). There is evidence that while infection rates are slowing in urban areas, HIV prevalence continues to increase in rural areas where 85% of the population live. The primary mode of HIV transmission remains through unprotected heterosexual sex (90%) (Malawi Demographic and Health Survey, 2004)

An estimated 80,000 AIDS-related deaths occur every year resulting in drastic increase of orphan hood. Malawi was estimated to have 501,963 orphans as a direct result of AIDS deaths, with slightly over a million orphans and vulnerable children in total. (2005 estimate). One-third of all households hosted at least one orphan and that 30% of households cared for at least one chronically ill person (C - Safe Malawi Baseline Survey, 2003).

High HIV infection levels have also resulted in an unprecedented increase in tuberculosis cases at a co-infection rate of 70%. The disease burden is exacerbated by endemic malaria affecting up to 4 million people annually, mostly women and children (Malawi Round 5 Final GFATM).

1b. Burden of Cancer

Globally: there were 11 million new cases of cancer by 2002, In Africa there are 0.5 million cancer deaths per year. The incidence of cancer is set to increase by 400% in next 50 years (WHO, 2002)In Malawi, the incidence and prevalence data on cancer in Malawi is lacking due to lack of comprehensive data collection mechanisms. However, Malawi, like other countries in Sub-Saharan Africa, is tackling a looming epidemic of cancer It is estimated that about 25 thousand Malawians live with cancer and there are countless others with other diseases for which there are no curative treatments available at this time. The majority of patients who need palliative care live in rural areas, often far away from their nearest health facility. In the public sector, oncology services are based at Queen Elizabeth and Kamuzu Central Hospitals, focusing on the treatment of paediatric cancers. There is No or limited surgery & chemotherapy Interventions, Most patients/clients come late or therapy is unavailable. It is estimated that only 5% of cancer patients in developing countries access treatment. As such there is no radiotherapy service available in the country and currently few patients are being managed through referral to neighboring countries such as Tanzania and South Africa.

Management of patients with cancer from the time of diagnosis requires a palliative care approach with optimal pain and symptom control. Morphine (sustained release tablets) and other essential drugs for palliative care are intermittently available, and research has shown that some health professionals have continued fears about prescribing opiates (Bates 2008).

Graphs below gives an overview of the problem of cancer in Malawi

Total New Registration of Cancer cases per year in Malawi

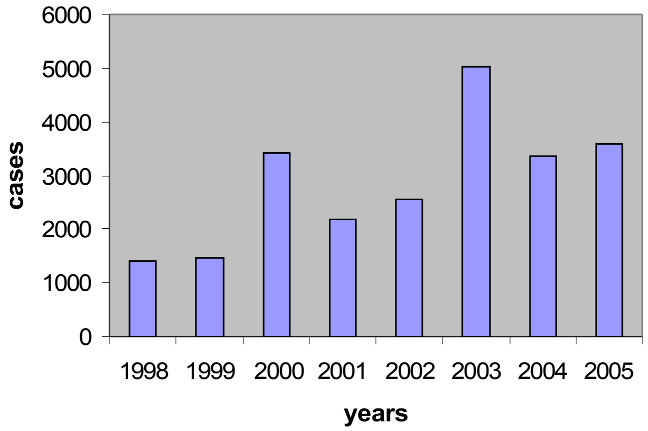

Bar graph 1: shows Cancer Epidemiology in Malawi with focus on Kaposi's sarcoma, cervical cancer and Burkitt's lymphoma (ministry of health, Malawi cancer registry; 2004-2005)

Common cancers in the total Population of Malawi

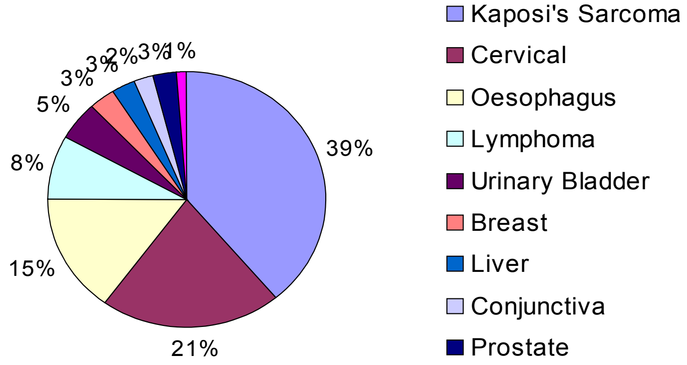

]

] **health, Malawi cancer**

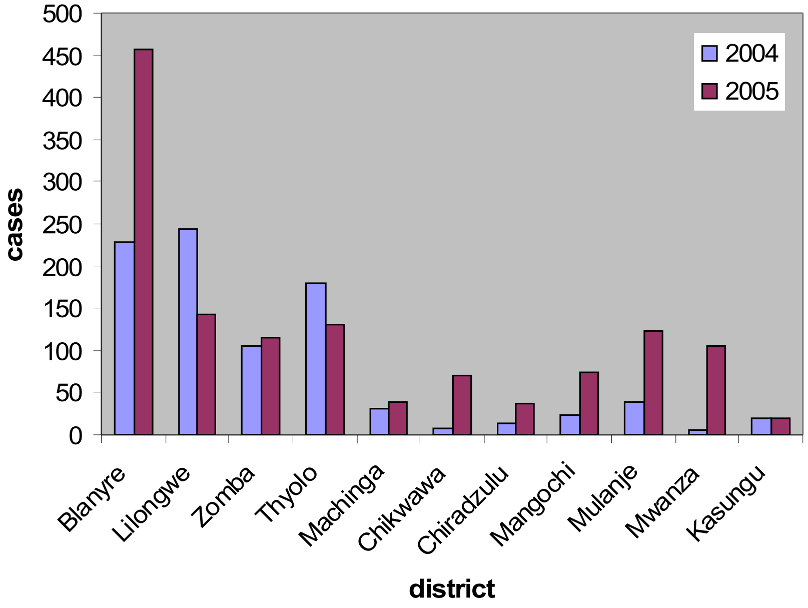

2. The Cancer -HIV / AIDS Journey

Graphic representation below shows the cancer journey versus the HIV/AIDS journey and Implications for intervention and approach using palliative care.

Figure 1: Cancer journey

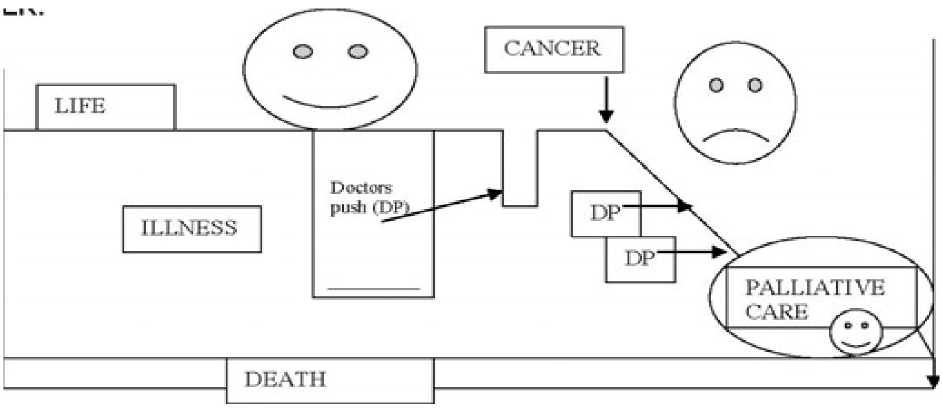

- The picture shows that the cancer journey from the time of diagnosis when the patient has good life but start experiencing problems, then she progresses to illness until the patient dies
- During the journey, when the patient experiences problems (physical, psychological, social, spiritual) and the doctor provides care to push the patient back to life known as Doctors push (DP), or to improve quality of life
- Cancer cure is rare, palliative care can be introduced to cancer patients at any stage of illness and palliative care is needed more towards the end of life. Palliative care helps to relief patients pain and distressing symptoms to improve quality of life.

Figure 2: HIV / AIDS journey

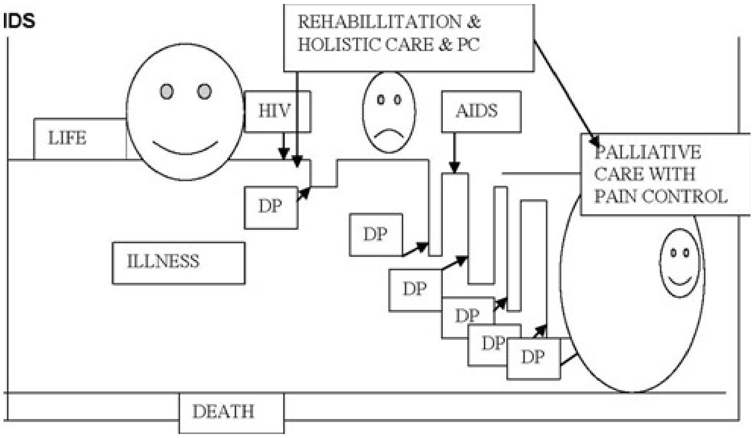

The graphic presentation illustrates doctor's interventions in improving the quality of life of patients towards the HIV/AIDS journey. NB DP stands for Doctor Push like cancer journey, HIV journey starts from the time of diagnosis when the patient has good life , progresses to illness then death. But with good doctors push quality of life of patient can be improved and become productive again for many years. During the journey, when the patient experiences problems (physical (opportunistic infections), psychological, social, spiritual) and the doctor provides care to push the patient back to life through providing palliative care, treatment and support.

Note: AIDS Patients often present with opportunistic infections or may be terminal. Aim of treatment is to modify the disease, to recover from opportunistic Infections and regain normal life and perhaps live longer

Figure 3: When to start providing Palliative Care

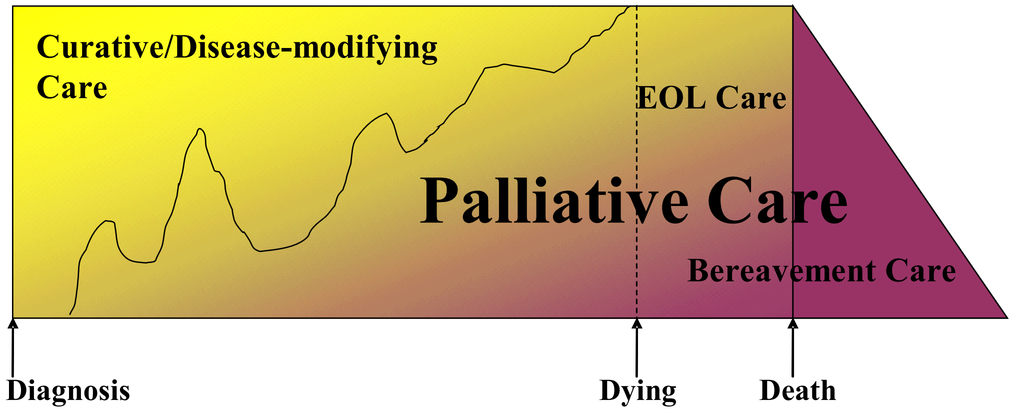

The above graphic presentations illustrates that;

- Palliative care starts right from diagnosing a life threatening illness.
- Curative treatment /disease modifying treatment such as chemotherapy and ARV s are part of palliation.
- Palliative care is not just End of Life (EOL) care
- Palliative care goes beyond death to grief and bereavement care.

Session 3: The Holistic Approach

AIM: To enable health care workers to acquire knowledge, skills and attitudes in the holistic approach

TIME ALLOCATION: 1 Hour

LEARNING OBJECTIVES

By the end of this session participants should be able to:

- Define the holistic approach in palliative care
- Describe the principles of the holistic approach
- Explain the components of holistic care
- Demonstrate holistic history taking using a case study

1. Definition of Holistic approach to Palliative care

Holistic care is the care of a whole person; incorporating physical, psychological, social and spiritual aspects. **Merriman et al (2002)**

Holistic care involves much more than drugs and physical care. It is emphasized by the WHO definition of health which is 'the wellbeing of a person physically, mentally, socially and spiritually not only the absence of disease'. Holistic care means caring for a patient as a whole being, in totality not only physical aspect.

Holistic care understands the patient in context of his environment made of:

- His family
- His social group
- His employment
- The whole social system

2. Principles of the Holistic Approach

- Your relationship with the patient must be other centered, giving precedence to the patient's needs

- Accept the patient and be committed to his or her welfare, give care and respect him/her
- Be objective in order to understand the patient without personal bias
- Recognize the patient's right to make his own decisions and the value of encouraging his/her independence
- Recognize the inter-dependence of the patient and others and realize that there may be times when patient's self direction needs to be limited to protect others or himself from harm
- Understands patient's needs and responds to them individually
- Uses a multidisciplinary team to achieve total care for the patient and family

3. Components of holistic care

a. Physical Care

- Includes physical assessment and examination normally done by Health Professionals
- Management of pain and other distressing symptoms
- Regular reviews

b. Psychological Care

- This focuses on relief of psychological pain-
- This relieves psychological pain, which could be due to hostilities, fear and anxieties from all angles
- Good communication from the caring team is very important

c. Social Care

This incorporates discussions on family and personal issues e.g. young children who will be orphaned, other financial issues e.g. loss of employment medical care, fees, food and nourishment and social status

d. Spiritual Care

- Having Known that the prognosis is poor, many patients start preparing for the next world
- Unresolved matters with God cause a lot of spiritual unrest
- The team must be prepared to listen, discuss and perhaps pray with the patient & emphasize God's love and understanding

4. Holistic History Taking

Focusing on the components of holistic care take a holistic history using the case study below:

'Case study: 30 years old man recently was diagnosed with EPTB He is married with 3 children a six year old boy and 2 year old twin girls. His wife left last year for study in South Africa. His complaints are: abdominal pains, numbness of the legs making

it difficult to walk, skin itch, & general body weakness. Take a history from this patient and identify his problems."

Session 4: Models of Care

AIM: To enable health care workers to acquire knowledge of various models of care

TIME ALLOCATION: 1 Hour

LEARNING OBJECTIVES
By the end of this session participants should be able to:
- Mention various models of palliative care service delivery
- Compare and contrast different models of care
- Identify an appropriate model of care for their own setting

1. Models of Patient Care
1. In-Patient Care
2. Home – Based Care
3. Hospital Palliative Care
4. Palliative Day Care
5. Outreach Clinic
6. Road-Side Clinic

2. Different Models of care

Model 1: In-patient Care
This takes place in a separate building; it can be a ward in a general hospital that admits palliative care patients. It is also referred to as a free standing hospice. It is ideal for patients who can not be looked after at home for medical or social reasons. The ideal unit has less than 25 beds. Patients are encouraged to bring in personal belongings such as furniture, etc.
Specialists in palliative medicine with the holistic approach, doctors, nurses, social workers, rehabilitation staff, dieticians, clergy and all other therapists sees the patient.

Some units look after children only. Some specifically care for patients with AIDS or cancer only.

Advantages

- Excellent standard treatment because experienced staff are 24hrs available.
- Drugs are readily available.
- A good place for training other staff palliative care, as it gathers patients and experienced staff in one place.
- Patients easily benefits from other therapists, for example: physiotherapists, etc.
- Allows care where there are social problems at home or in the community.

Disadvantages

- Expensive.
- Patients have fears of death, thinking that if they go they would die.
- Unit needs large number of qualified staff.

Model 2: Home Based palliative care

This is the commonest form of care in most African countries (Merriman et al, 2002). Care is provided at home mainly by the family, close friends and relatives. The patient is in the familiar environment, a place with memories, love and happiness.

Health workers often come as a home visit, once or more times per week, to help with supportive measures, counseling and pain & symptom control. On each visit the palliative care team assesses the patient and provides care holistically. If hospital admission is indicated the team recommends that the patient be admitted to a hospital for a close follow up.

Volunteers play an important role in the provision of day to day care and linking the patient with health workers if need arises.

Advantages

- Less costly for the patient and family; no waste of money for traveling.
- Guardians have time to do other household chores.
- Allows patient to use community support.
- Allows patient and family to use cheap available resources.
- Facilitates networking between health facility and community.
- Palliative services are available even to the poorest.
- Allows research in the community, for example: researches for useful local herb
- African countries have been using them for ages.

Disadvantages

- Nursing interventions may be inadequate, for example: daily wound care, etc.
- Poor resources, for example: counseling from experts, etc.

- Lack of privacy, space.
- Fears from family members, for example: fear of death in the house, etc.

Model 3: Hospital Palliative Care Team

The hospital has a palliative care team: at least one doctor and nurse with palliative care experience, and can access to other hospital services. The team receives referrals from consultants of patients with cancer or AIDS from different wards/OPD and from trained community volunteers in the villages who have identified patients with pain or palliative needs. Therefore patients with palliative care needs are seen in the wards where there are other general patients.

This model has an end-of-life care room that patients in terminal phase of life are managed there.

The team works hand in hand with a community nurse and is prepared to go out to visit difficult cases in the homes. Upon discharge from the hospital a follow up plan is made with the patient, hence the patient may be referred for care to other models of care.

Model 4: Palliative Day Care

Patients come for one or more days a week to a centre, which may be attached to another service (e.g. hospital) or on its own. They will often spend 5 – 6 hours at each visit. During this time patients are assessed and treatment given. Other therapies are given and patients take part in creative activities such as music, paintings, etc.

Patients may form strong friendships with other patients; which can be very therapeutic, preventing isolation and enabling patients to share thoughts and feelings with others who are experiencing similar problems. However when patients die others will be bereaved.

Volunteers are the key part of the model.

Day care services may vary in terms of criteria for acceptance of referrals and the emphasis of care.

Model 5: Outreach Clinic

This is where the mobile team has a monthly clinic at a health facility usually some distance from the parent organization. Patients and families who can travel meet the team there. Patients too sick to go to the health facility are visited at home by the local health professionals who follow them up.

Model 6: Road Side Clinics

En route to the outreach clinic, the team arranges to stop at a strategic place so that the local patients can be seen from the vehicle, or the team is met by relatives who take them to see the patient at home from the road side.

.

3. Local Models of Care in Malawi

Most facilities in Malawi are using a combination of Home based palliative care, hospital palliative care team, outpatient clinic and hospice .

Conclusion

Patients requiring palliative care services are encountered in a variety of settings. There is need for continuum of care from home to hospital and back again, working in partnership with other carers (e.g. by visiting the community) to ensure a high standard of pain and symptom control.

Session 5: Introduction to Communication Skills

AIM : To enable health care workers to acquire knowledge, skills and attitudes needed to communicate effectively with patients and their families who are receiving palliative care.

TIME ALLOCATION:1 Hour

LEARNING OBJECTIVES

By the end of this session participants should be able to:

- Define communication
- Explain the importance of communication in palliative care
- Recognize the importance of self awareness as a basis for Communication
- Demonstrate effective communication skills.
- Explain qualities and attitudes needed in communication.
- Describe barriers to communication.

1. Definition of Communication

Communication is a process of sharing ideas or information or thoughts ,feelings in order to come to a common understanding. Communication is a process by which people exchange information or express their thoughts, feelings and attitudes.

It is a two way process, which involves the transmitting and receiving of messages. The messages may be in the form of information, instruction, thoughts, feelings, signals or activities. For communication to be effective there is need to have a sender, message, channel, intended receiver and feedback.

Good communication with patients;

- Can make a difference in their lives and they can "start living again",
- Is an essential element in all areas of life and it is more important while dealing and working with sick people.
- Is greatly needed while handling a terminally ill patient and his/her family members/ carers.
- Enhance the quality of remaining life of a terminally ill patient and his/her family
- Is an essential part of human relationships. Human relationships play an essential role in working with people to solve problems and improve quality of life.

Poor communication on the other hand makes life difficult for a patient and his family/ carers. It creates a sense of rejection, confusion, misunderstanding and despair.

.

2. Importance of Communication

- Establish & maintain a relationship
- Promote equality in that relationship
- Gather information
- Provide information
- Facilitate self-expression
- Promote recovery
- Reassure the patient
- Manage & control symptoms

3. Importance of self awareness in communication

Communication starts with the provider. There is need for the provider to be more self-aware because;

- Once the provider is self aware, he/she becomes in charge of his/her lives and that can influence the way the provider communicate with others.
- The provider develops greater sensitivity to his/her own feelings and to those of others.

There is need to recognize or analyze how the provider sees her/him self and ask the following questions;

Intelligence

- Are you the kind of person who thinks that you are intelligent and can't talk with the less intelligent?
- Is it inherited intelligence or intelligence due to opportunities (being exposed opportunities) can you use your intelligence to help others or is your intelligence hindrance to effective communication.

Value system

Hierarchy of values: How do you perceive your values and how does it improve or affect communication?

Vulnerabilities

At one point or the other people are wounded in life. When they are reminded of those times they get frightened or angry, the client may feel the same. The provider should ask how he/she can avoid frightening or angering the patient during counseling. The provider should also ask herself how she/he feels about terminally ill patients. Does she/he feel comfortable with them? or avoid them? Before counseling it is important for the provider to review his/her feelings for the patient. Is the provider open or closed as she/he approach? Or distracted by her/his own worries Before counseling the provider need help and support from others and God, to put aside worries, fears, bias and open our hearts with a loving compassion.

3. Communication Skills

An effective communicator has the skills to use all types of communication that are appropriate for the needs of those involved. Communication is irreversible 'simply put when something has been said it cannot be taken back'.For communication to be effective both the sender and receiver should understand information.

Types of communication

Verbal: making use of spoken and written words
Non-verbal: use of body language e.g. facial expression, gestures, posture

Table : Communication skills

**Verbal 'CLEAR'**	**Remember Active Listening is important**
C Clarify<br>L Listening<br>E Encourage<br>A Acknowledge<br>R Reflect or repeat	Helps the patient feel accepted and attended to. Skills in Active Listening include;<br>• Paraphrasing<br>• Reflecting<br>• Clarifying<br>• Summarizing
**Non-verbal 'ROLES'**	**Remember the following issues**
R Relax<br>O Open<br>L Lean forward<br>E Eye contact<br>S Sit near the person	• Personal space<br>• Facial expression<br>• Eye contact<br>• Posture<br>• Gestures<br>• Touching

Effective questioning is also an important aspect of the communication process. The provider should be Ask the following type of questions depending on the type of information to be collected.

- Open ended questions
- Closed questions
- Leading questions
- Probing questions

Answering Questions

The responses we use can positively or negatively affect the way we communicate, and the way in which the patient or family members feel they are able to communicate with us.

Checking Of Understanding

It is important to check that we have understood what the person has said to us because:

- It gives an opportunity for persons to think again about how to cope with the problem.
- It also shows that we have been listening carefully.

4. Qualities and attitudes needed in communication with patients and families
Desire to help
In order to communicate well with our patients we should have an inner urge to help the patient and his/her family members.

Patience
When patients come to us as health workers they are in most cases weak and are not sure of what to say to us.
Make them take their time while expressing themselves. This calls for a high degree of patience although health professionals are always extremely busy.

Respect for others
Handle every patient as an individual. Respect individual beliefs, values and attitudes.

Genuineness
This involves being sincere and free from pretence while handling patients. Try to be honest to patients and their family members if you are to win their trust.

Confidentiality and Privacy
More often patients have issues which they do not share with other people but only with health professionals.
This calls for confidentiality with such information in order to sustain your helping relationship.
Ensure privacy while dealing with each patient and/or their families.

Knowledgeable
When giving Information to clients make sure it is accurate and clear.
This will enhance trust and future communication with the patient.

Ability to put others at ease
This involves creating rapport with the patient. Using empathy and a caring non-judgmental attitude patients and families can feel accepted and relaxed.

5. Barriers to effective communication

- Age
- Social and cultural factors (including language used)
- Physical appearance (either in the health care worker or patient)
- Physical environment (privacy, setting)
- Filtered listening
- Preconceived bias/prejudice
- Negative professional attitudes to palliative care

- lack of attention to detail
- Limited time

Session 6: Teamwork

AIM: To equip health care workers with knowledge, skills and attitudes required for effective multidisciplinary teamwork

TIME ALLOCATION: 1Hour

LEARNING OBJECTIVES

By the end of this session participants should be able to:

- Define a team
- Describe composition of a Multi-Disciplinary Team in palliative care
- Describe characteristics of an effective team
- Discuss challenges that may arise in a Multi-Disciplinary Team
- Discuss ways of managing conflict

1. Definition of a Team

A team is a group of individuals working together to achieve a common goal, a means to an end

2. Composition of a multidisciplinary team in palliative care

The team can comprise of a patient, close family members, doctor, clinical officer, medical assistant ,Nurse, Physiotherapist, Occupational therapist, Social worker, Chaplain, Priest/other religious leader ,Volunteers, Pharmacist, Dietician, Administrators

2. Characteristics of an effective team

- Recognizing the centrality of patient and family needs
- Mutual respect for specific personal contribution of each team member
- Competence of each team member in his/her own discipline
- Competent leadership appropriate to the structure and function of the team and the tasks at hand
- Mutual support
- Good communication
- Early referral

2.1 Some of the tasks of the team include:

- Diagnosis of progression or recurrence of disease – and appropriate communication of such information
- Formulation of treatment plans for the disease and/or symptoms, with due regard to ethical dimensions of the scenario
- Implementing, monitoring and formulation of new plans
- Recognition of the dying phase
- Comprehensive personal care, support, recognition of suffering, alleviation of distress, restructuring of hope
- Facilitating preparation for death
- Professional support of family/carers, and especially including any involved children

3. Challenges

a. Demoralization

This may result from or reinforce conflict. Demoralization in a team may be expressed in absenteeism, apathy, resistance to change, or deep sadness. A team may be wounded by poor leadership, or unreasonable burdens. Without a vision the people perish, and so does a team.

Re-examining the foundations may assist in rebuilding the team. The team leader may need assistance from an outside resource to assist in rebuilding the team.

b. Conflict

Is a situation that can occur within an individual, leading to inability to resolve divergent viewpoints, or involve at least two parties both with different viewpoints at can often be emotional. Conflicts may be inevitable among the team members.

Conflict may arise in a multidisciplinary team as a result of the following reasons;

- Differences in perception
- Differences in values and beliefs
- Un clear responsibilities
- In adequate communication
- Poor management style

Effects of unresolved conflicts

- Violent disposition/aggression
- Low self esteem
- Poor performance
- Resignation of team members
- Loss of interest to work
- Loss of commitment to achieve positive results
- Loss of punctuality and neglect of duties

4. Conflict management

Conflict should usually be resolved with minimal involvement of other persons. Where this is not possible the existence of the conflict needs to be brought to the notice of the team leader. Conflict between interfacing teams may jeopardizing patient care and need to be addressed. Fundamentally, the conflict needs to be admitted, the shape of it articulated, the difference in perception of fact or of judgment clarified at the same time as common ground is sought as a basis for consensus. Conflict resolution may involve the following approaches;

Avoiding

This involves avoiding conflict, to pretend that it does not exist and to withdraw. Personal goals are not important to the withdrawer. No effort is made to address the problem/conflict.

Self centered

The person who follows a self centered style of conflict management is very competitive and always puts her/his own interests and personal goals first. An egoistical approach is followed and the goal is always to win the case. This individual is not interested in harmony or positive relations in the organization. Other people's needs are not considered. A winning approach is followed by using an attacking, overpowering technique.

Delaying

This is a consoling approach, "Oh things are not so bad!" in this principle the concerned party becomes anxious in the face of the conflict and prefers to restore relations without necessarily addressing the problem. "After all it's not so bad; it can wait provided we maintain a working relationship" Conflict is thus avoided in order to restore harmony in the organization.

Negotiating

This is a way of solving the conflict by compromising. Both parties negotiate with each other to win something and at the same time to give away something. It is also called win-win/ lose-lose situation. Both parties should be prepared to sacrifice something, to meet each other half way so that both parties benefit equally. The actual root (cause) of the conflict is therefore never addressed.

Confronting

In this principle conflict is dealt with by way of the problem solving approach. Goal achievement as well as the restoration of relations in the organization is important to the

care provider. It is not necessarily a give and take situation; the problem is however addressed by confrontation so that the truth is exposed in order to find the most appropriate solution. Confrontation is therefore the best way of ensuring that problems are identified, analyzed and the best possible solution is sought for.

Session 7: Ethics in Palliative Care

AIM: To enable health care workers to acquire knowledge and attitudes on ethical principles relevant to Palliative care services

TIME ALLOCATION: 1 hour

LEARNING OBJECTIVES

By the end of this session participants should be able to:

- Define ethics
- Explain the importance of Ethics in Palliative care
- Describe the key principles of ethics
- Discuss how ethical principles can be applied in palliative care
- Discuss ethical dilemmas in palliative care

1. Definition

Ethics refers to collective belief and value system of any moral community, social or professional group. In medicine, Medical ethics is the study of rules, principles and values applied to the fields of medicine and health care.

Ethics describe what is good for the individual and society and what rules we need to prevent people from being harmed. It is concerned with right and wrong.

- Branch of philosophy
- Science of morals

2. Importance of Ethics

- Views about right and wrong vary

- Many ethical issues arise in palliative care—truth-telling, confidentiality, when to stop treatment etc
- Terminally ill patients are often vulnerable and require extensive care
- advancing medical technology means new ethical issues constantly arise

3. Principles of Medical Ethics

Much discussion about medical ethics is based on the four principles of Medical Ethics:

a. Respect for Autonomy- making voluntary decision

b. Beneficence – doing good

c. Non-Malificence- duty not to inflict harm in others

d. Justice – treating people equally

a. Respect for Autonomy

The principle of respect for a person's own choices based on his/her evaluation of what is best for his/her life. This involves (among other things):

- Respecting a patient's right to confidentiality.
- Telling the truth.
- Getting informed consent for procedures.
- Respect of patients' rights to have control over their own bodies and to make decisions about their medical treatment.

Respect for a patient means

- Recognizing they have their own feelings
- Helping the patient decide what is best for them
- Being courteous and respectful
- Maintaining confidentiality
- Protecting privacy
- Considering both what we are saying and the way we say it (links with communication skills)
- Avoid being judgmental
- Avoid being patronizing

Autonomy involves:

- The capacity to decide
- Understanding the implications of a decision
- Making a voluntary decision (not forced by doctor or family) (linked issues- Informed consent)

Limits to Autonomy

- Patients who have limited understanding, mental disability or severe mental illness
- An unconscious or severely ill patient
- Patients who have addictions
- Limited resources: not everyone can get what they want

- Social norms: society sets limits on what patients can get

b. Beneficence

Doing good; the principle of helping others by taking actions which promote the good of others. Related to:

Best interests: what makes a person's life go well. Most doctors, clinical officers and nurses will try to act in their patient's best interests

c. Non-maleficence:

This principle means a duty not to inflict harm on others.

e.g. If you know that a person may contract a sexually-transmitted disease by not being told by a partner, you have a duty to try to prevent this.

d. Justice

Fairness; the outcome in which everyone receives their due. People should be treated equally unless there are reasons for unequal treatment. e.g. If there is not enough to go round, which patients should get ARVs?

3. Ethical dilemmas in palliative care

Certain Ethical Conflicts may arise in Palliative care:

a. Patients refusal of treatment:

"a patient who is conscious and refuses further treatment must have his wishes respected whatever his condition, provided he is mature and lucid enough to make such a decision"

b. Withholding or withdrawing Active Treatment

We are morally and legally responsible for both our acts (withdrawing) and our omissions (withholding)

Considerations:

- Patient's wishes
- Patient's physical condition
- Financial issues
- Health- worker's autonomy

c. High tech medicine and resuscitation

When a patient is near death- a doctor is not obliged to embark upon or continue heroic treatment which has no prospect of benefiting the patient." This concept is known as 'medical futility'.

- Too painful
- Too difficult
- Too dangerous
- Kennedy 1984: elderly man, colonic cancer put on a respirator against his will
- "Death is not the enemy doctor: inhumanity is"

d. Pain Killing or Patient Killing?

"A doctor's obligation, when he can no longer hold back death, is to make the patient comfortable including easing his pain. If to make the patient comfortable the doctor must take measures which may hasten death, this is permissible providing the doctor's aim is only the relief of pain". Incorporates "double effect" theory in English Law (1957)

e. Decisions: by patient or relatives?

"The relative of patients have no right to make decisions unless the patient is incompetent"

Old age -Does not mean Incompetence

Lack of education - Does not mean Incompetence

g. Euthanasia

Means the deliberate killing of a human being to relieve their suffering. Or to relieve them of life in a body judged to be unable to function normally by others.

Types

1. "*Voluntary" Euthanasia*

Means that the person asks themselves to be put to death either at the time of the illness or have left a written request, while in a state of health, indicating that if they are terminally ill or suffer from an incurable illness, that they want to be helped to die.

"When someone asks for euthanasia – take note that someone, or society as a whole, has failed that person"

Dame Cecily Saunders, founder of the modern hospice movement

2. "Passive" Euthanasia

Means totally withdrawing food or curative treatments with the intention of letting the patient die. Withdrawing treatments which will make the patient uncomfortable, or are questionable in the dying patient is not passive euthanasia!

3. Euthanasia and assisted suicide:

"The doctor may not embark on any conduct with the primary intention of causing the patients death", "If a terminally ill patient expresses a desire to commit suicide a doctor may not in law facilitate suicide."

- Doctors need a relationship of trust with the patient and family, explaining all procedures in the light of the condition.

GUIDING PRINCIPLES IN ETHICAL DILEMNAS

'Thou shall not kill' **God s commandment on natural law**

"I will give no deadly medicine to anyone if asked, nor suggest any such counsel; and in like manner. I will not give a woman a peccary to produce an abortion" **Hippocratic Oath**

"I will maintain the utmost respect for human life from the time of conception; even under threat, I will not use my medical knowledge contrary to the laws of humanity"

Declaration of Geneva (amended Sydney 1967)

Doctors need to be able to elicit the fears of the dying and to discuss and answer those fears, so that patients can see that they will not be -abandoned or left helpless. Only when this becomes the norm can society expect to dissipate pressure to force doctors to do things that the medical profession should not accept.
Conclusions of BMA working party on Euthanasia 1988 (Sir Henry Yellowlees)

Ethics case study 1

Mr. DS is 40yrs old with advanced colon cancer . He is unmarried and has moved into his older sister's home as his general condition deteriorated. He understands that he has progressive cancer which has spread to his liver. He has had 3 admissions to the local hospital for symptom control when he developed acute breathlessness secondary to recurrent anaemia which he found very frightening. His breathlessness improved following blood transfusions at increasingly frequent intervals - 1st July, 23rd September, and 14th October. He presents again on 19th October with breathlessness on exertion,
He talks calmly about the fact that he will die soon, that he has wonderful support from his sister and her family and from his minister and friends in the congregation.
He says he is tired and does not want another transfusion. His sister is very distressed and asks if you can persuade him to accept a transfusion so that he can spend some quality time at home, "He is too young to die".

What are the ethical aspects to consider in deciding a current management plan for Mr. DS?
Think of: truth-telling, respect for the patient, confidentiality, family relationships

Ethics case study 2

James is 12 years old, HIV positive and has Kaposi's sarcoma. You have seen him over three months and he is losing weight and getting more breathless. His mother has said to you that he should not be told, and that everything should be tried for James. However, you see that he is more and more tired and in pain. He asks you in the clinic with his mother present: 'Am I ever going to get better?'
How do you deal with this? What do you say to James and his mother?
Think of: truth-telling, right to know, your relationship with James and his mother, communication skills and principle of palliative care

Session 8: Common Symptoms in Palliative Care

AIM: To equip health care workers with the knowledge and skills to recognize and manage common symptoms in palliative care patients

TIME ALLOCATION: 2 Hours and 40 Minutes

LEARNING OBJECTIVES
By the end of this session participants should be able to:
- State the common symptoms in palliative care patients
- Describe different causes of these symptoms
- Manage these symptoms holistically

1. General approach to symptom control in palliative care
- Assess cause and severity
- Treat reversible causes
- Initiate disease specific palliative care (drug and non-drug measures)
- Involve and explain the plan to patient and family
- Review

2. Causes and management of symptoms

1. Pain - Refer To Session 9 and 10

2. Insomnia

May consist of insufficient sleep, poor quality sleep or interrupted sleep.

Common Causes

- May result from chronic fatigue, poor concentration, irritability and depression
- uncontrolled pain
- Bladder or bowel discomfort or incontinence
- Diarrhea
- Nausea and vomiting
- Dyspnoea
- Fever / night sweats
- Fear of dying / anxiety
- Delirium
- Drug withdrawal – benzodiazepines, alcohol, nicotine
- Drugs – steroids, antidepressants
- Caffeine
- Environmental factors, e.g. noise

Management

- Identify cause and treat, if possible
- Provide pain and other symptom relief
- Review medication – ensure steroids are given early in the day
- Address causes of anxiety and fear – counseling, reassurance
- Provide a quiet and comfortable sleeping area – a light or lamp in a dark room may help reassure a frightened patient
- Discourage from drinking strong coffee or tea at night
- Encourage use of relaxation techniques – prayer, music
- Drugs – antidepressants, amitriptylline to treat nocturia, benzodiazepines or hypnotics for insomnia

3. Anxiety

An unpleasant emotion which may be acute (transient) or chronic (persistent) and varies in intensity.

Clinical Features

- Persistent and inability to relax
- Worry
- Poor concentration
- Insomnia / fatigue
- Irritability
- Inability to make decisions
- Sweating, tremor, nausea, anorexia
- Panic attack
- Palpitations
- Breathlessness
- Dry mouth

- Diarrhoea
- Frequency of maturation
- Headache
- Chest pains

Common Causes

- Uncontrolled pain or other symptoms
- Depression / psychosis
- Fear of illness, treatment or death
- Worry about family or financial issues
- Drug side-effects – steroids
- Drug withdrawal – opioids, benzodiazepines, alcohol

Management

- Identify cause and treat if possible relieve pain and other distressing symptoms
- Review / modify drug treatment
- Provide counseling, support, reassurance
- Drug treatment – anxiolytics, hypnotics

4. Depression

Depressed mood and sadness may be an appropriate response to approaching death for the terminally-ill patient. It is often under diagnosed and under-treated. It is important to identify depression as conventional treatment achieves a good response in the majority of patients. If untreated, depression can result in worsening of symptoms and in social withdrawal.

Clinical Features

- Depressed mood
- Loss of interest or pleasure in almost all activities
- Anorexia
- Marked weight loss or gain
- Insomnia / hypersomnia / early morning awakening
- Loss of self esteem
- Feelings of worthlessness / helplessness / guilt
- Poor concentration / indecision
- Thoughts of death or suicide

common causes

- Uncontrolled pain or other symptoms
- Loss of independence
- Lack of support from family / friends / healthcare workers
- Fear – of dying or death
- CNS tumours
- Drugs – steroids, diazepam, efavirenz

- Loss of body image, due to disfigurement

Management

- Relieve pain and other distressing symptoms
- Review / modify drug treatment
- Provide counseling, support, reassurance
- Drugs – antidepressants, e.g. amitriptylline (but warn the patient that these will not work instantly)

5. Delirium / Acute Confusion

This is the most common and serious neuropsychiatric complication in the patient with advanced illness. It leads to acute alteration in the levels of alertness, cognition and perception. It is often under diagnosed. Delirium often fluctuates and becomes worse at night. The onset may be sudden.

Clinical Features

- Restlessness, anxiety, irritability
- Sleep disturbances – drowsy by day, insomnia at night
- Disorientation to time, place, person
- Memory impairment
- Poor concentration
- Altered perception – delusions, hallucinations
- Rambling, incoherent speech
- Mood swings – euphoria, paranoia

Common causes

- uncontrolled pain or other symptoms
- CNS tumours
- Drugs – steroids, diazepam, efavirenz, promethazine etc
- Alcohol abuse and drug abuse
- Drug interactions and withdrawal
- Electrolyte imbalance
- Infection(CNS & sepsis)
- Metabolic causes – hepatic or renal

Management

- Identify cause and treat, if possible
- Provide company, support, reassurance
- Treat with respect
- Reassure family members that the patient is not insane
- Maintain a safe environment – in familiar surroundings, if possible
- Minimize the number of staff and visitors interacting with the patient
- Avoid physical restraint
- Modify drug treatment – stop drugs which may cause or exacerbate the problem
- Drugs (only if necessary) – e.g., haloperidol, chlorpromazine

6. Cough

Cough is most commonly seen in patients with lung cancer, tuberculosis and the pneumonias with AIDS.

Common Causes

- Bronchial obstruction from a primary tumour or mediastinal mass, most commonly enlarged mediastinal glands.
- Tuberculosis or pneumonia in immuno-suppressed patient.
- Secondary bronchial infection, tuberculosis, pneumonia or an abscess in a necrotic tumour.
- Left ventricular failure with characteristic dyspnoea and cough wakening the patient.
- Vocal cord paralysis due to hilar tumour or lymphadenopathy.

7. Productive Cough

Management

a) Postural drainage can aid expectoration and drainage if the condition of the patient can take it. Nurses must be experts in this. Rarely a physiotherapist is available but the family can be shown simple techniques within the range of comfort for the patient.

b) Antibiotics are often useful in clearing infection and facilitating easier expectoration. They should always be considered in the AIDS patient. Unscientific as it may appear they are occasionally useful even when no pathogens have been demonstrated in sputum cultures. (Remember culture and sensitivity may not always be accurate and sensitivities may be according to the pellets available from the manufacturing company. Avoid unless clinical knowledge not able to provide the diagnosis.

c) Cough syrup, may appear to be useful in spite of the paucity of scientific data to support their use, but only when used regularly for a minimum of four weeks. Keep prognosis in mind.

d) If bronchospasm is present a bronchodilator is helpful e.g. Ventolin (salbutamol) or cough linctus.

8. Dry cough

Common Causes

Tuberculosis, lung cancer, asthma, Kaposi's sarcoma, pleural effusion and CCF

Management

Is undoubtedly helped by:

a) Cough suppressants, for example: Codeine linctus (1mg/ml 10ml 4-hourly) or codeine tabs 30mgs 4 hourly. . Morphine solution (2.5mg 4-hourly). For cough suppression the dose of morphine used is the lowest needed to alleviate this symptom and always so small that depression of respiration is no problem.

b) Humidifying of the atmosphere with steam can be done in the home using a kettle or a basin of hot water breathed in under a towel.

c) Steroids (dexamethasone 2mg/day) may be used in cancer patients to reduce oedema surrounding a tumour, relieve bronchial oedema and lessen bronchospasm. They are also needed in lymphangitis and radiation pneumonitis, which also cause cough.

Limit to a short course if the patient is suspected of having AIDS and include an antifungal.

d) Local anaesthetic lozenges if available can be useful in laryngeal/tracheal irritation and patient should not eat or drink for an hour after taking.

Nursing techniques

a) If the cough is secondary to an uncomplicated bronchogenic carcinoma the patient is seldom comfortable propped upright in bed but better nursed with only two or three pillows. The key is the position most comfortable to the patient. Failure to do this may further embarrass respiration.

b) If due to chronic bronchitis and obstructive airways disease he should be propped up as straight as is comfortable.

c) In pneumonia the patient is usually more comfortable resting with two pillows, but always gives way to the position most comfortable for the patient.

d) If there is a pleural effusion (whether necessitating aspiration or not), he should lie on the side of the effusion in a semi-recumbent position. Pleural aspiration is often a painful procedure and the doctor must not only be prepared to prescribe appropriate analgesics but remember the problems of nursing a patient on the side of aspiration. A combination of frusemide and spironolactone useful in delaying the necessity for aspiration if there is an element of heart failure. Watch patient's hydration if combination of diuretics is commenced as there is a risk of dehydration. Do not persist if excessive dryness of mouth results.

Note: Among patients living with HIV/AIDS, cough persisting for more than 2-3 weeks should be considered suspicious for TB, and patient referred for investigation/treatment if available. In general, unless the underlying cause can be removed, there are few truly effective measures to relieve symptom entirely, though a

9. Dyspnoea

Dyspnoea is a subjective experience of difficult, labored, and uncomfortable breathing.

It is one of the most feared symptoms because all such patients anticipate death from suffocation and asphyxia.
No effort should be spared in managing this symptom and finding all means to relieve the patient's anxiety, which often borders on panic. Cause of breathlessness should be established and treated if possible particularly in patients with HIV/ in early stage disease. It must be recognised that very little can be done for the underlying pathology in obstructive airways disease, bronchial obstruction by tumour or lymphangitis carcinomatosis at the end-stages of disease. If patient is still conscious and aware of symptoms, low dose opioids and benzodiazepines, along with reassurance, can be very helpful in lessening distress. It is also vital to provide reassurance to the family members.

Common causes

- Caused by cancer and/or aids: Effusion atelectasis consolidation massive ascites replacement by cancer (KS) lymphangitis of functioning lung
- Related to treatment: post-radiation fibrosis post-pneumonectomy
- Related to debility: anaemia pneumonia pulmonary embolism
- Unrelated to cancer or treatment: chronic obstructive airway disease or asthma.

Management

- Attempt to modify the pathological process e.g. with anti-pyretics, cortico-steroids, (care in AIDS) radiation therapy, hormone therapy, chemotherapy, pleural aspiration when appropriate.
- Use such non drug measures as a calming presence, cool draught from an open window or fan, breathing exercises, relaxation therapy.
- Anxiolytics such as oral diazepam 2 -10mg each night for sustained effect, or triazolam 0.0625-0.125mgs (shorter acting benzodiazapine if available) to enable sleep during the most alarming time of the day.
- Equally effective in reducing sensation of breathlessness by a number of mechanisms, is low-dose morphine regularly, e.g. morphine solution 2.5 - 5mg every 4 hours or MST 10mg 12 hourly. If already using high dose morphine for pain, increase by 1/3 of total dose or in weaker patients, 2.5mg 4hrly increments until dyspnoea is controlled.
- Suppress paroxysmal cough exacerbating dyspnoea with morphine or codeine linctus and hot drinks.
- Panic attacks are best treated with oral or rectal diazepam (5 - 20mg). Oral or rectal routes act more quickly than I.V., because of the metabolism of diazepam. Rectal diazepam preparations are very expensive. However diazepam for injection can be

inserted into the rectum using a syringe (minus the needle!) and is equally as effective and usually more available.

Oxygen is necessary only for the same indications as at any other time in medical practice; contrary to what some patients and many relatives may feel, it has been not shown to be effective in treating dyspnoea/breathlessness in non-hypoxic patients.

Oxygen is often not available in the African situation and fanning with the local newspaper often gives just as much relief. The doctor has a responsibility to explain carefully that oxygen will not prolong the life of the patient with lung cancer, nor resuscitate a dying patient. **No medication replaces this reassurance.**

Specific causes of breathlessness

10. Pleural Effusion

Though not strictly a symptom this complication is common enough to pose a problem to the clinician. The rule is that it should be aspirated only:
(a) For diagnostic purposes
(b) If, by so doing, incapacitating dyspnoea will be relieved.

Where the effusion is likely to recur and the life expectancy of the patient is still likely to be long, it is worth considering the instillation into the pleural space of an agent producing pleurodesis. E.g. Bleomycin or tetracycline powder. Tapping of effusions has been shown to increase the rate of fluid accumulation and the tapping needs to be repeated at shorter and shorter intervals. Some patients respond to frusemide 40 mgs with spironolactone 50– 100mgs daily, and it is worth trying to delay the inevitability of aspiration.. It is worth remembering that pleural effusion secondary to a bronchogenic carcinoma or adenocarcinoma of the lung carries a poor prognosis. On the other hand, patients with breast carcinoma complicated by pleural effusion may still survive many months.

11. Superior Vena Cava Obstruction

Superior vena cava obstruction merits attention because of the extreme distress it can produce and its response to therapy.

Clinical features include:

- Breathlessness
- Neck and facial swelling
- Trunk and arm swelling
- A sensation of choking
- Headache
- Neck vein distension

- Plethora

Occasionally it may present as one of the emergencies of terminal care.

Management

- Steroids: e.g. dexamethasone 24mg stat iv or p.o. 1st day. 12mg 2nd day .Maintenance: 4-8mg per day in divided doses. or predinisolone 25 mgs qid x 5/7. If good response reduce if not stop
- Consider prompt radiotherapy, or cytotoxic chemotherapy depending on the tumour histology. Remember that a patient on radiotherapy may have this complication due to oedematous reaction around the tumour. or predinisolone 25 mgs qid x 5/7. If good response reduce if not stop

12. Haemoptysis

Catastrophic haemoptysis is alarming to both patient and family. The families need to be prepared if this is a possibility so that they do not start rushing to hospital in the terminal stages. This may occur as an early or initial event in the lung cancer journey or as a terminal event.

Common causes

It may a occur in TB, KS, pneumonia and heart failure

Management

Hospitalization may also be indicated if blood transfusion is available and still appropriate

If patient able to swallow give double usual dose of morphine +/- diazepam. This sedates the patient and relieves the panic. Calming presence of carer / relative who has been prepared and told not to appear to panic in front of the patient and can express this to visiting relatives. Keep free of blood using receptacle and changing materials soiled with blood. Dark coloured cottons should be used in preference to white or pastel shades when the colour red alarms the patient.

13. Stridor

This is an unusual complication experience but can be most distressing to the patient and the family.

Common causes

Laryngeal cancer, Thyroid cancer, Oralphryngeal and KS

Management

Morphine orally and benzodiazepine e.g. diazepam can relax the patient.
Explanation needs to be given to the patient and family.
High dose steroid can help if it is due to tumour compression..

14. Hiccup

This is a relatively common symptom in advanced disease which causes distress to the patient

Common Causes	Management
■ Irritation of the phrenic nerve by tumour involvement at the hilum of the lung.<br>■ Direct irritation of the diaphragm (infection, tumour).<br>■ Uraemia.<br>■ Dyspepsia (especially with hiatus hernia).<br>■ Elevation of diaphragm (from enlargement of the liver or ascites).<br>■ CNS tumour.	Immediate:<br>■ Pharyngeal stimulation e.g. by swallowing dry bread or two teaspoons of sugar.<br>■ Correct uraemia if possible.<br>■ Simple re-breathing from a paper bag to elevate pCO2 level.<br>■ Drugs:<br>(i) Haloperidol 0.5mg bd orally or 1.5mg intramuscularly during attacks.<br>(ii) Chlorpromazine 12.5- 25mg bd. or prn during attacks (although its sedative effect may distress the patient).<br>(iii) Metoclopramide 10mg qid or domperidone 10-20mg qid may be quite effective if due to gastric distension.<br>(iv) Muscle relaxants e.g. baclofen 5-10mgs TDS are effective for some patients

15. 'Death Rattle'

Noisy breathing due to the accumulation of secretions in the large airways, in the patient whose conscious level is falling prior to death, is often more distressing to those around than to the patient..

Management

- This should be explained, particularly to relatives
- Positioning and regular change of position to ensure that secretions drain from the mouth and to prevent pooling of secretions.
- Hyoscine butyl bromide (Buscopan) 10-20mgs subcutaneously helps reduce the production of secretions. But must be given early and regularly to be effective as it does not clear secretions already there.

- Morphine subcutaneously may be given along with the hyoscine, if the patient is too ill to swallow if the patient is still semi-conscious to relieve breathlessness.
- Suction should be reserved for the unconscious patient.

16. Halitosis

Halitosis – an unpleasant or bad breath. It occurs when exhaled air is combined with foul-smelling substances coming from various sections of the respiratory tract or the upper digestive tract.

The patient may be unaware of the condition, but it can cause distress and embarrassment to relatives and friends.

Common causes

Diseases of the Oral Cavity

60-85% of all cases of halitosis are a result of diseases of the oral cavity. Therefore, careful history and examination is important to exclude these causes.

- Poor dental and oral hygiene
- Oral thrush
- Dry mouth – loss of saliva, mouth breathing, dehydration
- Gingivitis (gum infection)

Diseases of the Respiratory Tract

sepsis in the mouth, pharynx, nose, nasal sinuses, lungs
tonsillar disease, lung abscess/cancer and necrotic ulcers

Diseases of the digestive tract

Gastric stasis, due to delayed gastric emptying or gastric carcinoma (‘cesspool halitosis’)

Metabolic Failure

A reduced intake of food can cause acidosis and ketosis, giving an acetone odor
Diabetic ketoacedosis. Uraemia – ammonia smell

Drugs: Causing dry mouth, e.g. antidepressants, cytotoxic drugs, may damage oral mucosa and smoking

Foods

Garlic, onions, alcohol. These foods contain certain products which are absorbed by the intestinal wall and then excreted through the lungs or saliva.
Alcohol also causes a reduced flow of saliva

Management

General Measures

Good oral hygiene

- The patient may be unable to do this for himself,
- Cleaning with soft tooth brush
- Rinse the mouth with salty water or diluted lemon juice
- Encourage oral fluids

chew or suck small pieces of fruit(pine apple or orange)
mouth wash and paste- crushed flagyl + sobo+crushed steroids (predinisolone) to reduce odour and pain

17. Dysphagia

This means difficulty in swallowing.

Clinical features

It is important to differentiate between true dysphagia and painful swallowing. Usually the patient can identify the site of the obstruction.

- Obstruction at the back of the mouth – food stays in the mouth and there may be drooling of saliva
- Pharyngeal dysphagia – food sticks in the throat and causes coughing, gagging, regurgitation
- Oesophageal disorders – the patient complains that food sticks behind the sternum
- Pain + dysphagia – may be due to inflammation or infection, or to tumour infiltration

Common causes

- Mechanical obstruction. Dysphagia is the presenting symptom in most pharyngeal and oesophageal cancers, but may also be a symptom of other cancers which have spread.
- Debility – due to extreme weakness and cachexia (marked weight loss and muscle wasting)
- Obstructive lesions cause dysphagia for solids initially and liquids in the later stages.
- Neuromuscular disorders cause dysphagia for both solids and liquids at the same time.
- Dry mouth
- Oesophageal candidiasis
- Pain
- Ulcers
- KS
- Neuromuscular causes – encephalopahy,
- Radiotherapy and chemotherapy

Management

Depends on the site of the obstruction and the stage of the disease

- Dietary advice and modification – soft, moist foods, small, frequent meals, eaten slowly. The effort involved in eating may be very tiring
- Upright position – to facilitate swallowing
- Analgesia
- Antifungal preparations, for candidiasis
- Steroids, to reduce inflammation or oedema. If no effect by the third day, stop treatment. Always give antifungals with steroid therapy
- Nasogastric tube – not recommended for long-term use due to patient discomfort
- Endo-oesophageal tube – used to maintain a passage for food and fluids through a narrowed portion of the oesophagus or gastro-oesophageal junction. It is inserted surgically or by gastroscopy. May be useful if the patient is relatively independent and active. Contraindicated in moribund patients
- Gastrostomy tube – generally contraindicated in advanced cancer.

18. Anorexia

This is the loss of appetite associated with advanced disease but also with chronic hunger. It may be accompanied by nausea, and usually results in weight loss.

Common Causes

- Cancer
- Opportunistic infections
- Pain
- Weakness / fatigue
- Treatment side-effects – antibiotics, chemotherapy
- Gastrointestinal problems – sore mouth, dysphagia, constipation, malabsorption, nausea and vomiting, tumours, infection, delayed gastric emptying, squashed stomach syndrome
- Anaemia
- Changes in taste sensation – common in cancer and HIV
- Psychological causes – depression, anxiety, fear
- Disease process – anorexia increases as disease progresses
- Unappetizing food, poorly

Management

- Explain to the patient and family that anorexia is normal as disease progresses
- Identify and treat underlying causes, if possible
- Encourage small, frequent meals of the type of food which the patient likes
- Present food attractively
- Cook food away from where the patient is, to reduce cooking smells
- Don't force feed
- Encourage the patient to eat with other family members – appetite is usually better when meals are eaten in company
- Encourage the patient to sit in an upright position when eating
- Encourage gentle exercise, to promote appetite
- Appetite stimulants – eg. Prednisolone 10mg tds, before meals; small amounts of alcohol

presented

- Strong food smells during cooking – may cause nausea
- Drugs, e.g. antiemetics to reduce nausea, Metoclopramide to speed up gastric emptying.
- Generally contraindicated in advanced cancer.

19. Diarrhoea

Chronic diarrhoea – the passage of unformed stools 3 or more times in 24 hrs continuously (or episodically) for more than 1 month. It may be accompanied by abdominal cramps, flatulence, haemorrhoids, fissures, rectal bleeding and dehydration

AIDS related diarrhoea is common and may produce up to 20 stools a day. Often associated with vomiting and faecal incontinence. May cause severe dehydration. Diarrhoea in an AIDS patient may be due to an opportunistic infection and therefore not fatal – treatment of the cause plus adequate rehydration may lead to recovery and a return to normal lifestyle.

Very distressing for patients – unable to continue with activities of daily living, uncomfortable to travel from home; exhaustion due to efforts of repeated trips to the toilet; embarrassment of faecal incontinence.

Common causes

In 60% of AIDS patients, no cause is identified

- Constipation- most common cause in terminally ill cancer patients. Constipation with faecal impaction leads to overflow diarrhoea
- Misuse of laxatives - erratic use of laxatives, eg. waiting until the patient is constipated and then prescribing high doses of laxatives
- Infection -Cryptosporridium is the most common pathogen found in HIV positive patients with diarrhoea in Africa
- Pre-existing disease - ulcerative colitis
- Partial bowel obstruction
- Drugs – recent use of broad spectrum antibiotics, iron
- Malabsorption
- Malignancy

Management

- Correct dehydration – ORS, oral fluids, IV fluids. IV fluids are not usually indicated in terminally-ill patients but may be of benefit where the condition is remediable, eg. in the AIDS patient with an opportunistic infection
- Exclude constipation / faecal impaction
- Review / modify current drug treatment
- Review / modify diet
- Drug treatment – antibiotics may be used if the cause of diarrhoea is known
- Anti-diarrhoeal drugs, eg. Loperamide, Codeine, Morphine, Lomotil

- Local remedies - give 1 teaspoon crushed pawpaw seeds bd, juice made from boiled mango, avocado or guava leaves
- Careful attention to skin care – keep clean and dry, especially if the patient is incontinent

20. Constipation

Infrequent or difficult defaecation, usually associated with small, hard stools

Clinical Features – vary from infrequent bowel movements to complete cessation with signs of faecal impaction

- Abnormally hard stools
- Overflow diarrhoea – due to faecal impaction
- Anorexia
- Nausea and vomiting
- Urinary retention
- Flatulence
- Abdominal cramps and 'bloating'
- Restlessness / confusion

Common causes

usually a combination of causes

- Poor nutrition – diminished fluid and fibre intake
- Immobility and weakness – unable to walk to the toilet to defaecate
- Lack of privacy / embarrassment
- Pain
- Haemorrhoids
- Metabolic disorders – dehydration due to vomiting or fever
- Drugs – opioids, iron, antidepressants

Management

General measures

- Stop / change drug treatment
- Encourage mobilization
- Ensure easy access to toilet facilities

Diet – increase fluid and fibre intake

Drugs – laxatives, e.g. Bisacodyl or Senna tablets. Natural remedies, e.g. 1 teaspoon crushed pawpaw seeds bd

Manual evacuation – may be necessary with severe faecal impaction

21. Nausea and Vomiting

Nausea – an unpleasant subjective sensation involving the upper GI tract and usually associated with a feeling of the need to vomit.

Vomiting – the expulsion of the gastric contents through the mouth

Clinical Features – often multifactorial

common causes

Sepsis

Broad-spectrum antibiotics

Delayed gastric emptying

Oral candidiasis

Drugs

Management

- Identify cause and treat e.g. Steroids for raised ICP, Antacids for regurgitation, Laxatives for constipation
- Review drug treatment and stop unnecessary drugs
- antiemetics – Promethazine, Metoclopramide 10mgs bd – given 30 minutes before eating – can speed gastric emptying
- Dietary advice – small, frequent meals, avoid fatty or spicy foods
- Avoid aggravating smells
- Topical antibiotics for infected lesions, eg. a paste of crushed Flagyl and steroids can reduce both odour and pain, but be careful as the steroid component may cause bleeding
- Mouthwash, made from crushed Flagyl tablets mixed with fruit juice
- Antifungal preparations, eg. Nystatin, Ketaconazole, Floconazole, Miconazole
- Discontinue the use of drugs causing the symptoms

22. Sore Mouth

A sore mouth is common in patients with HIV/AIDS or cancer, leading to difficulties in eating, drinking and swallowing.

Clinical Features – vary according to the cause

- Pain
- Dry, smooth tongue / coated tongue
- Alteration in taste sensation
- Angular stomatitis
- Dysphagia
- Halitosis
- Small, white plaques – candidiasis
- Associated anorexia and weight loss

Common causes

- Poor oral hygiene
- Poor nutrition
- Xerostomia (dry mouth) due to reduced saliva production
- Oral candidiasis

- Infection – infected KS lesions, aphthous ulcers, bacterial stomatitis
- Drug side-effects – antibiotics predispose to fungal infection, chemotherapy

mouth-breathing

Common Causes Management

- Identify cause and treat, if possible, e.g. antifungal drug for candidiasis
- Encourage nutritious diet
- Oral hygiene – clean mouth and teeth regularly, use of mouthwashes after meals (can be made by adding a pinch of salt to a glass of warm water), apply Vaseline to the lips to keep them moist
- Analgesia
- Stimulate flow of saliva – by sucking sweets or small pieces of fruit
- Give soft, moist food which is easy to swallow
- Encourage oral fluids, to keep the mouth moist
- Topical preparations – a paste made from crushed Metronidazole and Prednisolone tablets can be applied to ulcers to reduce pain and odour

23. Squashed Stomach Syndrome

This is a reduction in stomach capacity leading to a feeling of fullness after taking only a small amount of food.

Clinical Features

- Early satiety / constant feeling of fullness
- Oesophageal regurgitation
- Indigestion

Common causes

Liver enlargement, gross ascites, pelvic tumours, e.g. ovarian, bowel

Management

- Small frequent meals
- Antacids – to remove gas, thus increasing stomach capacity
- Antiemetics, e.g. Metoclopramide 10mg tds, taken 30 mins before meals, to control nausea and speed gastric emptying
- Steroids – to reduce tumour size

'Case study 1: A 23 year old man on treatment for 3 months, has problems with chest pain , poor appetite & oral sores . He is seperated from wife & children and has started vomiting every thing. How would you assess this man & suggest appropriate treatment.'

'Case study 2: You visit a 30 year old man at home who had surgery of rectum last year. He has lost weight, complains of the pain & smelly discharge from the anus and has lost interest in eating. How would you assess this patient & what management would you suggest'

Session 9: Pain Management in Palliative Care

AIM:To equip health care workers with knowledge skills and attitudes on the management of pain in palliative care

TIME ALLOCATION: 1 Hour

LEARNING OBJECTIVES

By the end of this session participants should be able to:

- Define pain
- Explain the types of pain
- Describe the different classes of pain
- Demonstrate how to take a pain history
- Describe tools used to quantify pain
- Discuss principles of pain control
- Explain holistic pain management using the WHO analgesic ladder

Introduction

Unacceptably high proportion of patients are living and dying with uncontrolled pain. Patients usually experience progressive pain as their disease advances.

In AIDS severe pain is more commonly related to the presence of opportunistic infections, or side effects of ART. The progression of pain is harder to predict, so pain medications need to be titrated up and down, and adjusted even more frequently

1. **Definition of pain**

Pain is an unpleasant sensory and emotional experience associated with actual or potential tissue damage (Twycross, 1995).

Pain is;

- What the patient says it hurts……….What the patient says it is …………………
- A subjective experience/personal.
- What maybe experienced by one person as terrible pain may be brushed aside by another sensation
- Pain begins with a physical stimulus but it is always modified by the mind
- A mother kissing away the pain of her child, and the doctor prescribing a pain killer, are both involved in treating pain

2. Types of Pain

When treating a person's pain all the factors should be taken into consideration.

Types of pain	Some examples of pain
Physical	1) Cancer: bone, nerve compression, nerve infiltration, soft tissue infiltration, visceral, muscle spasm, fungating tumours, lymphoedema, treatment-related<br>2) HIV / AIDS: Opportunistic Infections – headache due to Cryptococcal Meningitis; KS; musculo-skeletal pain; neuropathic pain; vulvar/mouth/ oesophageal candidiasis/ulcerations; herpes zoster; skin rashes<br>3) Side effects of ARVs: peripheral neuropathy, skin rashes, abdominal pain<br>4) Other: pressure sores, arthritis, angina, trauma, muscle pain, **wasting**
Psychological	5) Fear, grief, anxiety, depression, anger, humiliation
Social	Loneliness, isolation, rejection, troubled relationships, stigma, losses, family conflict and stress, worries, reduced income, loss of work or ability to work, unable to provide for family or self, hunger
Spiritual	Guilt, beliefs and faith may be questioned, fear, punishment, search for meaning (why?), hopelessness<br>Some cultural beliefs, secrecy, rejection, stigma, taboo, isolation

Total Pain

The experience of pain of life threatening illness can be a cycle that is physical, psychological, social and spiritual. In palliative care we refer to this complicated and holistic experience as 'Total Pain'.

Factors Influencing perception of Pain

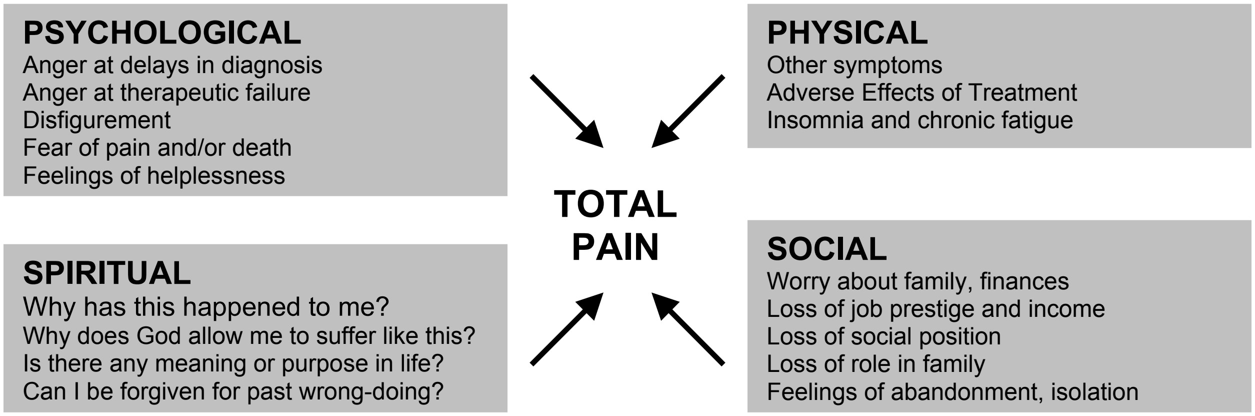

3. Classification of Pain

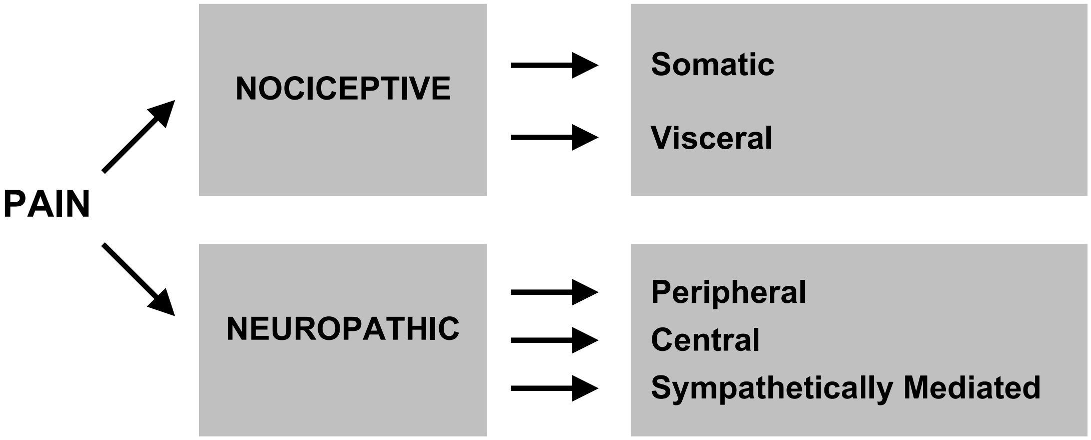

Pain can be either: acute (surgical/trauma/fracture/acute infection/recurrent)
or chronic arthritis/cancer/HIV/AIDS

Nociceptive pain

- Caused by damage to non-neural tissue
- Stimulates nociceptive nerve endings
- Impulse follows known nerve pathways to brain
- Usually sensitive to opioids –

Neuropathic pain

- Caused by compression or damage to nerve tissue
- Neural dysfunction results in spontaneous impulses
- These do not follow known pathways
- Complex disorganized blasts of impulses
- Reduced responsiveness to opioids

4.Taking a pain history

- History of each pain and description in patient's own words
- Effect on sleep, movement or any other function
- Map out patient's pain(s) on the body chart
- Careful evaluation considering the cause of pain.
- Diagnose each pain as Nociceptive or Neuropathic

Case study

You are with a 45 year old women with advanced breast cancer in Karonga district hospital. Her husband works in Blantyre. She has 5 children, 3 of whom live with her, one works in Mzuzu, the other is overseas. She has a lot of pain at the site of the breast cancer which is not helped by regular paracetamol. She also complains of a burning pain which travels down her right arm.

1. What classes of pains does she have?

2. For holistic assessment what other aspects of history do you need to complete?

3. What treatment can you offer her?

5. Pain assessment tools

The A B C D E Mnemonic for pain assessment

Ask about pain regularly. Assess pain systematically.
Believe the patient and family in their reports of pain and what relieves it.
Choose pain control options appropriate for the patient, family and setting.
Deliver interventions in a timely, logical, and coordinated fashion.
Empower patients and their families. Enable them to control their course to the greatest extent possible

Body Chart

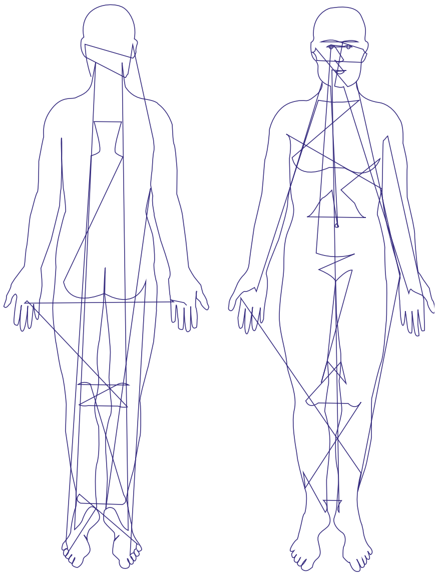

Pain Rating Scale

These are used to help patients describe the severity of their pain, and changes in pain over time. The health worker needs to explain the scales and assist the patient to score their pain.

Simple Descriptive Pain Intensity Scale

No pain mild pain moderate pain severe pain very severe pain worst possible pain

Numeric Pain Intensity Scale

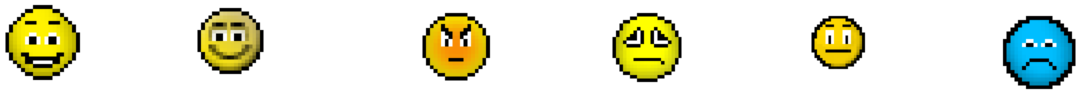

0 1 2 3 4 5 6 7 8 9 10

No pain mild pain moderate pain severe pain very severe pain

Numerical Pain Rating Scale

I Do not have any pain 0______1_____2______3______4______5 My pain could not be worse

Show on your fingers how severe is pain… 5 is most severe.

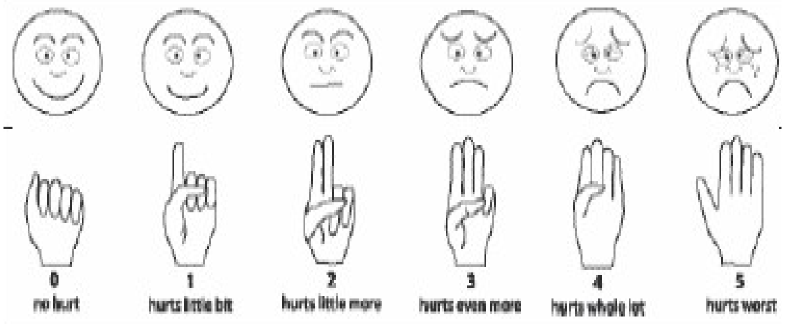

6.0 Pain Control

The World Health Organisation (WHO) claims that freedom from cancer pain and pain caused by other terminal diseases like HIV/AIDS should be a basic Human Right.

Good Pain Control Requires:

- Accurate and detailed assessment of each pain.
- Knowledge of different types of pain.
- A different therapeutic approach to chronic pain.
- Knowledge of which treatment modalities to use.
- Knowledge of the actions, side effects and pharmacology of analgesics.
- Assessment and treatment of other aspects of suffering that may aggravate pain.

The aim is to allow patients with terminal diseases to be pain free or pain to be sufficiently controlled that does not interfere with their ability to function or detract from quality of life.

Therefore, an approach was designed that was simple and easy to use anywhere in the world, even on a tight drug budget known as 'WHO Analgesic Ladder'. This approach was put forward to treat chronic pain caused by cancer. It has also shown to be beneficial in treating pain syndromes in HIV/AIDS (Merriman 2002).

6.1 WHO Analgesic Ladder

It is a stepwise approach to the treatment of pain which enables one to move from non-opioids analgesics to strong opioids in 3 small, simple steps.

This approach advocates that the severity of the pain determines which drug or drug category to select.

It works best for pain that is opioid responsive (nociceptive, visceral and somatic pain) as opposed to neuropathic pain which responds partially to opioids. However, it is worth to try opioids before giving adjuvant drugs as there is evidence that some patients with neuropathic pain may respond.

The basic principle underlying this step – wise approach is that analgesia must be tailored to suit the patient. This includes even adjuvant or secondary drugs.

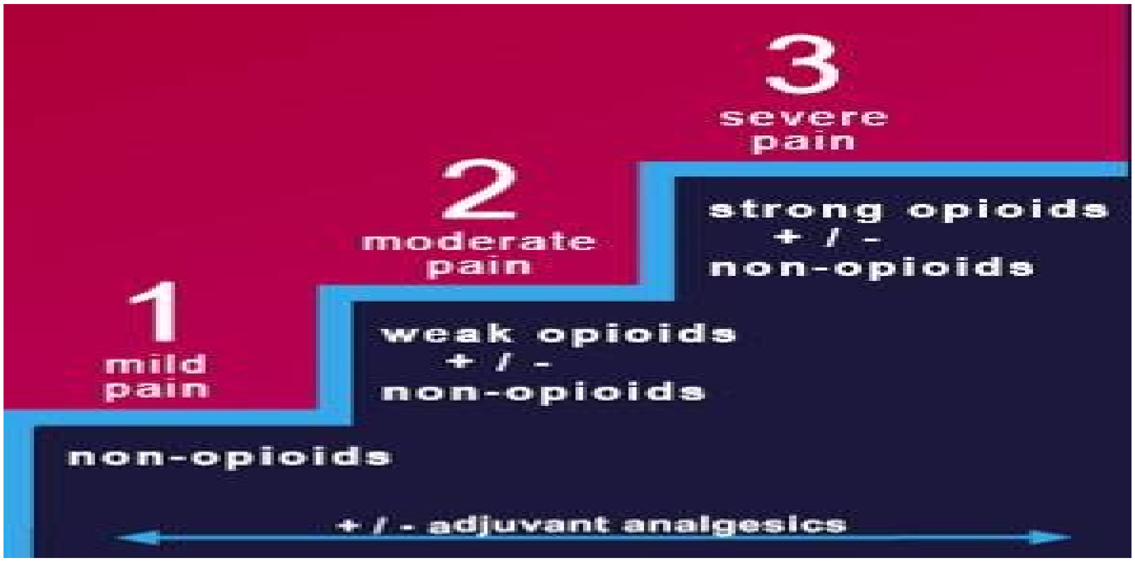

6.1.1 Non – opioids

- Main ones are NSAIDS and paracetamol.
- Anti – inflammatory effects – good for metastatic bone and soft tissue pains.
- Choice – several factors – availability, cost, frequency of admission, toxicity etc.
- All have analgesic ceiling effects Examples: ibuprofen, indomethacitin,diclofenac etc.
- Effective for bone and inflammatory pain.
- Causes gastric irritation and ulceration, therefore administer with food and milk.

6.1.2 Weak Opioids:

- Proto type- codeine phosphate.
- Examples: codeine, dihydrocodeine, Tramadol etc.
- Step 2 analgesics can be combined with step 1 but not with step 3.
- However they are expensive and have an analgesic ceiling effect.
- Laxative a must unless patient has diarrhoea.

6.1.3 Strong Opioids.

- Main active constituent of opium e.g. Morphine
- May be given together with step 1 analgesics.
- Always add laxative except in AIDS related diarrhea.

Note: Pethedine is not recommended due to its short duration of action and its side effects in an effective analgesic dose. This is due to accumulation of the metabolites norpethedine which can cause CNS excitation and convulsions.

6.1.4Adjuvant Analgesics

These are a miscellaneous group of drugs which relieve pain in specific circumstances. Generally given in addition with analgesics from the ladder.

i. Corticosteroids

These can be helpful for pain due to nerve root/nerve root compression which is caused by a tumor compressing the nerve. A combination of a steroid and opioid is usually effective.

E.g. Dexamethasone 4-8mgs daily then reduce by 1mg daily depending on symptoms.

Oral candidiasis and ankle oedema are common adverse effects of steroids therefore give antifungal alongside steroids in cancer and AIDS patients.

ii. Anti-Depressants and Anti-Convulsants

Helpful for neuropathic pain, which may present as burning, pricking, allodynia, paraesthesia or sharp, shooting .e.g. Amitryptilline 25mgs at night. Dose can be increased slowly up to 75mgs. Phenytoin: 100mgs bd, may increase slowly to 100mgs tds.

iii. Antispasmodics

Helpful in relieving visceral distension pain and colic.e.g. Hyoscine Butylbromide orally or subcutaneous.

iv. Muscle Relaxants

Helpful in painful muscle spasms (cramps) and myofascial pain. Diazepam 2.5mgs TDS may be tried.

6.2Principles of Analgesic Use

By the clock: Regular analgesia is necessary as patients require prevention therapy towards their persistent pain. When given regularly, the plasma concentration of the drug will be maintained thereby enabling effective pin control. PRN medication with chronic pain does not work.

By the mouth: Oral medication is the standard preferred in these patients.

By the ladder: Three step analgesic ladders should be used when prescribing analgesics. Treatment should move up as the pain increases and down the ladder as the pain improves.

By the patient: The dosage is determined on an individual basis. Some patients may need far higher doses than others depending on their pain and perception**.**

6.3.Non Pharmacological Pain Management

Palliative care includes many non-pharmacological ways to manage 'total' pain. These therapies address the physical, psychological, social and spiritual dimensions of pain. The following are a few holistic non pharmacological ways of treating pain.

Anything that enhances quality of life can, in turn, relieve pain.

- Listening and empathy
- Counseling – provides emotional support and practical suggestions
- Companionship and accompaniment – can help ease pain and increase comfort
- Activities such as favourite music, games, gardening, memory book – provides meaning and distraction
- Spiritual/pastoral support and prayer – provides comfort, meaning and hope
- Positioning – enhances comfort and relieves pressure areas
- Bathing, grooming, and other care measures – enhances comfort and self respect
- Exercise – improves mobility, circulation and skin integrity
- Massage, therapeutic touch
- Traditional therapies that are beneficial, healing and comforting
- Heat/cool applied locally – can reduce swelling and help relaxation
- Treatments such as radiotherapy can reduce inflammation, pain and tumour size

Note: Palliative care recognizes the great value of the complementary use of natural and herbal remedies in the treatment of distressing symptoms including pain.

Session 10: Use of Morphine in Palliative Care

AIM: To equip health care workers with knowledge, skills and attitudes in the use of morphine in palliative care

TIME ALLOCATION: 1 Hour and 30 minutes

LEARNING OBJECTIVES

By the end of this session participants should be able to:

- Outline background of morphine use at national and international level
- Describe different preparations of morphine
- Describe the side-effects of morphine
- Discuss fears and myths surrounding use of morphine
- Discuss importance of morphine in palliative care.

- Debate legislation on morphine use in Malawi
- Explain the administration of oral morphine

1. Background

- Morphine is a "gold standard'in palliative care
- The famous 17th Century English Physician Sydenham said, "Among the most powerful remedies that God bestowed to mankind in order to ease our suffering, none is as efficient or as universal as opium".

2. Preparations of Morphine and Prescription

a. Oral Morphine

Analgesic of choice in severe pain in cancer and HIV/AIDS. Available in the following preparations:

i. **Liquid Preparations**

Prepared in strength of 5mgs per 5ml and 50mgs per 5ml.It is made from powder form of morphine mixed with water. The solution may be coloured for identification and preserved with standard preservatives e.g. parabens/bronopol. Liquid morphine is cheap and accessible.

ii. Morphine Slow-Release Tablets (MST)

MST is a slow release tablet of morphine, the effect lasts for 12hours.

They are prepared in 10mgs, 30mgs and 60mgs. However they are expensive and therefore not accessible to many patients. Starting dose is 10mgs 12hourly, though care should be taken in elderly and those with liver or renal impairment where it is preferable to commence on liquid morphine to ascertain the correct dose .i.e. add up daily total requirement of liquid morphine then divide by 2 to give a twice daily dose.

b. Morphine Injection

Available in 10mg and 15mg ampoules. Best used in subcutaneous delivery systems (syringe pumps)

Points to Note

From analgesic ladder: discontinue all step 2 analgesics when starting patient on step 3.

Morphine does not have a ceiling effect and can be titrated against pain if it breaks through to achieve maximum control of pain.

3. Side effects of Morphine

a. Constipation

This is real problem and should be anticipated in all cases except where there is severe AIDS related diarrhea. Always prescribe a laxative if patient started on morphine.

b. Initial drowsiness:

The patient should be warned of this and it usually resolves in 2days. If persists up to 3days consider dose is too high and need to reduce.

c. Initial nausea and vomiting:
This has not been found in African patients and routine use of an anti- emetic is not recommended. However, if needed prochloperazine 5mg.tds or haloperidol 1.5-5mgs Od for 5days can be given.

d. Urinary Retention
Occurs in some patients due to the anticholinergic effects. It usually resolves with an in and out catheter, until normal tone is renewed in the bladder wall.

e. Morphine Twitches
This is a sign of over dosage. Most troublesome when patients' organs are failing before death. Therefore, reduce the dose by 50% initially and every 24 hours until twitching stops.

4. Fears and myths surrounding the use of morphine
i. Tolerance
It is not a clinical problem. Increasing dosage of morphine slowly is the accepted method in order to achieve pain control in advancing disease. The upper limit dose of morphine is the dose that controls pain.

ii. Physical dependence.
It does occur with chronic use of opiate as with a number of drugs like in asthma cases e.g. steroids. Therefore abrupt discontinuation of an opiate should be avoided as this causes withdrawal symptoms such as twitching and confusion, withdraw gradually.

iii. Psychological Dependence i.e. Addiction:
Addicts are looking for a high, which cannot be obtained when morphine is taken by mouth. This is only obtained with iv administration. Pain is now known to be a physiological antagonist to addiction.

iv. Cognitive impairment
When morphine therapy is initiated there may be some sedation and temporary attention deficit, manifested by reduced recent memory. These generally disappear within 2-3 days. Patients on morphine should be alert and feel normal, able to eat, sleep and attend normal affairs

v. Lethality
Morphine does not kill if properly prescribed and gradually increased according to need. In fact patients live longer because they can eat, sleep and live normal lives

vi. Respiratory Depression
Pain acts as a respiratory stimulus which is greater than the respiratory depressive effect of morphine.

Also had also been shown that morphine can be used to relieve dyspnoea (Walshe et al 1981)

5. Importance of morphine in palliative care

WHO Expert committee on Essential drugs (1995) defines Essential drugs as those that satisfy the health care needs of the majority of the population; they should therefore be available at all times, in adequate amounts and appropriate dose forms. Freedom from pain should be seen as a right of every patient and access to pain therapy as a measure of respect for this Right. (WHO Expert committee -1990). But Class A/ restricted drug. (CD)
have proven to provide pain relief up to 90% of cancer patients (Ripmonti and Dickerson 2001) But not available to majority of cancer and HIV/AIDS patients in Africa.

Morphine is an essential drug recommended by WHO in the treatment of both chronic and acute pain. Oral Morphine is the analgesic of choice in severe cancer and AIDS pain. No patient should live with chronic pain or reach end of life in pain. However, despite recent advances in the methods of pain relief, too many patients experience unnecessary pain. Relief of pain lessens the distress caused to individuals, families and communities.

6. Legislation on morphine use

- morphine is a Controlled drug (CD)
- prescribed by authorized clinicians – doctors
- should only be used in hospitals

Task
Discuss the current use of morphine in Malawi
Do we need to review the legislation?

7. Morphine administration

Starting dose with 2.5mgs – 5mgs 4hourly and may be increased slowly, if patient still experiencing pain, increase the dose by 30-50% of the previous dose. Give double dose at night to allow the patient to sleep all through the night.
Children; dose based according to body weight.

Session 11: Management of Skin Problems

AIM: To enable health care workers acquire knowledge and skills in the management of skin problems

TIME ALLOCATION: 1 hour

LEARNING OBJECTIVES

By the end of this session participants should be able to:

- Describe the principles of wound management
- Identify the clinical features of each of the following skin condition pruritus, pressure sores, fungating tumours, fistulae, herpes zoster, drug reaction
- Describe the factors predisposing to the development of pressure sores
- Discuss the measures appropriate to the prevention of pressure sores
- Discuss the management of each condition
- Discuss the management of each condition

Introduction

Skin problems are almost inevitable in palliative care patients. The general condition, and particularly the nutritional status, is reflected in the skin. Problems in advanced disease are often due to skin that is weakened by the general effects of chronic disease, eg. Malnutrition, dehydration and immobility.

1. Wound Care

Management of wounds in palliative care can be complicated because of the advanced nature of the underlying disease or condition. Treatment needs to be appropriate to the stage of the patient's disease. The principles involve

- Adequate assessment of the patient in general and wound
- Setting appropriate goals – control of pain and odour may be more appropriate goals than wound healing
- Evaluating progress

2. Pruritis

This is an unpleasant itching sensation which can affect the skin, conjunctiva and mucous membranes.

Common Causes	Management
■ HIV/AIDS<br>■ Pre-existing skin disease – eczema, psoriasis<br>■ Dry skin – common in elderly people<br>■ Allergic reactions – drugs (antibiotics), soaps<br>■ Renal disorders – chronic renal failure<br>■ Liver disorders – hepatitis, obstructive jaundice<br>■ Endocrine disorders – diabetes, thyroid disease<br>■ Cancer – Hodgkin's disease<br>■ Iron deficiency	■ Identify cause and treat, if possible<br>■ Review / modify drug treatment<br>■ Avoid overheating, sweating, hot baths<br>■ Discourage scratching – advise patients to keep their nails short and to gently rub the skin with their fingertips instead of scratching<br>■ Wash affected areas with a solution of Sodium Bicarbonate<br>■ Topical preparations – aqueous cream, calamine lotion, 1% Hydrocortisone cream<br>■ Drug treatment – Piriton 4mg tds, Premethazine 25-50mg bd. A short course of oral steroids in combination with an antifungal agent, may be effective in patients with KS or AIDS

3. Pressure sores (Decubitus Ulcers)

This is an ulcer of the skin +/- subcutaneous tissue, caused by ischaemia secondary to prolonged pressure or friction.
These occur in 20% of patients with advanced cancer.
Common sites for pressure sores are the areas over the coccyx and sacrum, ischial tuberosities, shoulder blades, elbows, heels and back of the head.

Prevention

This is theoretically possible, but a combination of immobility and poor nutrition and incontinence can make prevention virtually impossible

- Good nursing care, by staff and guardians. Frequent position change, good lifting technique, keep skin clean and dry, use of barrier creams, avoid rough clothing or sheets
- Frequent skin inspection for signs of skin damage or inflammation

Predisposing Factors	Management
■ Immobility, leading to prolonged pressure over one or more areas<br>■ Friction – pulling patients rather than lifting<br>■ Excess moisture – incontinence<br>■ Loss of sensation – due to neurological problems, paralysis	■ Avoid further pressure on ulcerated areas<br>■ Analgesia<br>■ Clean wounds prn – apply appropriate dressings; use local remedies, eg pawpaw or honey<br>■ Systemic antibiotics if there are signs of infection

- Emaciation, wasting
- Improve nutrition to promote healing

4. Fungating Tumours

This is a primary or secondary tumour which has infiltrated and ulcerated the skin.. The tumour may grow rapidly and often has a cauliflower appearance. It may ulcerate and form shallow craters or fistulae. Due to the growth of organisms in the necrotic tissues, these tumours generally produce a large amount of offensive exudate. They cause disfigurement, pain, bleeding and bad odour, all of which are distressing to the patient and the family, and may lead to social isolation.

Common causes

Advanced cancers including, KS, Carcinoma of the breast, Cancer of the head and neck

.Surgical debridement of dead tissue is generally not an option due to the high risk of serious bleeding

- Analgesia
- Frequent, regular dressings – aim to keep the area clean, dry and free from infection. Use careful dressing techniques to reduce bleeding due to trauma from dressing removal, etc Wounds may be cleaned with normal saline solution and local remedies, such as applying pawpaw flesh, honey or brown sugar, may also be used. Pawpaw contains an enzyme which both cleans and promotes healing.

Treat topically with metronidazole, which:

- Removes the smell
- Dries up the discharge
- Provides haemostasis
- Clears the infection caused by anaerobic organisms.

Place crushed metronidazole tablets on the fungating area.

- Oral Metronidazole is les effective as there is no blood supply to dead tissue
- Charcoal dressings
- Fresh air – nurse in a well ventilated room
- psychological care – to lessen feelings of isolation and increase morale
- radiotherapy can produce tumour shrinkage and lead to relief of symptoms

5. Fistulas

This is an abnormal communication between 2 hollow organs (eg. rectovaginal fistula) or between a hollow organ and the skin (enterocutaneous fistula)

There are 4 main categories of enterocutaneous fistula

- single orifice in an otherwise intact area, eg. healed scar, where the surrounding skin is flat and in good condition
- single or multiple orifices in the abdominal wall near to bony prominences, surgical scars, other stomas or the umbilicus
- fistula into a small dehiscence of a surgical wound or scar
- fistula into a large gaping wound dehiscence

Common Causes	Management
■ complications of surgery<br>■ infection<br>■ progressive malignant disease<br>■ radiotherapy	■ analgesia<br>■ prevention of skin excoriation around the fistula site – use of barrier creams, Vaseline<br>■ collection of effluent – use of stoma bags<br>■ odour control – Metronidazole tablets; frequent cleaning and dressing

6. Herpes Zoster (Shingles)

Herpes zoster infection is usually an erosive vesicular linear eruption which is unilateral and painful. As immunodeficiency progresses it may involve multiple nerves or be disseminated (as in chicken pox). It is contagious for contacts who have not had chicken pox. Post-herpetic neuralgia and scarring may be very troublesome.

cause

varicella Zoster virus

Management

- Starting acyclovir early in the infection may help resolve the severity of the lesions. Also give oral (systemic) acyclovir if there are complications, such as eye or facial nerve involvement, or if infection is disseminated, or patient is severely immunosuppressed. Give acyclovir 200 mg PO 5 times/day for 5 days.
- Frangipani milk helps reduce pain by paralysis of the nerve endings and helps healing with minimal scarring (Hospice Africa Uganda, 2002). Use on intact skin and not when vesicles are open. Apply or paint frangipani milk 3 times per day before the blisters ruptures. Make the fluid fresh daily.
- Treat or prevent secondary bacterial infection. Treat pain as effectively as possible starting with analgesics according to the WHO 3-Step Analgesic Pain Ladder. For severe or persistent pain, amitriptyline may be effective (amitryptyline 25 mg, titrated upwards for good relief

7. Drug Reactions

Assessment

Fixed drug eruption, Stevens-Johnson syndrome, (target-like lesions and mucosal ulcers) and toxic epidermal necrolysis (TEN) are all common drug reactions among people with HIV/AIDS.

In fixed drug eruption, round red-purple areas develop, may blister, and then heal with purple pigmentation. Lesions are up at the same site with every exposure to the particular drug.

In Stevens-Johnson syndrome, the patient has multiple red-purple lesions, many showing central skin necrosis. The mouth, lips, eyes, and genital mucosae may be inflamed and ulcerated, causing severe pain.

In TEN, skin necrosis causes purple areas, and the skin may strip off in patches, leaving large eroded areas

Patients on antiretroviral drugs can exhibit a range of skin reactions ranging from urticaria or vesicles to severe TEN.

Common Causes

- TB drugs
- Antibiotics – cotrimoxazole
- Antiretroviral drugs
- Anticonvulsants

Management

- Stop the drug causing the reaction. Severe drug reactions can be life-threatening and require immediate attention. Reactions involving large eroded areas are life-threatening and require burn treatment. Apply soaks with water or normal saline and potassium permanganate. Wash the skin gently every day.
- Use antihistamines and steroids. If severe, stop therapy until life-threatening reaction is over.
- Provide eye care and mouth care.
- Maintain fluid and electrolyte balance. Encourage adequate oral fluids, including glucose and electrolytes.
- Be vigilant to identify secondary bacterial infection.
- Monitor liver and renal function.
- Steroids are not indicated in treatment of Stevens-Johnson syndrome or TEN. Treat erythroderma (generalized redness and scaling) due to a drug reaction with a topical steroid.
- Give patient written information on the suspected cause.

Case study

31 year old teacher from Lunzu with Kaposi's sarcoma. He has been on ARVs for 2 months but is troubled by pain and swelling in the left foot. He has multiple foul smelling fungating wounds on his left calf and foot.

How would you assess this patient?

What management would you like to offer?

Session 12: Common Diseases in Palliative Care

AIM: To equip health care workers with knowledge, skills and attitudes in management of common diseases in palliative care

TIME ALLOCATION: 1 Hour 30 minutes

LEARNING OBJECTIVES

By the end of this session participants should be able to:

- Define the common diseases in Malawi that need palliative care
- Describe basic pathophysiology of these diseases
- Explain the disease specific palliative interventions for these conditions

1. Common diseases in palliative care

1. Kaposi's Sarcoma (KS)
2. Non-Hodgkins Lymphoma
4. Invasive Cervical Cancer
5. Cryptococcal Meningitis
6. Carcinoma Of The Oesophagus
7. Hepatocellular Carcinoma

1. Kaposi's Sarcoma

Commonest AIDS related malignancy. Its increase in incidence among homosexual men in the USA led to the recognition of AIDS and KS as an AIDS defining malignancy in 1981.
Human herpes virus type 8 (HHV8) has been identified as the causative agent for KS and has been designated by some researchers as KS associated virus (KSHV2)
In Africa, the incidence of KS is increasing in areas of Africa where both HHV8 and HIV seroprevelence are high.

The tumour originates in vascular endothelial cells. There are usually multiple skin lesions particularly of lower limbs. The lesions have a characteristic purplish appearance reflecting their vascular nature. They appear as macules, plaques or nodules and may ulcerate or bleed. The palate may be involved. There may spread to the regional lymph nodes, liver and lungs.
Children may present with generalized lymph nodes, oedema, KS lesions on the palate with breathlessness when lungs are involved.

Diagnosis

Diagnosis is clinical, supported by biopsy.

Common problems

- Pain
- Ulceration/fungating
- Bleeding
- Oedema
- Breathlessness
- Disfigurement and stigma felt by the patient
- Mass effect eg. upper airway obstruction

Management

Supportive care focusing on pain and symptom control. Pain management using the WHO the analgesic ladder .. All patients should start ARVs. In aggressive disease cytotoxic chemotherapy should be provided if possible.

Specific symptom management

Pain – Analgesic ladder
Fungating wound – apply crushed Metronidazole to reduce smell
Bleeding – local radiotherapy – not available in Malawi
Breathlessness – Low dose morphine, if pleural effussion tapping is recommended

2a. Non Hodgkins Lymphoma

Lymphoma is cancer of the lymphatic system. The lymphatic system is very important part of the immune system, which keeps the body free from infection.
There are 35 different types of lymphoma. These are grouped into two main groups called Hodgkins and non Hodgkins. Hodgkins are distinguished from other types by the presence of a distinctive abnormal lymphocyte called Reedsternberg cell. All types of lymphoma except Hodgkins are collectively known as non Hodgkins Lymphoma (NHL).. Aggressive NHL is more common in younger adults.

Non Hodgkins is a relatively late manifestation of HIV. NHL became AIDS defining in October 1985. in North America and Europe, about 3% of adults and 1% - 1.5% of children with AIDS present with NHL. Lymphoma's occur later in the course of HIV/AIDS than does K/S and at a more profound stage of immunosuppression. It is a WHO stage 4 – defining condition.

HIV associated NHL is of B-Cell origin in 80% - 90% of cases. Other Virus (Epstein Barr Virus) is also known to have an increased risk for NHL.

Clinical features

- Persistent Lymphadenopathy – inguinal, neck axilla
- Hepatosplenomegally
- Fatigue
- Night sweats
- Weight loss

- Itching
- Bruising
- Bone pain
- Recurrent infection

Diagnosis

Fine needle aspirate for cytology.

Common Problems	Management
■ Pain<br>■ Anaemia<br>■ Breathlessness<br>■ Oedema of face: In superior vena cava obstruction<br>■ Obstruction of other major organs	The treatment for NHL is the use of drugs of various combinations (Chemotherapy).<br>The common combination for NHL is known as CHOP – Cyclophosphamide, hydroxdaunorubicin (adriamycin) oncovin(Vincristine) and predniolone.<br>Drug regimens may be adapted depending on local availability and experience.<br>Steroids should be started when complications like superior Vena cava obstruction occur.<br>Use analgesic ladder for pain control.<br>When the patient is HIV positive start ARVs.<br>Relapse is very common.

2B. Burkitts lymphoma

- Tumour that was first described in 1958 by Denis Burkett, a surgeon working in Uganda.
- It is the commonest childhood malignancy in Malawi.
- It is a tumour of Lymphocytes which form part of the white cell population in the blood and lymph nodes. It is one form of non Hodgkins Lymphoma.
- The type of cell affected in B2 is B Lymphocyte which is normally involved in fighting infection by producing antibodies.
- It is very common in equatorial Africa and very rare in Western countries.
- In Malawi most cases come from along the lake and lower Shire Valley.

Causes

In almost all cases of Burkitts lymphoma the cells carry a virus known as Epstein – Barr virus (EBV). Other contributory factors include HIV and malaria.

Signs and symptoms

Jaw swelling is the commonest presentation, swelling is rapid and non tender. Other parts that can also be affected include abdomen, orbit and spine causing paraplegia.

Common Problems	Management
▪ Headache and bone pain when there is central nervous system and bone marrow involvement.<br>▪ Paraplegia with urine/ stool incontinence when there is spinal cord compression.<br>▪ Breathlessness<br>▪ Oedema.	• Give analgesics according to WHO analgesic ladder (if there is CNS an involvement give steroid).<br>• Incontinence: Teach the family about pressure area and frequent turning.<br>• and give advice about using a bottle or a condom catheter<br>• Breathlessness: Explain the cause to the family and Low dose morphine helps.<br>**Specific treatment**<br>Tumour responds very well to Chemotherapy. Cyclophosphamide IV/PO is the drug used with intrathecal methotrecate and hydrocortisone.<br>Cure rate is very good in early stages. Late stages respond very poorly hence need palliative care. Relapse is common with late stages.

3. Cancer of the cervix

Cancer of the cervix is a common tumour affecting women in sub-Saharan Africa.

Several oncogenic types of human papilloma virus (HPV) especially types 16 and 18 have emerged as causative agents for cancer of the cervix. HIV infected women have high HPV infection rates. HIV infected women tend to have a high percentage of abnormal pap smear.

Common Problems	Management
• Pain<br>• P.V bleeding<br>• Offensive smell due to infection<br>• Constipation – due to fear of pains when passing stool or effect of tumour.<br>• Family problems	• Use analgesic ladder<br>• Early stage – surgery<br>• Advanced disease treat symptoms<br>• Offensive smell and bleeding - apply metronidazole powder / pessary<br>• Frequent change of pants<br>• Cleanliness<br>• constipation - treat as per protocol<br>• Counseling the patient and husband about the disease and how they should live as a family. Husbands may be concerned that the cancer can be transmitted. Further sexual intercourse may promote bleeding.

4. Cryptococcal Meningitis

Meningitis caused by the organism 'Cryptococcus neoformans'. It is more common in patients with HIV infection, particularly where the CD4 count is very low.

Common Problems	Management
▪ severe headache – may have gradual onset<br>▪ confusion<br>▪ loss of consciousness<br>▪ neck stiffness<br>▪ photophobia<br>▪ blurred vision<br>▪ nausea and vomiting (vomiting may be projectile)	▪ analgesia – according to WHO analgesic ladder<br>▪ therapeutic tap, to reduce ICP. May be necessary to drain 15-20 mls CSF daily<br>▪ Fluconazole (regimes differ between health units), eg 800mg IV/PO stat, then 400ms IV/PO od for 8 weeks, then 200mg PO daily for life<br>▪ Explain to the patient and relatives about the need for long term treatment<br>▪ Steroids eg. Dexamethasone or Prednisolone, may be added if the patient has severe headache, the use is controversial – should not be used in bacterial meningitis

5. Carcinoma of the oesophagus

Carcinoma of the oesophagus (Ca Oesophagus) is a malignant tumour of the oesophagus. It is common in the elderly, and there is a strong association between Ca oesophagus and excess consumption of alcohol particularly spirits.

Clinical presentation

Dysphagia (difficulty swallowing) is the commonest single symptom. It is progressive, first with solids only then later on with liquids also. There is impact pain (odynophagia), but when it becomes more persistent in the front and back of the chest suggests infiltration and is a bad prognostic feature.

Loss of appetite and weight loss are partly due to dysphagia and advanced disease. Involvement of the recurrent laryngeal nerve causes hoarseness of the voice.

The tumour can spread to neighbouring structures such as bronchi, lungs or the aorta. Therefore perforations may occur resulting in tracheo-oesophageal fistula or other fistulas. Tumour can also spread to the liver through the lymph nodes.

By the time that oesophageal obstruction is causing difficulty in swallowing saliva, cough and other features of aspiration are common and may result in terminal broncho pneumonia.

Physical signs, apart from weight loss, are often absent. Anemia may be present and palpable lymph nodes can be found in the neck (D.J. Weatherall et al (1987)).

Common Problems	Management
• Dysphagia<br>• (Difficulty swallowing)<br>• Odynophagia<br>• (Painful swallowing)<br>• Weight loss<br>• Loss of appetite<br>• Aspiration pneumonia<br>• Anemia<br>• Tracheo-oesophageal fistula, or other fistulas<br>• Psychosocial, cultural and spiritual issues	• Give small liquid foods frequently.<br>• Palliative surgical stenting<br>• Steroids: high dose, short course may assist to shrinks oedema around the tumour.<br>• Analgesic ladder<br>• Nutritional support<br>• Steroids: low dose, short course gives appetite.<br>• Treat with antibiotics.<br>• Advice positioning and small frequent feeds.<br>• Treat accordingly: iron tablets or transfuse blood if appropriate.<br>• Refer to palliative care specialist, a celestin or a bougie tube may be inserted.<br>• Holistic approach and management.

6. Hepatocellular carcinoma

Primary cancer of the parenchymal cells of the liver, common in Africa and South East Asia.
Cause - Hepatitis B virus
Other possible factors
Hormonal – males are affected more than females
Toxin – aflatoxins from aspergillus fumigus, a fungus that contaminates stored groundnuts
Genetic – family clustering of cases is seen
Pathology
The tumour is commonly diffuse, particularly in a liver which is already cirrhotic. It may also be solitary or nodular, sometimes it may be limited to one lobe.

Clinical features

- Affects all ages but it is common in the ages 35-45 years. More common in males than females (3:1).
- The symptoms and signs depend on the local, systemic and metastatic effects of the tumour.

Di**agnosis**
Usually clinical, abdominal ultrasound may assist.
Other investigations may also assist:

- Alphafetoprotein – positive for over 65% (not available in Malawi)
- Liver function test
- Liver biopsy

Common problems

Local effects

- Pain – A persistent pain is felt in the right hypchondrium due to stretching of the capsule of the liver by the tumour. A friction rub can be heard over the liver on auscultation.
- Hard mass in liver – single or multiple nodules can be palpable that gives an irregular surface of the liver
- Ascites – Blook stained ascites can accumulate, if a tumour erodes a blook vessel a sudden blook can cause signs of acute peritonitis
- Jaundice – This can be due to intrahepatic biliary obstruction or due to hepatocellular failure.

Other effects

- weight loss
- weakness
- fever

secondary deposits in the lungs, bone and peritoneum

Management

- Manage pain using the analgesic ladder
- If there is massive ascites causing breathlessness, do therapeutic ascitic tap. A small dose of morphine may help breathlessness.
- Spironolactone 100 – 400mg daily and frusemide 40 – 80mg daily may help control ascites

Case study

You are seeing a 34 year old woman who has been complaining of worsening headache for 3 months. It is now severe, she is crying in pain and has difficulty speaking to you. Her husband died 3 months ago and she has 4 children at home. She carries the youngest on her back when you are seeing her. Investigations reveal that she has cryptococcal meningitis.

1. What further assessment would you like to do?
2. what factors may affect her experience of pain?
3. what should you offer this patient?

Session 13: Anti-Retroviral Therapy in Palliative Care

AIM: To equip health care workers with knowledge, skills and attitudes on the role of ART in palliative care services

TIME ALLOCATION:1 Hour and 40 minutes

LEARNING OBJECTIVES

By the end of this session participants should be able to:

- Describe the current situation of ART provision in Sub-Saharan Africa
- List common ARV drugs used in Malawi
- Explain the eligibility criteria for ART in Malawi
- Describe side effects and management of ARV drugs
- Explain the importance of palliative care in ART provision

1. Background

- In SSA, Uganda and RSA were first countries to start ART in 1990's.
- Botswana was the first country to role out free ARVs
- In 2009 Malawi had over I million people infected with HIV and an estimated 306,000 people needed ARVs
- Chiradzulu was the first district to distribute free ARVs in 2001
- By the end of June 2009, there were 329 ART sites supporting provision of free ARVs
- By June,2009, 266,325 people had been ever started on ARVs and 169,000 were alive on treatment
- It is predicted that, by June 2012 close to 320,000 people will have been commenced on ARVs in this country

2. Common ARV Drugs Used In Malawi

Trade name of the drug	Component Drug	Side effects	Management of side effects
Triomune or nevilast	Nevirapine	Skin rash<br><br>Hepatitis	Monitor. If severe discontinue treatment and refer<br>Incase of jaundice stop treatment and refer
	Stavudine	Mitochondrial toxicity<br>Lipodystrophy syndrome with long usage<br>Neuropathy<br>Pancreatitis<br>Gastro intestinal (GE) effects<br>Insomnia	Discontinue and refer<br>Monitor. If severe discontinue treatment and refer<br>For neuropathy give multivitamins & amitryptiline<br>For pancreatitis refer to hospital<br>For G/E Monitor. If severe refer
	Lamivudine	Mitochondrial toxicity<br>Lilpodystrophy<br>Nausea, Headache<br>Fatique<br>Muscle pains	Monitor if severe discontinue treatment and refer<br>cc content in above box

ARVs reduce viral load to minimal levels, there by giving the immune system opportunity to strengthen.

ARVs need to be taken for life, daily adherence is critical and drug sharing is prohibited

3. Eligibility for ARVs

Adults

- Patients known to be HIV-seropositive AND
- Patients understand implications of ARV therapy PLUS ONE OF THE FOLLOWING
- Assessed as being in WHO stage 3 – 4
- Assessed as being WHO stage 2 with TLC <1200 / mm3
- A CD4 count less than 250 / mm3

Children > 18 Months

- Children known to be HIV seropositive AND
- Guardians understand implications of ARV therapy PLUS ONE OF THE FOLLOWING
- Assessed as being in WHO stage 3 – 4
- Assessed as being WHO stage 2 with TLC < threshold value
- A CD4 count less than threshold value

4. Importance of palliative care in ART provision

- HIV/Aids is still a life threatening incurable disease. Those infected with HIV are still more likely to die earlier than those that are not affected.
- There is a high prevalence of pain and symptom management throughout the trajectory of the disease. ARVs are not available for all; for many, terminal care is the only option.
- ARVs are not available for all; for many, terminal care is the only option.
- Many people, particularly in Africa, present at advanced stages of the disease and palliative care is required. In Africa there is more integration between services than in the UK, with referrals going from hospice to ARV service providers as well as vice versa. Hospices often stabilize people on therapy.
- ART is associated with symptom prevalence. Treatments can be distressing and the symptoms that treatment produces are often not controlled. Symptoms need to be managed to ensure adherence.
- A number of cancers have not reduced in incidence and other co morbidities are emerging.

Although ARVs access is set to expand, access is still limited due to excess number of patients who need ARVs, there by needing palliative care until they die. Therefore patients on ARVs need palliative care to ensure adherence, improve clinical benefit, reduce potential for emergency resistance strains and offer care to those care fails. Above all, control of pain is paramount, basic treatment of opportunistic infections, psychosocial care and spiritual care. All these make patients comfortable while facing difficult times.

Session 14: Children's Needs

AIM : To equip health care workers with knowledge and skills on how they can identify special needs of children

TIME ALLOCATION: 1 hour

LEARNING OBJECTIVES

By the end of this session participants should be able to:

- Define a child
- Discuss children's' needs at different ages
- Identify the special needs of sick children
- Discuss the role of a health worker regards to children needs
- Discuss basic communication skills with children

1. Definition

A child is any human being below the age of 18 years.

2. Needs of Children

All children need to be provided with the basic requirements for survival, growth and development.

The needs are:

- Adequate care
- Shelter
- Food
- Clean water
- Education and supportive home environment

Additional Needs:

- Protection from abuse and neglect
- Protection from all forms of exploitation
- Access to health and health services
- Access to appropriate information
- Privacy
- Freedom of association

- Recreation
- Leisure opportunities

NOTE: Children have different needs at different ages of their growth and development. But sick and orphaned children have special needs which a health worker has to look at and understand them if they are to help these children and carers with challenges. All the needs mentioned above can also be defined as children's rights. (UN – convention on the rights of the child – 1996)

Needs in relations to age:

**Age**	**Needs**
0 – 1 year	Love<br>Security<br>Physical contact<br>Bonding
1 – 5 years	Approval<br>Attention<br>Safe, boundaries/support<br>Opportunities to explore<br>Independence
6 – 12 years	Mastery<br>Recognition
12 – 18 years	More freedom<br>Direction to future life<br>Finding own values compared to parents values

Figure 8: Needs assessment of a sick child

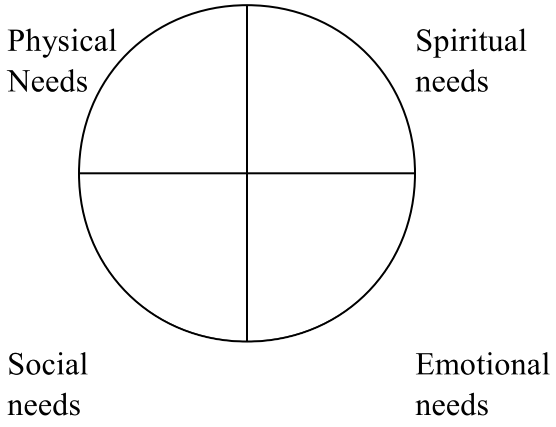

Physical Needs
These include food, shelter, clothing, sleep and fresh air. Most parents/carers of HIV infected children are unable to provide these basic needs may be due to the parents ill health, death or general poverty

Psychological/Emotional Needs
Children need care, attention, security, love acceptance and understanding. HIV evokes stigma and children who experience frequent sickness, and loss of parents are often rejected, isolated and discriminated against such children suffer from loss of esteem, self denial and subsequent withdrawal.

1. Care and Love
Children with terminal diseases need substantially more care, love and attention than other children because of the complex nature of their illness, pain, stress, fear anxiety and fatigue being experienced. The health care worker can provide additional care

2. Acceptance
It is essential that a sense of acceptance of the HIV infected child is encouraged. Sometimes these children are abused through neglect, mistreatment, discrimination and abandonment, which deny their attainment of their needs and right.

3. Security
Sick children often experience increased feelings of insecurity due to nature of illness, frequent exposure to clinic settings, loss or sickness of parents, inability to confide, change of environment and change in lifestyle. These can impede a child's growth and development and abilities to respond positively to interventions.

4. Confidentiality
Respect child's right to privacy and confidentiality especially those affected by HIV/AIDS to minimize their fears related to being stigmatized rejected and discriminated against.

Social Needs.

Children socialize through play, peer and gender identification, all which contribute to their growth and development

Play facilities, communication, stimulation, and experimentation provides an opportunity to explore growth through emotions. Often sick children find it difficult and fear participation in physical games and this can lead them to be quiet, reserved, miserable and lonely. Also some carers tend to over protect them from social activities

through fear of the impact on the child's health. However, it is important that they are encouraged to interact and play with others whenever possible.

Education is a basic need and right for children. These children are often denied access to education due to either stigma of family members, sense of helplessness for the child's future, or the ill health of the child. Children should be encouraged to participate in all normal activities of a child of their age and development.

Spiritual Needs
Often difficult to define though children will mimic spiritual words, actions and tendencies for the people and the environment to which they belong.
A sick child living with stress, pain and illness may require spiritual support as a way of instilling hope.

Role of A Health Worker
Long-term illness poses a challenge to health workers and carers when the child's needs are not identified, understood and addressed.

Some children are likely to develop abnormal behaviour like aggressiveness, restlessness, sleep disturbances, bed wetting, truancy, school refusal and bodily complaints if their needs are not properly identified.

It is therefore vital to establish which needs are not being met and help such children to talk about their problems/needs.

3. The health care worker should be able:

- To identify and discuss the child's problems pertaining to his/her illness and respective causes.
- To categorize the nature of need i.e. Physiological, psychological or social.
- To discuss other needs of the child in relation to the causes, effects and possible alternatives.
- To create supportive environment to enable him/her to express their feelings about the general situation.
- To provide information on other available services for referral.

4. Basic communication skills with children

- Self reflection
- Self awareness
- Speaking the language of children .

Session 15: Spiritual support

AIM: To equip health care workers with knowledge, skills and attitudes in providing spiritual support

TIME ALLOCATION: 45 minutes

LEARNING OBJECTIVES

By the end of this session participants should be able to:

- Describe concepts of spirituality and religion
- Explain the importance of spirituality in palliative care
- Identify the spiritual needs of an individual
- Explain the management of spiritual needs of the patient

1.0 Spirituality

Spirituality defines a relationship that exists with God throughout life and often comes to a greater meaning during times of crisis especially when coming towards old age or deaths.

Spirituality involves:-

Search for meaning and purpose in life

- Identity thoughts and feelings and the ability to accept and be comfortable with who you are.
- Relationship with others, friends and families as well as connection with the community and the world.

Spiritual needs may range from religious rituals such as prayers or holy communion, to secular observances like music/poetry. (Connor, 1988 And Dudley 1995).

Religion:-

- The means by which we organize our spiritual yearnings.
- Religion is the mode of transport while spirituality is a journey.
- Usual path to introduce God
- Offers formal structures to reach spirituality
- Ideally includes love for all if based on holy books.

- Gives basics to spirituality.
- Has often been abused by people seeking power
- All formal religion try to help people in their search for inspiration and insight when they are forced to grapple with great existential questions provoked by suffering (Becker 1973).
- The sense of purpose and meaning that religion provides is a comfort in the face of crisis. (Kushner 1981).

1.1 Spirituality and Religion

Spirituality is our relationship to God or the unseen/supernatural being and is reflected in our life. It is not dependant on our religion. Religion gives us structures in which to develop our spirituality. We must respect spirituality developed throughout life in our patients and families.

2. Importance of Spirituality

Each one of us will interpret spirituality according to our own culture, beliefs, experiences and social background. The meaning of spirituality may change as we journey through life. (Carol, 2001)

The spiritual care provider should be a person with deep sense of God and love for the sick in carrying out his or her holistic service.

- Spiritual care of the sick is very important in Africa. Once well done, it can make a difference to the sick and their families.
- Religious beliefs are not a consideration for accepting patients for palliative care. Palliative care supports patients and families in their own religious beliefs.
- Health care workers are prepared to work together with any denominations as long as they do not require that they try to convert patients and families to other religion.

"It is in giving that we receive."
The inspiration for palliative care comes from the suffering patients and family.
The centre of care is love for the patient and family and our concern to relieve suffering provide support with love and to be there as we are needed.

Health care workers, should consider:

- Awareness of spiritual needs of those with terminal illness
- The spiritual needs of this particular patient.
- How many may meet those needs.

3. General spiritual needs

We all have spiritual needs. What are they?

- Need for meaning to life
- Need to receive love
- Need to give love
- Need to have a sense of forgiveness
- Need for hope and creativity

4. Management of Spiritual Needs

Caring with compassion

- Understanding
- Empathy
- Being present and listening is often answering the need of the patient
- Don't give false promise like; you will be feeling fine soon, or theological reply like, "of course God loves you, Jesus said so."
- We need to reflect back what they are trying to say, let them know that you understand. Also to comfort them. involve the family.
- They may ask you to pray. Be prepared to do so. Don't suggest prayer to an obviously angry patient or whom has demonstrated negative feeling to God.
- Refer to appropriate services according to the wishes of the patient

Session 16: Breaking Bad News

AIM: To enable health care workers to acquire knowledge, skills and attitudes needed to break bad news effectively to patients and their families

TIME ALLOCATION: 1 Hour 30 minutes

LEARNING OBJECTIVES

By the end of this session participants should be able to:

- Define bad news
- Discuss the impact of breaking bad news on the patient, family and the health worker
- Explain the process of breaking bad news
- State the principles for communicating bad news
- Discuss the concepts of Denial and Collusion

1. Definition of bad news

Bad News is any information which alters a patient's view of their future for the worse.

- The bigger the gap between what the patient expects and the reality, the worse the news is.
- The way in which bad news is given has been shown to affect how the patient and family cope in the future.

2. Impact of breaking bad news

Patients often feel that they lack information and thus lack control over their situation. By giving adequate opportunity for discussion it is possible to:

- Reduce uncertainty about the future.
- Reduce inappropriate hope (which is demoralizing) - but may be difficult to do.
- Encourage informed choice of management options

- Enable appropriate adjustments to the reality of the situation.
- Maintain trust between the patient, the carers and the health care workers.

3. Process of breaking bad news

Remember that it is impossible not to communicate. Avoidance of discussion and negative body language usually leaves the patient feeling abandoned, anxious, guilty or depressed. A conspiracy of silence or the raising of false hopes may deny the patient the opportunity to use his/her remaining time the way s/he would wish.

When it can be anticipated that bad news is to be given, consider the following points:

1. The meeting: Ensure you have time, and are not exhausted. Arrange for privacy, sufficient seating; avoid interruptions, whenever possible offer the opportunity to have a close family member or friend present.

2. Ask what the patient understands of his/her situation. 'What do you think is going on?' 'Would you like me to tell you more about your illness?' Do not impose information. If the patient does not want to know, would she/he like you to explain to a family member? Ask the patient, and document this.

   Give a warning phrase/sentence to the patient. By using the patient's own phrases and avoiding medical jargon wherever possible, start to give a range of possibilities. This will include using euphemisms, e.g. shadow, lump, growth – which may subsequently require fuller explanation. Allow the patient to absorb the information at their own pace. If they do not ask questions or deny or protest at information given, do not continue to give more information at this stage: every patient has the right to know about their illness but also has the right Not to know. Allow denial.

3. Avoid assumptions. If a patient asks a question, never assume that you know what they are referring to. Ask a question to clarify, or you may give an inappropriate answer – 'How long will it be?' may be referring to discharge home, not prognosis! If in doubt, reflect the question back: 'How long will what be?'

4. Explanations must be clear and simple, in terms that the patient can understand. Diagrams often help, but may also become barrier between patient and health care workers. Avoid detailed explanations and treatment options; these are best discussed at a subsequent meeting. "Once he told me it was cancer, I did not hear anything".

5. Be positive: optimism is supportive, pessimism is not. Say for example 'we may not be able to cure you but there are things we can do to make you feel better and cope with your illnesses.

6. Confirm that the patient has understood the information so far.

7. Allow ventilation of feelings. Do not discourage emotions and acknowledge distress. Use prompts as necessary, such as ‘Is there anything that you are worried about?’ Listen and allow them time to think and how to phrase the questions.

8. Summarize the situation and arrange for a follow up meeting, stating the day and time if possible. In summarizing, emphasize the positive, and outline future treatment plans if appropriate. written information may be useful.

9. Ask who may be told about the diagnosis – ‘Would you like me to talk to your family?’

Ensure that the patient’s own health care worker is informed of what was said, e.g. through the health passport, although what was said and what the patient heard may be quite different.

4. Principles for communicating bad news
Do:

- Wherever possible, sit down to be on the same level as the patient – this is reassuring and courteous and signifies that you are ‘with’ them. This is just as important as with children too.
- Spend the first part of the interviewing listening to what the patient is saying or asking.
- Note questions or topics avoided by the patient.
- Watch for non verbal messages , e.g. posture, eye contact, hands, facial expression.
- Respect the patient’s right to ‘denial’. Patients will often ‘selectively perceive’ only that information they can cope with at that point in time.
- Remember that more than 60% of what you communicate is by non verbal means, e.g. posture, eye contact, attitude.
- Allow pauses for taking in and digesting what you have just said – move at the patient’s pace.
- Attempt to give information that is appropriate for that individual patient’s needs at that particular point in time.
- Realize that most patients become aware of their situation gradually rather than in a ‘once off’ confrontation.
- Realize that it is possible to communicate the ‘gentle’ rather than the ‘bitter’ truth by one’s attitude and by emphasizing positive aspects of the present or future situation.
- Realize that patients can and do cope positively with truth about their illness.

- Realize that certain euphemisms may be appropriate, e.g. tumour or growth. Try to find out what the patient understands by these words.
- Use the word "cancer" if appropriate.
- Realize that the patient who denied or did not want the information about his/her illness in the past may need and be ready for information at another time.
- Realize that there is no general rule as to how much to tell.
- Realize that hope is best communicated by genuine concern and reassurance of continuing care, 'no matter how things develop'
- Express your humanity and warmth.
- Realize that patients will often be shocked upon hearing bad news and that their many questions may only surface later.
- End meeting in which bad news is imparted by arranging to meet again in the near future to answer any questions. This also demonstrates to the patient your commitment to them.
- Write any information/insight you may have given/received in the patient's health passport.

Tell staff on duty what you have said. They may be involved in future discussions.

On the other hand, Do not:

- Ask the relatives whether or not the patients should be told. (This is unfair both on them and the patient).
- Agree not to tell the patient because the family forbids this.
- Be afraid of patients or relatives expressing negative feelings or crying. This reaction may be entirely appropriated and not caused by your clumsiness.
- Tell lies which would lead to a breakdown of trust at a later stage.
- Give more information than the patient needs, or is asking for.
- Use language that is too technical for the patient or family to understand.
- use misleading euphemisms, e.g. ulcer
- have general rules about 'telling', e.g. "Everybody must be told everything" or "Nobody must be told anything"
- Always answer direct questions directly. It may be appropriate to do so but often direct questions such as "Is it cancer?" or "Am I dying?" contain hidden question such as "Will I have uncontrolled pain? " or "Should I make a will?". These hidden questions can be discovered by replying initially with a question such as "I wonder what makes you ask that?" One may discover that the patient already knows about his diagnosis but he is looking for clarification or reassurance.
- Talk from the end of the bed with one foot in the door!

And finally:

- Be aware that it is unethical and technically a breach of confidentiality to tell the relatives without the patient's consent.

5.0 Denial

5.1 Definition

Denial is a basic primitive coping mechanism to protect people from information or events with which they can not cope. By blotting out unpleasant facts it allows them to continue to function. Denial may be practiced by the patient, family and/or by the health care worker. Denial can be a very normal protective measure but in some situations it can be harmful and should then be challenged.

Health care workers who feel that denial is unhealthy need to ensure that they are intervening in the best interest of the patient, not just because they feel the patient and family should fully accept the situation.

5.2 Management

It should be remembered that, in order for patients to be able to deal with their emotions, they need good symptom control.

The first step in assessing denial must be to establish that the patient has been told the diagnosis in terms that he/she can understand. Is there written confirmation in the notes? What terms were used?

If the patient is in denial, decide if this is healthy or unhealthy. There are two main aspects to consider:

- Is the denial reducing emotional distress?
- Is the denial affecting help-seeking behaviour and compliance?

If the patient is functioning well and the denial is not prejudicing treatment, then it may be quite healthy. On the other hand, if the denial acts as a barrier and prevents the patient from seeking treatment (for example, a woman denying the significance of early breast cancer) then it should be tackled. It is also appropriate to intervene in cases where the patient is in denial but is displaying a great deal of distress or pain that is not responding to treatment.

If the patient has dependants for whom provision must be made and planning is blocked by the patient's denial then this too is a situation where the denial should be challenged.

By gently exploring the patient understands and helping them to a more realistic view point it may help to resolve distressing symptoms/situations.

- Denial can be difficult for health care workers to work with, particularly when they prefer to communicate openly. However we must respect the needs of the patients and their ability to cope with the information at that particular time. Any attempts to modify denial should be for a specific reason, for example, improving compliance/adherence to treatment, reducing emotional distress or planning care for dependents.
- Phrases such as 'What if ….' and 'it's sometimes best to plan for the worst and hope for the best' can help to open up the conversation, but it is unrealistic to expect all patients to come to terms with their impending death. Indeed some are too ill and too close to death to open up the conversation.

- Carers may deny the seriousness of the illness and expect too much of the patient. They need extra support to understand that life cannot continue as before.

Health care workers may also deny the seriousness of the patient's condition and thus continue with or initiate inappropriate treatments. Teamwork and cross-referral often help in the transition from curative to palliative treatment.

6.0 Collusion

6.1 Definition:

Collusion occurs when the family conspires among themselves or with health care workers to withhold information from, or lie to the patient.

Collusion is a common problem particularly in the early stages of illness. We must remember that families are often well-intentioned and acting in what they believe to be the best interest of the patient. In trying to shield the patient, the family's actions are of a protective and a loving nature attempting to spare their loved one from further pain and distress.

We should also respect the fact that the patient has the right to information about his/her diagnosis first. Has the patient given permission for you to disclose information about diagnosis to their family? It is important to establish whether the family is trying to protect themselves or the patient.

6.2 Management

Listen to the family: they know the patient better than you do and may have valid concerns which should be explored. “What do you think s/he is expecting to hear?” “How has he coped with bad news in the past” “Has anyone else in the family had life threatening disease ,For example cancer?” Having given them an opportunity to express their concerns, show that you empathize with their feelings and help them to understand that the patient has the right to information. Do not rush this, or the family can become quite antagonistic and this may be hard to reverse.

Reassure them that you will not walk in to the patient and impose information, but that if s/he asks questions you should answer them honestly but gently. ‘If s/he is brave enough to ask, s/he deserves an honest answer.’

Explain to the relatives that if the patient asks a question it is often answered with a question in order to establish exactly what information the patient is seeking, e.g. “Is it cancer doctor?” If we reply “Is that what you think the tests may show?” The patient may then go on to confirm their suspicion or may declare that they do not want all the details, or that they would like their spouse to be present.

The relatives are usually distressed and coming to terms with bad news themselves, with a whole host of concerns and worries for the future. They have often not considered the consequences of their actions and not yet appreciated how difficult it can be to live with a lie and how isolated the patient will become, if the family and the health care workers collude and pretend that all will be well

Usually the family can be reassured that no one is going to shout out bad news and that the issue will be handled sensitively. They may initially find talking openly to the patient very difficult which is where a joint conversation between the patient, family and health care workers can help to open up channels of communication.

Role Play

A 36 year old business man from Dowa has had problems with abdominal swelling and itching for the past 8 months. A liver biopsy result is back which confirms hepatocellular carcinoma.
Task;
Break the bad news to the patient

Session 17 : Wills and Inheritance

AIM: To equip health care workers with knowledge, skills and attitudes in will writing and inheritance

TIME ALLOCATION: 45 minutes

LEARNING OBJECTIVES

By the end of this session participants should be able to:

- Define inheritance and Will.
- Explain the importance of will writing.
- Describe the contents of a will.
- Explain the process of writing a will
- State the conditions for changing a will.

1. Introduction

Quite often death robs people of our dear ones, leaving people behind with broken hearts. More so for the children and spouses (orphans and widows widowers) who are so much emotionally torn apart that they tend to be unsure of their survival following the death of the breadwinner. Palliative care providers should usually advise patients and their guardians to prepare the process of inheritance of whatever property the patient has.

Definition OF Terms

1.1 Inheritance

It is a process by which property left by the deceased person is shared out among specific persons according his/her wishes or according to the manner laid down in the law.

There are two ways of inheriting:

1. Where there is a will left by the deceased person.
2. Where there is no will by the administrator general.

1.2 Will

It is a written legal document, which expresses the wishes of the person, himself or herself in the presence of any two normal persons who are 21 years and above on how his or her property is to be shared among the people named in the document after the

owner of the property dies. A will can also contain other things that the person making it would like to be carried out e.g., it may give the name of the person who will be responsible for making sure that the wishes of the person are carried out once he dies. It should be written in a simple and well known language.

If the person making the will cannot write, he/she can ask another person, whom she/he trusts and tells him or her what to write. A lawyer can also write it on payment.

2. Eligibility for will writing
Any one, male or female, married or single.
The person should be 18 years old or above, of sound mind, and aware that she/he is making it.

3. Importance of writing a Will.

- Makes sharing of property easy,
- Gives one a chance to say whether he/she owes anybody a debt and how the debt is to be paid and vise versa..
- Ensures that people get what is allocated to them and avoids questions and quarrels among relatives.
- Avoids property grabbing.

.

4. Contents of the will

- Name and place one's live.
- The day, month and year on which a will is made.
- List all the properties one owns, the property should be own property not somebody's.
- Name of spouses, children and parents(a must) and any other beneficiaries
- How the property will be shared.
- If anybody owned anything (debt), name him/her state what was owned and how to pay him back.
- Guardian of the children if they are young.
- Names of persons who should carry out the wishes as stated in the will. Such person is called executor.
- Signs all pages of the will to prevent forgery if one cannot write then thumb mark it.
- When signing the will, two people should witness but are not supposed to read it.
- The two witnesses should write their full names, addresses, occupations on the will and then sign it.

5. Custodian of the will
The original copy should be at either

- A bank safe deposit box.
- Offices of resident judges.
- High court registry.
- District Commissioner's Office.

Copies can also be kept by a trust worthy friend, religious and traditional leaders.

6. Reasons for changing a Will
When some properties are added or lost.
When the list of beneficiaries changes

In case of no will
Refer to social welfare officer and administrator general

The work of the guardians in relation to inheritance.
To look after and guide the young children.
To look after the property of the children making sure it is used for the children only.
'When the child grows up the guardian must handover the remaining property to the child the law says that a guardian who misuses property of a child, must pay it back'

Session 18: Holistic Patient Assessments

AIM: To equip health care workers with knowledge, skills and attitudes in palliative care patient assessment

TIME ALLOCATION –2 hours 45 mins

LEARNING OBJECTIVES

By the end of this session participants should be able to:

- To take a holistic history
- Demonstrate a patient centered approach
- Demonstrate effective communication skills
- Develop plan of action for patients and their families
- Refer patients to relevant services

A. Holistic assessment

- Assessing patient and family needs holistically is important to address the physical, psychological, social and spiritual needs of the patient.
- The care provider should ask questions, listen and observe carefully.
- The checklist should be updated every time the care provider visits the patient.

1. Home assessment

During a home visit, the care provider should;

- observe the general cleanliness of home surroundings, waste disposal,
- Ask about availability of safe water and its utilization
- Ask about availability and food utilization in the home

2. A general assessment of the patient/client should follow to find out the needs of the patient and family. Assessment should include;

a . Physical assessment

- Observe the general condition (weak, sick, dehydrated)
- Ask patient for any problems/complaints by asking the following questions

- How are you feeling?
- Do you feel pain? Where?
- Ask if the patient had sleepless nights, diarrhoea, fever, nausea or vomiting?

- Ask about feeding pattern and any feeding problems
- Are if the patient is taking any medications or other remedies? Checking on the treatment/drugs taken, side effects and adherence

b. Psychological Assessment

• Ask if the patient has any worries or concerns?
• Observe if the patient is confused or depressed?
• Ask if the patient has any person whom he/she can talk to when he/she has problems?
Ask if the patient is worried about the family – wife /husband, children
(who is looking after them , their needs, do they children understand what is happening, are they going to school?)

c. Social – cultural assessment

• Ask if there is someone who helps the patient and What is the relationships ?
• Ask how the family is managing or coping in terms of finances, food, transport, provision of essential needs?
• Ask what the patient what he/she thinks is the cause of the illness

d. Spiritual assessment

• Ask if the patient is belonging to any denomination/ group
• Observe if the patient has fears or worries
• Ask if the patient is visited by the church members or receives support from the church/mosque?
• Find out if the patient needs prayers? Always refer the patient for pastoral care.

B. .What the care provider can do after assessment

After the assessment the care provider should;
• Help the patient and family identify and describe their problems.
• Help them begin to work out their problems.
• Provide counseling, education and emotional support.
• Respect the abilities and choices of the patient.
• Encourage community and pastoral support

B. Reporting and referral

Palliative care patient assessment using case studies. Format refer annex 2.

Session 19: Death and Dying

AIM: To equip health care workers with knowledge, skills and attitudes required to provide end of life care

TIME ALLOCATION:1 hour and 30 mins

LEARNING OBJECTIVES

By the end of this session participants should be able to:

- Identify the fears of dying patients and families
- Describe the palliative care approach to end-of-life care
- Describe basic comfort measures that ease the dying process for patient and family

1. Introduction

Death may be sudden or anticipated. Sometimes guardians do not recognize when the patient dies. Relatives of the patient have fears and it is important to assess these fears in order to assist them.

2. Fears of dying patients and relatives

The family members of the dying patient may have many fears as the condition of the patient weakens such as:

- Fear of not being able to cope with the death event.
- Fear of the patient dying in agony.
- Fear of being alone in the house at the moment of death.
- Fear of how life will go on when their loved one has died.
- Fear of not recognizing that the patient is dead.
- Fear of the unknown

3. Palliative care approaches to end of life care

a) Preparing the patient for death

Get close to the patient and remember the quality of good communication i.e. warmth genuineness – empathy – Compassion – Honesty.

- Explore: if there is anything troubling.
- Acknowledge their thoughts and feelings.

- Give Reassurance and make sure someone stays with the patient.
- Attend to spiritual needs i.e. if would like someone to pray

b) Preparing the family for death

- First assess your own fears honestly and enlist team support.
- Listen actively to the patient and family addressing their concerns.
- Keep in mind the different emotional responses; shock, anger, denial and anxiety
- Give any anticipatory guidance for the patient and family on what to expect.

3. Comfort measures

Comfort measures shall be provided depending on the presenting signs and symptoms of impending death

1. Decreasing Social Interaction.

Many dying patients remain aware of their surroundings until the time of death. This awareness may be limited, they may be confused about time, mumbling, staring into space, plucking the bedclothes, odd movements of hands, seeming to see things. It is thought that some of these behaviors are due to failing circulation of the blood, others due to the closeness with the next world, and others due to dreaming or memories.

It is important to remember that –

Hearing often remains intact even if comatose.

Dying people often have periods of agitation.

Points for care when the patient is in this state:

- Explain to the family what is happening, and encourage them to allow the patient to rest.
- Encourage the family to be observant.
- Continue skin care with explanation and teaching
- Keep surroundings familiar.
- Encourage the family to continue talking with the patient, saying farewells and to let go.
- Encourage the family to use therapeutic touch (i.e. holding hands etc) with the patient.

2. Pain

Points for care when the patient is in this state:

- Do not stop analgesics if the patient is comatose, continue giving regularly
- Monitor pain relief carefully.
- Drug dosage may need to be reduced: side effects may be more prominent at this stage.

3. Decreasing food and fluid intake.

The patient no longer has an appetite or feels hungry. He/she may feel very little thirst.
Points for care when the patient is in this state:

- It is important to respect the patient's wishes.
- Keep the mouth clean and moist.
- Support the family; show that changes are a normal part of the dying process.
- Assist family if questions arise about readmission or rehydration.

4. Changes in elimination

Passing urine decreases or even stops, the bowel movements are fewer, incontinence of urine and stool may occur.

Points for care when the patient is in this state:

- Reassure the family members that this is almost never uncomfortable for the ill person.
- Assist and educate the family on skin and pressure area care, use of mackintosh. Put a catheter when it is necessary.

5. Respiratory changes

There may be changes in breathing pattern, cheyne- stocks respiration, grunting,
'Death rattle' (a rattling noise produced by oscillatory movements of secretion, mainly in the hypo pharynx, associated with expiratory and inspiratory phases of respiration)

Points for care when the patient is in this state:

- Anticipatory guidance before this happens.
- Reassure the family that this is not uncomfortable or a sign of pain to the patient, relatives do get very distressed with the death rattle.
- Repositioning of the patient especially can alternate the side the patient is lying on to prevent aspiration if there are excessive secretions.
- Keep the mouth moist and clean, with regular mouth care.

6. Circulatory Changes

Extremities (legs and arms) are cold, sometimes appears grayish due to low oxygen supply.

Points for care when the patient is in this state:

- Gently ensure that the family understands that death is near and this is not comfortable for the patient.

7. Final signs of death the family should be prepared for:

Be aware that not everybody has experienced someone dying, and that every death is different. The family may not realize that the patient is dying or has died. It should be explained simply when death occurs.

- Breathing ceases entirely,
- Heart beat and pulse stops,
- Patient is totally unresponsive to shaking, shouting etc.
- Eyes may be fixed to one direction.
- Eyelids may be open or closed.
- The skin tone changes

Dying is a special time for both patient and family, be aware of what each one is going through. It is a time of reconciliation, forgiveness, sharing feelings of grief and loss, a time to draw together in sorrow and suffering. The patient is losing all that he hold dear in his/her life, and the family are battling in how life will go on after their loved one has died. Therefore be sensitive and support the family.

Session 20: Grief and Bereavement

AIM: To equip health care workers with knowledge, skills and attitudes in the management of grief and bereavement

TIME ALLOCATION: 1 hour

LEARNING OBJECTIVES

By the end of this session participants should be able to:

- Define terms associated with grief and bereavement
- Describe the grieving process
- Discuss the management of grief and bereavement
- Discuss the needs of grieving children

1.0 **Definition of terms associated with grief and bereavement**

Grief is the normal response to any loss, not just death. It is the process of adaptation to the changes that come with loss.

Anticipatory grief is the grief that patients and their families experience before the actual death, when they are anticipating their loss.

Mourning refers to rituals such as funerals and burial which are rooted deeply in our religious, social and cultural traditions and beliefsAttachment, loss and grief are fundamental to life. Grief is not an illness. It is a healthy, even though painful, natural response to loss.

Bereavement refers to loss of something dear to a person, family or community. Bereavement to the majority of people means death of a loved one and the grieving and mourning that follows.

Bereavement support is part of palliative care. A family's needs do not end with the death. In fact many of them become greater – for example the care and support of widows and of children, who may have now lost both parents.

Understanding the grieving process can help the care provider give more supportive care, both before, and after death. It can also help the carer to be more comfortable when helping grieving families, and more attentive to their needs.

The bereaved generally rely for support on their family, friends, and social and religious communities. The customary rituals of mourning also help them cope with the death. They may, however, also depend for support on health care providers with whom they have developed a trusting relationship. Care providers may provide a safe, comforting place to talk and express feelings, thoughts and needs as they struggle to cope with their grief.

It is therefore very important that health care providers be equipped with the knowledge and skill to support and counsel grieving families, and to work cooperatively with the people and the structures in the community that already offer support.

2. The grieving process

Grief affects every part of our being: our body, our mind and our spirit. It affects each person differently in terms of its intensity, duration, and effect on life. Some people suffer long term effects that can lead to serious physical, psychological and spiritual problems that can make life unbearable.

Although each of us experiences grief in our own way, we can identify some general reactions. These reactions come and go, or they can persist for a long time. They are different for each person. They do not all appear for everyone. They may be short lived or last a long time.

According to Kubler-Ross (1969); there are 5 stages of the grieving process of dying patients. Her work helped people to start talking about grief, death and dying. These are painful subjects that, in many societies, people traditionally avoid. She claimed that such avoidance contributes to the pain and suffering of patients and families. She found that people who were able to speak of their grief - with a person who really listened found great comfort and relief from their suffering.

Knowing these stages can be useful but not everyone experiences all of them. Many of the stages may or may not happen and they are not in any order, but they are important. The stages are as follows:

1. Denial – e.g. this can't be true, it has not happened
2. Anger – directed at people, God, fate, the person who has died
3. Bargaining – "if I am very good maybe he will come back"
4. Depression – a long term sadness with an effect on the ability to function
5. Acceptance – a serenity and acceptance of the death

3. Management of Grief and Bereavement

The grieving person is faced with a great deal of hard work - spiritual, emotional, social, practical and physical - as he or she deals with the 'tasks' of grieving. There are at least five tasks that the grieving person must complete in order to heal from the wounds caused by the loss. The feelings of grief and pain are universal to all human beings, but How People Mourn and express their grief varies from culture to culture, and from person to person.

Task 1: To accept that the loss has really happened – this is a mental task

When a person dies there is often the sense of unreality. The first task is to face the Reality that the person is dead and will not return in this life.

Many people find themselves calling out, or searching for the lost one. They may "see" or "hear" the person they have lost. This is a normal part of grieving.

If a person cannot accept the reality of the loss, they Deny, or cannot believe, that it has happened. There are many emotional ways of doing this. Denial may help in the short term, but if practiced for a long time can be very damaging.

To get on with life, a person needs, at some point, to face the reality of the loss. There are ways of helping a person do this.

How Can You Help?

- Allow them, and even encourage them, to talk as much as they want to about the illness, their memories of the deceased, the death, the funeral etc –
- Listen patiently. Ask sensitive questions.
- Help family and friends understand that a person often needs time to realize the finality of the loss.
- Just "be there" even if the person doesn't want to talk – do not avoid them

---

Task 2: To work through the pain and emotions of grief – this is an emotional task

It is important to work through the pain and deal with the emotions of grief in order to be able to heal. But a person may do everything to avoid the pain of grief. This can be

done in many ways that are damaging - such as drinking, promiscuous sexual behaviour, drugs.

There are many ways that society and culture make this task a very difficult one. It may be unacceptable to express feelings, especially for men. Feelings may be considered unhealthy, bad luck or a sign of weakness. People may consider it best to provide distraction, or, they may simply avoid the person who seems sad because it makes them feel uncomfortable or helpless.

One of the aims of helping a person deal with this task is to ensure that they do not carry the pain with them for the rest of their lives, and become chronically depressed or ill. Grieving people are in crisis and vulnerable to immuno-suppression and illness.

How Can You Help?

- Allow the person to express feelings such as guilt, anger, fear, shame, sadness, anxiety, helplessness – these feelings are an important part of grieving
- Be patient and a good listener
- Provide a safe place and time to grieve
- Do not judge another person's feelings
- Help them find acceptable ways to express their feelings with family and friends
- Reassure them that such feelings are part of the grieving process – he or she is not "crazy"

Task 3: To manage the chores of daily life without the person who has died – this is a practical task

The bereaved person has now to adjust to perhaps living alone, or living with other family members, perhaps leaving a home, raising children alone, managing daily life without the help of the person who has died, making decisions alone, learning new skills, taking on new roles, living with a reduced income, etc. For children it may mean living with new family members, or carers, acquiring new brothers and sisters, changing schools etc. The bereaved person needs to learn new ways of dealing with the world.

Some people do not adapt, and refuse to develop the skills needed to cope, or they may withdraw from the world, regress, become helpless and dependent.

One of aims of helping people deal with this task is to support them and help them to find ways of living as well as possible in their changed world

How Can You Help?

- Help the bereaved identify problems
- Help find ways of problem solving and making decisions
- Help the person find ways of dealing with family, cultural and social pressures
- Encourage the person not to make big decisions too soon
- Do not promote a sense of helplessness – help empower the person
- Help the person find supportive resources in the community

Task 4: To move on with life and resume relationships – this is an emotional task

You cannot forget a person who has been part of your life, or a person you have loved. However, to move on in life a person must find new meanings and new relationships. Some people find this very difficult. They are stuck in the past and cannot move on with their life. This can be seen in a mother who mourns her dead child while neglecting the needs of her living children. This can persist for years.

The aim, then, of helping a person with this task is to help them find ways of moving on emotionally, perhaps giving them permission to do so. Some people just need to find a place in their heart for the person they have lost that allows them to live and even love again.

How Can You Help?

- Help the person find ways to move on with their life that they can accept
- Help them find ways to reassure themselves that the person who has died is in a good place
- Help them find ways to honour the person's memory that allows their life to go on
- Point out the benefits of moving on – to themselves, to others
- If there is guilt, help them find ways to deal constructively with their guilt

---

Task 5 – To find spiritual comfort and meaning – this is a spiritual task

Spiritual beliefs and religion can be of great comfort to the grieving person and can give answers and hope to such agonizing questions such as: Why did this happen? How could it happen to such a good person? Why do bad things happen to good people? How could God let such a thing happen? Many grieving people need to blame someone or something for their loss and pain. It is not uncommon for them to blame God, and then to feel overwhelmed with guilt and even fear.

How Can You Help?

- Refer the bereaved person to their priest or pastor, or spiritual leader, for help in finding answers to these questions, and for spiritual comfort
- Prayer and accompaniment
- Accept individual, religious and cultural differences

Additional tools in berevement Counselling

In addition to the helping suggestions already given, the most useful techniques a care provider can use to help the grieving person are those that are basic to all helping and accompaniment. They are the techniques used in communication and listening which are discussed in the module on communication and counselling.

There are some additional techniques that can help If Used Appropriately, such as:

- The use of photos or other symbols of the deceased
- Writing down thoughts, poetry or messages
- Drawing pictures of memories or feelings – especially useful with children;
- Making a memory book or memory box
- Other techniques that the counsellor may have found useful

4. Needs of grieving children

- Children react differently to adults
- Small children see death as reversible and temporary
- Often children do not believe it could happen to them
- Children who see a lot of death may appear as though they do not feel deeply
- Children are often deprived of the attention of family members just when they need it most, as they are grieving too
- Children may think that the person who has died is still alive somewhere
- Helping a child remember the person who has died can be very healing – such as prayers, photos, memories, a story
- Children often grieve in small doses, on and off and often at the most unexpected moments
- Children should be allowed to grieve and express their feelings in their own way
- The person who has died was important to the stability of the child's life – it is natural that they might be angry
- Anger and grief may show in aggressive play, nightmares, irritability, or other ways
- Anger may be directed at other family members including adults or children
- A child might regress – acting younger that his or her age – demanding food, comfort, cuddling, attention more than usual
- A child may feel that he or she caused the death but not be able to say so

- A child may feel very guilty and blame him or herself
- A child may lost interest in friends, play, daily life and activities
- There may be loss of appetite , sleep, fear of being alone
- A child may imitate the person who has died
- A child may want to join the person who has died
- A child may start to do badly in school or refuse to go to school

Children need special attention when bereaved. They need unconditional love, support and acceptance of their feelings of loss. Simply the presence of a loving person who allows them to experience their grief in their own way may help them heal and travel through the long grieving process back to life. In more complicated grief it may be necessary to find experienced and professional help for the grieving child.

Session 21: Care for Carers

AIM: To equip health care workers with knowledge, skills and attitudes required in the provision of care for carers

TIME ALLOCATION: 45 minutes

LEARNING OBJECTIVES

By the end of this session participants should be able to:

Describe carers in palliative care

- Explain challenges faced by carers
- Discuss the causes of burnout
- Describe signs and symptoms of burnout
- Explain ways to prevent burnout

1. Definitions

A carer is an individual who has responsibility of caring for the sick in health facilities and community (CHBC policy and guide lines, 2005)

Formal carers: health care team (nurses, clinical officers, doctors, medical assistants)

Informal carers: These include HSAs, relatives, friends, neighbours and volunteers who care for the patient.

2. Benefits of being a caregiver

Informal	Formal Carers
• Feeling needed<br>• A sense of purpose and mission in life<br>• Positive feelings associated with loving and caring<br>• Feeling useful<br>• A sense of achievement<br>• Developing skills and knowledge<br>• Self-development<br>• Community recognition	• Opportunities for close communication with families<br>• Feeling useful Collegiality with other team members<br>Personal growth in facing mortality issues<br>• Learning opportunities<br>• Job satisfaction<br>• Professional status

3. Challenges faced by carers

Caring for a person with a chronic disease leaves little time for self-care. Carers often neglect their own nutrition, sleep requirements, exercise, or social needs. The physical tasks and consequences of care giving for family life act as chronic stressors that, coupled with inadequate self-preservation acts, lead to health problems and affective disorders amongst carers.

'Carer burden': refers to the physical, emotional, financial, and social problems associated with care-giving. The burden can be assessed in terms of objective or subjective impact. Objective impact refers to the extent to which care giving disrupts daily routines and social relationships and negatively affects resources. This includes missed days of work, family frictions, decreased social activities, and loss of income. The subjective impact includes becoming resentful about care giving and feeling trapped, exploited, nervous, or depressed about the relationship with the patient.

'Burnout': So-called 'burnout' is a process rather than a condition, and has various signs and symptoms…

Emotional exhaustion: This causes the carer to feel depleted, depressed, angry, or resentful.

Depersonalization: when carers lose their idealistic views or enthusiasm and become detached and negative towards their clients.

Loss of personal accomplishment and commitment to their profession: resulting in tardiness, absenteeism, and a feeling of relief when clients do not turn up.

Physical symptoms: such as susceptibility to cold, tension headaches, or chronic fatigue.

Causes of Burnout

Organizational	Individual	Interpersonal
▪ Lack of administrative support<br>▪ Lack of professional challenge<br>▪ Low salaries<br>▪ Difficulties in providing a service<br>▪ Intense repeated involvement with people<br>▪ Work overload<br>▪ No flexibility to meet human needs, such as sick leave or family responsibility leave	▪ Empathetic, altruistic, idealistic, over-committed to service to others<br>▪ Unassertiveness, inability to establish limits within helping relationship<br>▪ Younger, less experienced<br>▪ Single	▪ Need to be liked<br>▪ Cannot say 'no'<br>▪ Anxiety over competence<br>▪ High expectation of self<br>▪ Guilt if unable to help<br>▪

▪ Lack of training resulting in inadequate skills to perform their duties<br>▪ Lack of professional supervision	▪ Degree to which expectation of client and organization exceed actual expertise of caregiver	

Carer burnout has negative consequences for the carer, patient, and the health care system. Carers typically are highly motivated individuals who work in environments where they cannot 'afford' to burn out, or where they are just expected to cope. This can result in carers struggling for a long time with emotional exhaustion before recognizing they need help. As carers become more tired and detached, the sensitivity they show their patients declines and the quality of the services they provide suffers.

Home-based carers or community health workers are vulnerable because they are likeliest to have the least amount of training and often receive the least support. It is very important to build organizational and personal support into their working conditions. It is also important to remember that people do not necessarily feel 'called' to the role of carer, and are simply doing what is needed to care for their families. This lack of choices makes them even more vulnerable to the burdens of care giving.

4. Ways to prevent 'burnout'

Carers at all service levels should have the opportunity to examine the burden of care giving in their own lives. This creates awareness and, if combined with education on self-care and the signs and symptoms of burnout, the carer will be equipped to recognize danger signs early. A person can respond appropriately if able to name and understand an experience.

Learn to let go: Formal carers are generally very dedicated and involved in their work, and lose perspective on just how much one person can reasonably do.

Care for your health: Healthy lifestyles are important to reducing the effects of carer burden. This includes getting enough rest/sleep; exercise; not smoking; a balanced eating pattern; and avoiding 'stress-producing' foods such as alcohol, excess sugar and salt, and drinks with caffeine. It is most important to pay attention to the body's messages and attend to illnesses.

Get a life: The best way to ensure a long term involvement in care giving is to have a life outside of it. Neglecting friends, social occasions, family events, hobbies, or other pleasurable activities will reduce the carer's ability to cope with stress. For informal carers, outside employment might offer a respite from the burdens of care giving and the feeling of being trapped.

Learn to relax, not only rest: It is often difficult to stop worrying or escape the constant tension of care giving, even if one is ostensibly resting. 'Active rest' is relaxation, and can be done in many ways—including deep breathing exercises, imagery, hypnosis, progressive muscle relaxation, prayer, meditation, and massages. For some it might be calming music, dancing, or another activity that involves the quieter spiritual self and quietens the mind.

Admit when in trouble: It is often a matter of pride for health care professionals not to admit to distress or burnout. Organizations and older, more experienced professionals often frown upon succumbing to stress. These factors contribute to an expectation that carers will cope with impossible case loads without comment or complaint. It is important to remember that burnout is more common amongst younger, less experienced carers, and that there is no shame in admitting that one needs help.

Team work: Working with others in a team to share both the experiences and the work load reduces the risk of burnout.

Organizational Factors to Minimize Carer Burden

Some organizational factors could help to reduce the impact of caregiver burden on health care workers:

- Clear job descriptions and good referral systems
- Links with other service organizations and communities
- Safe, comfortable, and private work environments where possible
- Realistic work targets
- Restructured workloads that make them manageable and varied
- On-site health promotion programs
- Participation by staff in policy decisions that affect their work
- Regular time off and appropriate payment
- Built-in .flexibility in terms of back up in case of illness
- Scheduled memorial services, perhaps quarterly or yearly, for staff to take leave of the cases in their hearts and minds
- An environment that encourages self-care
- Open communication between team member

Session 22: Implementing Palliative Care in Malawi

AIM: To equip health care workers with knowledge, skills and attitudes on the establishment of palliative care services in their own setting

TIME ALLOCATION: 3 hours

LEARNING OBJECTIVES

By the end of this session participants should be able to:

- Describe WHO public health model for establishing palliative care Programmes
- Conduct a situation analysis in their setting
- **Develop an action plan for implementation of palliative car**

1. WHO public health model for palliative care

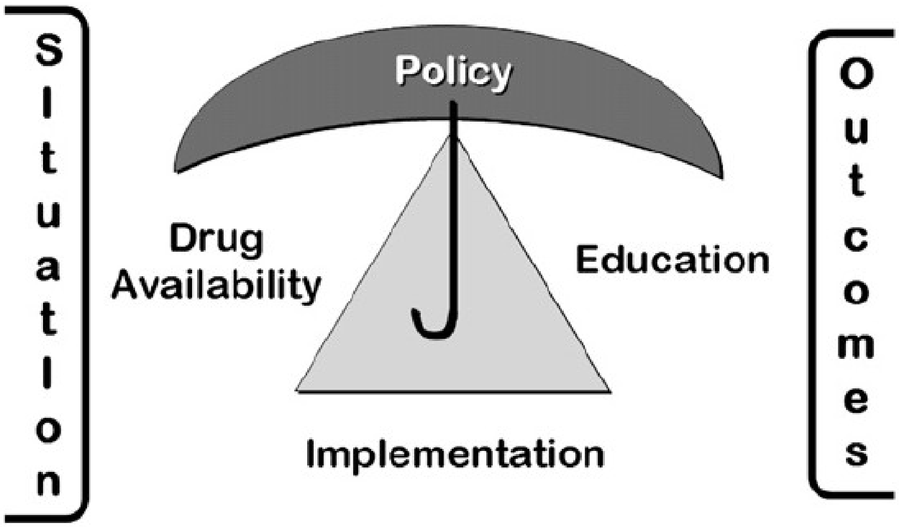

(Stjernsward,2007)
220072007)

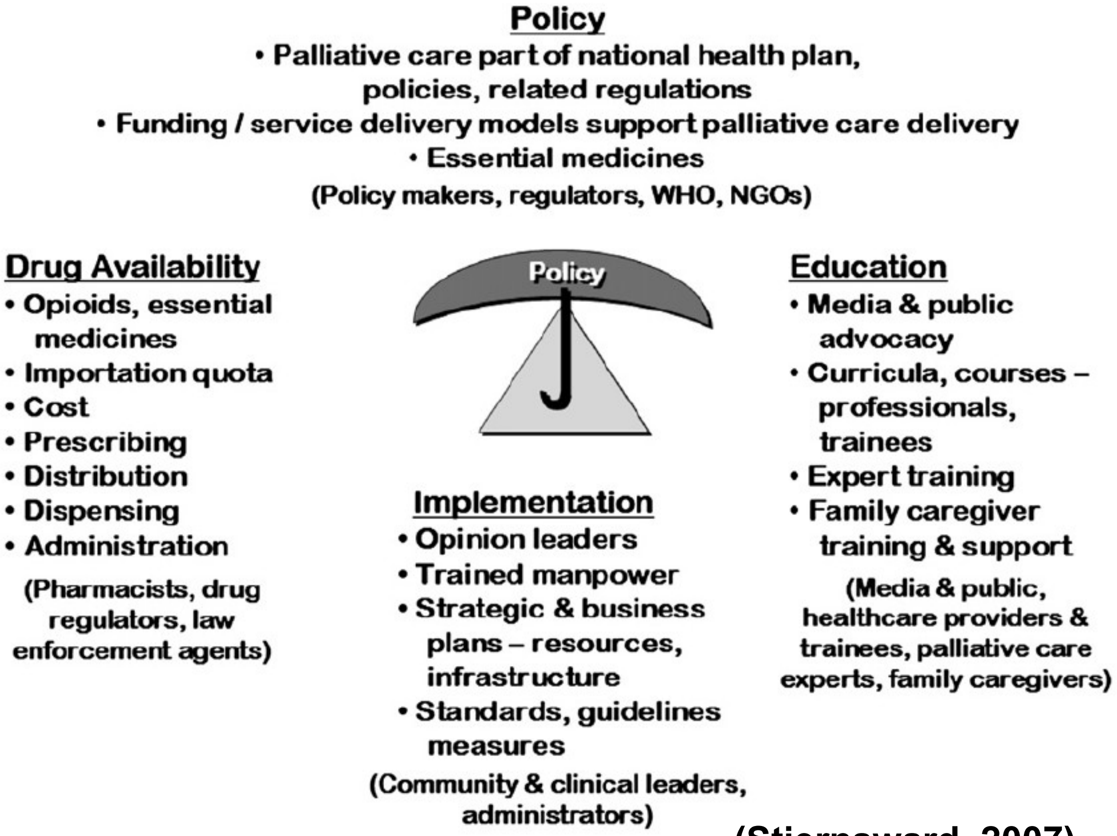

(Stjernsward, 2007)

2. Situation analysis

Who need palliative care? Where are they? How many?
What are their main problems?
What help are they getting at present?
What could be added to improve their care and make it holistic?

Points to consider when developing an Action Plan:

- Get the social demographic data on Cancer, HIV and AIDS and other life threatening condition in your area
- Conduct local needs assessment
- Assess your staff and administration willing ness to support you
- Choose model of care appropriate for your setting
- Establish hospital palliative care team

Example of an action plan

Activity	Responsible person	Time Frame	Required Resources
Selection of palliative care			

team			
Mobilization of resources			
Training			
Provision of palliative care services			
Monitoring and evaluation(supervision and scheduled meetings)			

Indicators

- Number of trained palliative care trainers according to gender and cadre

- Number of trained palliative care service providers according to cadre and gender(doctors,nurses,pharmacy technicians ,clinical officers, dental technicians etc)
- Number of trained palliative care health professionals providing palliative care services

- Number of palliative care sites
- Number of palliative care sites supervised

- Model of service delivery in use

- Number of palliative care patients seen as in patients in health facility according to gender
- Number of palliative care patients seen at home according to gender
- Number of drug day availability according to levels(step 1, step2 and step 3 drugs)
- Number of drug stock out according to levels.

- Availability of resources(palliative guideline, pain medications and essential supplies)

- Total Number of patients on program according to gender (children and adults to be reported separately)
- Total number of individuals provided with HIV related palliative care including TB and Cancer (according to gender)

Annex 1 :Program for 5 day introductory course in palliative care

Time	Day 1	Day 2	Day 3	Day 4	Day 5	Day 6
8:00-8:30		Registration, I Introduction, Welcoming remarks, Logistics & norms Course objectives	RECAP	RECAP	RECAP	RECAP
8:30-9:30		Pre-test course	Team work	Management of Skin Problems	Breaking Bad News	Grief & Bereavement
9:30-10:30		Introduction to palliative care	Ethics in Palliative Care	Common Diseases in Palliative		Care of Carers

				Care		
**10:30-11:00**	**REFRESHMENTS**					
11:00-12:00		When to start palliative care	Common Symptoms in Palliative Care	Common Diseases cont’	Wills & Inheritance	Post - Test
12:00-1pm		Holistic Approach		ART	Holistic Patient Assessment Group Work	Implementing Palliative Care (G/ work
1pm-2pm		**LUNCH**				
2pm-3pm	Participants’ Arrival	Models of Care	Pain Management	Children’s needs	Plenary	Plenary
**3pm-3:30**	**REFRESHMENTS**					
3:30-4:30	Facilitators’ meeting	Introduction to Communication skills	Use Of Morphine	Spiritual Support	Death & Dying	Course Evaluation
4:30-5pm		Facilitators’ meeting		Facilitators’ meeting		Feed back on Post- Test
5pm-5:15pm			Facilitators’ meeting		Facilitators’ meeting	Closure

Annex 2 : Palliative care holistic assessment form

Patient Name				Date seen
Sex	Age	marital status	physical address/location	Religion<br>Occupation
Next of kin /carer			History from- Patient { } Carer { } other (	Main source of income
Tribe			Patient insight into illness, Diagnosis, prognosis	
Referred from;<br>CHBC [ ]<br>Health center [ ]<br>OPD [ ]			Reason for Referral	Presenting complaints<br>History of illness and treatment to date

Hospital ward [ ]		

SPIRITUAL ASSESSMENT-	PSYSOCIAL HISTORY
Relationship with God<br>fears<br>What brings hope<br>Meaning of life	Effect of illness on family, age of children, cultural beliefs associated with illness, sexuality, main distress

Pain and symptom history Symptoms: 0 absent; + mild; ++ moderate +++ severe

**Symptom**	**0**	+	++	+++	**Comments (incl onset)**	**Symptom**	**0**	+	++	+++	**Comment**
Anorexia						Dry mouth					
Nausea						Skin Rash					
Vomiting						Skin Itch					
Dysphagia						Edema					
Painful Swallowing						Arthralgia (specify joints)					
Sore Mouth						Fatigue					
Dyspnoea						Confusion					
Cough						Drowsiness					
Headache						Diarrhea					
Paralysis						Other					

Pain chart

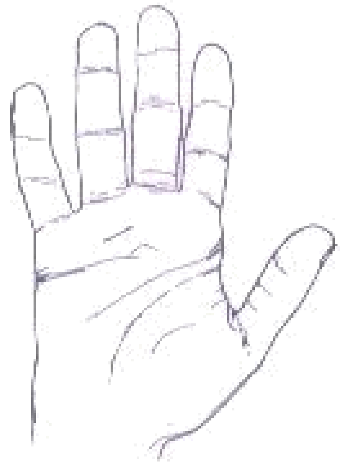

Plot each Pain Score into the graph below each time you see the patient please. Add N pains when they occur.

Visit		1st	2nd	3rd	4th	5th	6th	7th
**PAIN SCORE (Scale of 0-5)**	**5**							
	**4**							
	**3**							
	**2**							
	**1**							
	**0**							

<u>Keys (symbols) for the different types of pain</u>: If a new symbol is used, please indicate it below.

	Pain 1	Pain 2	Pain 3	Pain 4
Duration of pain				
Character/ description of pain				
Numerical Rating Scale (**0-5**)				
Periodicity (Constant /Intermittent)				
Precipitating Factors				
Relieving Factors				
Does pain affect sleep? Y/N				
Does pain affect mobility? Y/N				
Effect Of Current Medication – None, Partial, Complete Control				

	Pain 1	Pain 2	Pain 3	Pain 4
Duration of pain				
Character/ description of pain				
Numerical Rating Scale (**0-5**)				
Periodicity (Constant /Intermittent)				
Precipitating Factors				
Relieving Factors				
Does pain affect sleep? Y/N				
Does pain affect mobility? Y/N				
Effect Of Current Medication – None, Partial, Complete Control				

HIV status: +ve / -ve / not tested / disclosed	Diagnosis discussed with carers no / yes
Diagnosis discussed with patient no / yes	

Physical Examination<br>General condition:	Weight	
Ches		
Abdo		

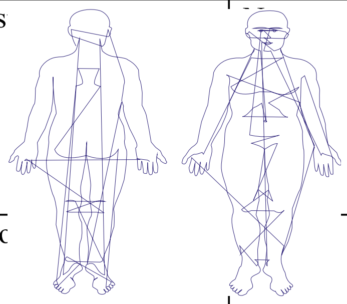

Palliative care Management Plan

Problem	Management Plan

Please list and number each problem (previous and new)<br><br>*No.*	*Problem*	*For each new and old problem note a brief management plan including non-pharmacological and pharmacological approaches. If problem no longer exists please explain why.*

Annex 3 : Palliative care patient register

**Patient Reg. no.**	**Date**	**Patient name**	**sex**	**age**	**Physical address**	**Referred from**	**diagnosis**	**Outcome Date (discharge/ referred/ died)**

Annex 4 :Palliative care service providers supervisory checklist

Name of Facility……………………………………………………………
Name of District………………………………………………..………….

Name of district palliative care coordinator: ……………………….……….
Name of facility palliative care coordinator: ……….…………………….
Contact address: ……………………………………………….………..…
TEL/CELL Number of facility coordinator.……………………………..…
Name of supervisor:..
Signature of supervisor:...
Position:...
Date:...

Rating Scale
5 – very good: out standing
4 – Good: moderate improvement necessary
3 – Fair: major improvement necessary
2 – Poor: total reorganization required
1 – Not available

Areas observed	Rating	Comments
**1. Client information and education**<br>▪ A roster for IEC available with topics and responsible persons<br>▪ A work plan available<br>▪ Diseases/condition education given		
**2. Interpersonal communication and counseling**<br>▪ Patients given an opportunity to talk to the service providers face to face<br>▪ Patients privacy observed<br>▪ Patient addressed by name		
**3. Patient care**<br>▪ Holistic patient history taken and recorded<br>▪ Correct steps followed in physical examination<br>▪ Patient told about findings<br>▪ Patient told about available services<br>▪ Patient helped to make informed decision<br>▪ Plan of care made together with patient plus family<br>▪ Appropriate Care given<br>▪ Documentation done<br>▪ Patient referred as necessary<br>▪ Follow up care planned		

4. **Home visit,**		
**a. Home assessment**<br>▪ General cleanliness of home plus surroundings(sanitation)<br>▪ Water availability<br>▪ Water source<br>▪ Food availability , utilization and security<br>**b. Patient assessment**<br>▪ Asks if any problems<br>▪ Checks patients' general condition (holistically)		

Mention problems encountered during implementation of palliative care services: ....
....
....
....

General comments / suggestions for improvement:
....
....
....
....
....

Annex 5 :Palliative Care Institutional Supervisory Check-List

1. Name of Facility
2. Name and titles of person (s) conducting the supervision
3. Date
4. Total number of trained palliative care service providers

Cadre	Female	Males
Drs		
COs		
SRNs		
EN/MMTs		
MAs		
others		

5. Number of patients seen per month
   New ☐ male ☐ Female ☐
   Subsequent ☐ Male ☐ Female ☐
   Total ☐ male ☐ Female ☐

6. How many days are clinics conducted at the hospital/site? ☐
7. How many days per week are home visits conducted? ☐

8. How many patients are referred per month by;
   a. volunteers ☐
   b.Institution to community. ☐
   c. Institution to institution ☐
9. Number of trained palliative care volunteers providing home based palliative care services in the community Female ☐ Male . ☐
10. Number of trained home based palliative care
11. List palliative care resources available at this facility

Item	Rating	Comments
**Resources**<br>Palliative Care guideline<br>Pain medications<br>Essential drugs and supplies<br><br>Transport for patient follow up		
**Record Keeping**<br>1 Patient register<br>2. Admission book		

3 Report files<br>[illegible]		

**Patient Care**	**Rating**	**Comments**
1 .Holistic history taken from patient		
2 .Pain managed according to WHO analgesic ladder<br>3 .Identification of symptoms done and managed according to developed plan<br>4. Other problems identified and patient referred to appropriate services,		

12. List problems being encountered ....
....
....

13. General comments / recommendations
....
....
....
....
....

\

Annex 6 :Referral form for health services in palliative care

Date:------------------------------<br>Name of the patient<br>---Tribe----------------------<br>sex------------Age-------------------- Religion----------------------Marital status<br>--------------------<br>Physical address of the<br>patient---<br>--<br>Next of Kin and relationship<br>---<br>Address of next of kin --<br>Name of carer ------------------------------------ Relationship to<br>patient------------------------------
Diagnosis(specify)--<br>-<br>Patient aware of diagnosis Y/ N<br>Carer aware of diagnosis Y /N
Main<br>problems--<br>---<br>---<br>--
Current treatment

--<br>--<br>---<br>Advice/counseling given on;<br>• Symptom management;---<br>• Care--<br>• Other--<br>--------------------------------------
Referred from: (Full Address)---Referred To:---<br>Reason for referral---<br>---

Referred by-----------------------Signature-----------------------Phone number---------------

(*Name and Designation*)

\

Annex 7 : WILL

_______________________________________ [Name of Testator]

I, _______________________________________ [Name of Testator] of ____________ [name of home village] Tradition authority _____________ in ______________________[name of District] in the republic of Malawi currently residing at ___________________[name of location] in the city of _______________[name of the city] ____________________[post address], being of sound and disposing mind and memory and over the age of eighteen (18) years do make, publish, and declare this to be my last Will, hereby expressly revoking all Wills and testamentary dispositions at any time heretofore made by me.

MARRIAGE AND CHILDREN

I am married to _______________________________________, and all references in this Will to my _________________ [husband or wife] are references to _________________ [him or her]. I have the following children:

Name: ____________________________________ Date of Birth: __________________

Name: ____________________________________ Date of Birth: __________________

Name: ____________________________________ Date of Birth: __________________

II. EXECUTOR

I appoint ___________________________________ [name and address] as Executor of this my Last Will and Testament and provide if this Executor is unable or unwilling to serve then I appoint ___________________________________ [name and address] as alternate Executor. My Executor shall be authorized to carry out all provisions of this Will and pay my just debts, obligations and funeral expenses.

II. GUARDIAN: In the event I shall die as the sole parent of minor children, then I appoint ___________________________________[name and address] as Guardian of said minor children. If this named Guardian is unable or unwilling to serve, then I appoint ___________________________________ as alternate Guardian.

IV. SIMULTANEOUS DEATH OF SPOUSE

In the event that my _________________ [wife or husband] shall die simultaneously with me or there is no direct evidence to establish that my _________________ [wife or husband] and I died other than simultaneously, I direct that I shall be deemed to have predeceased my _________________ [wife or husband], notwithstanding any provision of law to the contrary, and that the provisions of my Will shall be construed on such presumption.

V. SIMULTANEOUS DEATH OF BENEFICIARY

If any beneficiary of this Will, including any beneficiary of any trust established by this Will, other than my _________________ [wife or husband], shall die within 60 days of my death or prior to the distribution of my estate, I hereby declare that I shall be deemed to have survived such person.

VI. LEGACIES

(List down the property you own)

1. ____
2. ____ ___________________________
3. ____
4. ____

VII. BEQUESTS

I will, give and bequeath unto the persons named below, if he or she survives me, the Property described below:

Name:

Address:

Relationship:

Property:

Name:

Address:

Relationship:

Property:

Name:

Address:

Relationship:

Property:

If a named beneficiary to this will predeceases me, the bequest to such person shall lapse, and the property shall pass under the other provisions of this Will. If I do not possess or own any property listed above on the date of my death, the bequest of that property shall lapse.

VII. ALL REMAINING PROPERTY; RESIDUARY CLAUSE:

I give, devise, and bequeath all of the rest, residue, and remainder of my estate, of whatever kind and character, and wherever located, to my _________________ [state name of a person and relationship)], provided that _______ [he/she] survives me. If any of the said persons shall have predeceased me or fail to survive me for any other reasons, the gift to any of them shall fail then it shall be to the discretion of my trustees as to whether their issue where applicable shall get something.

VIII. ADDITIONAL POWERS OF THE EXECUTOR

My Executor shall have the following additional powers with respect to my estate, to be exercised from time to time at my Executor's discretion without further license or order of any court.

IX. WAIVER OF BOND, INVENTORY, ACCOUNTING, REPORTING AND APPROVAL:

My Executor and alternate Executor shall serve without any bond, and I hereby waive the necessity of preparing or filing any inventory, accounting, appraisal, reporting, approvals or final appraisement of my estate. I direct that no expert appraisal be made of my estate unless required by la

X.OPTIONAL PROVISIONS

I have placed my initials next to the provisions below that I adopt as part of this Will. Any unmarked provision is not adopted by me and is not a part of this Will.

If any beneficiary to this Will is indebted to me at the time of my death, and the beneficiary evidences this debt by a valid Promissory Note payable to me, then such person's portion of my estate shall be diminished by the amount of such debt.

____

Any and all debts of my estate shall first be paid from my residuary estate. Any debts on any real property bequeathed in this Will shall be assumed by the person to receive such real property and not paid by my Executor.

____

I desire to be buried in the ____________________________ cemetery in __________________ County

XII. SEVERABILITY AND SURVIVAL

If any part of this Will is declared invalid, illegal, or inoperative for any reason, it is my intent that the remaining parts shall be effective and fully operative, and that any Court so interpreting this Will and any provision in it construe in favour of survival.

IN WITNESS WHEREOF, I, ______________________________________ [Name of Testator], hereby set my hand to this last Will, on each page of which I have placed my initials, on this ________ day of ___________________, 20______ at ____, Malawi.

______________________________________ [Signature]

______________________________________ [Printed or typed name of Testator]

____

______________ [Address of Testator]

WITNESSES

The foregoing instrument, consisting of ________ pages, including this page, was signed in our presence by ______________________________________ [name of Testator] and declared by ________________ [him or her] to be ________________ [his or her] last Will. We, at the request and in the presence of ________________ [him or her] and

in the presence of each other, have subscribed our names below as witnesses. We declare that we are of sound mind and of the proper age to witness a will, that to the best of our knowledge the testator is of the age of majority, or is otherwise legally competent to make a will, and appears of sound mind and under no undue influence or constraint. Under penalty of perjury, we declare these statements are true and correct on this ________ day of ____________________, 20______ at ____, State of Malawi

_______________________________________ [Signature of Witness #1]
_______________________________________ [Printed or typed name of Witness]
_______________________________________ [Address of Witness]
____

_______________________________________ [Signature of Witness #2]
_______________________________________ [Printed or typed name of Witness]
_______________________________________ [Address of Witness]
_______________________________________ [Address of Witness]

Annex 8 : WHO Clinical Staging of HIV disease in Adults and Adolescents

CLINICAL STAGE 1

- Asymptomatic
- Persistent generalized lymphadenopathy

CLINICAL STAGE 2
Only eligible if TLC is less than 1200 cubic milli liters

- Moderate unexplained* weight loss (under 10% of presumed or measured body weight)*
- Recurrent respiratory tract infections (sinusitis, tonsillitis, otitis media, pharyngitis)
- Herpes zoster within the past 2 years
- Angular cheilitis
- Recurrent oral ulceration
- Papular pruritic eruptions
- Seborrhoeic dermatitis
- Fungal nail infections

CLINICAL STAGE 3

- Unexplained* severe weight loss (over 10% of presumed or measured body weight)**
- Unexplained* chronic diarrhoea for longer than one month

- Unexplained* persistent fever (intermittent or constant for longer than one month)
- Persistent oral candidiasis
- Oral hairy leukoplakia
- Pulmonary tuberculosis (active or within the past 2 years
- Severe bacterial infections (e.g. pneumonia, empyema, pyomyositis, bone or joint infection, meningitis, bacteraemia)
- Acute necrotizing ulcerative stomatitis, gingivitis or periodontitis
- Unexplained* anaemia (below 8 g/dl), neutropenia (below 0.5 x 109/l) and/or chronic thrombocytopenia (below 50 x 109 /l)

CLINICAL STAGE 4***

- HIV wasting syndrome
- Pneumocystis pneumonia
- Recurrent severe bacterial pneumonia
- Chronic herpes simplex infection (orolabial, genital or anorectal of more than
- One month's duration or visceral at any site)
- Oesophageal candidiasis (or candidiasis of trachea, bronchi or lungs)
- Extrapulmonary tuberculosis
- Kaposi sarcoma
- Cytomegalovirus infection (retinitis or infection of other organs)
- Central nervous system toxoplasmosis
- HIV encephalopathy
- Extrapulmonary cryptococcosis including meningitis
- Disseminated non-tuberculous mycobacteria infection
- Progressive multifocal leukoencephalopathy
- Chronic cryptosporidiosis
- Chronic isosporiasis
- Disseminated mycosis (extrapulmonary histoplasmosis, coccidiomycosis)
- Recurrent septicaemia (including non-typhoidal *Salmonella*)
- Lymphoma (cerebral or B cell non-Hodgkin)
- Invasive cervical carcinoma
- Atypical disseminated leishmaniasis
- Symptomatic HIV-associated nephropathy or HIV-associated cardiomyopathy

* Unexplained refers to where the condition is not explained by other conditions.
** Assessment of body weight among pregnant woman needs to consider the expected weight gain of pregnancy.

*** Some additional specific conditions can also be included in regional classifications, such as the reactivation of
American trypanosomiasis (meningoencephalitis and/or myocarditis) in the WHO Region of the Americas and penicilliosis in Asia.

Source: Revised WHO clinical staging and immunological classification of HIV and case definition of HIV for surveillance. 2006 (in press)

WHO Clinical Staging of HIV disease in Children

CLINICAL STAGE 1

- Asymptomatic
- Persistent generalized lymphadenopathy
- Not eligible for ARV therapy

CLINICAL STAGE 2

Only eligible if TLC/CD4 percentage isless than the threshhold

- Moderate unexplained* weight loss (under 10% of presumed or measured body weight)*
- Recurrent respiratory tract infections (sinusitis, tonsillitis, otitis media, pharyngitis)
- Herpes zoster within the past 2 years
- Angular cheilitis
- Recurrent oral ulceration
- Papular pruritic eruptions
- Seborrhoeic dermatitis
- Fungal nail infections

CLINICAL STAGE 3

- Moderate malnutrition
- TB lymphadenopathy
- Symptomatic LIP (lymphocystic interstitial pneumonia
- Chronic HIV lung disease
- HIV heart & Kidney disease

- Unexplained persistent diarrhea for more than 14 days
- Unexplained* severe weight loss (over 10% of presumed or measured body weight)**
- Unexplained* persistent fever (intermittent or constant for longer than one month)
- Persistent oral candidiasis
- Oral hairy leukoplakia
- Pulmonary tuberculosis (active or within the past 2 years
- Severe bacterial infections (e.g. pneumonia, empyema, pyomyositis, bone or joint infection, meningitis, bacteraemia)
- Acute necrotizing ulcerative stomatitis, gingivitis or periodontitis
- Unexplained* anaemia (below 8 g/dl), neutropenia (below 0.5 x 109/l) and/or chronic thrombocytopenia (below 50 x 109 /l)

CLINICAL STAGE 4***

- Severe malnutrition
- HIV wasting syndrome
- Pneumocystis pneumonia
- Recurrent severe bacterial pneumonia
- Chronic herpes simplex infection (orolabial, genital or anorectal of more than
- one month's duration or visceral at any site)
- Oesophageal candidiasis (or candidiasis of trachea, bronchi or lungs)
- Extrapulmonary tuberculosis
- Kaposi sarcoma
- Cytomegalovirus infection (retinitis or infection of other organs)
- Central nervous system toxoplasmosis
- HIV encephalopathy
- Extrapulmonary cryptococcosis including meningitis
- Disseminated non-tuberculous mycobacteria infection
- Progressive multifocal leukoencephalopathy
- Chronic cryptosporidiosis
- Chronic isosporiasis
- Disseminated mycosis (extrapulmonary histoplasmosis, coccidiomycosis)
- Recurrent septicaemia (including non-typhoidal *Salmonella*)
- Lymphoma (cerebral or B cell non-Hodgkin)
- Invasive cervical carcinoma
- Atypical disseminated leishmaniasis
- Symptomatic HIV-associated nephropathy or HIV-associated cardiomyopathy

* Unexplained refers to where the condition is not explained by other conditions.
** Assessment of body weight among pregnant woman needs to consider the expected weight gain of pregnancy.
*** Some additional specific conditions can also be included in regional classifications, such as the reactivation of
American trypanosomiasis (meningoencephalitis and/or myocarditis) in the WHO Region of the Americas and
penicilliosis in Asia.

Source: Revised WHO clinical staging and immunological classification of HIV and case definition of HIV for surveillance. 2006 (in press)

References

Basemera, B. (2004) *'Introduction to communication skills'* Hospice Africa Uganda.

Benson, T. F. and Merriman, A.. (2002) '*Palliative medicine pain and symptom control in the cancer/and AIDS patients in Uganda and other African Countries'* Third edition

C-SAFE Malawi Baseline Survey (September 2003) Executive Summary. TANGO International, Inc

Malawi Round 5 Final GFATM Proposal section 2.1.1.

Grand, E., Murray, S.A., Grant, A., and Brown J. 'A good death in rural Kenya? Listening to Meru patients and their families talk about care needs at end of life'

Hough, M. (1998) *Counseling skills and Theory,* Hodder and Stoughton

Keiza, K. (2002) Importance of will in matters of inheritance. *Palliative Care Manual for health professional.* (1st edition) Hospice Africa Uganda.

Kubler-Rose, E. (1969) *on death and dying* Tavistock, London

Makombe, S. Libamba, E. Mhango, o. Teck, J. Aberle-grasse, m. Hochgesang, E. schouten, A and Harries, A (2006) *Who is accessing antiretroviral therapy during national scale-up in Malawi?*' Transactions of the Royal Society of Tropical Medicine and Hygiene 100, 975-979

Merriman, A. (2002) '*Death and Dying'* (1st edition) Hospice Africa Uganda.
Merriman A. Opio D. and Kebirungi H. (2002) ***Palliative Care Training Manual for Health Care Professionals*** (1st edn) Hospice Africa Uganda.

Mpanga, S. and Lydia (2006) *Hospice Africa Uganda; Present and Future'* Presentation at Advocacy Drug Workshop, Uganda

Oxford textbook of pall medicine and oxford handbook of palliative care

Parry, E.H. 'Principles of medicine in Africa' Oxford University press

Quint, J. (1969) '*The nurse and the dying patient'* New York Macmillan

Robbins, RA (1995) '*Death anxiety, death competency and self – actualization in hospice volunteers'* Hospice J. 7: 29-35.

Makombe, S. ,Libamba, E. Muhango, E. Teck,O. A. , Aberle, J., Hochgesang, M., Erik, J. Schouten, A. Harries,D. and Higgisson , R. 'palliative care in Sub Sahara in Africa' *Lancet;* 2005: 365:1971-1977

Singer, P.A., Bowman, K.W. (2002) '*Quality care at the end of life'* BMJ 324: 1291-1292.

Shneidma, E. (1973) '*The death of man'* New York Quadrangle/ The New York.

Sliep, Y. (1997) '*Care Counseling for community Based Counselors'* Lilongwe: Action aid- Malawi.

Stedeford, A. (1994) F*acing death: patients, families and professionals'* (2nd edition) Sobell Publications, Edinburgh.

Twycross, R. (1995) *Introduction to palliative care*. Page 68

Weatherall,D.J., Ledingham , J.G.G. and Warrell,D.A. (1987) *Oxford Textbook Of Medicine,* Vol 1, 2nd Ed. Pg. 12.146

WHO (1996) WHO cancer pain relief. Geneva:
