# Supplementary material for: Assessing and Responding to Palliative Care Needs in Rural Sub-Saharan Africa: Results from a Model Intervention and Situation Analysis in Malawi
Source: PLoS One. 2014 Oct 14;9(10):e110457. doi: 10.1371/journal.pone.0110457 (PMC4197005; doi:10.1371/journal.pone.0110457)
Supplement: Appendix S5 — Malawi Guidelines for the Clinical Management of HIV in Children and Adults. (PDF) [file pone.0110457.s005.pdf]

Clinical Management of HIV in Children and Adults

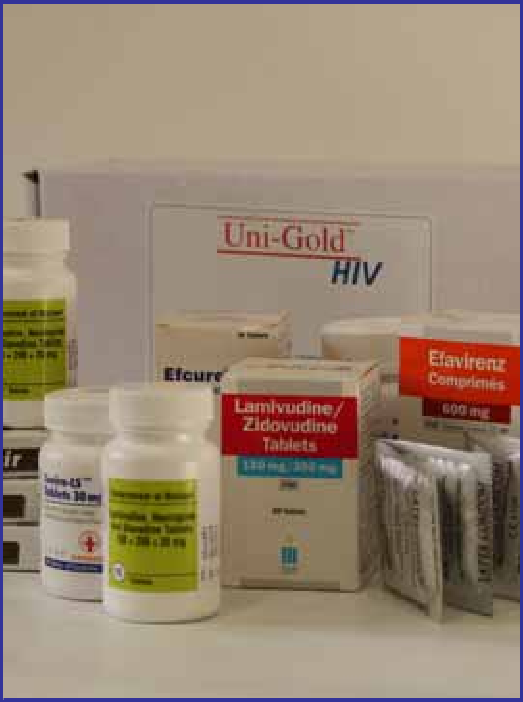

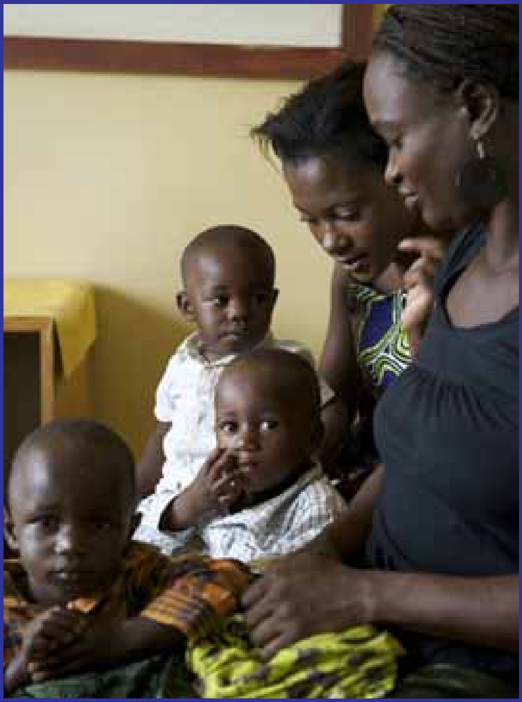

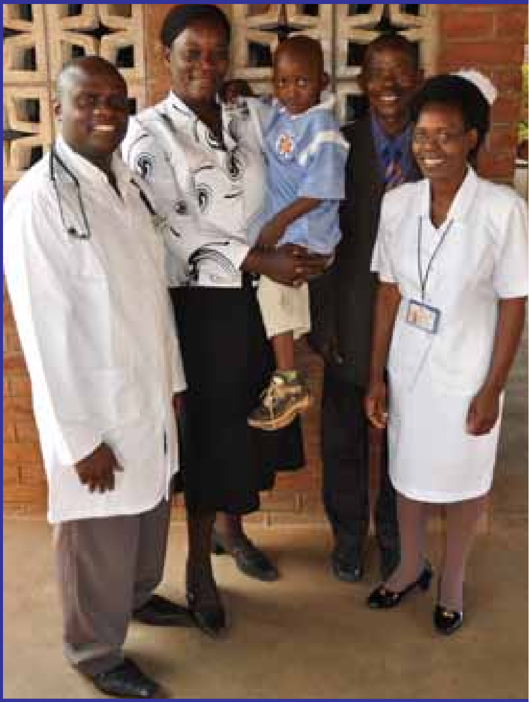

Malawi Integrated Guidelines for Providing HIV Services in:

- Antenatal Care
- Maternity Care
- Under 5 Clinics
- Family Planning Clinics
- Exposed Infant/Pre-ART Clinics
- ART Clinics

©2011 Ministry of Health, Malawi

Publications of the Ministry of Health enjoy copyright protection in accordance with the provisions of Protocol 2 of the Universal Copyright Convention. All rights reserved.

The Ministry of Health welcomes requests for permission to reproduce or translate its publications, in part or in full.

Applications and inquiries should be addressed to the Secretary for Health, P.O. Box 30377, Lilongwe 3, Malawi. We will be glad to provide the latest information on any changes made to the text, plans for new editions, and reprints and translations already available.

An electronic copy of this guideline is available on the website (www.hivunitmohmw.org) of the Department for HIV and AIDS of the Ministry of Health.

NOTE: The mention of certain manufacturers' products does not imply they are endorsed or recommended by the Ministry of Health in preference to others of a similar nature that are not mentioned.

Contents

Tables and Figures I

Acknowledgements II

Foreword III

1 Introduction 1

2 PMTCT strategy 2

3 Implementation Plan 3

4 How to use these guidelines 4

5 Integrating clinical HIV services 5

5.1 Follow-up for HIV exposed children 5
5.2 Pre-ART follow-up 5
5.3 The HIV Care Clinic (HCC) concept 6

6 Interventions 8

6.1 Provider initiated testing and counselling (PITC) 8
6.2 WHO clinical staging 12
6.3 Management of HIV-related diseases 14
6.4 Standard monitoring of HIV patients 18

6.4.1 Monitoring of nutritional status 18
6.4.2 Standard clinical monitoring checklist 22
6.4.3 CD4 monitoring for ART eligibility 27
6.4.4 Definition of ART eligibility 28

6.5 Preventive services for HIV patients 30

6.5.1 Provider initiated family planning (PIFP) 30
6.5.2 Prevention with positives (PwP) 31
6.5.3 Cotrimoxazole preventive therapy (CPT) 32
6.5.4 Isoniazid preventive therapy (IPT) 34
6.5.5 Insecticide treated bed nets (ITN) 35
6.5.6 Infant and child feeding counselling 36

6.6 Understanding ART regimens and formulations 37

6.6.1 Classification of individual ARVs 38
6.6.2 Choosing ART regimen, formulation and dosage 38
6.6.3 Choosing regimen and time of starting in special situations 44

6.7 ART prescription and dispensing 45

6.8 Starting ART ... **48**
6.8.1 Record keeping ... 49
6.8.2 Confirming HIV infection ... 49
6.8.3 Preparing the patient for ART ... 49
6.8.4 Baseline and routine lab investigations ... 50
6.8.5 Combining ART and TB treatment ... 51

6.9 Continuing ART ... **52**
6.9.1 Confirming adherence to appointment ... 52
6.9.2 Monitoring height and weight ... 52
6.9.3 Monitoring for new HIV-related diseases and drug side-effects ... 52
6.9.4 Indications for interrupting or stopping ART ... 52
6.9.5 Selecting regimen and formulation for continuation ... 53
6.9.6 Routine TB screening ... 53
6.9.7 Achieving optimal dose adherence ... 54
6.9.8 Keeping track of the number of months since ART initiation ... 54
6.9.9 Monitoring for treatment failure / HIV drug resistance ... 55
6.9.10 Updating follow-up outcome ... 57
6.9.11 Immune reconstitution inflammatory syndrome (IRIS) ... 62

6.10 Management of labour and delivery ... **63**
6.11 Newborn and postnatal care ... **63**
6.12 Initiating integrated mother/infant follow-up ... **63**
6.12.1 Dispensing infant NVP prophylaxis ... 64

6.13 Post exposure prophylaxis (PEP) ... **66**

7 Transition to the new PMTCT / ART regimens ... 70

8 Monitoring and Evaluation ... 72

8.1 Definitions ... **72**
8.2 Reporting of registration data ... **76**
8.3 Reporting of cohort outcomes ... **76**
8.4 Record keeping and filing ... **77**
8.4.1 Confidentiality of patient records ... 77
8.4.2 Use of clinic registers (ANC, Maternity, HCC, ART) ... 77
8.4.3 Use of patient cards ... 78

8.5 Ensuring adequate data quality ... **79**

Tables and Figures

Tables

Table 1: Integrated provision and scheduling and of clinical HIV services 7

Table 2: Schedule of HIV testing in children in HCC or ART follow-up: Choice of type of test, interpretation of results and follow-up management 11

Table 3: WHO clinical staging for children and adults with confirmed HIV infection and definition of presumed severe HIV disease for infants 13

Table 4: Standard checklist for clinical monitoring of HIV exposed children, pre-ART and ART patients 22

Table 5: Detailed clinical monitoring list for HIV exposed children, pre-ART and ART patients 23

Table 6: Dosage of Cotrimoxazole Preventive Therapy 33

Table 7: Dosage for Isoniazid Preventive Therapy 35

Table 8: Classification of ARVs 38

Table 9: Standard ART 1st line (Regimen 1 - 6) and 2nd Line (Regimen 7 - 9) 42

Table 10: Standard pack sizes and dosing of Paediatric and Adult formulations used in standard 1st and 2nd line ART regimens 43

Table 11: Choosing ART regimen and timing of initiation in special situations 44

Table 12: Quantity of ARVs to be supplied for visit intervals from 2-12 weeks for different daily doses 47

Table 13: Relevant interactions between ARVs and TB drugs 51

Table 14: Symptom-based identification and management of ARV side-effects 58

Table 15: Dosing of NVP syrup for infant prophylaxis 65

Table 16: Classification of risk of transmission after exposure to HIV 67

Table 17: Post exposure prophylaxis regimens and dosage 68

Table 18: Regimens and dose for emergency contraception 68

Table 19: Dosing of standard presumptive STI treatment after sexual exposure 69

Table 20: Overview of M&E systems for integrated HIV program reporting 75

Figures

Figure 1: Flowchart for routine ascertainment of HIV exposure / infection status in children under 24 months 10

Figure 2: Weight for Height classification of wasting / malnutrition for children 0 - 14 years 20

Figure 3: BMI classification of malnutrition for non-pregnant adults 15 years and above 21

Figure 4: Flowchart for classification of *Reason for Starting ART* (shaded boxes) based on the hierarchy of ART eligibility criteria 29

Figure 5: ART regimen transition for children and adults in Phase 1 (July 2011) and Phase 2 (to be announced by MOH circular) 71

// Acknowledgements

The Department for HIV and AIDS of the Ministry of Health gratefully acknowledges the contributions of the writing committee, under the chairmanship of Dr Frank Chimbwandira, Director of HIV Department:

Mrs Laura Anderson	I-TECH
Dr Belete Assefa	Dignitas International
Dr Beth Barr	Centers for Disease Control and Prevention
Dr Ann Akesson	Médecins Sans Frontières (Belgium)
Mrs Jane Banda	MCHIP- JHPIEGO
Dr Chris Buck	Baylor College of Medicine
Ms Pepukai Chikukwa	UNAIDS Malawi
Dr Zengani Chirwa	Department for HIV and AIDS, MOH
Dr Carrie Cox	Baylor College of Medicine
Mr Michael Eliya	Department for HIV and AIDS, MOH
Dr Carrie Golito	Baylor College of Medicine
Prof Anthony Harries	IUATLD
Dr Mina Hosseinipour	University of North Carolina
Dr Andreas Jahn	Department for HIV and AIDS, MOH
Mr Haswell Jere	DREAM Project Malawi
Mrs Effie Kamigoghe	CRS- IMPACT
Mr Henry Kanyerere	National TB Programme, MOH
Dr Giuseppe Liotta	DREAM Project Malawi
Dr Alice Maida	I-TECH Malawi
Dr Mwai Makoka	National AIDS Commission
Mr Simon Makombe	Department for HIV and AIDS, MOH
Mrs Eustice Mhango	Department for HIV and AIDS, MOH
Mrs Dalitso Midiyani	Department for HIV and AIDS, MOH
Mr Maxime Molisho	Clinton Health Access Initiative
Dr Agnes Moses	University of North Carolina
Mr Martin Msukwa	Clinton Health Access Initiative
Mr Joseph Njala	Department for HIV and AIDS, MOH
Mr Lucius Ng'omang'oma	Department for HIV and AIDS, MOH
Dr Kondwani Ng'oma	UNICEF Malawi
Prof Joep van Oosterhout	College of Medicine, University of Malawi
Dr Sam Phiri	Lighthouse Trust
Ms Antonia Powell	CRS-IMPACT
Dr Esther Ratsma	Dignitas
Dr Erik Schouten	MSH
Mr Lyson Tenthani	Department for HIV and AIDS, MOH
Dr Abdoulaye Sarr	Centers for Disease Control and Prevention
Dr Tom Warne	Centers for Disease Control and Prevention
Dr Aida Yemane Berhan	EGPAF

Foreword

This *1st Edition* of the *Malawi Guidelines for Clinical Management of HIV in Children and Adults* will be implemented from July 2011. It replaces all previous editions of the Malawi Antiretroviral therapy (ART) and Prevention of Mother to Child Transmission (PMTCT) guidelines.

These guidelines are written for medical doctors, clinical officers, medical assistants, nurses, midwives, health surveillance assistants (HSAs) and medical records clerks who are working in public and private sector health facilities in Malawi. The document is designed to be a practical guide for implementation of integrated HIV Services.

The guidelines have been compiled by the joint Technical Working Groups for PMTCT, ART, HTC and Paediatric HIV under the leadership of the Department for HIV and AIDS of the Ministry of Health. The guidelines are based on ***Malawi's Revised Policy for PMTCT and ART*** which was endorsed by the Ministry of Health in June 2010 and which was prompted by the release of the 2010 Revision of the World Health Organisation (WHO) PMTCT and ART Guidelines.

The protocols and policies presented in this document are adapted for health services in Malawi and follow a ***public health approach,*** aiming to provide the best possible services for the largest possible number of persons in need of these services.

This document defines the framework for Malawi's National HIV Programs. Considering public health benefits and risks, as well as funding and resource implications, **deviations from these guidelines are not supported by the Ministry of Health**.

The 2nd Edition of these guidelines is scheduled for release in 2013. Any updates or amendments to protocols and policies that are to be implemented between July 2010 and the release of the 2nd Edition of the guidelines will be communicated through an official MOH circular.

Acronyms and Abbreviations

**3TC**	Lamivudine
**AAFB**	Acid alcohol fast bacilli
**ABC**	Abacavir
**ANC**	Antenatal care
**ARM**	Artificial rupture of membranes
**ART**	Antiretroviral therapy
**ARVs**	Antiretroviral drugs
**AZT**	Zidovudine
**BCG**	Bacille Calmette-Guérin
**Benzyl pen**	Benzyl penicillin
**BF**	Breastfeeding
**BMI**	Body mass index
**CO**	Clinical Officer
**CPT**	Cotrimoxazole preventive therapy
**CSF**	Cerebrospinal fluid
**CTX**	Cotrimoxazole
**CXR**	Chest X-ray
**d4T**	Stavudine
**DBS**	Dried blood spot
**ddI**	Didanosine
**dl**	decilitre
**DNA-PCR**	Deoxyribonucleic acid polymerase chain reaction
**E**	Ethambutol
**EFV**	Efavirenz
**EHP**	Essential health package
**EPI**	Extended Programme on Immunization
**EPTB**	Extra-pulmonary tuberculosis
**FDC**	Fixed dose combination
**FP**	Family planning
**GIT**	Gastrointestinal tract
**H**	Isoniazid
**Hb**	Haemoglobin
**HCC**	HIV Care Clinic
**HIV**	Human immunodeficiency virus
**HTC**	HIV testing and counselling
**IEC**	Information, Education and Communication
**IM**	Intramuscular
**INH**	Isoniazid
**IPT**	Isoniazid preventive treatment
**IRIS**	Immune reconstitution syndrome

Acronyms and Abbreviations

**ITN**	Insecticide treated net
**IV**	Intravenous
**KS**	Kaposi sarcoma
**LFT**	Liver function test
**LPV/r**	Lopinavir/ ritonavir
**M&E**	Monitoring and evaluation
**MA**	Medical Assistant
**MCH**	Maternal and child health
**MDR-TB**	Multi-drug resistant tuberculosis
**MOH**	Ministry of Health
**MUAC**	Mid-upper arm circumference
**NS**	Non-standard ART regimen
**NVP**	Nevirapine
**OPD**	Out patient department
**ORS**	Oral rehydration solution
**PCP**	Pneumocycstis carinii (jiroveci)pneumonia
**PCR**	Polymerase chain reaction
**PEP**	Post-exposure prophylaxis
**PIFP**	Provider initiated family planning
**PITC**	Provider initiated testing and counselling
**PMTCT**	Prevention of mother to child transmission
**PO**	Per os
**PSHD**	Presumed severe HIV disease
**PTB**	Pulmonary tuberculosis
**PwP**	Prevention with Positives
**R**	Rifampicin
**S**	Streptomycin
**sdNVP**	Single dose nevirapine
**SP**	Sulphadoxine/ pyrimethamine
**STI**	Sexually Transmitted Infections
**TBT**	Anti-tuberculosis treatment
**TDF**	Tenofovir
**TF**	Therapeutic feeding
**VIA**	Acetic acid visualization (of the cervix)
**VL**	Viral load
**WHO**	World Health Organization
**Z**	Pyrazinamide
**ZDV**	Zidovudine

1 Introduction

In June 2010, the WHO released new recommendations for ART and PMTCT in resource limited countries. Recommendations were based on current research evidence and aimed at increasing access to quality ART and PMTCT services.

WHO recommended:

- Earlier initiation of ART to slow disease progression, increase survival and reduce HIV transmission.
- Phasing out of stavudine (d4T) based regimens to reduce long term side-effects.
- Use of more efficacious PMTCT regimens, starting at 14 weeks gestation and continuing through labour and breastfeeding to further reduce transmission and improve maternal and child health outcomes.

The above recommendations have prompted the development of this 1st Edition of the ***Malawi Guidelines for Clinical Management of HIV in Children and Adults*** that fully integrate protocols for:

- PMTCT
- Follow-up of HIV exposed infants
- Pre-ART follow-up for children and adults
- ART

The rapid scale up of the ART programme in Malawi has been achieved through the public health approach, which seeks to provide the best possible services for the largest possible number of persons in need of these services. The protocols in the new guidelines are an adaptation of the 2010 WHO recommendations to the Malawi programs, drawing on experience from the roll out of PMTCT and ART.

2 PMTCT strategy

Prong 1
- Primary prevention of HIV infection in parents

Prong 2
- Prevention of unintended pregnancies among HIV positive women

Prong 3
- Start of lifelong ART for HIV infected pregnant and breastfeeding women, regardless of CD4 count and/or clinical stage ('Option B+')
- Provision of nevirapine (NVP) prophylaxis for babies born to HIV infected mothers up to age 6 weeks
- Safe obstetric practices

Prong 4
- Provision of care, treatment and support for HIV-infected women, their children, and their families

Rationale for lifelong ART for pregnant and breastfeeding women (Option B+)

- **Increased access to ART:**
  - Because a positive HIV Antibody rapid test result in a pregnant woman is the only eligibility criterion for ART, antenatal clinics serve as an ideal entry point for ART.
  - High ANC attendance rates (91% for Q2 2010[1]) and availability of HIV rapid testing at all ANC sites enables a high ART coverage of HIV infected women.
- **Reduction in post-partum mortality rates in HIV infected women:**
  - High mortality rates have been documented in post-partum women with high CD4 counts (>350 cells/mm^3 in pregnancy) who were not on ART [2,3]
- **Reduction of HIV transmission:** Maternal ART reduces viral load (VL) which:
  - Provides optimal protection during pregnancy, delivery and for subsequent pregnancies, especially given high fertility rates in Malawi
  - Enables safe breastfeeding and avoids the need for extended infant HIV prophylaxis
  - Reduces HIV transmission to sexual partners, especially for discordant couples

---

[1] HIV Programme Quarterly Report Q2 2010

[2] Hargrove JW, Humphrey JH. Mortality among HIV-positive postpartum women with high CD4 cell counts in Zimbabwe. AIDS 2010; 24(3):F11-4.

[3] Van Lettow M, Landes M, Bedell R, et al. Uptake and outcomes of a prevention-of mother-to-child transmission (PMTCT) program for 387 mother-child pairs in Zomba district, Malawi. XVIII International AIDS Conference 2010, Vienna

3 Implementation Plan

Implementation of the 2011 guidelines will be conducted in 2 phases.

Phase 1

Phase 1 will begin in July 2011 with the following new protocols:

1. Standard clinical follow-up for children of HIV infected mothers up to the age of 2 years
2. Extended nevirapine prophylaxis for infants of HIV infected mothers up to age 6 weeks
3. Standard clinical follow-up and Isoniazid Preventive Therapy (IPT) for HIV infected children and adults who are not yet eligible for ART
4. Mandatory confirmation of HIV infection / antibodies for all patients immediately before ART initiation
5. Use of paediatric ARV formulations for all children under 25kg
6. New standard first line regimen for children under 15 years of age (zidovudine / lamivudine / nevirapine)
7. ART initiation for patients in WHO stage 1 and 2 from a CD4 count below 350 cells/mm^3
8. Universal initiation of life-long ART for children with confirmed HIV infection under the age of 2 years
9. Initiation of life-long ART for children aged 2-4 years who are in WHO stage 1 and 2 with a CD4 count below 750 cells/mm^3 (CD4 % is no longer required)
10. Universal initiation of life-long ART for HIV-infected pregnant and breastfeeding women
11. Provider initiated provision of Depo-Provera and condoms in pre-ART and ART clinics
12. Use of fixed-dose combination of tenofovir / lamivudine / efavirenz for:
    - ART initiation of pregnant and breastfeeding women
    - ART initiation of patients on TB treatment
    - Substitution of stavudine- or zidovudine-based first line regimens in patients with confirmed lipodystrophy

Phase 2

Phase 2 is planned for 2012-13 following successful implementation of Phase 1 and additional resource mobilization. MOH will issue a specific circular to all health facilities to announce the launch of Phase 2:

13. Use of fixed-dose combination of tenofovir / lamivudine / efavirenz for ART initiation of all patients from age 15 years
14. Scale-up of routine scheduled viral load monitoring for all patients on ART

4 How to use these guidelines

These guidelines have been developed to standardise clinical management of HIV positive patients and of HIV exposed children using an integrated approach. They also incorporate relevant protocols from other national guidelines (TB, IPT, FP, STI, Reproductive health).

Most clinical interventions for HIV patients are provided in different service delivery settings and these guidelines provide **standardised simplified protocols** for each intervention that will facilitate the job of the health workers and improve the standard of care for patients.

Key Facts for Providers and Patients

- The most important information and key instructions are presented in a purple box at the beginning of each section. It is appropriate and helpful to share this information with patients during Information, Education, and Communication (IEC) sessions, and in individual counselling.

Short bullet points and 'plain language' are used throughout this document to make the information as clear and as easily accessible as possible.

The standard package of clinical HIV interventions

Chapter 5 on page 5 shows which of the **clinical HIV interventions** should be provided in each of the regular service delivery points of the health system. It also defines the standard package of services and explains which interventions are appropriate for which patient groups and when to deliver them.

Protocols for how to deliver clinical HIV interventions

Chapter 6 on page 8 explains in detail how to deliver each of the HIV interventions. The protocols and directions are the same for *all* service delivery settings. This chapter also contains several checklists, tables and flow charts. These are distributed by MOH to all health facilities as laminated job aids for use in the consultation room.

Transition from previous PMTCT and ART protocols

Chapter 7 on page 70 gives detailed instructions on how to manage care for patients through the transition from previous treatment protocols to the new protocols. This includes both new patients and patients who have already begun treatment and are in follow-up.

5 Integrating clinical HIV services

Clinical HIV services are an integral part of the essential health package (EHP). This section shows the **standard schedule** for the **minimum package of** clinical HIV interventions to be delivered within the established service points. **Table 1** on page 7 outlines the HIV interventions to be offered at various service delivery points. Refer to the page number for details on how to deliver the specific intervention.

5.1 Follow-up for HIV exposed children

- Actively screen all children under 24 months for HIV exposure (see Section 6.1 on page 9)
- Enrol all children born to and/or breastfeeding from HIV infected mothers as soon as possible.
- Do one DNA-PCR test as soon as possible from age 6 weeks. This will detect perinatal HIV infection and allow for ART initiation as early as possible.
- Visit schedule:
  - Monthly visits until age 6 months. Align with EPI vaccination visits.
  - 3 monthly visits from age 6 to at least 24 months. Most children are breastfed beyond 18 months and actual weaning can be difficult to confirm, so assume that all children are receiving breast milk until at least 18 months.
  - Schedule more frequent visits if the child is not doing well.
  - Discharge only after obtaining a negative HIV rapid test at least 6 weeks after stopping of breast feeding.

5.2 Pre-ART follow-up

- Enrol all children and adults who were found HIV positive but not yet eligible for ART in pre-ART follow-up
- Visit schedule:
  - If IPT is started: review patient 1 month after starting IPT and then 2 months later (at month 3).
  - Give 3 monthly appointments thereafter
  - If IPT is not started: give 3 monthly appointments throughout
  - Schedule more frequent visits if the patient is not doing well.
- Keep the patient in pre-ART follow-up until he starts ART

5.3 The HIV Care Clinic (HCC) concept

- ***HCC*** is a further integration of services designed to facilitate access for clinical monitoring, preventive services and ART for family members affected by HIV. For this reason, the following services should be provided together at the same time in the same clinic:
    - Follow-up for HIV exposed children
    - pre-ART follow-up for children and adults
    - ART
- Family appointments can be given to encourage family members to attend together for HIV services.
- Family members can be seen in the consultation room at the same time or seen individually if there are sensitive issues to discuss.

Table 1: Integrated provision and scheduling and of clinical HIV services

Interventions that are provided only under special circumstances are marked with brackets (●)

HIV Service	Page	Schedule	OPD	In-Patients	Fam. Plan. Clin.	ANC	Maternity	Postnatal Clinic	U5 Clinic	Exp. Infant FUP	Pre-ART FUP	ART Clinic	TB Clinic
										HIV Care Clinic			
Provider initiated testing and counselling (PITC)	*8*	Ascertain current status at each visit	●	●	●	●	●	●	●	●	(●)	(●)	●
WHO clinical staging	*12*	When first found HIV+, then 3-monthly in pre-ART	●	●		●	●	●		●	●	●	●
Management of HIV-related diseases	*14*	When diagnosed	●	●		(●)	(●)			●	●	●	●
Standard monitoring of HIV patients	*18*	At every visit								●	●	●	●
CD4 monitoring for ART eligibility	*27*	6-monthly if last CD4 ≥500, 3-monthly if 350-499									●		
Provider initiated family planning (PIFP)	*30*	At every scheduled visit									●	●	
Prevention with positives (PwP)	*31*	At every visit									●	●	
Cotrimoxazole preventive therapy (CPT)	*32*	At every scheduled visit				●	●			●	●	●	●
Isoniazid preventive therapy (IPT)	*34*	Dispense for 1, 2 and then 3 monthly thereafter									●		
Insecticide treated bed nets (ITN)	*35*	Dispense 1 ITN every 24 months				●			●	●	●	●	
Infant and child feeding counselling	*36*	At every visit	●			●	●		●	●	●	●	
Starting ART	*48*	Within 7 days of being found eligible for ART				●	●					●	●
Continuing ART	*52*	monthly for the 1st 6 months; 3 monthly thereafter				●						●	●
Management of labour and delivery	*63*	At admission					●						
Newborn and postnatal care	*63*	After delivery					●	●					
Initiating integrated mother/infant follow-up	*63*	At first opportunity when known HIV+					●	●	●				
Dispensing infant NVP prophylaxis	*64*	At first opportunity when known HIV+				●	●	●	●	(●)			
Post exposure prophylaxis (PEP)	*66*	As soon as possible after risk exposure	●				●						

6 Interventions

6.1 Provider initiated testing and counselling (PITC)

Key Facts for Providers and Patients

- HIV testing and counselling (HTC) is offered in many different forms (client- or provider-initiated, community based, etc.) Models of delivering HTC include: stand-alone, integrated, outreach, mobile, home-based, door-to-door
- Ascertain HIV status for all patients attending health services (PITC)
- Encourage patients to attend HTC with their sexual partner and ensure that all children of HIV infected parents are tested.
- Remind patients during pre-test education (group or individual) that they can decline HIV-testing without any consequences
- From July 2011, all patients need a confirmatory HIV test to rule out any possibility of mix-up of test results or fraudulent access to ART (also see **Section 6.8.2** on **page 49**):
    - Either at enrolment into pre-ART follow-up,
    - Or before starting ART if the test to confirm was not done in pre-ART.
    - Children under 12 months starting ART with a positive DNA-PCR do not need another confirmatory test before starting ART, but all need a confirmatory rapid antibody test at age 12 and 24 months (see below).
- Enrol all children and adults with confirmed HIV infection for ART or for HIV care to ensure they can start ART as soon as they become eligible
- Enrol all children born to and/or breastfeeding from HIV infected mothers (*'HIV exposed children'*) in the HIV Care Clinic and follow to at least age 24 months or longer if breastfeeding continues
- From age 12 months, over 95% of children with a positive rapid test are confirmed HIV infected. Therefore, rapid testing should be used to determine universal eligibility for ART for children aged between 12 and 24 months.
- Examine all children under 12 months of age with confirmed HIV antibodies for clinical conditions that constitute *Presumed Severe HIV Disease (PSHD, see* **Table 3** *on* **page 13***)*. All of these need to start ART without delay.
- All children under 24 months in exposed infant follow-up and those who have started ART need confirmatory rapid antibody tests:
    - at age 12 months
    - at age 24 months
- Use DNA-PCR as 'tie-breaker' for a child who was started on ART below 24 month of age and whose confirmatory rapid antibody test at age 12 or 24 months is negative. Stop ART only if DNA-PCR comes back negative, too.

Routine ascertainment of HIV infection status for children and adults

- Ask every client at every visit about the most recent HIV test and review their health passport for previous HIV test results.
- Offer HIV testing to all patients attending health facilities for any reason, if:
  - never tested
  - tested negative more than 3 months ago
  - claims to have been tested any time in the past, but without documentation (Being on ART counts as documented evidence)
- Routinely document HIV test results on page 6 of the patient's health passport unless the patient declines. For in-patients, also document test result in in-patient notes.

Routine ascertainment of HIV exposure status for children under 24 months

- Routinely ascertain the mother's HIV status for all children under 24 months of age seen at the U1 / U5 clinic, regardless of whether the child is healthy or sick:
  - Review mother's health passport (page 6) for the latest HIV test result
- Initiate a new HIV rapid test:
  - For the mother:
    - If she was not tested at least once during pregnancy or delivery
  - For the child:
    - If the mother is not available / has died
    - If the child is sick, even if the mother was tested negative during pregnancy or delivery. This is to rule out new HIV infection in the child.
- **Figure 1** on page 10 shows the conditions for testing of mother and/or child and the actions to be taken.

Figure 1: Flowchart for routine ascertainment of HIV exposure / infection status in children under 24 months

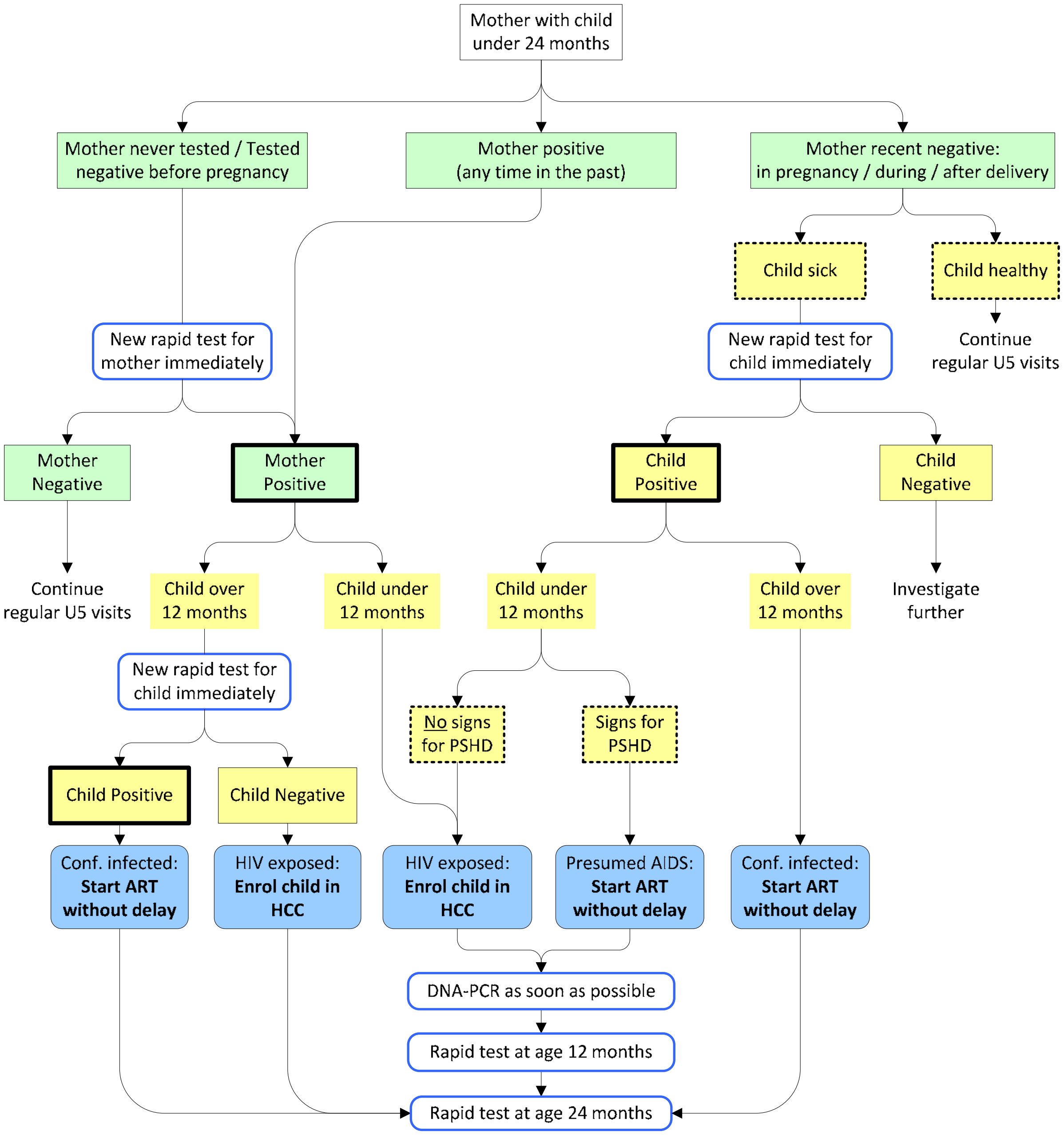

Table 2 on page 11 shows the routine testing schedule for children under 2 years of age, the selection of the type of HIV test (DNA-PCR or rapid antibody test) depending on the child's age and the correct interpretation and action depending on the test result.

Table 2: Schedule of HIV testing in children in HCC or ART follow-up: Choice of type of test, interpretation of results and follow-up management

Age (months)	Test	Schedule	Result	Interpretation	Action
**Under 12**	DNA-PCR (if available)	First opportunity from age 6 weeks	Negative	Not infected, but at risk of infection if breastfeeding	Continue HCC<br>Do rapid test at age 12 months
			Positive	HIV infected	Start ART<br>Confirm with rapid test at 12 and 24 months
	Rapid antibody	Immediately if signs of PSHD identified<br>***OR***<br>If mother's HIV status cannot be ascertained	Negative	Not infected, but at risk of infection if breastfeeding	Treat condition<br>Continue HCC<br>Repeat rapid test at age 12 and 24 months
			Positive	Possibly HIV infected if no PSHD symptoms	Enrol in HCC<br>Do DNA-PCR at first opportunity
				Likely AIDS if symptoms for PSHD	Start ART<br>Confirm with rapid test at 12 and 24 months
**12 to under 24**	Rapid antibody	Age 12 months<br>***OR***<br>If mother's HIV status cannot be ascertained	Negative	Not infected, but at risk of infection if breastfeeding	Continue HCC, repeat rapid test at age 24 m.<br>If on ART don't stop unless confirmed by negative DNA-PCR
			Positive	HIV Infected	Start ART<br>Confirm with rapid test at 24 months
**24 and above**	Rapid antibody	From age 24 months but ensure that BF stopped at least 6wks ago	Negative	Not infected	Discharge child from HCC
			Positive	HIV Infected	If already on ART, continue ART. Otherwise continue pre –ART follow up in HCC.

6.2 WHO clinical staging

Key Facts for Providers and Patients

- Untreated HIV infection leads to a gradual destruction of the immune system.
- Different HIV-related diseases tend to appear at different levels of immune suppression
- HIV-related diseases are grouped into 4 WHO clinical stages that correlate with disease progression and prognosis of survival:
    - Stage 1: Asymptomatic
    - Stage 2: Mild
    - Stage 3: Advanced
    - Stage 4: Severe
- Many patients have several HIV-related diseases from different stages
    - List all conditions on the *ART Patient Card*
    - Note that the most severe condition determines the WHO clinical stage
- Most WHO stage defining conditions apply to all ages, but some are only for children under 15 years and others are only for adults
- Patients in WHO stage 3 or 4 are always eligible to start ART. Other conditions apply to patients in stage 1 or 2 (see Section 6.4.4 on Page 28)
- WHO clinical staging requires confirmed HIV infection
- An infant aged under 12 months with only a positive HIV rapid antibody test can NOT be given a WHO clinical stage because in infants, HIV antibodies do not confirm HIV infection.
- However, an infant with HIV antibodies and specific clinical conditions is very likely to have AIDS and needs to start ART without delay (see definition of *Presumed Severe HIV Disease* below)
- WHO clinical staging is mandatory for all HIV patients, including those who are universally eligible for ART (confirmed infected children under 2 years, pregnant or breastfeeding women) or those with a CD4 count result
    - Keep a stack of blank (pre-) *ART Patient Cards* at OPD and complete staging for every new HIV patient

Table 3: WHO clinical staging for children and adults with confirmed HIV infection and definition of presumed severe HIV disease for infants

	Adults and Children	Adults only (15 years or older)	Children only (below 15 years)
**1**	• Asymptomatic • Persistent generalized lymphadenopathy		
**2**	• Respiratory tract infections, recurrent (sinusitis, tonsillitis, otitis media, pharyngitis<br>• Herpes zoster<br>• Angular cheilitis<br>• Oral ulcerations, recurrent<br>• Papular pruritic eruptions / Fungal nail infections	• Moderate weight loss <10%, unexplained<br>• Seborrhoeic dermatitis	• Hepatosplenomegaly, persistent unexplained<br>• Lineal gingival erythema<br>• Wart virus infection, extensive<br>• Molluscum contagiosum, extensive<br>• Parotid enlargement, persistent unexplained
**3**	• Fever, persistent unexplained, intermittent or constant, >1 month<br>• Oral hairy leukoplakia<br>• Pulmonary tuberculosis (current)<br>• Tuberculosis (PTB or EPTB) within the last 2 years<br>• Anaemia, unexplained < 8 g/dl<br>• Neutropaenia, unexplained < 500 /mm^3<br>• Thrombocytopaenia, chronic < 50,000 /mm^3	• Severe weight loss >10% and/or BMI <18.5kg/m^2, unexplained<br>• Diarrhoea, chronic (>1 month) unexplained<br>• Oral candidiasis<br>• Severe bacterial infections (pneumonia, empyema, pyomyositis, bone/joint, meningitis, bacteraemia)<br>• Acute necrotizing ulcerative stomatitis, gingivitis or periodontitis<br>• Hepatitis B or C infection	• Moderate unexplained wasting / malnutrition not responding to treatment (weight-for-height/ -age 70-79% or MUAC 11-12cm)<br>• Diarrhoea, persistent unexplained (14 days or more)<br>• Oral candidiasis (from age 2 months)<br>• Acute necrotizing ulcerative gingivitis or periodontitis<br>• Lymph node tuberculosis<br>• Bacterial pneumonia, severe recurrent<br>• Symptomatic lymphoid interstitial pneumonitis<br>• Chronic HIV-associated lung disease including brochiectasis
**4**	• Pneumocystis pneumonia<br>• Candidiasis of oesophagus, trachea, bronchi or lungs<br>• Extrapulmonary tuberculosis<br>• Kaposi's sarcoma<br>• HIV encephalopathy<br>• Cryptococcal meningitis or other Extrapulmonary cryptococcosis<br>• Disseminated non-tuberculous mycobacterial infection<br>• Cryptosporidiosis, chronic with diarrhoea<br>• Isosporiasis >1 month<br>• Disseminated mycosis (coccidiomycosis or histoplasmosis)<br>• Symptomatic HIV-associated nephropathy or cardiomyopathy<br>• Progressive multifocal leukoencephalopathy<br>• Cerebral or B-cell non-Hodgkin lymphoma	• HIV wasting syndrome (severe weight loss + persistent fever or severe weight loss + chronic diarrhoea)<br>• Bacterial pneumonia, recurrent severe<br>• Chronic herpes simplex infection (orolabial, genital / anorectal >1 month or visceral at any site)<br>• Cytomegalovirus infection (retinitis or infection of other organs)<br>• Toxoplasmosis of the brain<br>• Non-typhoidal Salmonella bacteraemia, recurrent<br>• Invasive cancer of cervix<br>• Leishmaniasis, atypical disseminated	• Severe unexplained wasting / malnutrition not responding to treatment (weight-for-height/ -age <70% or MUAC <11cm or oedema)<br>• Bacterial infections, severe recurrent (empyema, pyomyositis, bone/ joint, meningitis, but excluding pneumonia)<br>• Chronic herpes simplex infection (orolabial or cutaneous >1 month or visceral at any site)<br>• Cytomegalovirus infection: retinitis or other organ (from age 1 month)<br>• Toxoplasmosis of the brain (from age 1 month)<br>• Recto-vaginal fistula, HIV-associated<br><br>**Presumed Severe HIV Disease in infants <12 months *(PSHD)***<br>Positive antibody (rapid) test *PLUS*<br>one or several of the highlighted clinical conditions in the WHO staging list<br>*OR* combination of at least 2 of the following:<br>• Oral thrush • Severe sepsis • Severe pneumonia

6.3 Management of HIV-related diseases

Use the following list to identify and manage the main HIV HIV-related diseases seen in Malawi. A more detailed discussion is available in the *Management of HIV Associated Diseases* guidelines.

Oral candidiasis

Clinical Signs

Multiple whitish or red patches anywhere inside mouth

Primary Management

Nystatin oral suspension

Treat for 7-14 days; keep in mouth as long as possible; apply to mother's nipples if breastfeeding

Adult: 4ml 6-hourly

Child: 1ml 6-hourly

Secondary Management

3 Alternative treatment options if severe or no response to nystatin:

Fluconazole tablets

Treat for 14 days

Adult: 100 mg 24-hourly

Child: 6mg/kg on day 1 then 3mg/kg daily

Ketoconazole tablets

Do not give with NVP

Adult: 200mg 24-hourly for 14 days

Child: 5mg/kg 24-hourly for 14 days

Miconazole gum patch or gel

Use for children > 4 months and adults

Treat with 1 patch 24-hourly for 14 days

Oesophageal candidiasis

Clinical signs

Retrosternal pain on swallowing; infants and children refusing to eat; +/- oral thrush

Primary management

Fluconazole tablets

Treat for 14 days

Adult: 200mg 24-hourly for 14 days

Child: 12mg/kg day one then 6mg/kg

Chronic diarrhoea

Clinical signs

More than 3 loose non-bloody motions per 24 hours for more than 2 weeks

Diagnosis / investigations

Based on response to stepwise empirical treatment:

Step 1 treats: isospora, cyclospora, bacterial

Step 2 treats: giardia, clostridium, amoeba, microspor.

Step 3 treats: microspor., helminths

Primary management

ORS (Thanzi)

drink 5ml/kg 4-hourly and after every episode of diarrhoea.

drink 5ml doses every 5 min if vomiting occurs

IV Fluids

if severe de-hydration

Loperamide tablets

Adult: 2mg after every loose stool (max 12mg in 24 hours)

Child: Do NOT use for children

Step 1: Cotrimoxazole tablets

Adult: 960mg 8-hourly for 7 days

Child: 80 mg/kg 8-hourly for 7 days

Zinc tablets

Give for 10 days

Child 0-6mths: 10 mg 24-hourly

Child 6mths – 5 yrs: 20 mg 24-hourly

Secondary management

Continue with step 2 and 3 if no improvement

Step 2: Metronidazole tablets

Adult: 800mg 8-hourly for 7 days

Child: 15mg/kg 8-hourly for 7 days

Step 3: Albendazole tablets

Adult: 400mg 12-hourly for 14 days

TB

Clinical signs

Very variable: Persistent fever / drenching night sweats; weight loss; failure to thrive; persistent cough; anaemia <8g/dl; enlarged nodes; meningitis signs

Diagnosis / investigations

TB case / TB suspect in household; 3x sputum for AAFB; CXR; fine needle aspiration nodes (for

microscopy); pleural tap for biochemistry: straw coloured effusion?; lumbar puncture: CSF for biochemistry, microscopy

Primary management

1st Line TB treatment

Consider presumptive TB treatment in HIV patients with suspected TB

New smear-positive or negative PTB:

Intensive phase: 2 RHZE
Continuation phase: 4 RH

TB Meningitis:

Intensive phase: 2 SRHZ
Continuation phase: 7 RH

Secondary management

Relapse/ return after default/ treatment failure/ recurrent TB

Admit for Intensive phase: 2 SHRZE
1 RHZE (in hospital)
Continuation phase: 5 RHE

Chronic/MDR-TB

Specialised treatment

Kaposi sarcoma

Clinical signs

Single or multiple purple patches or nodes, mainly mouth, skin, conjunctiva, lung, GI tract; +/- enlarged nodes; +/- Oedema

Diagnosis / investigations

Usually clear picture; children often present with lymphadenopathy only; consider KS even without skin or oral lesions if no response to EPTB therapy within 4 weeks

Primary management

For all patients:

Analgesia
Symptomatic treatment
ART
For KS stage T0 (skin KS without oedema):

Delayed chemotherapy if no improvement after 3 months on ART

For KS stage T1 (KS in mouth or internal organs, nodular skin KS, skin KS with oedema):

Immediate chemotherapy
Contraindications for chemotherapy: Severe PN; Hb<10g/dl; platelet count <50/mm^3; jaundice; pregnancy

1st Line: Vincristine

Each cycle consists of 6 doses; ensure strictly IV injection as infiltration causes burns; document therapy and response in health passport; examine for recurrence at every visit

Adult: 2mg vincristine IV
Child: 0.05 mg/kg vincristine IV (max 2mg)

Review after every cycle:

Severe neuropathy / constipation: stop
Lesions cleared: stop
Good response but residual lesions: continue next cycle
Poor response: Start 2nd line chemotherapy

1) Initial cycle:
   1 dose every 7 days for 6 weeks
2) Second cycle:
   1 dose every 14 days for 12 weeks
3) Final cycle:
   1 dose every 28 days for 6 months

Secondary management

2nd Line: Vincristine + Bleomycin

Cumulative max. lifetime dose for Bleomycin is 400 units for adults and 17 doses for children; stop bleomycin immediately if any sign for lung fibrosis (incl. cough, shortness of breath) are seen; give one combined dose every 14 days until cumulative max. dose is reached or until response is achieved; refer for 3rd line chemotherapy (doxorubicin) if poor response

Adult: 15 units bleomycin IM / IV / SC **plus** 2mg vincristine IV
Child: 0.5 mg/kg bleomycin IM **plus** 0.05 mg/kg vincristine IV (max 2mg)

Cervical cancer

Clinical signs

No early symptoms therefore active screening needed; abnormal vaginal discharge

Diagnosis / investigations

Acetic acid visualisation (VIA)

Use good light source
Expose cervix with cusco speculum, visualise cervix after washing for 2 minutes with a large cotton swab immersed in 4% acetic acid

Primary management

Surgical, depending on stage

Shingles (Herpes zoster)

Clinical signs

Grouped blisters in one patch; intense pain / burning; +/- fever; +/- body pains; lesions do not usually cross the body's mid-line

Primary management

Analgesic Ladder
Rigorous pain control
Acyclovir tablets
Must be started before blisters burst
Adult: 800mg 5 times per day for 7 days
Child: 20 mg/kg 8-hourly for 7 days
If face affected:

Refer to Eye specialist
Monitor for secondary bacterial infection

Seborrhoeic dermatitis

Clinical signs

Greasy, scaly rash in axilla, groin, scalp, neck, face

Primary management

Clotrimazole or Miconazole cream / ointment

Secondary management

Ketoconazole tablets
200 mg twice daily for 7 days

Tinea corporis / cruris / pedis

Clinical signs

Round reddened plaques with scaly edge in multiple sites, poss. widespread

Primary management

Whitfield's ointment
Clotrimazole cream or Gentian-Violet paint
Apply twice daily for 3-4 weeks

Secondary management

Griseofulvin tablets
Adult: 500 mg 12-hourly for 4-6 weeks
Child: 20mg/kg per day for 4-6 weeks

Pruritic papular eruptions

Clinical signs

Severe itching, evenly distributed normal- or dark-coloured papules on trunk, arms or legs, often scratch-lesions

Primary management

Calamine Lotion
Antihistamines

Secondary management

Corticosteroid cream or tablets
Metronidazole tablets
250mg 12-hourly for 7-14 days

Pneumocystis carinii (jiroveci) pneumonia (PCP)

Clinical signs

- Extreme shortness of breath; dry cough; +/- fever
- Severe pneumonia in infants <12 months

Diagnosis / investigations

O_2 saturation: hypoxia

CXR: Diffuse interstitial or hyperinflation; bats wing shadow

Treat for empirically for PCP any HIV exposed or confirmed infected infant presenting with severe pneumonia

Primary management

Admit
Oxygen
Cotrimoxazole tablets
Adult: 4 x 480mg 8-hourly for 21 days
Child: 80mg/kg 8-hourly for 21 days
Lifelong maintenance (CPT)
IV Cotrimoxazole if unable to swallow and NGT impossible to place
Prednisolone tablets:
Give 15-30 minutes before cotrim
Adult: 8 tablets 12-hourly for 5 days
8 tablet 24-hourly for 5 days
4 tablets 24-hourly for 11 days
Child: 2mg/kg 24-hourly for 7 days
1mg/kg 24-hourly for 7 days
0.5mg/kg 24-hourly for 7 days

Secondary management

Clindamycin
300mg 6-hourly for 3 weeks
plus
Primaquine
30mg 24-hourly for 3 weeks

Cryptococcal meningitis

Clinical signs

Slow onset severe headache; confusion; convulsions; +/- fever; +/- neck stiffness

Diagnosis / investigations

CSF India ink stain; cryptococcal antigen in serum or CSF

Primary management

Admit

Daily therapeutic spinal tap
(up to 20ml per puncture)

Fluconazole tablets

Adult: 1200mg 24-hourly for 14 days
400mg 24-hourly for 42 days
200mg 24-hourly for life

Child: 12mg/kg 24-hourly for 2 weeks
6mg/kg 24-hourly for life

Secondary management

Amphotericin B

Specialised sites only
Adult and Child: 0.7-1mg/kg IV over 6 hours 24-hourly for 14 days
Follow acute treatment with Fluconazole for life

Fluconazole tablets

Adult: 400mg 24-hourly for 42 days
200mg 24-hourly for life

Child: 6mg/kg 24-hourly for life

Pneumonia

Clinical signs

Productive cough; chest pain; fever; tachypnoea / dyspnoea

Diagnosis / investigations

Infiltrations on CXR

Primary management

Child:

Mild: Tachypnoea but no dyspnoea

(See IMCI guidelines)
Adult:

Mild to moderate presentation:

Amoxicillin tablets
500mg 8-hourly for 5 days
Doxycycline or Erythromycin if no response

Secondary management

Severe presentation:

Chloramphenicol + Benzyl Penicillin
Add Gentamycin if no response

Sepsis

Clinical signs

Severe illness; fever (can be absent, especially in children); fast heart rate; fast breathing

Diagnosis / investigations

+/- Malaria parasites; do not rule out sepsis if malaria parasites are seen; blood culture for culture and sensitivity (if available)

Primary management

Health Centre Level:

Immediate presumptive treatment
Referral to hospital

Child:

Benzyl Pen 50,000 IU/kg IV or IM stat +
Gentamycin 7.5mg/kg slow IV / IM stat +
Quinine 10mg/kg IM stat

Adult:

Chloramphenicol 1g IV or IM stat +
Gentamycin 240mg slow IV or IM stat +
Quinine 1200mg IV in 5% dextrose over 4 hours

Secondary management

Hospital management:

Neonate:

Benzyl Pen 50,000 IU/kg IV 8-hourly +
Gentamycin 7.5 mg/kg IV 24-hourly

Child:

Gentamicin 7.5.mg/kg 24-hourly + **Benzyl Pen** 50,000 IU/kg IV 8-hourly
OR
Ceftriaxone 50-100 mg/kg IV 24-hourly
OR (if pneumococcal sepsis suspected)
Chloramphenicol 25 mg/kg IV 8-hourly (max. 1g per dose)

When stable continue to complete 10 days:
Amoxicillin 40 mg/kg 12-hourly +
Ciprofloxacin 15 mg/kg 8-hourly

Adult:

Ceftriaxone 2g IV 24-hourly + **Ciprofloxacin** 500 mg tablets 12-hourly + **Amoxicillin** 500 mg tablets 8-hourly for 5 days

6.4 Standard monitoring of HIV patients

Key Facts for Providers and Patients

- All patients in HIV Care (exposed infants, pre-ART, ART) need the same standard clinical assessment at every visit
- Check actively – do not rely on patients to report problems unprompted
- The Standard Clinical Monitoring Checklist (Table 5 on Page 23) helps to find:
    - HIV-related diseases
    - WHO clinical stage
    - ART treatment failure
    - drug side effects (ART, TB, CPT, IPT, etc.)
- It can be difficult to distinguish HIV-related diseases from drug side effects
    - An ambiguous symptom is likely a side-effect if it started or worsened after the start of medication

6.4.1 Monitoring of nutritional status

- One of the simplest methods to detect HIV disease progression / ART treatment failure
- Record length / height to the nearest cm at every visit (children) / once at enrolment (adults)
- Record weight in kg to the nearest 100g at every visit (children and adults)
- Use appropriate nutrition indicator for children and adults

Children 0-14 years

- Classify wasting / malnutrition status according to weight-for-height form **Figure 2** on page 20
- Watch out for flattening of the growth curve (weight for age)
- Weight-for-height less than 80% (below green curve in **Figure 2**) and/or MUAC less than 12cm:
    - Investigate for TB
    - Refer / admit for Therapeutic Feeding
    - Start ART if no response to TF after 3 weeks (WHO stage 3)

Non-pregnant adults 15 years and above

- Classify nutrition status according to BMI from **Figure 3** on page 21
- $BMI = \frac{weight(kg)}{height(m) \times height(m)}$
- Watch out for any weight loss over time

  - Review documented previous weight whenever available as reported weight loss can be unreliable
  - Investigate any weight loss for TB
- Weight loss >10% and/or BMI under 18.5 (below green curve in **Figure 3**
  - Investigate for TB
  - Start ART if weight loss unexplained (WHO stage 3)
- BMI under 17 (below yellow curve in **Figure 3**)
  - Start TF for ‘moderate malnutrition’
- BMI under 16 (below yellow curve in **Figure 3**)
  - Start TF for ‘severe malnutrition’

Pregnant and lactating women

- Use MUAC instead of BMI
- Universally eligible for ART if confirmed HIV infection
- MUAC less than 22cm: start TF for ‘moderate malnutrition’
- MUAC less than 19cm: start TF for ‘severe malnutrition’

Figure 2: Weight for Height classification of wasting / malnutrition for children 0 - 14 years

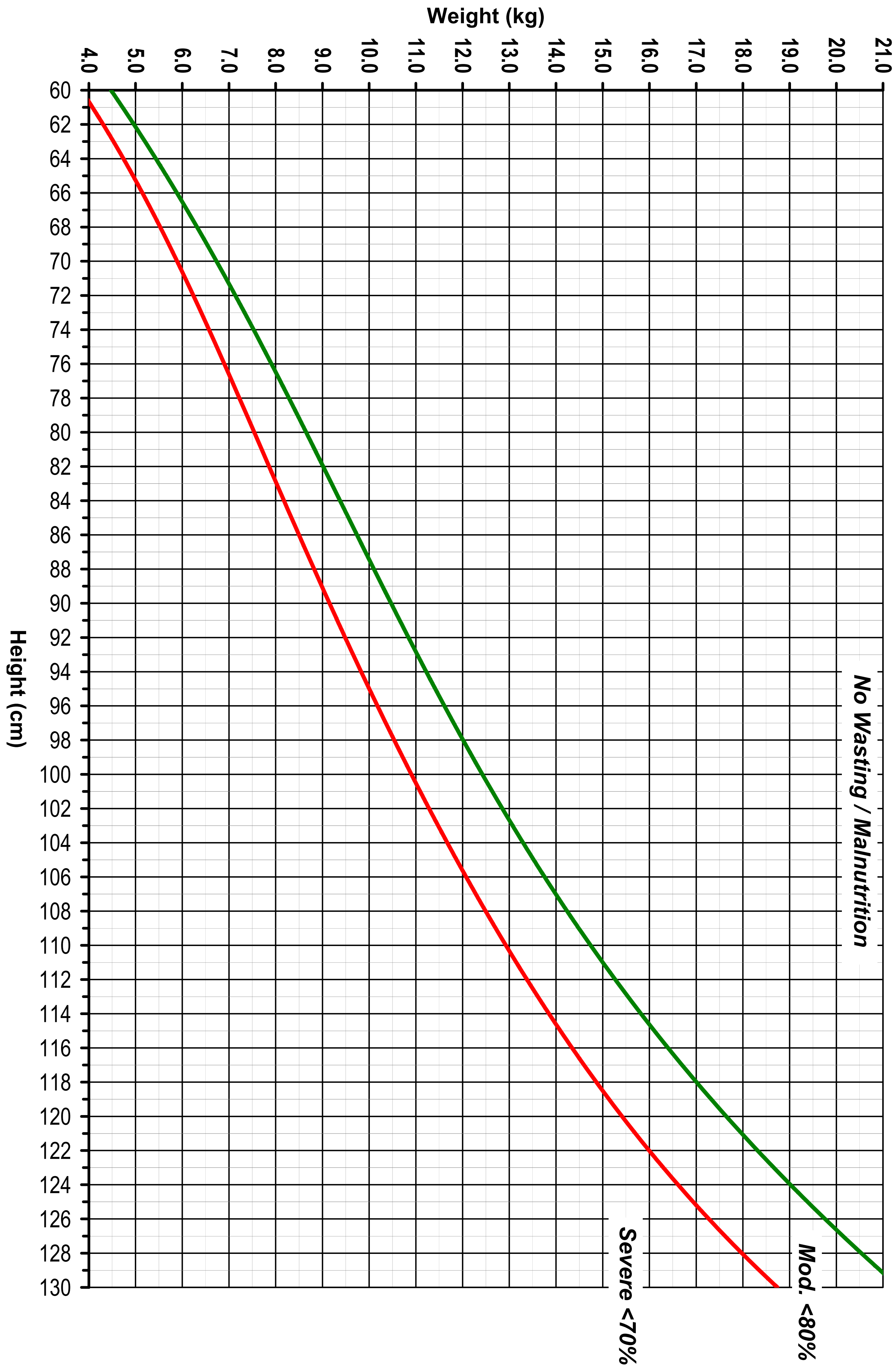

Figure 3: BMI classification of malnutrition for non-pregnant adults 15 years and above

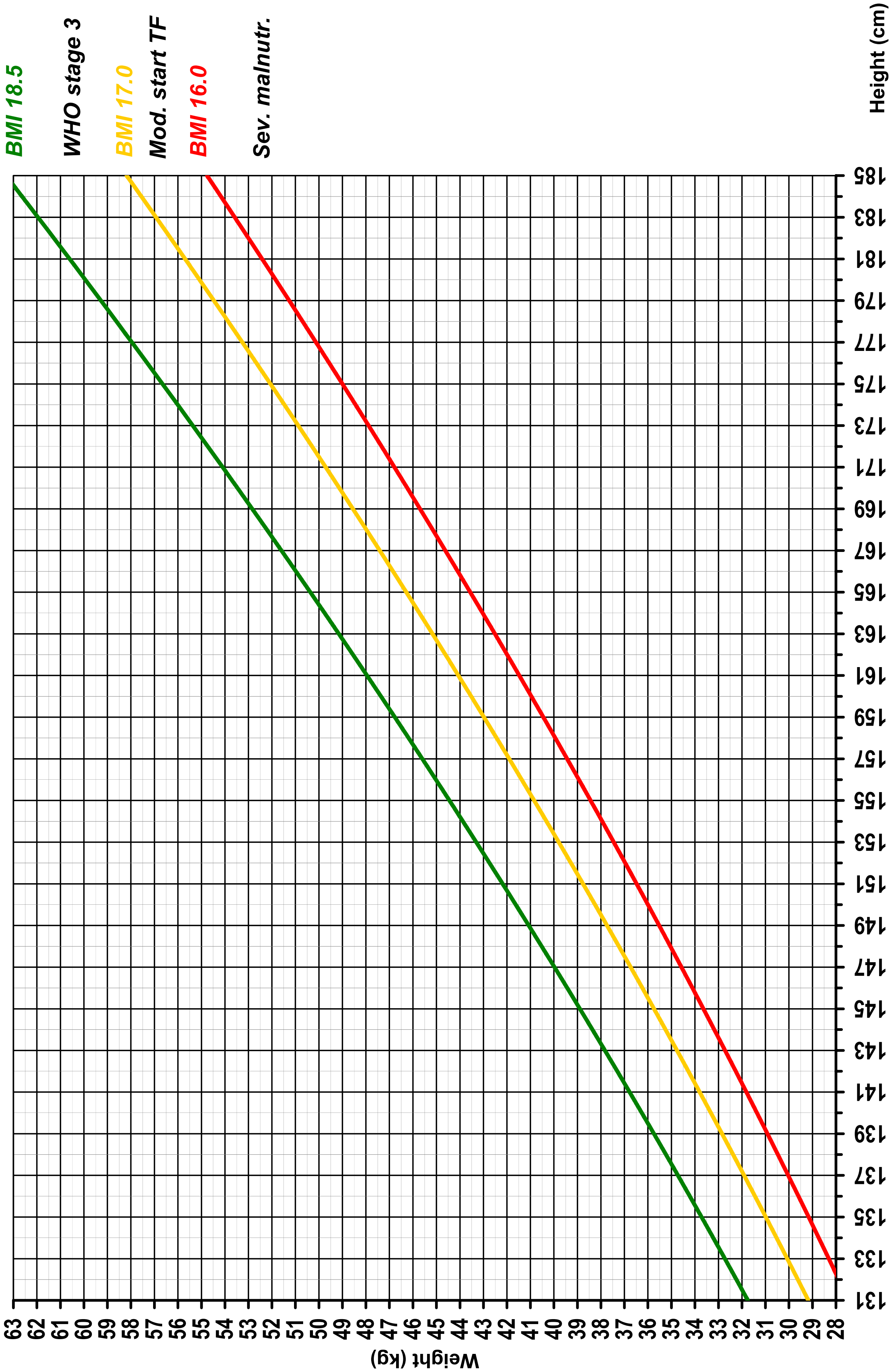

6.4.2 Standard clinical monitoring checklist

- Use the summary clinical monitoring checklist to actively screen every HIV patient (exposed child, pre-ART and ART) for clinical symptoms at every visit.
- Refer to **Table 5** on page 23 for more detailed screening instructions and interpretation of signs and symptoms for further management.

Table 4: Standard checklist for clinical monitoring of HIV exposed children, pre-ART and ART patients

Ask for / Examine			
Appearance:	Body shape change	N	Y
	Malnutrition	N	Y
	Swollen glands	N	Y
Headache / confusion / dizziness		N	Y
Yellow eyes		N	Y
Mouth sores		N	Y
Cough		N	Y
Shortness of breath		N	Y
Fever / night sweats		N	Y
Vomiting / abdominal pain		N	Y
Diarrhoea		N	Y
Leg pain / numbness / weakness		N	Y
Rash on arms, legs or trunk		N	Y

Table 5: Detailed clinical monitoring list for HIV exposed children, pre-ART and ART patients

Ask / Examine	Look for	Assess	Disease (most common)	Drug Side-Effects
**Appearance**	• Weight loss<br>• Failure to thrive	• Weight loss: trend from patient card / health passport<br>• BMI (adults)<br>• Weight for height, weight for age, MUAC (children)	1) TB<br>2) Chronic diarrhoea<br>3) Malnutrition<br>4) ART treatment failure<br>5) Malignancy (lymphoma)	**Lactic acidosis due to ART**<br>1) d4T<br>2) ddI<br>3) AZT
	• Body Shape	• Slimming of cheeks<br>• Slimming of forearms, buttocks and legs +/- protruding veins<br>• Fattening of chest / belly / buttocks<br>• Buffalo hump		**ART induced lipodystrophy**<br>1) d4T<br>2) ddI<br>3) AZT<br>4) 3TC
	Swollen glands	• Cervical / axillary lymphadenopathy	1) PGL<br>2) EPTB<br>3) Lymphoma<br>4) KS (+/- skin lesions)<br>5) BCG adenitis	
**Headache, confusion, dizziness**	• Neck stiffness<br>• Nausea / vomiting		1) Meningitis (bacterial/ TB, cryptococcal)<br>2) Toxoplasmosis<br>HIV dementia	1) EFV
**Yellow eyes**	• Yellow sclera	• Jaundice	1) Viral hepatitis<br>2) Alcoholic hepatitis<br>3) Malaria<br>4) Cancer<br>5) Hep B-IRIS	**Drug hepatitis**<br>1) NVP<br>2) EFV<br>3) Rifampicin<br>4) INH<br>5) PZA<br>6) Fluconazole

Ask / Examine	Look for	Assess	Disease (most common)	Drug Side-Effects
**Mouth sores**	• Mucosa lesions	• Whitish patches<br>• Painful red patches	1) Oral thrush<br>2) Oral hairy leukoplakia	
		• Purple lesions	1) KS	
		• Ulcerations	1) Acute ulcerative stomatitis/ gingivitis/ periodontitis<br>2) Herpes simplex<br>3) Angular cheilitis<br>4) Aphthous ulcers	**Hypersensitivity**<br>1) ABC<br>2) NVP<br>3) Cotrimoxazole
**Cough**	• Duration<br>• Productiveness	• Less than 2 weeks<br>• Fever<br>• +/- Productive	1) Pneumonia (bacterial)<br>2) TB suspect: circle on card<br>3) PCP	**Hypersensitivity**<br>1) ABC
		• More than 2 weeks<br>• Fever / night sweats	1) TB suspect: circle on card<br>2) KS	
**Shortness of breath**	• Observe breathing<br>• Pleural effusion	• Pleural effusion	1) EPTB<br>2) Bacterial pneumonia<br>3) Heart failure<br>4) KS	
		• No pleural effusion	1) Bacterial pneumonia<br>2) PCP<br>3) TB +/- pneumothorax	**Lactic acidosis due to ART**<br>1) d4T<br>2) ddI<br>3) AZT
	• Conjunctiva	• Pale conjunctiva	1) HIV anaemia<br>2) Chronic severe malaria<br>3) Nutritional anaemia	**Anaemia**<br>1) AZT

Ask / Examine	Look for	Assess	Disease (most common)	Drug Side-Effects
**Fever / night sweats**	• History / Duration<br>• Current temperature	• Less than 1 month	1) URTI / viral<br>2) Sepsis<br>3) Malaria<br>4) TB	**Hypersensitivity**<br>1) ABC<br>2) NVP<br>3) Cotrimoxazole
		• More than 1 month	1) TB<br>2) Malignancies (lymphomas)	
**Vomiting / abdominal pain**	• Hydration status<br>• Palpate abdomen	• Dehydration<br>• Tenderness	1) TB<br>2) NTS sepsis<br>3) Acute Gastro-enteritis<br>4) Malaria<br>5) Abdominal TB<br>6) Ulcer disease<br>7) CNS disease<br>8) Hepatoma	**Drug-induced pancreatitis**<br>1) d4T<br>2) ddI<br>3) 3TC<br>**Lactic acidosis due to ART**<br>1) d4T<br>2) ddI<br>3) AZT
**Diarrhoea**	• History<br>• Blood in stool	• Less than 1 month<br><br><br><br><br>• Longer than 1 month	1) Salmonella<br>E. Coli<br>Amoeba, Shigella<br>2) HIV / OI<br><br>1) HIV / OI<br>2) Abdominal TB	**GI toxicity**<br>1) LPV/r<br>2) NVP<br>3) AZT<br>4) ABC<br>5) 3TC<br>Antibiotics:<br>Pseudomembranous enterocolitis
**Leg pain, numbness, weakness**	• History<br>• Neurological exam	• Sleep disturbance (moderate)<br>• Motor involvement (severe)	1) HIV peripheral neuropathy<br>2) EPTB	**Drug neuropathy**<br>1) d4T<br>4) INH<br>2) ddI<br>3) AZT<br>5) Vincristine<br>6) Metronidazole

Ask / Examine	Look for	Assess	Disease (most common)	Drug Side-Effects
**Rash on arms, legs and trunk**	• Skin lesions	• Purple lesions	1) KS	
		• Blisters/ vesicles	1) Shingles/ varicella zoster	Stevens-Johnson Syndrome<br>1) NVP<br>2) Cotrimoxazole
	• Generalized rash	• Maculo-papular	1) HIV associated rash (PPE)<br>2) Fungal skin infections<br>3) Molluscum contagiosum<br>4) Scabies	**Skin toxicity**<br>1) NVP<br>2) EFV<br>3) CTX<br>4) Fluconazole

6.4.3 CD4 monitoring for ART eligibility

Key Facts for Providers and Patients

- CD4 counts are the most direct routine measure for HIV immune suppression, but can be influenced by several factors:
  - Gender, time of day, physical exercise, pregnancy, smoking, etc.
- CD4 counts do not replace clinical staging
- Use CD4 counts only to monitor ART eligibility in patients who would otherwise not be eligible. CD4 counts are not needed for the following patients:
  - Age under 2 years
  - WHO clinical stage 3 or 4
  - Pregnant or breastfeeding
- Do not use CD4 counts for routine monitoring of patients on ART
- CD4 counts must not be used to delay or cancel ART initiation in patients who are otherwise eligible (pregnant or breastfeeding, age <2 years, WHO clinical stage 3 or 4)
- Repeat CD4 counts for patients over 5 years in pre-ART follow-up every 6 months
  - Move to 3-monthly CD4 counts if last count was less than 500
  - Repeat CD4 counts for children aged 2-5 years every 3 months
- CD4 counts may fail or give wrong results unless the following protocol is used:
  - Collect a minimum of 2ml venous blood in tube with anticoagulant (EDTA)
  - Immediately turn tube upside down to mix the blood with the EDTA. Do not shake the tube vigorously.
  - The blood must be processed in the lab within 6 hours or 48 hours, depending on the type of CD4 machine used (PARTEC or FACS Count, respectively)
  - Storing the tube at 2-8°C in the dark will extend the life-span by a few hours
  - Protect the tube from hard vibrations during transport

CD4 monitoring of patients in HIV Care Clinic follow-up

- Routinely perform clinical monitoring for all HIV exposed children and pre-ART patient at every HCC visit (see Table 5 on Page 23). This covers WHO clinical staging and identification of HIV-related diseases and potential drug toxicity
- Every 6 months (at every 2nd 3-monthly visit), do routine CD4 count for patients with confirmed HIV infection (pre-ART patients) who are not otherwise eligible for ART
  - Give CD4 count result to the patient at the next scheduled visit (after 3 months). Giving an extra (earlier) appointment for picking up of CD4 results is usually too burdensome for the patient

- Stop CD4 monitoring once a patient has CD4 results or a clinical condition which makes them eligible for ART.

6.4.4 Definition of ART eligibility

Key Facts for Providers and Patients

- All patients need to be assessed clinically, regardless of other criteria that may make them eligible to start ART (CD4 count results, universal eligibility based on age, pregnancy or breastfeeding)
- Patients always remain eligible to start ART if they have satisfied eligibility criteria once
- *Universal ART* is limited to certain patients for certain time periods:
  - Children aged 24 months and above are NOT universally eligible to start ART (see Section 6.4.4)
  - HIV infected women who did not start ART while pregnant or breastfeeding need to be in WHO stage 3 or 4 or have a CD4 count below the threshold
- Use the flowchart in **Figure 4** on page 29 to classify the *Reason for starting ART.* Some patients may be eligible for ART on the basis of different conditions. In this case, WHO clinical stage 3 and 4 are considered to 'override' other eligibility criteria.

Infant under 12 months

- **Universal ART:** Confirmed HIV infection (DNA-PCR needed), regardless of WHO stage and CD4 count or CD4 %
- **Presumed severe HIV disease (PSHD):** HIV antibodies (HIV rapid antibody test) and PSHD-defining clinical conditions (see WHO Clinical Staging Chart)

Child 12 to under 24 months

- **Universal ART:** Confirmed HIV infection (HIV rapid antibody test or DNA-PCR), regardless of WHO stage and CD4 count

Child 24 months to under 5 years

- Confirmed HIV infection (HIV rapid antibody test) **and**
  - WHO stage 1 or 2 and CD4 ≤750 cells/mm^3 or ≤25%

    OR
  - WHO clinical stage 3 or 4 regardless of CD4 count

Child or adult 5 years and over

- Confirmed HIV infection (HIV rapid antibody test) **and**
    - Pregnant or breastfeeding women (regardless of the age of the child) regardless of WHO stage and CD4 count

      OR
    - WHO stage 1 or 2 and CD4 ≤350 cells/mm^3

      OR
    - WHO clinical stage 3 or 4 regardless of CD4 count

Figure 4: Flowchart for classification of *Reason for Starting ART* (shaded boxes) based on the hierarchy of ART eligibility criteria

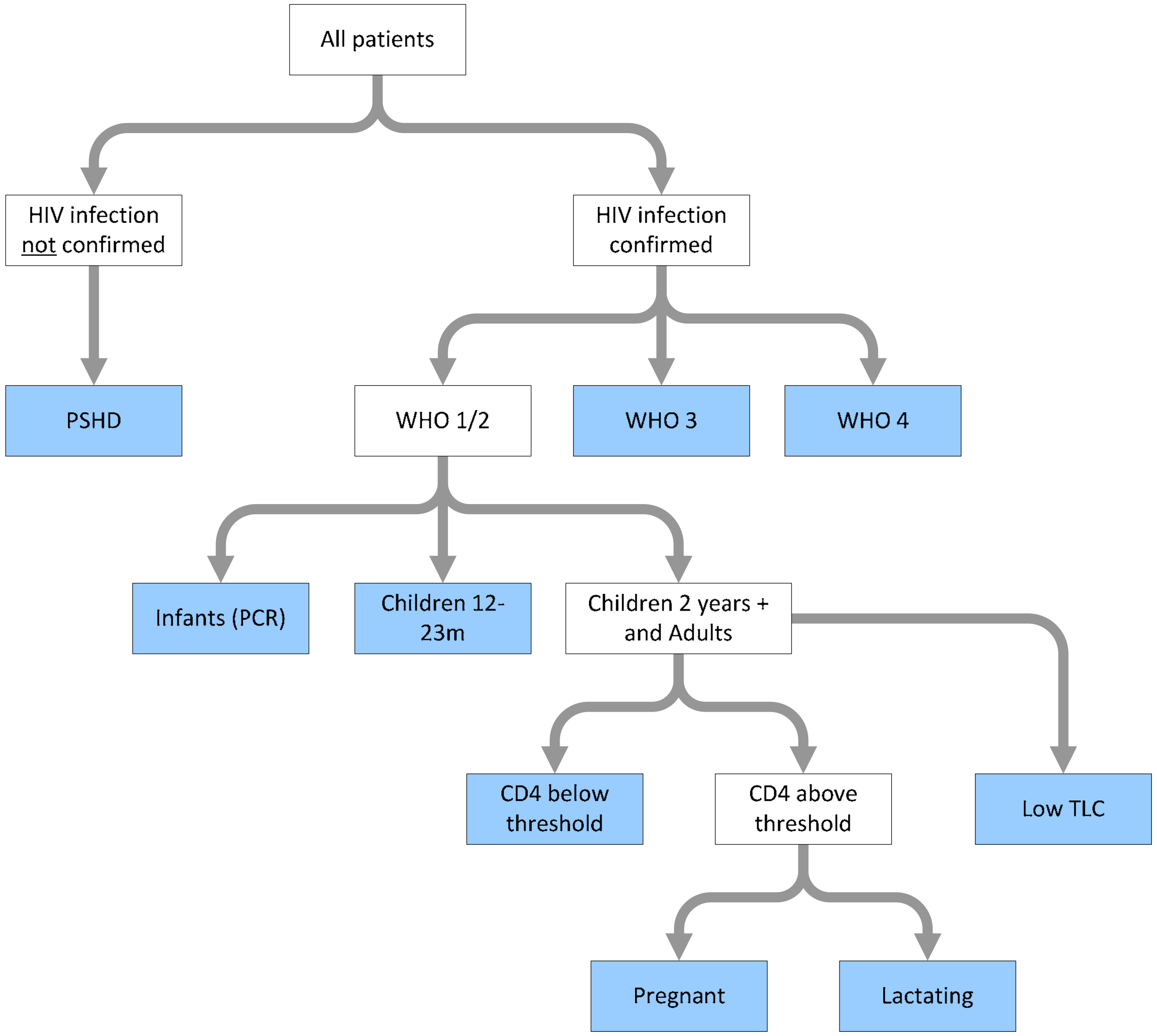

6.5 Preventive services for HIV patients

Key Facts for Providers and Patients

- A simple standard package of preventive services is provided for all patients in HIV Care (exposed children, pre-ART children and adults) and ART Clinics. This includes:
    1. Provider initiated family planning (condoms + Depo-Provera)
    2. 'Prevention with positives'
    3. Cotrimoxazole preventive therapy
    4. Isoniazid preventive therapy (not for patients on ART)
    5. Insecticide treated bed nets
- This package effectively reduces:
    - HIV transmission to sexual partners
    - HIV transmission from mother to child by preventing unwanted pregnancies
    - Serious HIV-related diseases (TB, diarrhoea, pneumonia, malaria, etc.)
    - HIV disease progression, deferring the need to start ART
    - Risk of premature death

6.5.1 Provider initiated family planning (PIFP)

Key Facts for Providers and Patients

- Avoid unwanted pregnancies, regardless of HIV infection status
- Unprotected sex is a risk for discordant and concordant HIV infected couples
- Couples should use dual protection – condoms alone are not enough for family planning as they have to be used very consistently
- Use the 3-monthly injection (Depo-Provera) for family planning for HIV infected women. It is safe with TB treatment and ART.
- Encourage HIV positive women to make an informed choice about pregnancy. Health workers should not actively discourage pregnancy as the risk of transmitting HIV to the baby is less than 5% if the mother :
    - Starts ART in the second trimester
    - Is fully adherent to ART throughout pregnancy and breastfeeding

Implementing routine PIFP in HIV clinic

- Assume that all patients aged 15 years and above are sexually active
- Offer condoms to all men and condoms and Depo-Provera to all women age 15 years and above:
    - Minimum **30** male or female condoms
    - 1 Depo-Provera injection every 3 months
- Give patients the opportunity to refuse either method if they feel they don't need it
- Refer clients to FP clinics for further counselling or for other FP methods

Giving Depo-Provera

- Depo-Provera is the best hormonal contraception for women on ART and/or TB treatment as there are no relevant drug interactions.
- Inject 1 vial (150mg) in the deltoid muscle

Contraindications

- Jaundice
- Possibility of current pregnancy
- Suspected or known breast cancer

6.5.2 Prevention with positives (PwP)

- Assess and counsel at every visit for:
    - High risk sexual activity
    - Partner's and children's HIV status (See Section 6.1 on Page 8)
    - Disclosure to partner/ guardian/ treatment supporter
    - Signs and symptoms of STIs
    - Pregnancy status
    - Adherence to ART and other medications (See Section 6.9.6 on Page 53);
    - Abuse of alcohol and other substances
    - Positive living (nutrition, alcohol and smoking cessation)

6.5.3 Cotrimoxazole preventive therapy (CPT)

Key Facts for Providers and Patients

- CPT prevents PCP pneumonia, diarrhoea, malaria and other HIV-related diseases and prolongs survival
- Start all HIV exposed and infected children from age 6 weeks and adults regardless of clinical stage or CD4 count on CPT
- Provide CPT to all patients in HCC and ART follow-up
- Continue CPT for life for all HIV positive patients
- Stop CPT in HIV exposed children when confirmed negative after stopping of breastfeeding (when discharged from exposed infant follow-up)
- CPT is tolerated very well by most patients, can be taken at the same time with ART, TB treatment and IPT and is safe in pregnancy
- Do not combine CPT with SP – HIV positive pregnant women should only take CPT
- Children from 30.0kg and adults take one 480mg tablet of cotrimoxazole 12-hourly
- Dispersible paediatric tablets (120mg) are used for children under 14.0kg. Dosing of paediatric CPT and ART is both based on the same weight bands.
- CPT is usually available in blister-packs of 60 tablets – enough for 30 days supply
- Poor adherence to CPT is a warning sign for poor adherence to ART

Eligibility and when to start CPT

- All infants born to HIV infected mothers (without confirmed HIV infection) from age 6 weeks
  - Aim to start CPT straight after the infant has finished NVP syrup
  - NB: having taken NVP prophylaxis is NOT a condition for starting CPT
  - Keep the infant on CPT until s/he is confirmed HIV-negative and is discharged from HCC follow-up (around age 24 months)
- Confirmed HIV infected children from age 6 weeks and adults
  - Regardless of CD4 count or clinical stage
  - No contra-indication against CPT in the first trimester of pregnancy
  - Start CPT as soon as possible in pregnancy
  - Do not give SP to HIV infected pregnant women on CPT
  - If SP has already been taken, wait for 14 days before starting CPT

Contraindications

- Jaundice
- Renal failure
- Suspected allergy to any of the following sulfonamide drugs (skin rash, mucosal ulceration, severe anaemia, leukopenia)
    - Cotrimoxazole
    - Sulfadoxine/Pyrimethamine (SP)

Table 6: Dosage of Cotrimoxazole Preventive Therapy

Weight	480mg tablets	120 mg dispersible tablets	Syrup
less than 6kg	¼ tablet 24-hourly	1 tablet 24-hourly	2.5ml 24-hourly
6.0 – 13.9kg	½ tablet 24-hourly	2 tablets 24-hourly	5ml 24-hourly
14.0 – 29.9kg	1 tablet 24-hourly		
30.0kg and above	1 tablet 12-hourly		

Who and when to stop on CPT

- HIV exposed children: when confirmed HIV negative at age 24 months (or older when confirmed HIV negative at least 6 weeks after stopping of breastfeeding)
- HIV infected children and adults continue CPT for life, unless severe side effects develop
- Poor adherence to CPT will reduce the effectiveness of preventing HIV-related diseases, but it is less risky than poor adherence to ART.

6.5.4 Isoniazid preventive therapy (IPT)

Key Facts for Providers and Patients

- Daily IPT can prevent TB in people who are at high risk of developing TB
- Give IPT to the following:
    - HIV infected children and adults who are not on ART, regardless of WHO clinical stage or CD4 count
    - Children under 5 years (regardless of HIV status) who live with a patient with pulmonary TB (sputum smear negative or positive) who has not yet completed 2 months of TB treatment
- Start IPT at enrolment for pre-ART follow-up and continue for as long as the patient is in pre-ART follow-up
- Stop IPT when ART is started
- Do not give IPT to a patient who has any signs suggestive of active TB: such patients need full investigation for TB and combination TB treatment if confirmed to avoid TB drug resistance
- IPT is well tolerated and can be taken with CPT and in pregnancy
- Stop IPT if any of the following are seen:
    - Severe skin rash
    - Yellow eyes
    - Confusion / convulsions
    - Dizziness
    - Severe numbness/burning pain and muscular weakness of legs and/or arms

Eligibility for IPT

HIV infected patients are eligible to start IPT if they fulfil all of the following conditions:

- Age 2 years and older (as all children under 2 years with confirmed HIV infection should be on ART)
- Not on ART
- Active TB ruled out. Use the standard TB screening questions below:
    - Current cough: any duration, productive or non-productive
    - Unexplained weight loss (adults)
    - Failure to thrive and/or malnutrition (children)
    - Fever and/or night sweat

IPT contraindications

- Suspected or confirmed active TB

- Active hepatitis
- Severe peripheral neuropathy

IPT dosage and duration

- Provide IPT during pre-ART visits. One extra visit is needed 1 month after starting IPT
- Review patient at month 1, 3 and 6 after starting IPT for any side-effects
  - IPT initiation: Give INH and pyridoxine for 1 month
  - 1 Month IPT review: Give INH and pyridoxine for 2 months
  - From 3 Month IPT review: Continue giving INH and pyridoxine for 3 months
- Give pyridoxine 1 tablet 25mg 24-hourly to children and adults who are taking IPT
- Stop IPT when the patient starts ART, regardless of how long IPT has been taken

Table 7: Dosage for Isoniazid Preventive Therapy

Weight	Isoniazid Dose
Under 10kg	100 mg (1 tablet) 24-hourly
10- 13.9 kg	150 mg (1 ½ tablet) 24-hourly
14- 19.9 kg	200 mg (2 tablets) 24-hourly
20- 24.9 kg	250 mg (2 ½ tablets) 24-hourly
25 kg or above	300 mg (3 tablets) 24-hourly

6.5.5 Insecticide treated bed nets (ITN)

- Dispense 1 ITN to each patient at enrolment into the HIV Care / ART clinic
- Dispense 1 replacement ITN every 2 years and document this on the (pre-) ART patient card

6.5.6 Infant and child feeding counselling

Key Facts for Providers and Patients

- Feeding recommendations are the same for all infants, regardless of HIV exposure or HIV infection status
- Give only breast milk up to age 6 months
- Gradually start complementing breastfeeding with suitable hygienically prepared foods from age 6 months (such as Likuni Phala, fruits, vegetables, beans, ground nuts and soya)
- Stop breastfeeding around age 24 months
- Stop breastfeeding gradually over a period of 1 month (no *rapid cessation*)

Additional key messages for health workers and patients

- Replacement feeding (formula) is **NOT** recommended unless women are unable to breast feed
- Monitor weight, height and MUAC according to schedule using standard MOH charts and intervene if no adequate weight-gain
- Give only medicines prescribed by a health professional
- Start breastfeeding immediately after birth. Explain and observe optimal breastfeeding:
    - Empty both breasts properly to avoid breast engorgement
    - Ensure proper attachment and positioning to minimize nipple cracks and fissures
    - Watch out for signs of breast infection (pain, swelling, heat, redness)
        - Don't feed baby from infected breast
        - Express infected breast to avoid engorgement. Discard expressed milk – do not feed to baby.
        - Go to health facility for treatment

6.6 Understanding ART regimens and formulations

Key Facts for Providers and Patients

- ART requires combining **3 different ARVs** that act differently in order to avoid development of drug-resistant HIV.
- Use only the standard ARV regimens for the specified patient groups shown in these guidelines. Other ARV combinations may cause more side effects or lead to drug-resistant HIV. Non-standard (NS) regimens can only be prescribed by specialists for complicated cases.
- Do not change ART regimens without clear medical indication. Unnecessary regimen changes spoil future treatment options.
- **1st Line regimens** are the best. Patients can remain on the same 1st line regimen for many years if they are fully adherent. All 1st line regimens:
    - Are easy to prescribe and easy to take
    - Have a low risk of side effects
    - Require no lab monitoring for toxicity
    - There are **6 different 1st line** regimens:
        - **3** are used for **initiating ART** depending patient characteristics (see Table 9 on Page 42). All of these are fixed-dose combinations: only 1 type of tablet has to be taken.
        - Move all patients with significant side effects to an alternative 1st line regimen without delay. Alternative regimens are chosen by **substituting** only the ARV responsible for the side effects.
- **2nd Line regimens** are a lifeline for patients who have confirmed treatment failure on 1st line regimen (usually due to poor adherence in the past). Moving from 1st to 2nd line ART is called **switching**. 2nd line regimens:
    - Contain a completely different class of ARVs (proteinase inhibitors)
    - Are more complicated to prescribe and take
    - Can have more side effects
    - There are **3 different 2nd line** regimens. The appropriate 2nd line regimen is determined by the 1st line regimen that the patient was taking when failing.
- **3rd Line regimen** is 'salvage therapy' and a last resort for patients failing on second line in spite of good adherence. This requires confirmation of drug resistant virus using genetic analysis in the lab. 3rd line can currently only be initiated on a study basis by a specialised expert ARV clinician.
    - Very expensive
    - Can have more side effects and be difficult to take

Key Facts for Providers and Patients (continued)

- From July 2011, **paediatric tablets** are used for children under 25kg (1st Line) or 35kg (2nd Line) to make prescribing, dispensing and taking of the drugs easier.
- All children who were on the new standard 1st line paediatric regimen (AZT / 3TC / NVP, Regimen 2) when they were under 15 years continue on the same regimen after their 15th birthday. These patients continue on Regimen 2A through adolescence and adulthood unless they develop toxicity or fail.

6.6.1 Classification of individual ARVs

- Main classification is based on **mode of action** against HIV replication
- Sub-classification is based on **biochemical structure** of the drug
- Only ARVs with the same dosing interval are available as fixed-dose combinations

Table 8: Classification of ARVs

Mode of action	Biochem. structure	Abbrev.	ARVs	Dosing interval
Reverse Transcriptase Inhibitors	Nucleosides	*NRTI*	**d4T, AZT, ABC**	***12**-hourly*
			**3TC**	***12**- or **24**-hourly*
			**TDF**	***24**-hourly*
	Non-Nucleosides	*NNRTI*	**NVP**	***12**-hourly*
			**EFV**	***24**-hourly*
Protease Inhibitors		*PI*	**LPV/r**	***12**-hourly*

6.6.2 Choosing ART regimen, formulation and dosage

- **Table 9** shows the standard ART regimens for Malawi
- Regimens are numbered for ease of reference:
  - Regimen 1 – 6 are 1st line regimens, including alternative 1st line regimens
  - Regimen 7 – 10 are 2nd line regimens
  - An “A” is added to the regimen number to signify adult formulations (e.g. Regimen 1A) and a “P” is added for paediatric formulations (e.g. Regimen 3P)
- Fixed dose combinations (FDC) are shown with a slash (e.g. AZT / 3TC / NVP)
- Combinations made up of different tablets are shown with + (e.g. AZT/3TC + EFV)

- **3TC (lamivudine)** is used as backbone in **ALL** 1st an 2nd line regimens because it is extremely well tolerated and remains active even with drug-resistant HIV is present
- **Paediatric / Adult Formulations:** Most regimens are suitable for children and adults and are available as both adult and paediatric strength tablets, but:
    - TDF may affect growing bones and is not given to children under 12 years
    - ABC/3TC is only supplied for 2nd line ART for children under 35kg (Regimen 9P: ABC/3TC + LPV/r). Children on 2nd line who pass the 35kg mark are moved to adult 2nd line regimen (Regimen 7A: TDF/3TC + LPV/r)
- **Start Regimen:** Regimen **1, 2** and **5** are used to initiate patients for the first time on ART. Table 9 shows which *start regimen* to select for which patient groups.
- **Initial Prescriber Level:** All MOH-certified PMTCT/ART providers are authorized to start any of the six 1st line regimens, but only experienced ART staff (certified Level 2 providers) are authorized to initiate 2nd line regimens. However, follow-up prescriptions for 2nd line regimens can also be made by Level 1 providers. See details in Section 6.6.3 on page 44
- **Starter Pack:** Regimens with NVP (regimen **1, 2** and **6**) need to be *phased in* to avoid potentially severe hepatitis or skin toxicity. During the first 2 weeks, the NVP-containing FDC is taken only once daily (before bed). The other 2 ARVs are taken in the morning to achieve 12-hourly dosing from the first day.
- *Starter packs* are dispensed as a 2-week supply of one pack of the triple ARV fixed-dose combination (with NVP) plus one pack of the other 2 ARVs in combination (without NVP).
- *Starter packs* are needed for all patients starting Regimen **1, 2** or **6:**
    - For the **first time** (new ART initiation)
    - After interrupting ART for more than **14 days** (re-initiation / re-start)
- *Starter packs* are **NOT** given when changing without interruption from an EFV-containing regimen (3, 4 or 5) to regimen 1, 2 or 6. This is because patients who have taken EFV excrete NVP at a faster rate.
- **Tail needed:** NVP and EFV remain in the body much longer than the other ARVs. Stopping any 1st line regimen due to side-effects (or due to patient's decision, etc.) therefore requires giving a 7-day '*tail'* of the other 2 ARVs in the regimen to avoid exposing the virus to only NVP or EFV, which would risk development of NVP- and EFV-resistant HIV and spoil future treatment options.
- However, do **NOT** give a *tail* in case of severe potentially life-threatening side effects (lactic acidosis, pancreatitis), but stop all ARVs immediately.
- **Contraindications:** Most contraindications are not absolute for a specific regimen: balance risks, benefits and alternatives. Usually, a suitable alternative regimen can be chosen from Table 9. The following conditions are absolute contraindications:
    - Patients who developed severe toxicity to any specific ARV (hepatitis or Stevens-Johnson Syndrome from NVP or EFV, severe anaemia from AZT, hypersensitivity from ABC) must **NEVER AGAIN** be given a regimen containing the responsible ARV.
    - Do not use **TDF**-containing regimens in severe **renal failure** (creatinine clearance <50ml/min)

- **Possible adverse event / If adverse event confirmed, use regimen:** Chose the appropriate alternative regimen from **Alternative 1** for patients with contraindications or with significant side-effects that did not improve within 2 months with symptomatic treatment. Use **Alt. 2** if **Alt. 1** can not be used due to previous toxicity or other specific contraindications.
- The appropriate 2nd line regimen depends on the 1st line regimen the patient was on when confirmed with treatment failure. Only certified **Level 2 ART providers** can initiate 2nd line.
- **Table 10** shows the number of tablets to be taken by children and adults once or twice per day.
- 10 weight-bands are used to determine the number of paediatric tablets to be given.
- Most paediatric formulations are **tablets** that can be crushed if necessary. The only exceptions are:
    - LPV/r for children **under 6kg** requires **liquid suspension** (80/20mg per ml)
    - LPV/r tablets must be **given whole** (not split or crushed)

Rationale for using Regimen 5A for pregnant and breastfeeding women

- 5A is the regimen of choice for universal ART for all HIV-infected pregnant and breastfeeding women (PMTCT 'option B+')
    - Other regimens with NVP can cause severe toxicity in patients with high CD4 counts. Other patient groups do not start ART with such high CD4 counts
    - TDF is more suitable than AZT for B+ women because it can not cause anaemia, which is a particular risk in pregnancy
    - TDF is more suitable than d4T for B+ women because long-term side-effects are less likely and these women will be on ART for longer than other patients (because they start ART earlier).

Use of EFV in women of reproductive age

- EFV has been suspected to increase the risk of birth defects. However, this remains inconclusive and, if any, the risk is very low. Take the following pragmatic approach:
    - Wait with ART initiation until 13 weeks of pregnancy and start on TDF/3TC/EFV (regimen 5A)
    - Don't change ART regimen if the woman became pregnant while on an EFV-containing ART regimen.[4]

[4] The potential risk of EFV is related to neural tube defects in the embryo and therefore only relevant to the first month of pregnancy. It is very unlikely that pregnancy will be identified in the first month and any regimen change would therefore not reduce the risk of birth defects.

Table 9: Standard ART 1st line (Regimen 1 - 6) and 2nd Line (Regimen 7 - 9)

Regimen	Paediatric Formulation	Adult Formulation	Used for ART initiation 'Start regimen'	Initial prescriber level	Starter pack	'Tail' needed	Contraindications	Possible adverse event	If confirmed, use regimen Alt 1	Alt 2
**1**	**d4T** 6mg / **3TC** 30mg / **NVP** 50mg	**d4T** 30mg / **3TC** 150mg / **NVP** 200mg	• Standard for adults aged 15 years or older<br>• *EXCEPT*: Patient groups listed for Regimen 5	1	Yes	Yes	• Jaundice / hepatitis	• Neuropathy<br>• Hepatitis, Skin rash<br>• Lipodystrophy[5], Lactic acidosis<br>• Treatment failure	2<br>3<br>5<br>7	5, 6, NS<br><br>NS<br>9
**2**	**AZT** 60mg / **3TC** 30mg / **NVP** 50mg	**AZT** 300mg / **3TC** 150mg / **NVP** 200mg	• Standard for children under 15 years	1	Yes	Yes	• Anaemia <8g/dl<br>• Jaundice / hepatitis	• Anaemia<br>• Hepatitis, Skin rash<br>• Lipodystrophy[5], Lactic acidosis<br>• Treatment failure	1<br>4<br>5<br>7	5, 6<br>3<br>NS<br>9
**3**	**d4T** 6mg / **3TC** 30mg + **EFV** 200mg	**d4T** 30mg / **3TC** 150mg + **EFV** 600mg	No	1	No	Yes	• History of psychiatric illness	• Neuropathy<br>• Hepatitis, Skin rash[6], psychiat. disorder<br>• Lipodystrophy[5], Lactic acidosis<br>• Treatment failure	2<br>1<br>5<br>7	5, 6, NS<br>NS
**4**	**AZT** 60 mg / **3TC** 30mg + **EFV** 200mg	**AZT** 300 mg / **3TC** 150mg + **EFV** 600mg	No	1	No	Yes	• History of psychiatric illness<br>• Anaemia <8g/dl	• Anaemia<br>• Lipodystrophy[5], Lactic acidosis<br>• Hepatitis, Skin rash [6], psychiat. disorder<br>• Treatment failure	3<br>5<br>2<br>7	5<br>9<br>NS<br>9
**5**		**TDF** 300mg / **3TC** 300mg / **EFV** 600mg	• Pregnant women<br>• Breastfeeding women<br>• Adults already on TB treatment	1	No	Yes	• History of psychiatric illness<br>• Renal failure<br>• Child under 12 years	• Renal failure<br>• Hepatitis, Skin rash [6], psychiat. disorder<br>• Treatment failure	lower dose<br>6<br>8	2<br>NS
**6**		**TDF** 300mg / **3TC** 300mg + **NVP** 200mg	No	1	Yes	Yes	• Jaundice/Hepatitis<br>• Renal failure<br>• Child under 12 years	• Renal failure<br>• Hepatitis, Skin rash<br>• Treatment failure	lower dose<br>5<br>8	2<br>NS

[5] Changing to **Regimen 5A** due to **lipodystrophy** requires that the patient has clear clinical signs of lipodystrophy and has been on ART for at least 2 years. Suspected lipodystrophy cases who have been on ART for less than 2 years need assessment and confirmation by an expert ART clinician before they can be moved to Regimen 5A.

[6] A mild transient skin rash is common after starting an EFV-containing regimen. This usually resolves by itself and is not usually a reason to interrupt or change regimen.

Regimen	Paediatric Formulation	Adult Formulation	Used for ART initiation 'Start regimen'	Initial prescriber level	Starter pack	'Tail' needed	Contraindications	Possible adverse event	If confirmed, use regimen Alt 1	Alt 2
7		TDF 300mg / 3TC 300mg + LPV/r 200/50	No	2	No	No	• Renal failure<br>• Child under 12 years	• Renal failure<br>• Nausea, vomiting<br>• Treatment failure	8<br>NS	 <br>(3rd line)
8		AZT 300mg / 3TC 150mg + LPV/r 200/50	No	2	No	No	• Anaemia <8g/dl	• Anaemia<br>• Nausea, vomiting<br>• Treatment failure	7<br>NS	 <br>(3rd line)
9	ABC 60mg / 3TC 30mg + LPV/r 100/25		No	2	No	No	• Abacavir hypersensitivity	• ABC hypersensitivity<br>• Treatment failure	8 or 7	 (3rd line)

Table 10: Standard pack sizes and dosing of Paediatric and Adult formulations used in standard 1st and 2nd line ART regimens

ARV	Tablets per tin: Paed.	Tablets per tin: Adult	3 – 3.9 kg AM	3 – 3.9 kg PM	4 – 5.9 kg AM	4 – 5.9 kg PM	6 – 9.9 kg AM	6 – 9.9 kg PM	10 – 13.9 kg AM	10 – 13.9 kg PM	14 – 19.9kg AM	14 – 19.9kg PM	20 – 24.9kg AM	20 – 24.9kg PM	25 – 29.9kg AM	25 – 29.9kg PM	30 – 34.9 kg AM	30 – 34.9 kg PM	35 – 39.9 kg AM	35 – 39.9 kg PM	40 kg + AM	40 kg + PM
d4t / 3TC	60	15 or 60	1	1	1	1	1 ½	1 ½	2	2	2 ½	2 ½	3	3	1	1	1	1	1	1	1	1
d4t / 3TC / NVP	60	15 or 60	1	1	1	1	1 ½	1 ½	2	2	2 ½	2 ½	3	3	1	1	1	1	1	1	1	1
AZT / 3TC	60	60	1	1	1	1	1 ½	1 ½	2	2	2 ½	2 ½	3	3	1	1	1	1	1	1	1	1
AZT / 3TC / NVP	60	60	1	1	1	1	1 ½	1 ½	2	2	2 ½	2 ½	3	3	1	1	1	1	1	1	1	1
ABC / 3TC	60	60	1	1	1	1	1 ½	1 ½	2	2	2 ½	2 ½	3	3	4	4	4 ½	4 ½				
LPV / r	120	120	*1ml*	*1ml*	*1.5ml*	*1.5ml*	2	1	2	1	2	2	2	2	3	3	3	3	2	2	2	2
EFV	90	30							0	1	0	1 ½	0	1 ½	0	2	0	2	0	2	0	1
TDF / 3TC		30																	0	1	0	1
TDF / 3TC / EFV		30																	0	1	0	1

6.6.3 Choosing regimen and time of starting in special situations

Table 11: Choosing ART regimen and timing of initiation in special situations

Condition	Timing of ART initiation	Age 6wks – 14yrs	Age 15 yrs +
**Anaemia (<8g/dl)**	• Within 7 days of diagnosis	d4T/3TC/NVP	d4T/3TC/NVP
**Active TB**	• Within 14 days of diagnosis<br>• TBT + ART can be started on the same day if the patient is stable<br>• Don't delay either TBT or ART	**Under 3 years**<br>AZT/3TC/NVP<br>**3 years +**<br>AZT/3TC+EFV	TDF/3TC/EFV
**Jaundice**	• Refer to District or Central Hospital<br>• After investigation and stabilisation	AZT/3TC+EFV	d4T/3TC + EFV
**1st trimester pregnancy**	• Start ART in 2nd trimester		TDF/3TC/EFV
**In labour (new HIV+)**	• As soon as possible		TDF/3TC/EFV
**Renal failure**	• Refer to District or Central Hospital<br>• Start within 7 days of diagnosis	AZT/3TC/NVP	AZT/3TC + EFV

Key Facts for Providers and Patients

- ARVs should be taken after the same number of hours every day (e.g. every 12 or every 24 hours). Most ART regimens can be taken in the morning, at noon or at night and it does not matter if they are taken before, after or with food.
- Missing a dose: what to do if a patient remembers to take his ARVs late? If the patient remembers:
    - **Less than half-way** to the next scheduled dose: take the missed dose immediately, and take the regular next dose at the normal time
    - **More than half way** to the next scheduled dose: skip the missed dose and take the regular next dose at the normal time
- Dispense ARVs only in the original sealed container. Never open containers to dispense hand-counted quantities of tablets separately
- Only the patient or his registered guardians/treatment supporter is allowed to collect ARVs
- In an emergency, patients are allowed to collect ARVs from any ART clinic in Malawi following special rules (see below)

6.7 ART prescription and dispensing

Rules for ARV dispensing

- All certified clinical PMTCT/ART providers are authorized to prescribe and dispense ART (Doctors, Clinical officers, Medical Assistants, Registered Nurses, Nurse/Midwife Technicians)
- Dispense ARVs only in the original sealed container. Never open containers to dispense hand-counted quantities of tablets separately.[7]
- Only the patient or his registered guardians/treatment supporter is allowed to collect ARVs.

Emergency dispensing to patients from another PMTCT/ART site

- In an emergency, patients are allowed to collect ARVs from any ART clinic in Malawi under the following conditions:
    - The patient must present an ART identity card or the health passport with ARV dispensing information
    - If in doubt about a patient's authenticity, confirm by calling the site where the patient is registered.
    - Document emergency ARV dispensing in the patient's health passport

[7] To prevent crumbling of tablets in plastic bags, etc;. to prevent mixing of different batches of drugs with different expiry dates; and to ensure hygiene and avoid contamination.

- ARV dispensed to patients registered at another site must be recorded in the *Emergency ARV Dispensing Register* (to be implemented in late 2011*)*
- Instruct patient to return to their ART clinic of registration as soon as possible to ensure the patient is not recorded as defaulter

Determining quantities to be dispensed and next appointment

- **Table 12** on page 47 shows the number of tablets to be supplied for appointment intervals of 2, 4, 8 or 12 weeks for the total number of tablets taken of each ARV per day (paediatric and adult formulations).
    - Use Table 10 to add up the 'total tablets taken per day' for each ARV contained in the regimen. For example: a child of 15kg on AZT/3TC/NVP (Regimen 2) takes **2½** paediatric tablets in the morning and **2½** tablets in the evening, adding up to **5** total tablets per day.
    - The *Actual number of tablets needed* is the minimum number of total tablets the patient needs to take home to cover the time to the next appointment. (Total tablets = tablets remaining from the previous visit **+** tablets newly dispensed). The number needed includes an extra 2-day supply to act as a safety-buffer. The total tablets must meet or exceed the *Actual number of tablets needed*.
    - Different ARVs come in tins of 30, 60, 90 or 120 tablets (see Table 10). Given that only full tins should be dispensed, the number of tablets needed is *rounded up* to multiples of full tins.
    - *Rounding up* may result in a considerable *over-supply.* For some regimens and dosages, perfectly adherent patients will be left with more than half a tin of ARVs at their next appointment. Explain this to the patient / guardian and emphasize the importance of keeping the next appointment.
    - The number of tablets expected to be used in the interval is shown for 'perfect adherence' (100%) and for 'good adherence' (95%-105%).
    - Calculate the number of tablets used by subtracting total tablets remaining at the current visit from total tablets available at the end of the previous visit.
- Give next appointment date at least 2 days before ARVs would be finished to allow for the safety buffer
- Take account of the weekly ART clinic schedule (e.g. Mondays + Wednesdays) when giving the next appointment. Usually, appointments are given for 2 weeks (starter pack), 4, 8 or 12 weeks.
- Patients initiating standard or alternative first line ART have to be reviewed clinically after 2 weeks if they have been given a starter pack / otherwise after 1 month and then every month for the first 6 months.
- Thereafter, stable and adherent patients can be given up to 12-week (3-month) appointments.
- In exceptional cases (e.g. international travel), up to 6 or even 12 months of ARVs can be dispensed
- Patients starting 2nd line ART have to be seen every 4 weeks for the first 6 months. Thereafter, patients who are stable and adherent to 2nd line ART can be given up to 8-week appointments
- Align dispensing of CPT with ART visits
- Push back appointment date to allow patients to use up accumulated 'hanging' tablets, e.g. give an appointment after 5 instead of 4 weeks

Table 12: Quantity of ARVs to be supplied for visit intervals from 2-12 weeks for different daily doses

> ***Note:*** *supply and consumption must be calculated <u>separately for each component</u> in the regimen. Example: separate calculation for AZT/3TC and AZT/3TC/NVP making up a starter pack of Regimen 2*

Dispensing interval	Total tabs taken per day	Supply needed							Total tabs **USED** in interval - Adherence	
		Actual	*Multiples of full tins*							
			**Tins of 60**		**Tins of 90**		**Tins of 120**		**Perfect**	**Good**
		tabs *	tabs	**tins**	tabs	**tins**	tabs	**tins**	100%	95% – 105%
**2 weeks**	1	***16***	60	**1**	90	**1**			14	*14 – 14*
	1 ½	***24***	60	**1**	90	**1**			21	*20 – 22*
	2	***32***	60	**1**	90	**1**			28	*27 – 29*
	2 ½	***40***	60	**1**					35	*34 – 36*
	3	***48***	60	**1**					42	*40 – 44*
	4	***64***	120	**2**					56	*54 – 58*
	5	***80***	120	**2**					70	*67 – 73*
	6	***96***	120	**2**					84	*80 – 88*
**4 weeks**	1	***30***	60	**1**	90	**1**			28	*27 – 29*
	1 ½	***45***			90	**1**			42	*40 – 44*
	2	***60***	60	**1**	90	**1**			56	*54 – 58*
	3	***90***	120	**2**			120	**1**	84	*80 – 88*
	4	***120***	120	**2**			120	**1**	112	*107 – 117*
	5	***150***	180	**3**					140	*133 – 147*
	6	***180***	180	**3**			240	**2**	168	*160 – 176*
	8	***240***	240	**4**					224	*213 – 235*
	9	***270***	300	**5**					252	*240 – 264*
**8 weeks**	1	***58***	60	**1**	90	**1**			56	*54 – 58*
	1 ½	***87***			90	**1**			84	*80 – 88*
	2	***116***	120	**2**	180	**2**			112	*107 – 117*
	3	***174***	180	**3**			240	**2**	168	*160 – 176*
	4	***232***	240	**4**			240	**2**	224	*213 – 235*
	5	***290***	300	**5**					280	*266 – 294*
	6	***348***	360	**6**			360	**3**	336	*320 – 352*
	8	***464***	480	**8**					448	*426 – 470*
	9	***522***	540	**9**					504	*479 – 529*
**12 weeks**	1	***86***	120	**2**	90	**1**			84	*80 – 88*
	1 ½	***129***			180	**2**			126	*120 – 132*
	2	***172***	180	**3**	180	**2**			168	*160 – 176*
	3	***258***	300	**5**					252	*240 – 264*
	4	***344***	360	**6**					336	*320 – 352*
	5	***430***	480	**8**					420	*399 – 441*
	6	***516***	540	**9**					504	*479 – 529*

** Actual tabs needed includes a 2-day safety-buffer*

6.8 Starting ART

Key Facts for Providers and Patients

- ART does not cure HIV infection
- ART stops the HIV virus from multiplying, which allows the body's defence system to recover
- The virus will 'wake up' as soon as ART is interrupted and it will learn how to evade ART. This means that ART may no longer work for this patient
- Once started, ART must be taken every day for life
- Patients on ART can still pass on HIV to other and must use condoms
- From July 2011, all patients (incl. pregnant women tested on the same day of starting ART) need a confirmatory HIV antibody test to rule out any possibility of mix-up of test results or fraudulent access to ART:
    - Either at enrolment into pre-ART follow-up, or before starting ART if the confirmatory test was not done in pre-ART.
    - Children under 12 months starting ART with a positive DNA-PCR do not need another confirmatory test before starting ART, but all need a confirmatory rapid antibody test at age 12 and 24 months (see **Section 6.1** on **page 9**).
- ARVs must not be dispensed outside of certified PMTCT/ART facilities and must not be shared, sold or passed on to others
- Unused ARVs (e.g. after a patient's death) should be returned to the clinic for proper disposal
- Bring back any remaining ARV tins and tablets at every clinic visit to allow the provider to count them
- Patients who are late for their ART appointment will be actively followed from the clinic (home visit, phone, guardian)
- All patients are asked for consent for active follow-up at the time of starting ART. Patients can withdraw consent at any time
- A small number of patients on ART develop side-effects
    - Most side-effects are mild and disappear while ART is continued
    - Some side-effects require a regimen change
    - EFV can cause bad dreams and dizziness in the first few weeks of treatment, but this usually disappears by itself and it is important to continue treatment
    - Very few patients develop serious side effects. Stop all drugs immediately and present to the hospital if any of the following conditions are seen:
        - Yellow eyes / hepatitis
        - Severe stomach pain and vomiting
        - Shortness of breath
        - Severe skin rash with blisters, involving eyes, mouth or genitals

6.8.1 Record keeping

- Fill ART patient cards immediately when ART eligibility is established (PMTCT/ART nurse or clinician). For this reason, keep blank ART treatment cards at OPD, on the wards, etc.
- Dispensing of ARVs must be recorded on the patient treatment cards
- The ARV drug register will no longer be used.
- Complete ART treatment cards before giving out the first supply of ARVs
- Patients should only be entered in the ART register after receiving their supply of ARVs

6.8.2 Confirming HIV infection

- From July 2011, all patients need a confirmatory HIV antibody test to rule out any possibility of mix-up of test results or fraudulent access to ART. This also applies to pregnant and breastfeeding women who have been tested on the same day of starting ART:
    - Either at enrolment into pre-ART follow-up,
    - Or before starting ART if the confirmatory test was not done in pre-ART.
    - Children under 12 months starting ART with a positive DNA-PCR do not need another confirmatory test before starting ART, but all need a confirmatory rapid antibody test at age 12 and 24 months (see **Section 6.1** on **page 9**).
- Do not delay ART initiation if HIV test kits are not available for the confirmatory test, but do confirmatory test at the next scheduled visit as soon as testing is available

Testing protocol for confirmatory HIV test

- Confirmatory testing should be done by a dedicated HTC counsellor attached to the HCC / ART clinic. Ensure that all Quality Assurance protocols for HTC (proficiency testing, quality control) are being followed
- **Use only the first rapid test** (Determine) for confirmatory HIV testing:
- Confirmatory test is **positive**:
    - Record confirmatory HIV test results on pre-ART or ART patient card.
    - **Do not** record confirmatory HIV testing in the **MOH HTC register** (2007 version)
    - Enrol in pre-ART / Start ART
- Confirmatory test is **negative**:
    - Send patient to referral hospital to repeat regular HIV testing and for review by an experienced ART clinician. If test result is still inconclusive, do viral load (VL) and send specimen to CHSU reference lab

6.8.3 Preparing the patient for ART

- Patients who are clinically stable should start ART no later than 7 days after being found eligible.
- Pregnant women should be offered to start ART on the same day.
- Confirm that patient (or parent/guardian if patient is <15 years) understands implications of ART and is committed to lifelong adherence.

- Identify long-term treatment support for patients who are unable to take responsibility for their own treatment (persons with mental disability or drug-addiction, etc.)
- Ask all patients to attend the initial group counselling and/or the ART initiation visit with a named guardian/treatment supporter
    - If the patient is unable to identify a suitable guardian, another patient can be used as the named treatment supporter

Mandatory IEC procedures when starting ART

- All patients must receive individual counselling at ART initiation
    - Women starting ART in labour can receive individual ART counselling after delivery
- In addition, all patients should attend an ART group counselling session. Recommended practice:
    - Attended group counselling between 1 to 5 days before the day of ART initiation
        - But: group counselling can be attended on the same day as ART initiation to avoid delay beyond 7 days
        - Pregnant women may attend the group counselling at the next scheduled visit to ensure they can start ART on the same day
    - Ask patients to attend with their named guardian (also see Section 6.8.2 on page 49)

ART group counselling

- Use standard ART flip chart (to be updated)
- Share "Key Facts for Providers and Patients"

Individual ART counselling

- Confirm that patient and guardian have understood the following:
    - Commitment to lifelong adherence
    - Dosage and interval of taking ARVs
    - Potential side-effects
    - Date of next appointment

6.8.4 Baseline and routine lab investigations

- The national program does not support baseline lab investigations before starting ART or routine investigations for ART toxicity
- Routine scheduled CD4 monitoring of patients on ART is not supported by the national program
- Use targeted investigations if clinically indicated
- Scheduled VL monitoring will be rolled out (see Routine scheduled viral load (VL) monitoring on page 56)

6.8.5 Combining ART and TB treatment

Key Facts for Providers and Patients

- Each year 27,000 (3%) of the 900,000 HIV infected Malawians develop TB
- 2 out of every 3 TB patients in Malawi are HIV infected
- The risk of developing TB remains high for the first 6 months on ART
- Most HIV patients with TB do not have typical TB symptoms (productive cough) and most are sputum smear negative
- HIV infected TB patients must start ART and TB treatment as soon as possible. The long term outcome is poor if only one treatment is taken.
- There is no problem with taking ART Regimen 5A at the same time as TB treatment

- Certain combinations of ARVs and TB drugs increase the risk of side-effects or reduce each other's effectiveness (due to accelerated excretion)
- The following table shows the relevant interactions. The colours give:
    - **Green:** Combination causes no problems
    - **Yellow:** Combination causes usually no problems but monitor patient for possibly increased side-effects or adjust dosage as shown
    - **Red:** Do not combine without specialist advice

Table 13: Relevant interactions between ARVs and TB drugs

	INH	RH	Streptomycin	EMB	PZA
**TDF**	OK	OK	renal toxicity	OK	OK
**AZT**	OK	OK	OK	OK	OK
**3TC**	OK	OK	OK	OK	OK
**d4T**	neuropathy	OK	OK	OK	OK
**ddI**	neuropathy	OK	OK	OK	OK
**EFV**	OK	OK	skin rash	OK	hepatitis
**NVP**	skin rash	start NVP full dose, hepatitis	skin rash	OK	hepatitis
**ABC**	OK	OK	OK	OK	OK
**LPV/r**	OK	major dose adjustment	OK	OK	OK

6.9 Continuing ART

6.9.1 Confirming adherence to appointment

- On the patient card, look at the *Next Appointment Date* given at the previous visit to confirm that the patient is not late.
- The patient is likely to have missed doses if s/he is more than 2 days late. Compare and validate with *Pill Count* and the reported number of *Doses Missed.*

6.9.2 Monitoring height and weight

- Record current weight (and height for children under 18 years).
- Look for weight changes compared with previous measurements. Patients are expected to normalize their weight in the first 6-12 months on ART.
- Classify nutrition status based on weight for height (children) or BMI (adults).
- Investigate any consistent weight loss over 2 or more consecutive visits. Remember to confirm that the scale is correctly calibrated and any heavy clothing was removed.

6.9.3 Monitoring for new HIV-related diseases and drug side-effects

- Use the standard clinical monitoring checklist for HIV patients to actively screen for symptoms of HIV-related diseases and/or drug side effects.
- Use the syndromic guide shown in **Table 14** (page 58) to identify the likely cause of symptoms and to choose the right primary and secondary management.
- A symptom that could be caused by an HIV-related disease or by a side-effect is more likely a side-effect if it started or worsened after the start of medication.
- Circle all symptoms that are likely drug side-effects on the patient card.
- Change the ART regimen if medically indicated (see below).
- Write any new HIV-related disease under *Notes* on the back of the patient card.

6.9.4 Indications for interrupting or stopping ART

- ART should be stopped in patients with chronic poor adherence. Stopping should be considered if 3 intensive counselling sessions have failed.
- ART should be stopped abruptly and completely if any of the following severe side-effects are suspected:
    - Lactic acidosis
    - Pancreatitis
- Stopping ART in patients with less severe toxicity against EFV or NVP (skin rash, psychiatric effects) should be done by giving a ‘tail’ of the other 2 ARVs for 7 days to prevent ‘monotherapy’ due to the long half-life of NVP and EFV (see **Table 9** on page 42)

6.9.5 Selecting regimen and formulation for continuation

- Don't change regimen without clear medical indication. Unnecessary changes spoil future treatment options.

Do NOT change ART regimen:

- If a woman became pregnant while on an ART regimen that contains EFV [8] (Regimen 3, 4 or 5. Also see page 40 for further explanation)
- All children who were on the new standard 1st line paediatric regimen (AZT / 3TC / NVP, Regimen 2) when they were under 15 years continue on the same regimen after their 15th birthday. These patients continue on Regimen 2A through adolescence and adulthood unless they develop toxicity or fail.

Change dosage and formulation:

- Review current weight for children and adjust dosing if necessary. Children on 1st line regimens change to adult formulation and dosage when their weight is over 25kg (see **Table 10** on page 43).
- Start a new *ART Patient Card – Adult ARV Formulations* for children who change from paediatric to adult ARV formulation. File together with the old card in the same polythene sleeve.

Change ART Regimen:

- Use **Table 9** on page 42 to select the appropriate alternative regimen for patients with significant side-effects that did not improve within 2 months with symptomatic treatment.
- Children who were on paediatric 2nd line regimen (Regimen 9P) routinely change after their 12th birthday to standard adult 2nd line regimen (Regimen 7A). This is to reduce the pill burden while continuing on an equally effective regimen.
- Add any new regimen to the *ART Regimens* history section on the card header and specify any non-standard regimen here.

6.9.6 Routine TB screening

- Screen all patients at each visit for signs of active TB using 4 standard screening questions
  - Cough of any duration
  - Fever
  - Night sweats
  - Weight loss / failure to thrive / malnutrition
- Classify screening outcome as follows:
  - ***TB not suspected*** if none of the 4 signs are positive. In this case, the patient is very unlikely to have active TB.
  - ***TB suspected*** if one or several of the 4 signs are positive and thoroughly investigate further (full clinical exam, sputum for AAFB, chest x-ray, fine needle aspirate, etc.)

---

[8] The potential risk of EFV is related to neural tube defects in the embryo and therefore only relevant to the first month of pregnancy. It is very unlikely that pregnancy will be identified in the first month and any regimen change would therefore not reduce the risk of birth defects.

- *TB confirmed* if the patient has a current confirmed episode of TB (clinical or lab diagnosis). Always confirm if the patient is currently taking TB treatment – initiate TB treatment without delay or provide intensive adherence support. Classify ***on TB treatment*** or ***not on treatment***

6.9.7 Achieving optimal dose adherence

Key Facts for Providers and Patients

- Patients must take more than 95% of doses at the prescribed interval for life to prevent HIV drug-resistance. Repeated skipping of individual doses or repeated longer interruptions inevitably lead to development of HIV drug-resistance.
- **Example:** HIV drug-resistance will develop if a patient on Regimen 5A (TDF/3TC/EFV) continues to skip more than 3 tablets in every 8 week period.

- Ask at every visit:
    - Have you had any problems taking your ARVs? Can you explain what problems you have had?
    - Were there any days when you did not manage to take all of your tablets at the right time? (Weekends, weekdays, mornings, evenings?)
    - Additionally for children: Who is responsible for giving ARVs? Who stands in for the guardian if s/he is away? How do you give the tablets?
- Remind patients of the importance of perfect adherence at every clinic visit
    - Initial ART counselling
    - Follow-up group counselling
    - Individual counselling if any sign for poor adherence
- Give practical advice how to achieve optimal adherence:
    - Build ARVs into the daily routine (e.g. before washing the face, after evening meal)
    - Ask family or friends to remind
    - Set a daily alarm on the cell phone
    - Keep a 'drug diary' and mark every tablet taken
- Encourage honest dialogue. Avoid giving the impression of 'policing' the patient. Work with patients to help them achieve good adherence.
- Poor adherence always has valid reasons and most can be resolved: vomiting, transport problems, domestic problems, (perceived) side effects, psychological problems, wrong understanding, etc.

6.9.8 Keeping track of the number of months since ART initiation

- This is necessary to determine when blood samples for routine VL monitoring are to be drawn

- Calculate and document on the ART patient card the number of months since the patient first started ART. Simply calculate the number of months since first ART initiation, ignoring any potential gaps (periods of stopping / defaulting).

6.9.9 Monitoring for treatment failure / HIV drug resistance

Key Facts for Providers and Patients

- ARV drug resistance starts gradually and the virus will still be partly suppressed by for many months. Emerging drug-resistant virus does not cause any immediate clinical symptoms.
- HIV will grow resistant to more and more ARVs if a patient continues to take a failing ART regimen for several months. Accumulated multiple resistance can make it difficult to find a second line regimen that still works.
- HIV drug resistance usually affects different ARVs of the same class.
- ***Example:*** HIV that has grown resistant to EFV will also be resistant to NVP, even if the patient has never taken NVP before.
- Drug resistant virus can be transmitted to other people.
- ***Example:*** About 5% of Malawians who got newly infected with HIV in 2009 acquired virus with some level of drug-resistance against d4T/3TC/ NVP.

Clinical suspicion and diagnosis of treatment failure

- **Suspect ART failure** if both of the following clinical conditions are met:
  - On ART for 12 months or more
  - New WHO clinical stage 3 or 4 condition
- For all **suspected ART failure** cases, look for indications for poor adherence in the last 6 months
  - Adherence was good:
    - Do a VL or refer to have this done immediately.
  - Adherence was questionable:
    - Start intensive adherence support
    - Do VL after 3 months if adherence was satisfactory.
- See page 56 for an explanation of how to interpret VL results.

Routine scheduled viral load (VL) monitoring

Key Facts for Providers and Patients

- VL is the best measure for the level of progression of HIV infection
  - VL = number of viral particles per ml of blood.
  - More virus ⇨ Faster destruction of CD4 cells ⇨ More severe immunosuppression
- VL is done using an advanced lab method (RNA-PCR) on a blood sample.
- VL are costly
- VL can be done from:
  - Blood (plasma): Transport in cooler box to lab within 24 hours.
  - Dried blood spot (DBS): Transport in plastic bag with desiccant at ambient temperature, sample viable for 3 months or more
- VL are required to confirm suspected ART failure (clinical and/or CD4-based)
- Routine VL monitoring will be scaled up gradually from 2011 to 2015.
- The recommended routine VL schedule is designed to detect ART failure early while avoiding unnecessary tests to save cost:
  - Patients harbouring drug-resistant HIV when starting ART will be found with a VL after **6 months** on ART. Such patients may have been infected with drug-resistant HIV or may have developed it after taking sdNVP. Otherwise, a high VL at 6 months is an important early sign for poor adherence.
  - After that, patients who are adherent and clinically well have a low risk of ART failure. Therefore, routine follow-up VLs are done at **2 years**, **4 years**, **6 years**, **etc**. after ART initiation.
  - Do additional VLs outside of this schedule for patients with suspected ART failure

When to do VL

- **Targeted**: Mandatory before starting 2nd line ART to confirm suspected ART failure
- **Routine** VLs are scheduled at specific times after ART initiation:
  - At 6 months
  - At 2 years, at 4 years, and every 2 years thereafter

Interpretation of VL and action

VL undetectable (threshold lab specific):

- No specific action required

VL detectable:

1) Start intensive adherence counselling and monitoring

- Monthly appointments with pill counts and adherence counselling
- Use ART diary, alarm clock, etc.

2) Assess adherence after 3 months:

  - Adherence in the last 3 months was not good:
    - Intensify adherence counselling (social services, find effective guardian / CBO / FBO, substance abuse?)
    - Re-assess
    - Consider stopping ART if all fails
  - Adherence in the last 3 months was good: Repeat VL:
    - **VL less than 5,000 copies/ml**: continue intensive adhere support
    - **VL 5,000 copies/ml or more**: Treatment failure confirmed, start 2nd Line regimen, continue checking VL according to routine schedule.

6.9.10 Updating follow-up outcome

- Regularly review all patient cards and keep an appointment register to identify patients who are overdue for their appointment as soon as possible.
- Try to contact the patient or the named guardian by phone or by home visit from 2 weeks after the missed appointment. Confirm from ART Patient Card that consent was given for home visit.
  - Patient is alive: counsel to return to the clinic as soon as possible and continue treatment
  - Patient has stopped, died or transferred out: update outcome and date of outcome on patient card and in register
- ‘Defaulter’ tracing is expensive and time-consuming. Prioritize patients on ART and HCC patients who are eligible to start ART.
- Loss to follow-up:
  - Patient is overdue for the appointment and is not known to have stopped ART, died or transferred to another facility
  - Classify as ‘defaulted’ if the patient has run out of ARVs 2 or more months ago (based on the number of tins given at the last visit)
- Patients who are alive but known to have stopped ART (for any reason) should be classified as ‘stopped’ and not as ‘defaulted’
- Ask guardians to notify the clinic if an ART patient has died. Bring back the patient health passport and/or ART ID and any remaining ARVs.

Table 14: Symptom-based identification and management of ARV side-effects

Body pains, weakness

**Cause** (in order of likelihood)	**Diagnosis**	**Primary Management**	**Secondary Management**
AZT, 3TC	Severe anaemia: Hb <7 g/dl	Stop AZT, consider transfusion	Substitute AZT, continue ART without gap
d4T, ddI, AZT	Lactic acidosis (LA): shortness of breath, nausea<br>Serum lactate: suspect: 2-5 mmol/l, confirmed: ≥5 mmol/l	*Any suspected LA:* Stop all ART immediately<br>IV fluids, treat at hospital	Do not re-start ART before lactic acid <2mmol/l<br>Can re-start ART with AZT after suspected LA<br>Never give d4T, AZT, ddI after confirmed LA<br>Can use ABC or TDF containing regimen

Fever

**Cause** (in order of likelihood)	**Diagnosis**	**Primary Management**	**Secondary Management**
Onset independent of drugs: Bacteraemia, malaria	FBC, MPs, blood culture, urine dipstick		
Onset within 8 weeks of starting drugs:<br>ABC, NVP	ABC/NVP hypersensitivity: Body pains, vomiting, diarrhoea, abdominal pain, sore throat, cough, shortness of breath, rash, jaundice	Any suspected hypersensitivity: Stop all ART immediately, treat at hospital	Do not re-start before symptoms have resolved<br>Never use NVP or ABC again<br>Replace NVP with EFV and ABC with TDF

Slimming: Cheeks, forearms, buttocks, legs (often with prominent veins) Fattening: Back of the neck ('buffalo hump'), chest, stomach, and waist

**Cause** (in order of likelihood)	**Diagnosis**	**Primary Management**	**Secondary Management**
d4T, ddI, AZT, LPV/r, 3TC, HIV	Lipodystrophy<br>(induced by ART and/or HIV-associated)	Reassure patient<br>Substitute likely causative ARV	

Skin Rash

**Cause** (in order of likelihood)	**Diagnosis**	**Primary Management**	**Secondary Management**
Onset before starting drugs:<br>Seborrhoeic dermatitis<br>(“bumpy itch”)	HIV-related skin rash	Adults only: Promethazine 25 mg 12-hourly<br>Adults or children (lower dose):<br>Chlorphenamine (Piriton) 10 mg 8-hourly<br>Calamine lotion	Consider scabies, etc.
Onset within 8 weeks of starting drugs:<br>NVP, ABC, Cotrimoxazole, EFV	Mild hypersensitivity<br>Macular/popular rash not involving mouth, eyes, and genitalia<br>No fever, body pain, weakness, etc.	Continue on half dose NVP (if on NVP starter pack) for further 2 weeks<br>Adults only: Promethazine 25mg 12-hourly<br>Adults or children (lower dose):<br>Chlorphenamine (Piriton) 10 mg 8-hourly<br>Calamine lotion	If no improvement on half dose NVP, stop NVP<br>Substitute to EFV once rash has resolved.<br>If patient unable to take EFV, consult with ART specialist for alternatives

Upper GI symptoms: Nausea, vomiting

**Cause** (in order of likelihood)	**Diagnosis**	**Primary Management**	**Secondary Management**
AZT, LPV/r, d4T, 3TC	Lactic acidosis ? (see ‘*Body pains and weakness*’)<br>Jaundice? (see ‘Yellow eyes’)	Adults only: Promethazine 25 mg up to 12-hourly<br>Adults or children (lower dose):<br>Chlorphenamine (Piriton) 10 mg up to 8-hourly-oral rehydration solution(ORS)	If no lactic acidosis: try to continuing the same ART regimen<br>If persistent, substitute

Lower GI symptoms: Diarrhoea, lower abdominal pain

**Cause** (in order of likelihood)	**Diagnosis**	**Primary Management**	**Secondary Management**
Onset before ART initiation:<br>HIV-induced	Stepwise empirical treatment	Stepwise empirical treatment of chronic HIV diarrhoea (see page 14)	
Onset within 6 weeks of starting drug:<br>LPV/r, AZT, d4T, 3TC	Drug toxicity	For adults only: Loperamide 2 mg 8-hourly (mainly for LPV/r induced diarrhoea)	Try to continue same ART regimen<br>If persistent substitute

Severe upper abdominal pain, nausea and vomiting

**Cause** (in order of likelihood)	**Diagnosis**	**Primary Management**	**Secondary Management**
d4T, ddI, 3TC	Pancreatitis<br>Serum amylase >1.5 times above upper normal limit	Stop all ART immediately<br>Treat at hospital	Restart ART after complete remission<br>Use TDF- or AZT-containing regimen
NVP, EFV, alcohol, viral hepatitis	Acute fulminant liver failure<br>Liver function tests	Discontinue ART immediately<br>Treat at hospital<br>Identify cause and manage accordingly	Never re-start NVP or EFV if this was the suspected cause<br>Reinitiate ART one month after jaundice is resolved, and LFT <2.5 of upper normal limit

Yellow eyes

**Cause** (in order of likelihood)	**Diagnosis**	**Primary Management**	**Secondary Management**
Viral hepatitis, alcohol, NVP, INH, EFV, ABC, severe malaria,<br>cancer	LFT and ultrasound scan to differentiate:<br>Viral hepatitis, cirrhosis, drug hepatitis, primary liver cancer, metastases	Discontinue ART immediately if jaundice develops on ART<br>Identify cause and manage accordingly (LFT, ultrasound, hepatitis serology)	Never re-start NVP or EFV if this was the suspected cause<br>Re-initiate ART 1 month after jaundice has resolved and LFT <2.5 times upper normal limit

Swollen face and eyelids, particularly in the morning/tiredness, too much or too little urine

**Cause** (in order of likelihood)	**Diagnosis**	**Primary Management**	**Secondary Management**
Onset before starting drugs<br>HIV, diabetes, hypertension	Confirm nephropathy with serum creatinine	Identify cause and manage accordingly	Adjust ART dosage according to creatinine clearance
Onset within 1 year of starting drugs:<br>TDF, streptomycin	Confirm nephropathy with serum creatinine	Admit to hospital<br>Substitute TDF to AZT (or d4T) without gap<br>Stop streptomycin	Adjust ART dosage according to creatinine clearance

Drowsiness, confusion, nightmares

**Cause** (in order of likelihood)	**Diagnosis**	**Primary Management**	**Secondary Management**
EFV	Neuropsychiatric EFV toxicity	Usually disappears by itself without the need to discontinue ART<br>Take EFV before bed	If intolerable replace EFV with NVP

Leg pain, numbness or burning, inability to walk

**Cause** (in order of likelihood)	**Diagnosis**	**Primary Management**	**Secondary Management**
Onset before starting drugs:<br>HIV neuropathy<br>Onset or worsening after starting drugs<br>d4T, ddI, INH, vincristine<br>Onset independent of drugs<br>Alcohol, diabetes	Mild peripheral neuropathy (PN): no sleep disturbance	Amitryptiline 25 mg nocte for 4 weeks<br>Pain control using WHO analgesic ladder	If no improvement after 4 weeks: stop amitryptilline, continue analgesics<br>Substitute d4T with AZT or TDF without gap
	Moderate PN: sleep disturbance	Both: Substitute d4T with AZT or TDF without interruption of ART (gap)<br>WHO analgesic ladder	
	Severe PN: severe pain, muscular weakness		

6.9.11 Immune reconstitution inflammatory syndrome (IRIS)

Key Facts for Providers and Patients

- A small number of patients may get worse in the first 6 months after starting ART
- The most common causes for this are (in the order of likelihood):
    - Undiagnosed / untreated OI, mainly TB
    - Poor adherence to ART
    - Drug-resistant TB (if on TB treatment)
    - IRIS
- IRIS is an over-aggressive response of the body's defence system caused by a sudden recovery on ART
- IRIS appears as a severe bout / worsening of an OI:
    - TB
    - Cryptococcal meningitis
    - Herpes zoster
    - KS
    - Hepatitis
- IRIS should only be considered if the more common causes for worsening have been ruled out
- Patients who start ART with very advanced AIDS are at a higher risk of developing IRIS

Management of IRIS

- Confirm that ART is actually taken as prescribed
- Continue ART if ART toxicity has been ruled out as the underlying cause
- Treat the OI
- Consider TB treatment failure if worsening occurs after more than one month on TB treatment
- Admit severe cases to hospital
- Seek specialist advice on whether NSAIDs and/or prednisolone should be given

6.10 Management of labour and delivery

Ascertain HIV status

- Review HIV testing page in health passport on admission
- Offer PITC[9] if never tested or tested negative more than 3 months ago

Provide ART

- Mothers already on ART: continue the same ART regimen at regular prescribed intervals
- HIV positive mothers not yet on ART / who interrupted / stopped ART: emergency ART initiation
  - Start lifelong TDF/3TC/EFV (Regimen 5A) as soon as possible, during labour or after delivery
  - Deliver individual ART counselling and IEC before discharge

Reduce obstetric risk of HIV transmission

- Use a partogram to allow early detection and management of prolonged labour
- Artificial rupture of membranes (ARM) increases the risk of HIV transmission
  - ARM is not indicated if labour is progressing well
  - If prolonged labour due to poor uterine contraction: perform ARM at ≥6cm cervical dilation and augment with oxytocin (pitocin)
- Do not perform routine episiotomy except for specific obstetric indications (e.g. vacuum extraction)
- Avoid frequent vaginal examinations
- Do not 'milk' the umbilical cord before cutting

6.11 Newborn and postnatal care

- Follow regular post natal care
- Do not suction with a naso-gastric tube unless there is meconium-stained liquor
- Immediately after birth, wipe the baby dry with a towel to remove maternal body fluids
- Give BCG and oral polio vaccine after birth to all babies born to HIV infected mothers (as for all other infants)

6.12 Initiating integrated mother/infant follow-up

- Ensure continued follow-up for HIV infected mothers and babies
- Enrol baby in HCC before discharge from post natal ward:
  - Fill Exposed Child patient card, enter in HCC register

[9] There is no general time limit for offering PITC. Consider that other important interventions such as C-section or tubal ligation are also offered with emergency counseling very late in labour.

- Mothers on ART before delivery:
  - Confirm next ART appointment
  - Synchronise mother's ART appointment with baby's first HCC visit. Aim for HCC enrolment at post-natal visit or first vaccination visit.
- Mother initiated ART in labour:
  - Fill ART patient card and enter in ART register
  - Give regular 4 week ART + HCC appointment
- If mother wants to continue HCC and ART at another facility:
  - Record 'transfer out' in HIV clinic and ART register and give mother HIV clinic and ART patient card

6.12.1 Dispensing infant NVP prophylaxis

Key Facts for Providers and Patients

- NVP syrup is given to all babies born to HIV infected mothers
  - NVP syrup shields the baby from HIV infection during the most risky time
  - Give NVP syrup to the baby 24-hourly for the first 6 weeks of life
  - All babies should take NVP syrup for the same duration regardless of the mother's ARV regimen and regardless if the mother was taking ARVs at all
- Store NVP syrup bottles and dosing syringe in a place that is dark, cool, clean and dry and out of the reach of children
- Demonstrate how to draw up 1.5ml of syrup in the syringe
- Hand out one example syringe where the 1.5ml line has been marked with a pen
- Squirt the syrup in the back of the infant's mouth between the cheek and the gum to ensure it gets swallowed (use cup to demo)
- Rinse the dosing syringe carefully with clean water after every use and let dry.
- Bring back to the health facility at the 6 week vaccination visit all NVP bottles (whether used or unused). The nurse will check if the right amount was used.

Prescription and dispensing of NVP prophylaxis

- When to dispense NVP syrup for infant prophylaxis to take home:
  - At ANC (or maternity) as soon as the mother is known to be HIV-infected.
  - Unopened bottles of NVP syrup have a long shelf-life. Therefore never delay dispensing until later in pregnancy.
  - Ask at every following visit if the NVP syrup and the syringes are still available. Replace without delay any items that may have been lost or spoilt.
- Dispense **3** x 25ml-bottles of NVP syrup (with dosing syringe included)

Dosing

- The dose of NVP syrup remains the same for the whole 6 week period – do not change the dose according to age or body weight, etc.

Table 15: Dosing of NVP syrup for infant prophylaxis

Birth weight	NVP syrup (10mg per ml)
**2500g or less**	**1.0 ml** 24-hourly
**Over 2500g**	**1.5 ml** 24-hourly

Timing and duration

- Start giving NVP syrup to the baby as soon as possible after birth. The earlier the start, the more effective.
- NVP syrup can be started anytime between birth and 4 weeks of age if the mother presents late. Starting later is not effective.
- Stop giving NVP syrup when the infant is 6 weeks old. The infant will receive less than 6 weeks of prophylaxis if NVP syrup has been started late.

6.13 Post exposure prophylaxis (PEP)

Key Facts for Providers and Patients

- HIV infection can be prevented after a high risk contact with body fluids from an HIV infected person
    - Remove immediately as much as possible of the body fluid
    - Immediately give a 3-day supply of ARV prophylaxis (PEP) and start taking it as soon as possible.
    - Do assessment of risk and HIV test as soon as possible. Continue a 30-day course of ARV prophylaxis (PEP) if exposure is classified as 'risk' and exposed person is HIV negative
- PEP, if taken correctly, reduces the risk of infection by 80%
- ARVs taken for PEP are usually well tolerated.
    - Mild side-effects: Vomiting
    - Severe side effects: Anaemia (stop PEP and go to clinic)
- Keep ARVs for PEP at maternity for 24-hour access and at other well-advertised locations in every facility
- Offer STI treatment and emergency contraception, when indicated, for rape victims accessing PEP
- The risk of getting infected may be high or low, depending on the type of substance and contact. However, PEP should always be started if there is a possible risk of transmission (see classification in **Table 16** on page 67)

Classification of risk

- Use **Table 16** to find out if the exposure is a possible risk for infection
- Obtaining a new HIV test from the source person can help to reassure that the risk is low, but PEP should still be given if the test result is negative as the person could be newly infected himself and may be in the window period.

Table 16: Classification of risk of transmission after exposure to HIV

	Substance	Type of contact	Source person
**Risk**	• Blood<br>• Semen<br>• Vaginal fluid<br>• Cerebro-spinal fluid<br>• Pleural fluid<br>• Amniotic fluid<br>• Synovial fluid<br>• Ascites fluid	• Skin penetrated with contaminated needle (hollow or non-hollow)<br>• Large amount of substance on mucous membrane<br>• Sexual intercourse no condom<br>• Risk substance on lacerated skin / open wound	• Known HIV infected<br>• Unknown HIV status<br>• Recently tested negative (may be in window period)
**No Risk**	• Urine<br>• Stool<br>• Pus<br>• Tears<br>• Saliva<br>• Sputum<br>• Nasal secretions	• Risk substance on intact skin	

Immediate measures

- Remove infectious substance
  - Wash exposed wounds and skin sites thoroughly with soap
  - Flush mucous membranes with water
  - Do not use bleach, antiseptics or other caustic substances

Eligibility to start PEP (ARV prophylaxis)

- Any exposure classified as risk in the last 72 hours (see **Table 16**)
- Never refuse PEP on moral judgement about the kind of exposure (accident, negligence, rape, 'burst condom')
- New HIV test is mandatory to confirm negative HIV status
  - BUT: Don't delay starting PEP if HTC is not immediately available (no test kits, night, etc.). Do HTC as soon as possible.
- PEP is safe in pregnancy and breastfeeding
- Severe anaemia (<8g/dl) is contraindication for standard PEP regimen (AZT/3TC)
  - Alternative PEP regimen: Stavudine / Lamivudine (d4T / 3TC) tablets 150mg 12-hourly for 30 days

How to start PEP

- Start taking PEP as soon as possible after high risk exposure, ideally within 2 hours.
- Starting PEP more than 72 hours after exposure is not effective and should not be done.
  - However, still do HTC at baseline, at 3 and 6 months.
- Explain dosage and importance of adherence
- Advise to return immediately if side effects are suspected

- Advise all exposed adults to practice safe sex until confirmed HIV negative at 3 months.
  - Give 30 condoms and re-supply as requested
- Do not stop breastfeeding
- Write case details in PEP register (to be revised 2011)

Table 17: Post exposure prophylaxis regimens and dosage

	Standard		Alternative	
**Weight**	AZT 60mg / 3TC 30mg paed. tabs	AZT 300mg / 3TC 150mg adult tabs	d4T 6mg / 3TC 30mg paed. tabs	d4t 30mg /3TC 150mg adult tabs
**3.0 – 5.9 kg**	1 tab 12-hourly		1 tab 12-hourly	
**6 – 9.9 kg**	1 ½ tabs 12-hourly		1 ½ tabs 12-hourly	
**10 – 13.9 kg**	2 tabs 12-hourly		2 tabs 12-hourly	
**14 – 19.9 kg**	2 ½ tabs 12-hourly		2 ½ tabs 12-hourly	
**20 – 24.9 kg**	3 tabs 12-hourly		3 tabs 12-hourly	
**≥ 25.0 kg**		1 tab 12-hourly		1 tab 12-hourly

PEP follow-up

- At 30 days: completion of ARV prophylaxis,
  - Assess adherence
  - Give 60 condoms
- At 3 months: repeat HTC
- At 6 months: repeat HTC

Additional prevention measures after rape / sexual exposure

- Give emergency contraception (EC) within 72 hours if needed (see **Table 18**)
  - Repeat dose if vomiting occurs within 1 hour of taking EC
  - Explain that next menstrual period should occur before or around the expected time.
- Consider giving presumptive treatment for STIs using **Table 19**

Table 18: Regimens and dose for emergency contraception

Contraceptive drug	Immediately	After 12 hours
Postinor 2 (750µg levonorgesterel)	2 tablets	
OR		
Lo-Feminal or Microgynon	4 tablets	4 tablets

Table 19: Dosing of standard presumptive STI treatment after sexual exposure

STI drug	Child <15 years	Adult
Benzathine pen. vials	50,000 IU/kg IM stat (max 2.4 million IU)	2.4 Mega Units IM stat
Gentamicin vials	7.5 mg/kg IM stat (max 240mg)	240mg IM stat
Erythromycin tabs	12.5 mg/kg 6-hourly for 14 days (max 500 mg per dose)	500mg 6-hourly for 7 days
Metronidazole tabs	5 mg/kg 8-hourly for 7 days (max 2 grams per day)	2g stat
Nystatin pessaries	N/A	100,000 units 12 hourly for 7 days

7 Transition to the new PMTCT / ART regimens

Moving to the new PMTCT regimen

- AZT and sdNVP will no longer be dispensed as soon as TDF/3TC/EFV (Regimen 5A) becomes available at the respective facility
- Women on any of the previous PMTCT prophylaxis regimens should stop taking it and start lifelong TDF/3TC/EFV (Regimen 5A):
  - Stop AZT combination prophylaxis. Start Regimen 5A without gap.
  - Instruct women who were given take home sdNVP not to take it.
  - Women arriving in labour after taking AZT in pregnancy stop AZT or AZT/3TC and immediately start TDF/3TC/EFV (Regimen 5A) for life.
- Women who started ART (d4T/3TC/NVP, Regimen 1A) while pregnant continue on this regimen
- Start all breastfeeding HIV infected women on ART (even if they have taken one of the previous PMTCT prophylaxis regimens during pregnancy and/or delivery)
- Bring back any remaining ARVs to the clinic for controlled disposal.

Moving to new ART regimens

New ART initiation (and re-initiation after interruption) of children and adults

- Use Table 9 on page 42 to select the right start regimen for the respective patient

Transition for patients already on ART

- **Figure 5** on page 71 shows how and when to change patients from the old to the new regimens. Use alternative regimens if the patient has specific contraindications against the new standard regimen.
- Calculate current age for all children returning for ART follow-up visits after 1st July 2011.
- Change all children who are currently under 15 years to one of the new paediatric regimens (see below)
- Use **Table 10** on page 43 to select the right formulation and dosage depending on the child's weight. Heavier children will be given the adult formulation of the respective regimen.
- Change all adults on the old standard 2nd Line ART (AZT / 3TC + TDF + LPV/r) to the new standard 2nd Line (TDF / 3TC + LPV/r, Regimen 7A).

Figure 5: ART regimen transition for children and adults in Phase 1 (July 2011) and Phase 2 (to be announced by MOH circular)

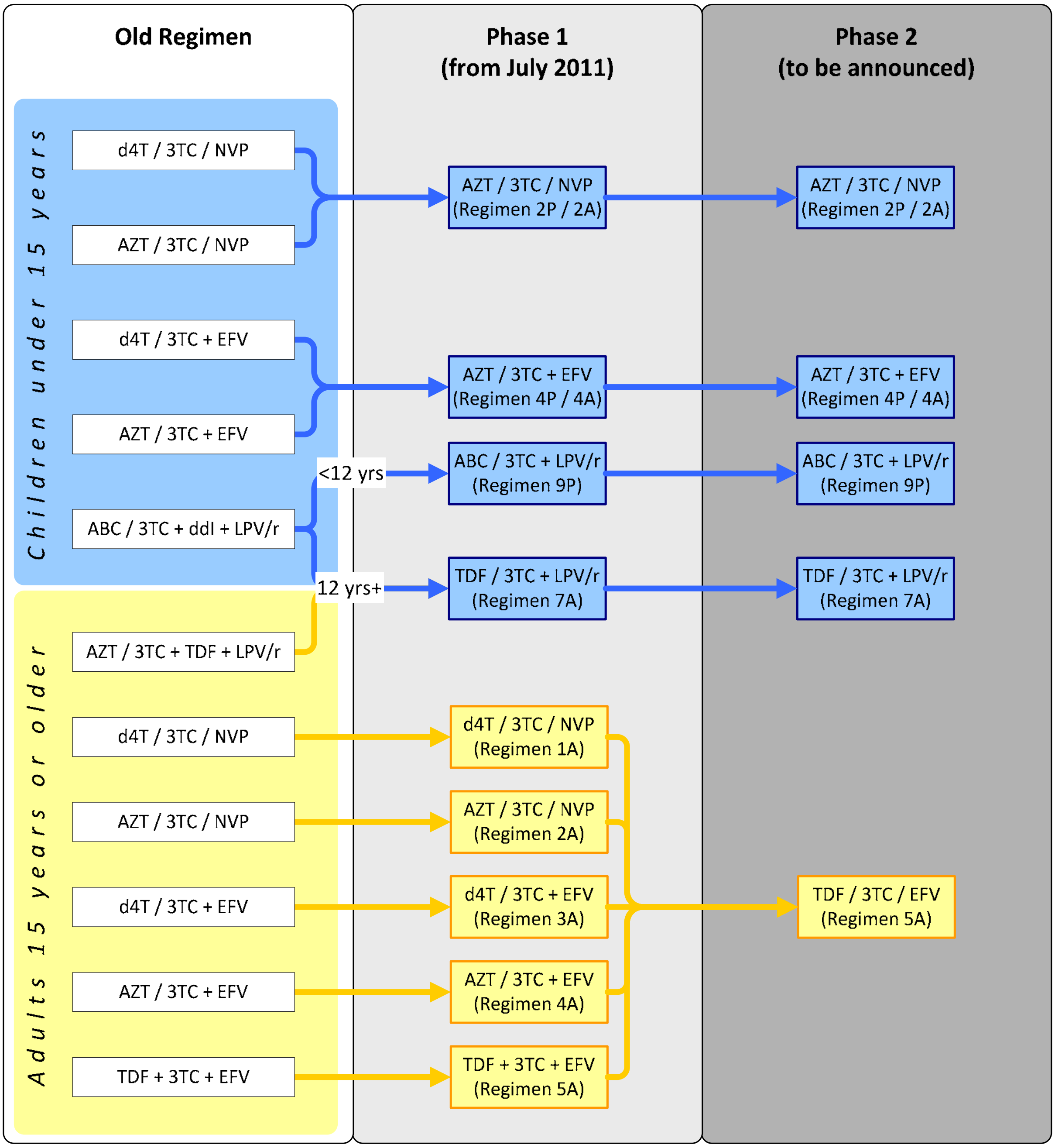

8 Monitoring and Evaluation

Key Facts for Providers

- HIV programs rely heavily on accurate and timely data for planning, reporting to donors and for drug procurement.
- Data analysis and reporting is done from patient cards and clinic registers at most facilities, but electronic systems for monitoring will increasingly be used at sites with many patients.
- Reporting is done monthly for ANC, maternity and exposed child follow-up and quarterly for pre-ART and ART (see Table 20 on page 75)
- Cohort analyses are needed to report outcomes of patients in ANC, exposed child, pre-ART and ART follow-up. Cohort reports look at the current / latest status of all patients enrolled in follow-up and require a review of all patient records to classify primary and secondary outcomes before data can be aggregated for reporting.
- Reports from facilities are to be completed within 5 working days after the end of the reporting period
- HIV Program reporting will be further integrated into the regular Health Management Information System. Monthly / quarterly facility reports will be entered directly into the District Health Information System at the District Health Offices for national reporting.

8.1 Definitions

PMTCT site

- A facility is counted as a PMTCT site if they have initiated on ART at least one pregnant or breast feeding woman during the reporting period.
- Depending on the mode of integration of PMTCT/ART interventions into the general health services, ART initiation may be initiated in any of the following service points: ART, ANC, maternity, post-natal or under 5 clinic.

ART site

- A facility is counted as an ART site if they had retained at least on patient alive on ART at the end of the reporting period.

ART status at registration

- Refers to the patient’s status at the time of first registration at this ART clinic – this status will never change as long as the patient remains at this clinic.
- **First time initiation:** Never taken ART (triple ARV combination treatment) in the past. Having taken ARVs for prophylaxis (PEP, single dose nevirapine, AZT combination prophylaxis for PMTCT) does NOT count as having taken ART and is ignored for the *ART status at registration*.

- **Re-initiation**: Received ART (triple ARV combination for treatment) from another ART site in the past but has NOT been taking it for 2 weeks or more as of the day of registering at this clinic. Patients who have interrupted for 2 weeks or more need to take a starter pack for re-initiation (if started on a regimen containing NVP).
- **Transfer in**: Received ART from another ART site in the past and is currently taking ART or has interrupted for less than 2 weeks. Count as *Transfer In* regardless if the patient brings his old Patient Card or not ('official' or 'unofficial' transfer).

Defaulted / Lost to follow-up

- Patients are counted as 'defaulted' in the cohort report if they have not returned to the clinic and are not known to have transferred out, stopped or died.
- The following times apply in the different clinics:
  - HCC (HIV exposed children and pre-ART patients): 2 months after the *Next Appointment Date* given at the last visit
  - ART: 2 months after the patient is expected to have run out of ARVs
- Patients may revert to 'alive on ART' at the next cohort analysis if they returns to the clinic and continue (pre-)ART

ART stop

- Patients are counted as 'stopped' if they are last known to be alive and have stopped taking ART. Stop is used regardless:
  - of the reason the patient has stopped (clinician's or patient's own decision)
  - if the ART interruption is intended to be permanent or temporary
  - of the duration of the ART interruption at the time of doing the cohort analysis
- Patients may revert to 'alive on ART' at the next cohort analysis if they re-start ART

Died

- Patients are counted as 'died' if there is a reliable report about the patient's death. 'Died' is used regardless:
  - of the cause of death (HIV- or non-HIV related disease, accident, suicide or homicide)
  - if the patient was on ART or not at the time of death

ART re-start

- Interrupted ART for more than 2 months while registered at the respective ART site. Update the number of re-starts in the ART clinic register whenever the patient re-started ART after defaulting or stopping for more than 2 months (i.e. returns after 'defaulting'). Patients who have interrupted for 2 weeks or more need to take a starter pack for re-initiation (if started on a regimen containing NVP).

ART adherence level

- Reporting of adherence levels is based on a classification of the number of doses missed at the last visit before the end of the quarter evaluated.

- The translation of the number of doses missed into adherence % depends on the number of days since the last visit. In practice, it is too complicated to consider varying intervals when analysing cohort adherence. Therefore, 2 monthly visits are assumed for all when classifying adherence for reporting.
- Patients who are supposed to take 2 tablets per day (e.g. Regimen 1A) and who have missed more than 6 doses are classified as 'less than 95% adherent'.
- Patient who are supposed to take 1 tablet per day (e.g. Regimen 5A) and who have missed more than 3 tablets are classified as 'less than 95% adherent'.

Table 20: Overview of M&E systems for integrated HIV program reporting

Service	M&E tools		Report cycle	Report elements			
	Patient card	Register		New registrations	Cohort outcomes		
					Definition of cohort	Primary outcomes	Secondary outcomes
**ANC**	–	ANC Clinic Register	Monthly	New first visits	• Registration group (6 months after first ANC visit)	–	(Final status at end of ANC)<br>• HIV test status<br>• On ART
**Maternity**	–	Maternity Register	Monthly	New deliveries	–	–	–
**ART**	ART Patient Card (separate cards for Paediatric and Adult Formulations)	ART Clinic Register	Quarterly	Patients newly registered at ART clinics	• Cumulative (all ever registered)<br>• Registration group (survival analysis)	• Alive on ART<br>• Died<br>• Defaulted<br>• Stopped ART<br>• Transferred out	• ART regimen / formulation<br>• Adherence level<br>• Side effects<br>• TB status<br>• On CPT<br>• Using FP
**Pre-ART**	HIV Care Patient Card, Pre-ART Child/Adult	HIV Care Clinic Register	Quarterly	Patients newly registered at HCC	• Cumulative (all ever registered)	• Alive in pre-ART care<br>• Started ART<br>• Transferred out<br>• Defaulted<br>• Died	• TB status<br>• On IPT<br>• On CPT<br>• Using FP
**Exposed child FUP**	HIV Care Patient Card, Exposed Child Under 24 Months	HIV Care Clinic Register	Monthly	Patients newly registered at HCC	• Birth cohort (children who (would) have turned 2, 12 and 24 months of age)	• Alive in exp. child FUP<br>• Discharged uninfected<br>• Started ART<br>• Defaulted<br>• Transferred out<br>• Died	• Age when received DNA-PCR result<br>• Latest HIV status

8.2 Reporting of registration data

- For all new patients registered, baseline data (such as age at registration, sex, pregnancy status, clinical stage, etc.) are recorded on patient treatment cards and copied into the clinic register
- These details do not change over time and tallying of these data needs to be done only once when reporting on new patients registered during the reporting month or quarter
- *Page summaries* in the clinic registers are filled as soon as each page is full. Count the number of circled values for each column on the page
- **Monthly** or **quarterly registration reports** are obtained by adding the page summaries from each page in the respective reporting month or quarter
- **Cumulative registration reports** are obtained by adding the data from the new monthly or quarterly registration report to the data from the previous cumulative registration report
- Data elements in most sections should add up to the respective total number of patients registered. Examples:
    - Males, non-pregnant females and pregnant females must add up to the total number registered
    - Age groups must add up to the total number registered
    - ART status (first time initiations, re-initiations, and transfer ins) must add up to the total number registered
- Some registration data (such as the number of patients with KS at the time of ART initiation) are counted separately and are not part of a section. These data elements are not expected to add up to the total number registered.

8.3 Reporting of cohort outcomes

- *Cohort analyses* are needed to measure outcomes of patients in follow-up.
- In principle, the outcome status of any patient ever registered can change at any time. Therefore, the records of all patients ever registered have to be reviewed each time a cumulative cohort outcome analysis is done. Current outcome data can not be obtained by addition from the previous quarterly outcome data.
- Patient outcomes are considered as of the last day of the reporting period. Any events (e.g. death) that happened after that day are ignored in the respective cohort analysis, but will be counted in the next report.

Primary follow-up outcome

- The primary outcome shows if a patient has been retained alive in care or if he has dropped out and why
- The primary outcome categories must add up to the total patients registered in the cohort
- Table 20 lists the primary follow-up outcomes used for the different reports
- For ART only, deaths are further classified according the time after ART initiation. The categories used are: death within 1st, 2nd, 3rd month after ART initiation or after 3rd month of ART initiation.

Secondary outcome

- Secondary outcomes are the latest treatment details of the patients retained alive in care
- Secondary outcomes are counted directly from the cards of the patients retained alive in care, usually by looking at the last visit before the end of the month or quarter evaluated. This visit might be several months before the end of the quarter, for example if the patient is on long ARV dispensing intervals (as long as the patient is still classified as 'retained alive in care' at the end of the quarter evaluated).
- Each set of secondary outcome categories must add up to the total number of patients retained alive in care.
- Table 20 shows the secondary outcomes used for the different reports

Definition of cohorts for different program reports

- 3 slightly different methods are used to define cohorts for outcome analyses:
- **Cumulative cohort** (Pre-ART and ART): Follow-up status of all patients ever registered at the respective clinic. The number of patients with adverse follow-up outcomes (death, default, etc.) inevitably increases over time. The number of patients retained in care is calculated by subtracting all patients with adverse follow-up outcomes from the total patient ever registered.
- **Registration group cohort** '*Survival analysis*' in ART: Follow-up status of patients registered during the quarters that ended 12, 24, 36, 48 and 60 months ago (ART). *ANC cohort outcomes:* final status as of the last ANC visit for the women who started ANC 6 months ago. This method standardises follow-up times and makes outcome data comparable between sites and over time.
- **Birth cohort** (HIV exposed child follow-up): Follow-up status of children who (would) have turned 2, 12 and 24 months old. Patient cards are filed in batches by month and year of birth (birth cohorts) and only the cards of children born 2, 12 and 24 months ago are pulled out for reporting. Outcomes are counted separately for the 2-, 12- and 24-month birth cohort. Reporting is done monthly and a different birth cohort is covered in each reporting month. This method standardises ages and is used for children enrolled in HIV exposed child follow-up.

8.4 Record keeping and filing

8.4.1 Confidentiality of patient records

- All patient cards and clinic registers are property of the MOH and may only be kept at the respective facility or at the National Archives
- Patient cards and clinic registers must be kept in a locked room and are only to be accessed by clinic staff responsible of providing the respective service and by the national supervision team. Patients and named guardians have access to their own patient card.

8.4.2 Use of clinic registers (ANC, Maternity, HCC, ART)

- Keep patient registration for each different service centralized in each facility: Use only one set of registers in each facility.
- Each patient has only one row[10] in each register: Continue using the same row for returning transfers and re-starts after default or stop.

[10] In the ANC register, each woman has one separate section with rows for each subsequent visit.

- Turn to a new page when starting to register patients in a new quarter. Leave any unused rows at the bottom of the previous page empty. This is necessary to separate the quarters when adding up page totals.
- Assign continuous registration numbers (by sequence of registration). Take care not to duplicate registration numbers.
    - Continue assigning cumulative registration numbers in the HCC- and ART-Register. These number series are never re-started.
    - Re-start assigning registration numbers annually for the ANC- and Maternity Register. Re-start with number 1 on the 1st of July.

8.4.3 Use of patient cards

- Each patient has only one patient card at any one time (Exposed child, pre-ART, ART). Attach another patient card once the old card is full
- Patient cards are filed in polythene sleeves in lever arch files, up to 100 cards per arch file.
- Separate filing systems are used for the different types of patient cards:

Exposed Child Under 24 Months Cards

- File in batches by year and month of birth
- Within each birth month, sort in ascending order by HCC registration number
- Do not remove the cards of children who have died, defaulted or transferred out from this filing system
- Files with birth cohorts who (would) have now reached at least age 3 years can be removed from the clinic for archiving.

Pre-ART Child / Adult Cards

- File cards in ascending order by HCC registration number
- Prepare separate filing systems for **ACTIVE** (retained in pre-ART care) and **INACTIVE** patients (started ART, transferred out, defaulted, died)
- One arch file can hold approximately 100 cards.
    - Label the spine of the **ACTIVE** files with HCC numbers 1-100, 101-200, 201-200, etc.
    - Label the spine of the **INACTIVE** files with HCC numbers 1-200, 201-400, 401-600, etc.
- Each time the quarterly cohort analysis is done, update in the HCC register the outcome for patients who have dropped out of pre-ART (started ART, transferred out, defaulted or died). Straight after this, move these cards of from the **ACTIVE** to the **INACTIVE** filing system.

ART Patient Cards, Paediatric and Adult ARV Formulations

- File ART Patient Cards in ascending order by ART registration number
- Prepare separate filing systems for **ACTIVE** (retained in ART) and **INACTIVE** patients (stopped ART, transferred out, defaulted, died)
- One arch file can hold approximately 100 cards.

- Label the spine of the **ACTIVE** files with ART numbers 1-100, 101-200, 201-200, etc.
- Label the spine of the **INACTIVE** files with ART numbers 1-200, 201-400, 401-600, etc.

- Each time the quarterly cohort analysis is done, update in the ART register the outcome for patients who have dropped out of ART (stopped ART, transferred out, defaulted or died). Straight after this, move these cards of from the **ACTIVE** to the **INACTIVE** filing system.
- Do not separate **Paediatric** and **Adult** cards into different files

8.5 Ensuring adequate data quality

- Use only the standard national reporting forms.
- The clinic's own analysis and reports are checked by the PMTCT/ART supervision team each quarter from primary records.
- Copies of the checked reports are kept at the clinic.

Index

3TC....39
abacavir hypersensitivity
  ART regimen substitution....43
ABC/3TC
  dosing....43
abdominal pain
  differential diagnosis....25
  differential diagnosis and management....60
active patient file....78
adherence
  appointment....52
  calculation....46
  classification for reporting....73
  counseling....54
  dose....54
age cohort....77
amitryptilline....61
anaemia
  ART regimen substitution....42
  starting ART....44
ANC
  standard HIV services delivered in....7
ANC register....77
appointment scheduling....46
archiving of patient records....77
ARM....63
ART
  adherence classification....73
  alternative regimens....42
  continuation....52
  dispensing....45
  'emergency dispensing'....45
  prescribing....45
  second line regimens....43
ART clinic
  standard HIV services delivered in....7
ART clinic register....77
ART contraindications....39
ART counselling
  group....50
  individual....50
ART dispensing
  calculation of quantities....46
  maximum supply....46
  patients on 2nd line regimens....46
  safety buffer....46
  supply and adherence calculation (table)....47
ART duration, monitoring of....54
ART eligibility
  adults and children over 5 years....29
  children 2-5 years of age....28
  definition....28
  infants under 12 months....28
ART failure
  confirmed....56
  suspected....55
ART initiation....48
  counselling....50
  labour....63
  preparing the patient....49
ART patient card....49, 78
  adult ARV formulation....78
  paediatric ARV formulation....78
ART prescriber level....39
ART regimen
  selecting regimen, formulation and dosage....38
  selection for continuation....53
  transition....71
  transition to new guidelines....70
ART regimens....37
  first line....37
  second line....37
  table....42
  third line....37
ART registration status definitions....72
ART re-start (M&E definition)....73
ART site definition....72
ART stop (M&E definition)....73
artificial rupture of membranes....63
ARV classification....38
ARV drug register....49
ARV formulations
  adult and paediatric formulations....39
  dosing table....43
ascertainment of HIV exposure in children....9
  flowchart....10
ascertainment of HIV status....9
AZT/3TC
  dosing....43
AZT/3TC/NVP
  dosing....43
BCG vaccination....63
birth cohort....77
blisters
  differential diagnosis....26
BMI
  classification....21
  definition....18
body pains
  differential diagnosis and management....58
breast infection....36
breastfeeding
  counseling....36
buffalo hump....23
bumpy itch.... *See* pruritic papular eruptions
cancer....60
CD4 monitoring
  sample collection....27
  schedule....27
cervical cancer
  diagnosis and management....15
clinical monitoring checklist....22
cohort analysis....75
  defintion of cohorts for reporting....77
  registration groups....77
condoms
  routine dispensing....31
confidentiality of patient records....77
confirming HIV infection....49
  testing protocol....49

confusion 61
cotrimoxazole preventive therapy 32
cough
differential diagnosis 24
CPT 32
contraindications 33
dosage 33
duration 33
eligibility 32
cryptococcal meningitis
management 16
cumulative reporting
outcome data 77
registration data 76
d4T/3TC
dosing 43
d4T/3TC/NVP
dosing 43
death (M&E definition) 73
defaulter definition 73
delivery
management 63
Depo-provera 31
diarrhoea
differential diagnosis 25
differential diagnosis and management 60
diarrhoea, chronic
management 14
died (M&E definition) 73
DNA-PCR
timing 5
doses missed
classification for reporting 73
doses missed (ART) 52
drowsiness 61
efavirenz
skin rash 42
teratogenicity 40
EFV
dosing 43
emergency ARV dispensing register 45
emergency contraception 68
emergency dispensing (ART) 45
exposed child follow-up
enrolment 63
Exposed child follow-up
standard HIV services delivered in 7
exposed child under 24 months patient card 78
failure to thrive
differential diagnosis 23
family planning 30
Family planning clinic
standard HIV services delivered in 7
fattening
diagnosis and management 58
FDC 38
fever
differential diagnosis 25
differential diagnosis and management 58
filing systems (M&E) 77
first time initiation (ART registration status) 72
fixed dose combination 38
glands, swollen
differential diagnosis 23
growth curve 18
hanging tablets 46
HCC
concept 6
HCC register 77
hepatitis 60
ART regimen substitution 42
starting ART 44
herpes simplex 24
herpes zoster
management 16
HIV Care Clinic 6
HIV dementia 23
HIV exposed child follow-up
appointment schedule 5
HIV exposure
risk classification 66
HIV testing
schedule in children 11
HIV-related diseases
clinical management 14
monitoring 52
immune reconstitution inflammatory syndrome 62
implementation plan 3
inactive patient file 78
infant and child feeding 7, 36
In-patient department
standard HIV services delivered in 7
insecticide treated bed nets 7, 35
integration
clinical HIV services 5
standard package of services 7
interaction
ART and TB treatment 51
IPT 34
contraindications 34
dispensing schedule 5
dosage and duration 35
eligibility 34
ruling out of active TB 34
IRIS 62
differential diagnosis 62
management 62
isoniazid preventive therapy 7, 34
ITN .... *See* insecticide treated bed nets, *See* insecticide treated bed nets
jaundice
differential diagnosis 23
starting ART 44
Kaposi sarcoma
management 15
lab investigations 50
labour
management 63
starting ART 44
lactic acidosis 59
ART regimen substitution 42
lamivudine 39
leg pain
differential diagnosis 25
differential diagnosis and management 61
lipodystrophy
ART regimen substitution 42
indications for change of ART regimen 42
liquid suspension (ART) 40
loss to follow-up (ART) 57
loss to follow-up definition 73
LPV/r

dosing 43
lymphadenopathy
differential diagnosis 23
M&E 72
malaria 60
master cards 78
Maternity
standard HIV services delivered in 7
maternity register 77
missed dose (ART) 45
monitoring and evaluation 72
mouth sores
differential diagnosis 24
MUAC
mid upper arm circumference 18
mid upper arm circumference in pregnant women .. 19
nausea
differential diagnosis 23
differential diagnosis and management 59
neck stiffness
differential diagnosis 23
needle stick injury 67
nephropathy
differential diagnosis 61
nevirapine prophylaxis 64
dispensing 64
dosing 65
timing and duration 65
newborn care 63
night sweats
differential diagnosis 25
nightmares 61
NNRTI 38
NRTI 38
nutrition status
BMI 18
monitoring 18
weight-for-height 18
nutritional monitoring 52
oesophageal candidiasis
management 14
OPD
HIV services delivered in 7
OPV 63
oral candidiasis
management 14
oral hairy leukoplakia 24
oral polio vaccination 63
outcome
ART follow-up 57
outcome data (reporting) 76
page summary 78
page summary (reporting) 76
patient cards 78
PCP
management 16
PEP 66
peripheral neuropathy
ART regimen substitution 42
differential diagnosis 25
differential diagnosis and management 61
PI 38
PIFP 30
pill count (ART) 52
PITC 8
pleural effusion
differential diagnosis 24
PMTCT regimen transition 70
PMTCT site defintion 72
pneumocystis carinii (jiroveci) pneumonia (PCP)
management 16
pneumonia
management 17
poor adherence 54
post exposure prophylaxis 66
eligibility for ARV prophylaxis 67
immediate measures 67
postnatal care 63
Postnatal clinic
standard HIV services delivered in 7
Pre-ART
standard HIV services delivered in 7
Pre-ART follow-up
appointment schedule 5
pre-ART patien card 78
pregnancy, first trimester
starting ART 44
presumed severe HIV disease
ART eligibility 28
definition 13
presumptive STI treatment 69
prevention with positives 31
preventive services for HIV patients 30
primary outcome 76
primary outcomes 75
protease inhibitors 38
provider initiated family planning 30
provider initiated testing and counseling 8
pruritic papular eruptions
management 16
PSHD
definition 13
psychiatric disorder (EFV side effect)
ART regimen substitution 42
PwP 31
rape 68
rash
differential diagnosis 26
reasons for starting ART
hierarchy of criteria 29
Regimen 5A (TDF/3TC/EFV)
rationale forusing in pregnant women 40
registration data (reporting) 76
registration numbers 78
re-initiation (ART registration status) 73
renal failure
ART regimen substitution 42
starting ART 44
reporting
outcome data 76
registration data 76
reporting cycle 75
re-start ART (M&E definition) 73
reverse **t**ranscriptase **i**nhibitor 38
seborrhoeic dermatitis
management 16
secondary outcome 75, 77
sepsis
management 17
shingles
management 16
shortness of breath

differential diagnosis 24
skin rash
ART regimen substitution 42
differential diagnosis and management.................. 59
slimming 23
differentialdiagnosis and management................... 58
standard monitoring of HIV patients 18
starter pack
rationale 39
schedule of next appointment 46
when to give and when not to give 39
Stevens-Johnson-Syndrome 26, 39
STI treatment, presumptive 69
stopping ART
indications 52
substitution (ART regimen)
strategy 40
survival analysis.. 77
swelling (face)
differential diagnosis and management.................. 61
tail (stopping ART)
rationale 39
when not to give 39
TB
management 14
TB clinic
standard HIV services delivered in 7
TB screening, routine 53
TB treatment
combining with ART 51
TB, active
starting ART 44
TDF/3TC
dosing 43
TDF/3TC/EFV
dosing 43
tenofovir
bone growth 39
tinea corporis / cruris / pedis
management...16
toxicity (ART, severe) 39
transfer in (ART registration status) 73
transition (new guidelines) 70
umbilical cord cutting 63
Under 5 clinic
standard HIV services delivered in.............................7
universal ART
children 12-24 months of age 28
children under 12 months of age.............................28
vacuum extraction 63
veins, protruding 23
viral load
interpretation 56
monitoring 56
monitoring schedule 56
routine monitoring..56
targeted 56
vomiting
differential diagnosis 23, 25
differential diagnosis and management 59
vomiting (LPV/r side effect)
ART regimen substitution 43
weakness
differential diagnosis and management 58
weight loss
differential diagnosis 23
Weight loss >10%...19
weight-for-height
classification..20
nutrition monitoring in children 18
WHO clinical staging 12
criteria for adults 13
criteria for children 13
yellow eyes
differential diagnosis 23
differential diagnosis and management 60

First Edition | July 2011 | Ministry of Health, Malawi
